# Crotofolane Diterpenoids and Other Constituents Isolated from *Croton kilwae*

**DOI:** 10.1021/acs.jnatprod.2c01007

**Published:** 2023-02-07

**Authors:** Emanuel T. Mahambo, Colores Uwamariya, Masum Miah, Leandro da Costa Clementino, Luis Carlos Salazar Alvarez, Gabriela Paula Di Santo Meztler, Edward Trybala, Joanna Said, Lianne H. E. Wieske, Jas S. Ward, Kari Rissanen, Joan J. E. Munissi, Fabio T. M. Costa, Per Sunnerhagen, Tomas Bergström, Stephen S. Nyandoro, Mate Erdelyi

**Affiliations:** † Chemistry Department, College of Natural and Applied Sciences, University of Dar es Salaam, P.O. Box 35061, Dar es Salaam, Tanzania; ‡ Department of Infectious Diseases/Virology, Institute of Biomedicine, Sahlgrenska Academy, University of Gothenburg, S-413 46 Gothenburg, Sweden; ∥ Laboratory of Tropical Diseases - Prof. Dr. Luiz Jacinto da Silva, Department of Genetics, Evolution, Microbiology and Immunology, Institute of Biology (IB), University of Campinas - UNICAMP, Campinas, 13083-970 SP, Brazil; § Department of Chemistry and Molecular Biology and Centre for Antibiotic Resistance Research (CARe), University of Gothenburg, SE-405 30 Gothenburg, Sweden; ⊥ Department of Chemistry, University of Jyvaskyla, Survontie 9B, 40014 Jyväskylä, Finland; △ Department of Chemistry − BMC, Uppsala University, SE-751 23 Uppsala, Sweden

## Abstract

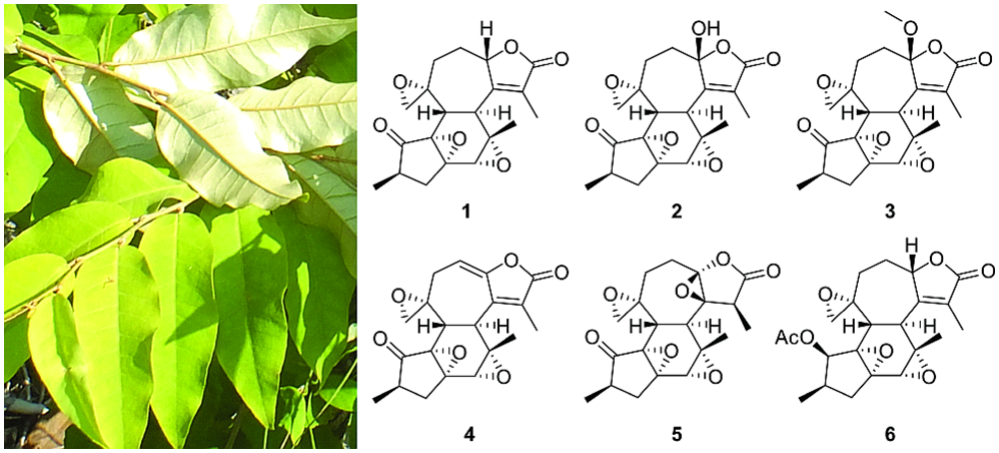

Six new crotofolane diterpenoids (**1**–**6**) and 13 known compounds (**7**–**19**) were isolated from the MeOH–CH_2_Cl_2_ (1:1, v/v) extracts of the leaves and stem bark of *Croton kilwae*. The structures of the new compounds were elucidated by extensive analysis of spectroscopic and mass spectrometric data. The structure of crotokilwaepoxide A (**1**) was confirmed by single-crystal X-ray diffraction, allowing for the determination of its absolute configuration. The crude extracts and the isolated compounds were investigated for antiviral activity against respiratory syncytial virus (RSV) and human rhinovirus type-2 (HRV-2) in HEp-2 and HeLa cells, respectively, for antibacterial activity against the Gram-positive *Bacillus subtilis* and the Gram-negative *Escherichia coli*, and for antimalarial activity against the *Plasmodium falciparum* Dd2 strain. *ent*-3β,19-Dihydroxykaur-16-ene (**7**) and ayanin (**16**) displayed anti-RSV activities with IC_50_ values of 10.2 and 6.1 μM, respectively, while exhibiting only modest cytotoxic effects on HEp-2 cells that resulted in selectivity indices of 4.9 and 16.4. Compounds **2** and **5** exhibited modest anti-HRV-2 activity (IC_50_ of 44.6 μM for both compounds), while compound **16** inhibited HRV-2 with an IC_50_ value of 1.8 μM. Compounds **1**–**3** showed promising antiplasmodial activities (80–100% inhibition) at a 50 μM concentration.

The genus *Croton* (Euphorbiaceae) comprises approximately 1300 species occurring in the tropical and subtropical regions of the world,^1^ of which 17 can be found in Tanzania.^2,3^*Croton* species are used widely in folk medicine in Tanzania to treat worm infections, colds, stomachache, constipation, malaria, tuberculosis, ear infections, and cancer.^4^ The phytochemical investigations of various members of the genus *Croton* have revealed terpernoids,^5^ alkaloids,^6^ flavonoids,^7^ and diterpenoids.^4−15^ The diterpenoids include clerodanes,^4^ kauranes,^12^ crotofolanes,^8−11^ labdanes,^13^ cembranes,^14^ and abietanes,^15^ some of which have exhibited cytotoxic,^16^ anti-inflammatory,^12^ antifungal,^17^ acetylcholinesterase inhibition,^18^ and neurite outgrowth-promoting properties.^19^

*Croton kilwae* Radcl.-Sm. is a plant species endemic to Tanzania and Mozambique. In Tanzania, it grows in the Kilwa District of the Lindi Region.^20^ The leaf morphology of *C. kilwae* resembles *C. dichogamus* Pax and *C. menyhartii* Pax.^20^ Chemical analysis of the leaf constituents of *C. dichogamus* led to the isolation of several crotofolanes, a rare class of ditepernoids that so far have been reported from a few *Croton* species.^8,9,11,21,22^ The genus *Croton* has previously yielded interesting secondary metabolites, which makes *C. kilwae* a suitable addition to our ongoing phytochemical investigation of *Croton* species native to Tanzania. Herein we report the isolation and structure determination of six new crotofolane diterpenoids (**1**–**6**) along with 13 known compounds (**7**–**19**) and evaluation of their antiviral, antibacterial, antiplasmodial, and cytotoxic activities.
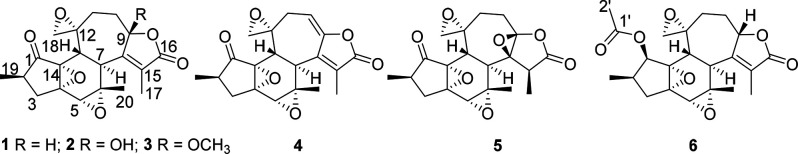


## Results and Discussion

The MeOH–CH_2_Cl_2_ (1:1 v/v) extracts of the leaves and stem bark of *C. kilwae* were separately subjected to repeated silica gel 60 (230–400 mesh) column chromatography, followed by gel filtration on a Sephadex LH-20 column and/or HPLC. The stem bark extract yielded one new crotofolane (**1**) and six known compounds (**7**–**12**), while the leaf extract afforded six new crotofolanes (**1**–**6**) and seven known compounds (**13**–**19**, Figure S1, Supporting Information). The structures were characterized by the analysis of their spectroscopic data, including the single-crystal X-ray diffraction analysis of compound **1**. The known compounds *ent*-3β,19-dihydroxykaur-16-ene (**7**),^23^^24^*ent*-3β-hydroxykaur-16-en-19-oic acid (**8**),^25^*ent*-16β,17-dihydroxykauran-19-oic acid (**9**),^25^*ent*-3β,16α,17-trihydroxykaurane (**10**),^26^ 16β,17-dihydroxykaurane (**11**),^27^*ent*-3β-hydroxykaur-16-ene (**12**),^28^ quercetin-3-rhamnose-4,7-dimethyl ether (**13**),^29^ 3,7,4′-tri-*O*-methylkaempferol (**14**),^30^ 3,7,3′,4′-tetra-*O*-methyl quercetin (**15**),^31^ ayanin (**16**),^32^ stigmasterol (**17**),^33^ the pinane-type monoterpenoids 2-hydroxy-2-(hydroxymethyl)-6,6-dimethylbicyclo[3.1.1]heptan-3-one (**18**),^34^ and *p*-hydroxyphenylethyl ferute (**19**)^35^ were identified by comparison of their spectroscopic data (Figures S49–S145, Supporting Information) to those reported in the literature.

Compound **1**, [α]^24^_D_ −6 (*c* 0.1, CHCl_3_), was obtained from both the stem bark and the leaf extracts as a white solid. Its molecular formula was established as C_20_H_23_O_6_ based on the HRESIMS ([M + H]^+^ at *m*/*z* 359.1483, calcd 359.1495; Figure S9, Supporting Information) and NMR data (Table 1). The IR spectrum showed absorption bands at 1737 cm^–1^ (lactone carbonyl) and at 1666 cm^–1^ (C=C bond stretch). The ^1^H NMR spectrum (Figure S2, Supporting Information) displayed a characteristic signal for an oxo-allylic proton at δ_H_ 4.92 (H-9) of a crotofolane^8^ and for the presence of three sets of methyl groups at δ_H_ 1.08 (H-20), 1.11 (H-19), and 1.97 (H-17) (Table 1). The ^13^C NMR spectrum (Figure S3, Supporting Information) displayed 20 carbons, of which two were assigned to carbonyl groups of a ketone (δ_C_ 211.0, C-1) and lactone (δ_C_ 173.3, C-16), two nonprotonated olefinic carbons [δ_C_ 161.0 (C-8), 128.4 (C-15)], and four oxygenated quaternary carbons [δ_C_ 64.2 (C-14), 61.4 (C-4), 58.4 (C-12), and 56.7 (C-6)]. The combination of ^13^C NMR and HSQC (Figures S3 and S5, Supporting Information) experiments further revealed the presence of an oxo-allylic carbon resonating at δ_C_ 82.0 (C-9), four methylene carbons at δ_C_ 57.6 (C-18), 34.1 (C-11), 32.5 (C-10), and 34.2 (C-3), three tertiary carbons at δ_C_ 36.9 (C-2), 38.0 (C-7), and 36.8 (C-13), and one epoxy carbon with a single proton at δ_C_ 57.4 (C-5).

**Table 1:** NMR Spectroscopic Data (500 MHz, CDCl_3_) of Crotokilwaepoxide A (**1**)

position	δ_C_, type	δ_H_ (*J* in Hz)	HMBC^a^
1	211.0, C		
2	36.9, CH	2.58 ddq (8.5, 8.4, 7.1)	C-1, C-3, C-19
3	34.2, CH_2_	1.68 dd (14.0, 8.5)	C-2, C-4, C-5, C-19
		2.92 dd (14.0, 8.4)	C-1, C-2, C-4, C-14
4	61.4, C		
5	57.4, CH	3.10 s	C-3, C-4, C-6, C-14, C-20
6	56.7, C		
7	38.0, CH	3.13 d (13.2)	C-6, C-8, C-9, C-12,
			C-13, C-14, C-15, C-16, C-20
8	161.0, C		
9	82.0, CH	4.92 dd (11.0, 3.7)	C-8, C-10, C-15, C-16
10	32.5, CH_2_	1.54 dddd (13.6, 12.7, 11.0, 4.4)	C-8, C-9, C-11, C-12, C-18
		2.47 dddd (12.7, 4.4, 3.7, 3.7)	C-8, C-9, C-11, C-12
11	34.1, CH_2_	1.61 ddd (13.8, 4.8, 3.7)	C-9, C-10, C-12, C-13, C-14
		2.17 ddd (13.8, 13.6, 4.4)	C-9, C-10, C-12, C-13
12	58.4, C		
13	36.8, CH	2.60 d (13.2)	C-6, C-7, C-8, C-11, C-12, C-14
14	64.2, C		
15	128.4, C		
16	173.3, C		
17	10.0, CH_3_	1.97 s	C-6, C-7, C-8, C-9, C-10, C-15, C-16
18	57.4, CH_2_	2.79 m	C-11, C-12, C-13
19	13.0, CH_3_	1.11 d (7.1)	C-1, C-2, C-3
20	20.0, CH_3_	1.08 s	C-4, C-5, C-6, C-7, C-8, C-15

a HMBC correlations, optimized for 6 Hz, are from the stated proton(s) to the indicated carbon.

**Figure 1:**
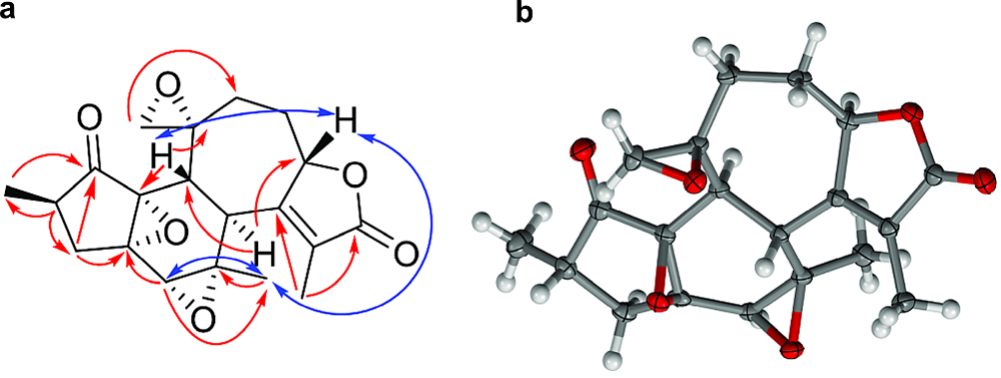
(a) Key HMBC (red) and NOESY (blue) correlations and (b) the X-ray crystal structure of crotonkilwaepoxide A (**1**) (thermal ellipsoids at 50% probability, H_2_O solvate omitted for clarity).

The HMBC cross peaks (Figure 1a and Figure S6, Supporting Information) of H-17 (δ_H,_ 1.97) to C-15 (δ_C_ 128.4), C-8 (δ_C_ 161.0), and C-16 (δ_C_ 173.3) indicated the presence of a methyl butenolide moiety constituting an α,β-unsaturated γ-lactone unit. The COSY and TOCSY spectra (Figures S4 and S7, Supporting Information) showed correlations between H-9 (δ_H_ 4.92) and H-17 (δ_H_ 1.97), with scalar *J* < 1 Hz as expected for long-range coupling, between H-9 and H-10 (δ_H_ 1.54, 2.47), and between H-10 (δ_H_ 1.54, 2.47) and H-11 (δ_H_ 1.61, 2.17), H-7 (δ_H_ 3.13), and H-13 (δ_H_ 2.60). This, combined with the HMBC cross peaks of H-10 (δ_H_ 1.54, 2.47) and H-11 (δ_H_ 1.61, 2.17) to C-9 (δ_C_ 82.0) and C-12 (δ_C_ 58.4) as well as H-11 (δ_H_ 1.61, 2.17) to C-13 (δ_C_ 36.8), and of H-7 (δ_H_ 3.13) to C-9 (δ_C_ 82.0), C-15 (δ_C_ 128.4), and C-8 (δ_C_ 161.0) established the seven-membered ring as being fused via C-8–C-9 of the α,β-unsaturated γ-lactone moiety.

Furthermore, the HMBC cross peaks of H-18 (δ_H_ 2.79) to C-11 (δ_C_ 34.1), C-12 (δ_C_ 58.4), and C-13 (δ_C_ 36.8) and those of H-11 (δ_H_ 1.61, 2.17) to C-12 (δ_C_ 58.4) and C-18 (δ_C_ 57.6) indicated an epoxide group fused to the seven-membered ring. The HMBC cross peaks of H-13 (δ_H_ 2.60) to C-14 (δ_C_ 64.2) and of H-7 (δ_H_ 3.13) to C-6 (δ_C_ 56.7), as well as H-20 (δ_H_ 1.08) to C-6 (δ_C_ 56.7) and C-5 (δ_C_ 57.4), in combination with those of H-5 (δ_H_ 3.10) to C-4 (δ_C_ 61.4) and C-6 (δ_C_ 56.7) confirmed the fused six-membered ring via C-7–C-13, with the deshielded nonprotonated carbons C-4 (δ_C_ 61.4), C-12 (δ_C_ 58.4), C-6 (δ_C_ 56.7), and C-14 (δ_C_ 62.4) assigned to the corresponding epoxide carbons. Further analysis of the HMBC spectrum revealed correlations between H-19 (δ_H_ 1.11) and C-1 (δ_C_ 211.0), C-2 (δ_C_ 36.9), and C-3 (δ_C_ 34.2), and those between H-2 (δ_H_ 2.58) and C-1 (δ_C_ 211.0) and C-14 (δ_C_ 62.4) combined with H-3 (δ_H_ 2.92) and C-1 (δ_C_ 211.0), C-4 (δ_C_ 61.4), and C-14 (δ_C_ 62.4) suggested the presence of a cyclopentanone unit fused to a six-membered ring via C-4–C-14. The COSY and TOCSY spectra confirmed the cyclopentanone moiety through the observed correlations involving H-19 (δ_H_ 1.11), H-2 (δ_H_ 2.58), and H-3 (δ_H_ 1.68, 2.92).

The relative configuration in compound **1** was established based on scalar coupling constants (Table 1) and NOESY correlations (Figure S8, Supporting Information). NOEs between the methyl protons H-20 (δ_H_ 1.08) and H-5 (δ_H_ 3.10) and H-9 (δ_H_ 4.92), as well as between H-9 (δ_H_ 4.92) and H-13 (δ_H_ 2.60), indicated these protons to be *syn*-oriented. This was consistent with the *trans*-disposed bridgehead protons H-7 (δ_H_ 3.13) and H-13 (δ_H_ 2.60), both with ^3^*J*_HH_ = 13.2 Hz. To determine the absolute configuration and structure of this compound, single-crystal X-ray crystallographic analysis was performed (Figure 1b).^36,37^ The absolute configuration of **1** was determined by refinement of the Flack parameter,^36,37^ which gave the unambiguous value of 0.09(5). The configuration established for this compound is similar to previously reported crotofolanes.^11^ Based on the spectroscopic data, this new compound (**1**) was identified as a crotofolane diterpenoid bearing C-4–C-14, C-5–C-6, and C-12–C-18 epoxide structural motifs and was given the trivial name crotokilwaepoxide A (**1**).

**Figure 2:**
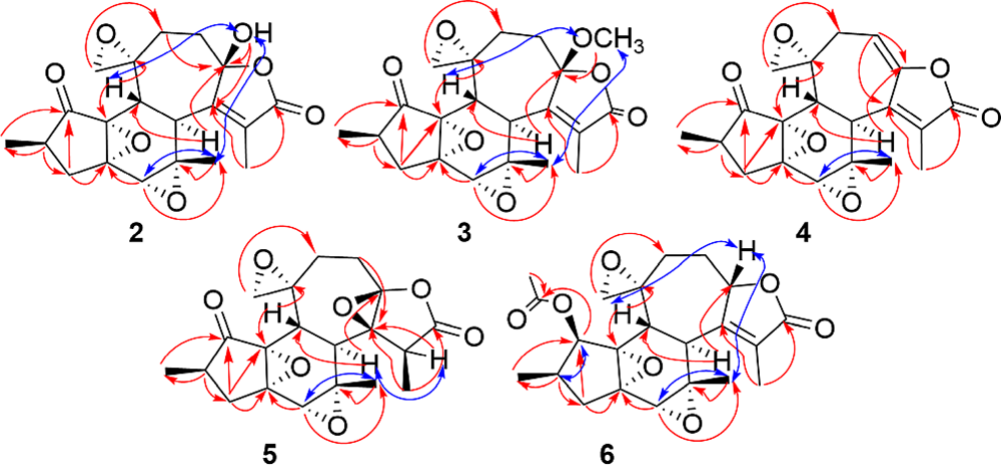
Key HMBC (red) and NOESY (blue) correlations of **2**–**6**.

Compound **2**, [α]^24^_D_ −8 (*c* 0.1, CHCl_3_), was isolated from the leaf extract as a white solid. Its molecular formula, C_20_H_23_O_7_, was based on the HRESIMS ([M + H]^+^*m*/*z* 375.1429, calcd 375.1444 and [M – H_2_O]^+^*m*/*z* 357.1323; Figure S17, Supporting Information) and NMR data (Table 2). The IR spectrum showed sharp absorption bands indicative of carbonyl bond stretches of a ketone and γ-lactone at 1728 and 1761 cm^–1^, respectively, and an intense absorption band for a hydroxy group at 3445 cm^–1^. Comparison of NMR spectra (Figures S10–S16, Supporting Information) of **2** with those of **1** revealed their structural similarity. The absence of an oxo-allylic proton H-9 (δ_H_ 4.92), in combination with the large chemical shift change for C-9, which resonated at δ_C_ 107.8 in **2** instead of δ_C_ 82.0 as observed for compound **1**, suggested the presence of a hydroxy group at position C-9, forming a ketal carbon. This was confirmed by the HMBC cross peaks of proton H-7 (δ_H_ 3.04), H-11 (δ_H_ 2.38, 1.51), H-10 (δ_H_ 2.38, 1.93), and OH-9 (δ_H_ 4.32) to C-9 (δ_C_ 107.8) (Figure 2 and Figure S14, Supporting Information). The relative configurations of the stereocenters of compound **2** were found to be the same as those of compound **1**, based on the similar NOE correlations of protons H-5 (δ_H_ 3.12), H-13 (δ_H_ 3.16), and OH-9 (δ_H_ 4.32) to H-20.

**Table 2:** NMR Spectroscopic Data (500 MHz, CDCl_3_) of 9-Hydroxycrotokilwaepoxide A (**2**)

position	δ_C_, type	δ_H_ (*J* in Hz)	HMBC^a^
1	211.4, C		
2	36.9, CH	2.56 ddq (8.7, 8.4, 7.1)	C-1, C-3, C-4, C-19
3	34.1, CH_2_	1.71 dd (13.9, 8.7)	C-2, C-4, C-5, C-19
		2.90 dd (13.9, 8.4)	C-1, C-2, C-4, C-5, C-14
4	61.5, C		
5	57.7, CH	3.12 s	C-4, C-20
6	57.2, C		
7	38.0, CH	3.04 dd (12.7, 1.1)	C-6, C-8, C-9, C-13, C-15
8	158.5, C		
9	107.8, C		
OH-9		4.32 s	
10	36.7, CH_2_	1.93 ddd (14.0, 13.0, 7.4)	C-9, C-11, C-12
		2.38^b^ m	C-8, C-9, C-11, C-12
11	32.9, CH_2_	1.51 m	C-8, C-9, C-10, C-12, C-13, C-14, C-19
		2.38^b^ m	C-8, C-9, C-10, C-12
12	58.6, C		
13	35.7, CH	3.16 d (12.8)	C-6, C-7, C-8, C-11, C-12, C-14
14	64.3, C		
15	130.3, C		
16	171.1, C		
17	9.9, CH_3_	1.95 d (1.1)	C-6, C-7, C-8, C-10, C-15, C-16
18	57.8, CH_2_	2.79, s	C-11, C-12, C-13
19	13.1, CH_3_	1.11 d (7.1)	C-1, C-2, C-3
20	20.4, CH_3_	1.18 s	C-5, C-6, C-7, C-15

a HMBC correlations, optimized for 6 Hz, are from the stated proton(s) to the indicated carbon.

b Protons with overlapping resonances

Compound **3**, [α]^24^_D_ +60 (*c* 0.1, CHCl_3_), was obtained as a white solid from the leaf extract. The HRESIMS displayed a [M + H]^+^ peak at *m*/*z* 389.1584 (calcd 389.1600, Figure S25, Supporting Information), corresponding to a molecular formula of C_21_H_24_O_7_. The IR spectrum showed a band characteristic for a carbonyl functionality at 1762 cm^–1^. The NMR spectra (Figures S18–S24, Supporting Information) and the extracted data (Table 3) of **3** were similar to those of compounds **1** and **2**. In contrast to **1** and **2**, **3** exhibited a methoxy group at position C-9 (δ_C_ 109.8), instead of a proton or a hydroxy group. The position of OCH_3_-9 (δ_H_ 3.55, δ_C_ 52.0) was confirmed by the HMBC cross peak of OCH_3_-9 (δ_H_ 3.55) to the ketal carbon C-9 (δ_C_ 109.8) (Figure 2 and Figure S22, Supporting Information). Therefore, compound **3** was identified as the new crotofolane 9-methoxycrotokilwaepoxide A (**3**), being an *O*-methylated derivative of compound **2**.

**Table 3:** NMR Spectroscopic Data (500 MHz, CDCl_3_) of 9-Methoxycrotokilwaepoxide A (**3**)

position	δ_C_, type	δ_H_ (*J* in Hz)	HMBC^a^
1	211.3, C		
2	36.9,CH	2.57, ddq (8.4, 8.4, 7.1)	C-1, C-3, C-19
3	34.2, CH_2_	1.69 dd (13.9, 8.4)	C-2, C-4, C-5, C-19
		2.90 dd (13.9, 8.4)	
4	61.4, C		
5	57.8, CH	3.09 s	C-3, C-4, C-6, C-20
6	57.3, C		
7	38.0, CH	3.02 d (12.8)	C-6, C-8, C-9, C-13, C-14, C-15
8	158.7, C		
9	109.8, C		
OMe-9	52.0, CH_3_	3.55 s	C-9
10	30.5, CH_2_	1.75 ddd (15.0, 13.9, 4.0)	C-9, C-11, C-12
		2.67 ddd (15.0, 3.9, 3.6)	C-8, C-9, C-11, C-12
11	32.9, CH_2_	1.49 ddd (13.8, 4.0, 3.6)	C-9, C-10, C-13, C-18
		2.15 ddd (13.9, 13.8, 3.9)	C-9, C-10, C-12, C-13
12	58.4, C		
13	35.7, CH	3.07 d (12.8)	C-7, C-8, C-11, C-12, C-14, C-18
14	64.3, C		
15	129.9, C		
16	170.8, C		
17	10.0, CH_3_	1.95 s	C-6, C-7, C-8, C-9, C-10, C-15, C-16
18	57.9, CH_2_	2.78 s	C-11, C-12, C-13
19	13.1, CH_3_	1.11 d (7.1)	C-1, C-2, C-3
20	20.5, CH_3_	1.15 s	C-6, C-7

a HMBC correlations, optimized for 6 Hz, are from the stated proton(s) to the indicated carbon.

Compound **4**, [α]^24^_D_ +36 (*c* 0.07, CHCl_3_), was isolated from the leaf extract as a white solid. Its molecular formula, C_20_H_21_O_6_, was based on the HRESIMS ([M + H]^+^*m*/*z* 357.1324, calcd 357.1338; Figure S33, Supporting Information) and NMR data (Table 4). The IR spectrum showed bands at 1660 and 1738 cm^–1^, which are indicative for ketone and γ-lactone moieties, respectively. The NMR spectra (Figures S26–S32) of **4** suggested it to have a similar carbon structural scaffold to compounds **1**–**3**. However, the spectra for compound **4** differed from those of **1**–**3** for signals associated with C-9 (δ_C_ 149.3) and C-10 (δ_C_ 109.4, δ_H_ 5.81), indicative of an endocyclic double bond between C-9 and C-10, presumably formed via enzymatically mediated dehydration of compound **2**. The positions of the carbons generating these signals were confirmed by the HMBC (Figure 2 and Figure S30, Supporting Information) cross peaks of H-11 (δ_H_ 2.17, 3.21), H-7 (δ_H_ 3.14), and H-10 (δ_H_ 5.81) to C-9 (δ_C_ 149.3) and those of H-11 (δ_H_ 2.17, 3.21) and H-18 (2.83, 2.87) to C-10 (δ_C_ 109.4). Moreover, coupling between the olefinic proton H-10 (δ_H_ 5.81) and the diastereotopic protons H-11 (δ_H_ 3.21, 2.17) was observed in the COSY spectrum (Figure S28, Supporting Information). The relative configurations of **4** were determined to be the same as those of compounds **1**–**3** for the stereogenic centers these compounds have in common (Figure 2 and Figure S32, Supporting Information). Thus, this new compound, crotokilwaepoxide B (**4**), was characterized as the C-9–C-10 dehydrated derivative of compound **2**.

**Table 4:** NMR Spectroscopic Data (500 MHz, CDCl_3_) of Crotokilwaepoxide B (**4**)

position	δ_C_, type	δ_H_ (*J* in Hz)	HMBC^a^
1	210.9, C		
2	37.1, CH	2.59 ddq (8.5, 8.4, 7.1)	C-1, C-3, C-19
3	34.2, CH_2_	1.71 dd (13.9, 8.5)	C-2, C-4, C-5, C-19
		2.93 dd (13.9, 8.4)	C-1, C-2, C-4, C-14
4	61.4, C		
5	58.0, CH	3.10 s	C-3, C-4, C-6, C-14, C-20
6	57.3, C		
7	38.7, CH	3.14 dq (12.7, 1.6)	C-6, C-8, C-9, C-13, C-14, C-15, C-16, C-20
8	147.2, C		
9	149.3, C		
10	109.4, CH	5.81 ddd (6.9, 2.9, 0.9)	C-8, C-9, C-11, C-12, C-14
11	38.0, CH_2_	2.17 dd (16.9, 6.9)	C-8, C-9, C-10, C-12, C-13, C-14, C-15, C-18
		3.21 ddd (16.9, 2.9, 1.0)	C-9, C-10, C-12
12	57.1, C		
13	36.6, CH	2.79 dd (12.7, 1.0)	C-4, C-7, C-8, C-12, C-14
14	64.6, C		
15	130.7, C		
16	169.4, C		
17	11.1, CH_3_	2.09 dd (1.6, 0.9)	C-6, C-7, C-8, C-9, C-10, C-15, C-16
18	56.5, CH_2_	2.83 d (3.9)	C-10, C-11, C-12
		2.87 d (3.9)	C-4, C-11, C-12, C-14
19	13.0, CH_3_	1.13 d (7.1)	C-1, C-2, C-3
20	19.6, CH_3_	1.12 s	C-5, C-6, C-7, C-8, C-13, C-15

a HMBC correlations, optimized for 6 Hz, are from the stated proton(s) to the indicated carbon.

Compound **5**, [α]^24^_D_ −29 (*c* 0.1, CHCl_3_), was isolated from the leaf extract as a white solid. Its molecular formula, C_20_H_23_O_7_, was determined from the HRESIMS (*m*/*z* 375.1432 [M + H]^+^, calcd 375.1444; Figure S41, Supporting Information) and NMR data (Table 5). The IR spectrum revealed absorption bands at 1741 and 1799 cm^–1^, which could be attributed to a ketone and lactone functionality, respectively. Analysis of the NMR spectra (Figures S34–S40, Supporting Information) of **5** revealed it to have a crotofolane skeleton similar to compounds **1**–**4**. However, contrary to the NMR spectroscopic data for **1**–**3** that indicated an α,β unsaturated γ-lactone moiety in each case, the ^13^C NMR spectrum (Figure S35, Supporting Information) of **5** suggested the saturation of C-15 (δ_C_ 45.5) and C-8 (δ_C_ 65.8). In addition, the COSY spectrum (Figure S36, Supporting Information) indicated an isolated coupling between the CH_3_-17 methyl protons (δ_H_ 1.48, d, *J* = 7.5 Hz) and the H-15 proton (δ_H_ 3.14, q, *J* = 7.5 Hz). The chemical shift of C-8 (δ_C_ 65.8) and that of C-9 (δ_C_ 89.9) were consistent with epoxidation at C-8 and C-9, which was confirmed by the HMBC cross-peaks (Figure 2 and Figure S38, Supporting Information) of protons H-15 (δ_H_ 3.14), H-10 (δ_H_ 2.18, 2.78), H-17 (δ_H_ 1.48), and H-7 (δ_H_ 2.44) to C-8 (δ_C_ 65.8) as well as H-10 (δ_H_ 2.18, 2.78) and H-11 (δ_H_ 2.64) to C-9 (δ_C_ 89.9). The relative configuration of C-8 and C-9 could not be determined from the NMR data obtained. However, the relative configuration at C-15 for **5** was established through the NOE interaction of H-15 (δ_H_ 3.14) with H-7 (δ_H_ 2.44). The other configurations of the stereogenic centers of **5** were established to be identical to **1**, based on similar NOE correlations for the two compounds observed (Figure 2 and Figure S40, Supporting Information). This new compound, crotokilwaepoxide C (**5**), was therefore characterized as the C-8–C-9 epoxy derivative of **1**.

**Table 5:** NMR Spectroscopic Data (500 MHz, CDCl_3_) of Crotokilwaepoxide C (**5**)

position	δ_C_, type	δ_H_ (*J* in Hz)	HMBC^a^
1	210.8, C		
2	37.0, CH	2.54 ddq (8.4, 8.3, 7.2)	C-1, C-3, C-19
3	34.2, CH_2_	1.68 dd (14.0, 8.4)	C-2, C-4, C-5, C-19
		2.90 dd (14.0, 8.3)	C-1, C-2, C-4, C-14
4	61.6, C		
5	58.0, CH	3.02 s	C-3, C-4, C-6, C-14, C-20
6	56.9, C		
7	39.8, CH	2.44 d (13.0)	C-5, C-6, C-8, C-13, C-14, C-15, C-20
8	65.8, C		
9	89.9, C		
10	24.7, CH_2_	2.18 ddd (15.7, 12.9, 7.7)	C-9, C-11
		2.78 ddd (15.7, 6.2, 1.3)	C-8, C-9, C-11, C-12
11	33.6, CH_2_	1.61 ddd (15.1, 12.9, 6.2)	C-10, C-12, C-13
		2.64 ddd (15.1, 7.7, 1.3)	C-9, C-10, C-12, C-18
12	58.6, C		
13	32.8, CH	2.54 d (13.0)	C-6,C-7, C-8, C-11, C-12, C-14
14	64.1, C		
15	45.5, CH	3.14 q (7.5)	C-7, C-8, C-16, C-17
16	175.7, C		
17	10.5, CH_3_	1.48 d (7.5)	C-8, C-15, C-16
18	52.4, CH_2_	2.72 d (4.0)	C-7, C-11, C-12
		2.78 d (4.0)	C-10, C-13
19	13.2, CH_3_	1.10 d (7.2)	C-1, C-2, C-3
20	21.6, CH_3_	1.48 s	C-5, C-6, C-7

a HMBC correlations, optimized for 6 Hz, are from the stated proton(s) to the indicated carbon.

Compound **6**, [α]^24^_D_ −20 (*c* 0.05, CHCl_3_), was obtained from the leaf extract as a colorless oil with the molecular formula C_22_H_27_O_7_, as established by HRESIMS ([M + H]^+^ at *m*/*z* 403.1744, calcd 403.1757; Figure S49, Supporting Information) and NMR data (Table 6). The IR spectrum showed a band at 1744 cm^–1^, which was indicative of a carbonyl group. The NMR spectra (Figures S42–S48, Supporting Information) of **6** resembled those of **1** except for the presence of an additional oxymethine proton signal at δ_H_ 5.53 and that of a deshielded methyl group at δ_H_ 2.19, which were assigned to H-1 and H-2′, respectively, with their corresponding carbons at δ_C_ 75.6 and 20.9. The ^13^C NMR spectrum (Figure S43, Supporting Information) showed one more additional signal at δ_C_ 170.0 (C-1′), with HMBC to H-1 (δ_H_ 5.53) and H-2′ (δ_H_ 2.19) (Figure 2 and Figure S46, Supporting Information), indicating the formation of an acetate moiety at C-1 instead the carbonyl group as present for compounds **1**–**5**. The position of the acetate group was confirmed by the HMBC cross peaks of proton H-3 (δ_H_ 2.45) and H-19 (δ_H_ 0.95) to C-1 (δ_C_ 75.6). The relative configuration at C-1 for **6** was based on the NOE correlation between H-1 (δ_H_ 5.53) and H-2 (δ_H_ 2.15) (Figure 2 and Figure S48, Supporting Information), along with their scalar coupling constant (^3^*J*_HH_ = 5.4 Hz) that indicated a *syn* orientation. The other configurations at the stereogenic centers of **6** were similar to those of **1** and of another related compound previously confirmed by X-ray crystallographic analysis.^21^ Therefore, the new compound crotokilwaepoxide D (**6**) was characterized as the C-1 acetoxy derivative of **1**. Crotofolanes **1**–**6** are similar to the crotocascarins published by Kawakami et al.^22^ and have identical configurations. However, their side chains are different, and they have an epoxide at C-12–C-18 instead of an alkene.

**Table 6:** NMR Spectroscopic Data (500 MHz, CDCl_3_) of Crotokilwaepoxide D (**6**)

position	δ_C_, type	δ_H_ (*J* in Hz)	HMBC^a^
1	75.6, CH	5.53 d (5.3)	C-3, C-4, C-14, C-1′
2	33.1, CH	2.15^b^ dddd (10.3, 7.2, 7.0, 5.3)	
3	36.4, CH_2_	1.63 dd (13.8, 10.3)	C-2, C-4, C-19
		2.45^b^ (13.8, 7.2)	C-1, C-4, C-14
4	61.0, C		
5	57.4, CH	3.10 s	C-4, C-6, C-14, C-20
6	55.9, C		
7	38.1, CH	3.12 dd (12.9, 1.3)	C-6, C-8, C-9, C-13, C-15, C-20
8	161.7, C		
9	81.9, CH	4.92 m	
10	32.5, CH_2_	1.51^b^m	C-7, C-9, C-11, C-12, C-13, C-18
		2.42^b^ m	C-8, C-9, C-11, C-12
11	34.6, CH_2_	1.50^b^ m	C-7, C-9, C-10, C-12, C-13, C-18
		1.75 ddd (15.0, 15.0, 4.4)	C-9, C-10, C-13
12	58.7, C		
13	37.4, CH	2.38 d (12.9)	C-6, C-7, C-8, C-11, C-12, C-14, C-18
14	66.8, C		
15	128.2, C		
16	173.4, C		
17	10.0, CH_3_	1.96 dd (1.4, 1.4)	C6, C-8, C-9, C-10, C-15, C-16
18	57.0, CH_2_	2.86 d (4.7)	C-11, C-12
		3.18, d (4.7)	C-10, C-11, C-12
19	12.4, CH_3_	0.95, d (7.0)	C-1, C-2, C-3
20	20.2, CH_3_	1.09, s	C-5, C-6, C-7
1′	170.0, C		
2′	20.9, CH_3_	2.19,^b^ s	C-1, C-1′

a HMBC correlations, optimized for 6 Hz, are from the stated proton(s) to the indicated carbon.

b Overlapping signals.

Crotofolanes are rare ditepernoids that have been previously reported from several *Croton* species including *C. corylifolious*,^24,38^*C. dichogamus*,^9,39^*C. haumanianus*,^21^*C. cascarilloides*,^10,11,22^*C. caracasanus*,^8^ and *C. megalocarpus*.^40^ This unusual skeleton has been hypothesized to be biosynthesized from geranyl pyrophosphate, which is subsequently transformed via cembrane, casbane, and lathyrane to crotofolanes.^10^ Compounds **1**–**6** presented in this study are multiepoxidized crotofolanes having the unprecedented epoxidation of the exocylic double bond found on the heptacyclic ring of the previously reported analogues, with compound **5** having an additional epoxide moiety. All ^13^C NMR chemical shifts for compounds **1**–**6** were in good agreement with those predicted by CSearch.^41^*C. kilwae* was recently reported to be in the same clade as *C. dichogamus*,^42^ one of the *Croton* species reported to biosynthesize crotofolanes.^3,9,39^ Therefore, the present findings provide additional insight into the chemotaxonomic relationships among *Croton* species, which warrant further investigations.

The crude extracts and the isolated compounds from *C. kilwae* were tested for antiviral activity against both HRV-2 and RSV. The antiviral activities against RSV are given in Table S1, and the dose response for the most active compounds (**7** and **16**) is presented in Figure S147 (Supporting Information). Compounds **7** and **16** showed anti-RSV activity with IC_50_ values of 10.2 and 6.1 μM, while marginal cytotoxic effects (CC_50_) on HEp-2 cells were observed, which resulted in selectivity index (SI; CC_50_/IC_50_) values of 4.9 and 16.4, respectively. Lopes et al. reported^43^ that acetylation of quercetin enhanced the virucidal activity of this compound against RSV particles. Since compound **16** is a 3,7,4′-tri-*O*-methylated derivative of quercetin, it was investigated for the occurrence of this type of activity. However, it exhibited no RSV particle inactivating (virucidal) activity at the doses tested. The anti-HRV-2 activity of selected compounds is shown in Table S2 (Supporting Information). Compounds **2** and **5** inhibited HRV-2 at relatively high concentrations only (IC_50_ of 44.6 μM for both, SI > 2.2). However, compound **16**, besides its anti-RSV activity, also exhibited anti-HRV-2 activity with an IC_50_ value of 1.8 μM. Using the MTS (tetrazolium)-based cytotoxicity assay no substantial toxicity of **16** for HeLa cells was observed at a concentration of ≤100 μM (SI > 55.6); however, microscopic observation revealed the presence of morphological alterations in HeLa cells treated with **16** at ≥20 μM (Table S2, Supporting Information). Compound **16** is also known for its antimicrobial activity against *Mycobacterium tuberculosis*.^44^ Crotokilwaepoxides **1**–**5** gave IC_50_ and CC_50_ values of >100 μM, which indicated a lack of both anti-RSV activity and cytotoxicity for HEp-2 cells at concentrations up to100 μM (Table S1, Supporting Information). Indeed, similar compounds have been shown to be nontoxic to the PC-3, HeLa, and MCF-7 human tumor cell lines, exhibiting CC_50_ values of >50 μM.^8^ In our hands, compounds **1**, **2**, **4**, and **5** showed no cytotoxicity at ≤100 μM for HeLa and HEp-2 cells. Similar compounds have recently been reported to inhibit HIV-1 replication,^3,40^ indicating the biomedical potential of these types of natural products. Neither the crude extracts nor the isolated pure compounds showed antibacterial activities against *Bacillus subtilis* or *Escherichia coli* at a ∼2 mM concentration.

**Figure 3:**
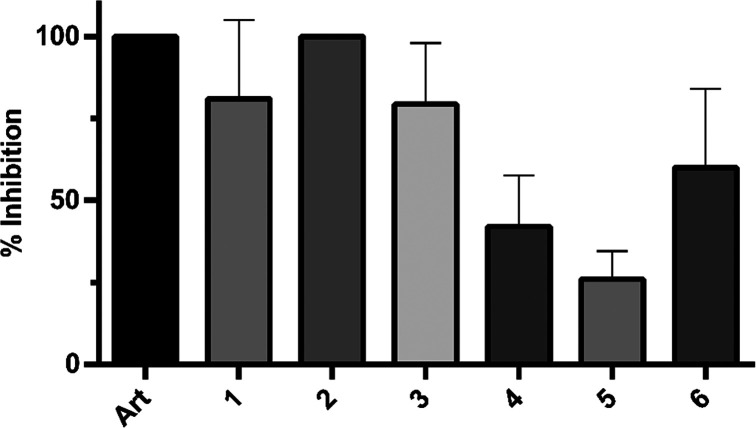
In vitro growth inhibition of asexual blood stage *P. falciparum* (Dd2) for crotofolanes **1**–**6**. The inhibitory potential was tested at a concentration of 50 μM, and the inhibition of parasitemia was measured after 72 h of incubation. The data are from at least two independent experiments shown as the average ± standard deviation (artesunate and **2** gave 100% inhibition for all replicates, and therefore no standard deviation is given). Art: artesunate, 5 μM.

Compounds **1**–**6** were tested against chloroquine-resistant^45^*Plasmodium falciparum* Dd2 cells at 50 μM (Figure 3 and Table S3, Supporting Information). The most active compounds, **1**–**3**, inhibited parasite growth at 80–100%, whereas compounds **4**, **5**, and **6** controlled parasitemia at 26%, 42%, and 60%, respectively. For all compounds, low (<10%) or no hemolysis was observed (Figure S142, Supporting Information). The present results suggest that crotofolane diterpenoids may be interesting compound scaffolds for antimalarial drug development.

In conclusion, six new crotofolanes (**1**–**6**) and 13 known compounds (**7**–**19**) were isolated from *C. kilwae* leaf and stem bark extracts. Compounds **7** and **16** showed anti-RSV activity, and compounds **2**, **5**, and **16** inhibited HRV-2 in HeLa cells. Compounds **1**–**3** displayed antiplasmodial activities at a 50 μM concentration. The isolation of crotofolane diterpenoids from *C. kilwae* is of chemotaxonomic significance.

## Experimental Section

### General Experimental Procedures

Optical rotations were measured using a 341 LC OROT polarimeter (589 nm, 24.0 °C). UV spectra were obtained in CH_2_Cl_2_ using a Shimadzu UV-1650PC UV/vis spectrophotometer. Infrared (IR) spectra were recorded on a PerkinElmer Spectrum FT-IR instrument using liquid/solid samples. NMR spectra were acquired on a Bruker Avance NEO 500 MHz NMR spectrometer equipped with a 5 mm TXO cryogenic probe and were processed using the MestReNova (v14.0.0) software. Chemical shifts were referenced to the residual carbon and proton signals of the deuterated solvents (CDCl_3_ δ_H_ 7.26 and 77.2 or CD_3_OD δ_H_ 4.87 and 49.0) as internal standards. HRESIMS spectra were obtained with a Q-TOF-LC/MS spectrometer using a 2.1 × 30 mm 1.7 μM RPC18 and H_2_O–CH_3_CN gradient (5:95–95:5 in 0.2% formic acid, v/v) at Stenhagen Analys Lab AB, Gothenburg, Sweden. Silica gel 60 (230–400 mesh) and Sephadex LH-20 (GE Healthcare) were used for column chromatography. Percolated silica gel plates (Merck F_254_) were used for thin layer chromatography, which were visualized under UV light at 254 nm and stained with 4-anisaldehyde (7:5:8:180; volume ratio of 4-anisaldehyde, concentrated H_2_SO_4_, glacial acetic acid, and MeOH), followed by gentle heating, to visualize UV-inactive compounds and characteristic color changes of UV-positive spots. Preparative reversed-phase (RP)-HPLC was performed on a VWR LaPrep P110 instrument with single-wavelength detection, 220 nm, using a chiral column, Phenomenex Lux Amylose-1 column (5 μm, 1000 Å, ⦶ 21.2 mm, L 250 mm), with isocratic elution using CH_3_CN–H_2_O (3:7, v/v) as mobile phase and a flow-rate of 15 mL/min. The method was optimized on an analytical HPLC instrument (VWR LaChrome Elite system) with a Phenomenex Lux Amylose-1 column (5 μm, 1000 Å, ⦶ 4.6 mm, L 100 mm) and a flow-rate of 1.0 mL/min.

### Plant Material

The stem bark and leaves of *C. kilwae* were collected in November 2018 along a road to Rushungi (4 km outside of the village) at GPS location S 09°27.55.1 E 039°36.17.3 at an elevation of 103 m in the Kilwa District of the Lindi Region, Tanzania. The plant was identified and authenticated by F. M. Mbago, a senior taxonomist of the herbarium at the Botany Department of the University of Dar es Salaam, where a voucher specimen (FMM 3904) was deposited.

### Extraction and Isolation

The air-dried and pulverized leaves (1.8 kg) and stem bark (1.2 kg) were extracted with MeOH–CH_2_Cl_2_ (1:1) twice for 48 h at room temperature. Upon concentration of each extract using a rotatory evaporator at 40 °C, 130 and 100 g of leaf and stem bark crude extracts, respectively, were obtained. The dried leaf extract (130 g) was subjected to silica gel column chromatography and eluted with a gradient system of EtOAc–*n*-hexane (10:90 to 100 EtOAc) followed by 5% MeOH in EtOAc to afford 140 fractions, which were combined into nine pooled fractions (chronologically labeled FL1–FL9) based on TLC characteristics. Compound **1** (8 mg) was isolated from FL8 after further purification by Sephadex column chromatography LH-20 (MeOH–CH_2_Cl_2_, 1:1) and subsequent purification of fractions 11–24 by another silica gel column (MeOH–CH_2_Cl_2_, 1:4). Compounds **2** (6.4 mg) and **4** (4.3 mg) were obtained after FL6 was subjected to Sephadex column chromatography LH-20 (MeOH–CH_2_Cl_2_, 1:1) and further purification by gel column chromatography (EtOAc–CH_2_Cl_2_, 1:4). FL4 yielded compounds **3** (3.8 mg) and **18** (6 mg) after purification by Sephadex column chromatography LH-20 (MeOH–CH_2_Cl_2_, 1:1) and subsequent silica gel chromatography (EtOAc–CH_2_Cl_2_, 0.5:9.5). Compound **3** was obtained after preparative TLC (MeOH–EtOAc–*n*-hexane, 1:3:6) of subfractions 4–7 eluted from the second silica gel column. Compound **15** (25 mg) was isolated from FL2 after further purification by Sephadex LH-20 column chromatography (MeOH–CH_2_Cl_2_, 1:1) and the washing of subfractions 11–18 with *n*-hexane. Compound **7** (2 mg) was obtained after purification of fraction FL3 by Sephadex LH-20 column chromatography (MeOH–CH_2_Cl_2_, 1:1). Compounds **5** (7.2 mg) and **14** (14 mg) were isolated from FL1 after purification of subfractions 7–21, obtained from the Sephadex LH-20 column (MeOH–CH_2_Cl_2_, 1:1), utilizing a second silica gel column (EtOAc–CH_2_Cl_2_, 1:9). From the second silica column, compound **14** was eluted as subfractions 1 and 2, whereas compound **5** required an additional purification step of subfractions 3–6 by further silica gel column chromatographic separation (EtOAc–CH_2_Cl_2,_ 0.5:9.5). Compounds **1** (3 mg) and **6** (2 mg) were obtained from FL7 after isolation by Sephadex LH-20 column chromatography (MeOH–CH_2_Cl_2_, 1:1), of which subfractions 8–19 were further purified by silica gel column chromatography (EtOAc–CH_2_Cl_2_, 3:7) and collection of subfractions 8–11. The final compounds were separated by RP-HPLC chromatography (CH_3_CN–H_2_O, 3:7) with retention times of 5.89 and 11.41 min for compounds **1** and **6**, respectively. Compounds **16** (20 mg) and **19** (5.7 mg) were obtained after purification of FL5 and FL9, respectively, by Sephadex LH-20 column chromatography (MeOH–CH_2_Cl_2_, 1:1).

The stem bark extract (100 g) of *C. kilwae* was fractionated by silica gel column chromatography with a gradient elution of an EtOAc–petroleum ether solvent system with increasing polarity from 10% EtOAc in petroleum ether to 100% EtOAc and then 5% MeOH in EtOAc, to afford 110 fractions. Based on TLC profiling, the obtained fractions were pooled into 14 fractions labeled FSB1–FSB14. Compounds **1** (5 mg), **9** (5 mg), and **10** (4.3 mg) were all isolated from FSB12 after purification of subfractions 30–44 using a second silica gel column (EtOAc–petroleum ether, 2:3) and a third silica gel column (EtOAc–CH_2_Cl_2_, 1:4). Compound **1** was obtained from washing subfractions 4–9 eluted from the third silica column with MeOH. Compound **9** was obtained after subjecting subfractions 60–80 from the third silica column to passage over another silica column (EtOAc–CH_2_Cl_2_, 1:4) and further purification using Sephadex LH-20 (100% MeOH). Isolation of compound **10** was achieved after further purification of subfractions 45–59 from the third silica column with an additional silica gel column (EtOAc–CH_2_Cl_2_, 1:4). Compounds **8** (4 mg) and **13** (5 mg) were obtained from fraction FSB11 after purification by silica gel column chromatography (EtOAc–petroleum ether, 2:3). Subfractions 57–66 were further purified by silica gel column chromatography (EtOAc–CH_2_Cl_2_, 1:4) and subsequent Sephadex LH-20 column chromatography (100% MeOH) to yield compound **8**. Subfractions 26–36 from the first silica gel column performed on FSB11 were subjected to Sephadex LH-20 column chromatography (MeOH–CH_2_Cl_2_, 1:1) followed by a final column chromatographic step on silica gel (EtOAc–CH_2_Cl_2_, 1:9) to afford compound **13**. Compound **17** (60 mg) was isolated from fraction FSB6 after further purification by silica gel column chromatography (EtOAc–petroleum ether, 1:4). Compound **11** (10 mg) was isolated from fraction FSB10 after purification by silica gel column chromatography (EtOAc–CH_2_Cl_2_, 1:9), from which subfractions 42–46 were further purified by Sephadex LH-20 column chromatography (MeOH–CH_2_Cl_2_, 1:1) and subsequent preparative TLC (EtOAc–CH_2_Cl_2_, 1:9). Compound **12** (6.3 mg) was obtained from fraction FSB5 after purification by Sephadex LH-20 gel column chromatography (MeOH–CH_2_Cl_2_, 1:1) and crystallization of subfractions 8–13 from MeOH.

#### Crotokilwaepoxide A (**1**):

white solid; [α]^24^_D_ −6 (*c* 0.1, CHCl_3_); IR ν_max_ 2930, 1737, 1666, 1444, 1096, 1018, 928, 905 cm^–1, 1^H and ^13^C NMR, see Table 1; HRESIMS *m*/*z* 359.1483, [M + H]^+^ (calcd *m*/*z* 359.1495 for C_20_H_23_O_6_).

#### 9-Hydroxycrotokilwaepoxide A (**2**):

white solid; [α]^24^_D_ −8 (*c* 0.1, CHCl_3_); IR ν_max_ 3445, 2936 1761, 1730, 1436, 1329, 954, 906 cm^–1^; ^1^H and ^13^C NMR, see Table 2; HRESIMS *m*/*z* 357.1323 [M – H_2_O]^+^ and *m*/*z* 357.1323 [M + H]^+^ (calcd *m*/*z* 374.1366 for C_20_H_23_O_7_).

#### 9-Methoxycrotokilwaepoxide A (**3**):

white solid; [α]^24^_D_ +60 (*c* 0.1, CHCl_3_); IR ν_max_ 2932, 1762, 1445, 1318, 1123, 969, 906 cm^–1^; ^1^H and ^13^C NMR, see Table 3; HRESIMS *m*/*z* 389.1584 [M + H]^+^ (calcd *m*/*z* 389.1600 for C_21_H_25_O_7_).

#### Crotokilwaepoxide B (**4**):

white solid; [α]^24^_D_ +36 (*c* 0.07, CHCl_3_); IR ν_max_ 2924, 1738, 1660, 1447, 1410, 1066, 907, 890 cm^–1^; ^1^H and ^13^C NMR, see Table 4; HRESIMS *m*/*z* 357.1324 [M + H]^+^ (calcd *m*/*z* 357.1338 for C_20_H_21_O_6_).

#### Crotokilwaepoxide C (**5**):

white solid; [α]^24^_D_ −29 (*c* 0.1, CHCl_3_); IR ν_max_ 2923, 2852, 1799, 1741, 1456, 1377, 970, 890 cm^–1^; ^1^H and ^13^C NMR, see Table 5; HRESIMS *m*/*z* 375.1432 [M + H]^+^ (calcd *m*/*z* 375.1444 for C_20_H_23_O_7_).

#### Crotokilwaepoxide D (**6**):

white solid; [α]^24^_D_ −20 (*c* 0.05, CHCl_3_); IR ν_max_ 2924, 1744, 1232, 1019, 801 cm^–1^; ^1^H and ^13^C NMR, see Table 6; HRESIMS *m*/*z* 403.1744 [M + H]^+^ (calcd *m*/*z* 403.1757 for C_22_H_27_O_7_).^36,37^

### X-ray Diffraction Analysis

SCXRD measurements were performed using a Rigaku SuperNova dual-source Oxford diffractometer equipped with an Atlas detector using mirror-monochromated Cu Kα (λ = 1.54184 Å) radiation. The data collection and reduction were performed using the program CrysAlisPro, and a Gaussian face index absorption correction method was applied.^46^ The structures were solved by intrinsic phasing (SHELXT)^47^ and refined by full-matrix least-squares techniques against *F*^2^ using all data (SHELXL).^47^ All non-hydrogen atoms were refined with anisotropic displacement parameters. Hydrogen atoms were constrained in geometric positions to their parent atoms using OLEX2.

### X-ray Crystallographic Data of Compound **1**

Diffraction-quality crystals were obtained from dichloromethane. Crystal data for compound **1**: 2(C_20_H_22_O_6_)·H_2_O, M = 734.77, colorless block, 0.18 × 0.31 × 0.46 mm, monoclinic, space group *I*2 (no. 5), *a* = 14.5703(2) Å, *b* = 8.6823(1) Å, *c* = 14.1008(2) Å, β = 103.077(1)°, *V* = 1737.54(4) Å^3^, *Z* = 2, *T* = 120.0(1) K, μ = 0.87 mm^–1^, *D*_calc_ = 1.404 g cm^3^, *F*(000) = 780, 7982 measured reflections (7.8° ≤ 2θ ≤ 152.6°), 3425 unique reflections (*R*_int_ = 0.017), which were used in all calculations. The final *R*_1_ was 0.031 (*I*_o_ > 2σ(*I*_o_)) and *wR*_2_ was 0.084 (all data). The Flack parameter^36,37^ was 0.09(5). The X-ray structure of **1** (CCDC-2209300) has been deposited at the Cambridge Crystallographic Data Centre.^48^ Copies of the data can be obtained, free of charge, on application to the Director, CCDC, 12 Union Road, Cambridge CB2 IEZ, UK (fax: + 44-(0)1223-336033 or email: deposit@ccdc.cam.ac.uk).

### Antiviral Assays

Human laryngeal epidermoid carcinoma (HEp-2) cells were used for the testing of antirespiratory syncytial virus (RSV) and the cytotoxic activities of both crude extracts and the pure compounds isolated therefrom, while the human uterine cervical cancer cells (HeLa) were employed for similar assays performed with human rhinovirus type 2 (HRV-2). The anti-RSV plaque assay was performed as described by Mollel et al.^49^ Briefly, serial 5-fold dilutions of the test sample in maintenance DMEM (Dulbecco’s modified Eagle’s medium supplemented with 2% heat-inactivated fetal calf serum, 1% pest stock, and 1% l-glutamine stock) were added to HEp-2 cells growing in a cluster 24-well plate and incubated at 37 °C for 15 min in a humidified atmosphere comprising 5% CO_2_ (the CO_2_ incubator). Subsequently, 50 μL of fresh DMEM comprising approximately 100 plaque-forming units of RSV A2 strain^50^ (ATCC, VR-1540) was added and placed in the CO_2_ incubator for a further 2.5 h. The final concentrations of the samples tested were 100, 20, 4, 0.8, 0.16, and 0.0 μM. Then, the virus–test sample mixture was removed, and the cells were overlaid with 1% methylcellulose solution in DMEM that comprised the same concentrations of the test sample. The assay plates were left for 3 days in the CO_2_ incubator, then stained with a 0.75% solution of crystal violet, with the developed RSV plaques counted under a microscope.

The anti-HRV-2 activity of the test samples was assayed as follows. The HeLa cells growing in cluster 96-well plates received 60 μL of maintenance EMEM (Eagle’s minimum essential medium supplemented with 2% fetal calf serum, 1% pest stock, 1% l-glutamine stock, 30 mM MgCl_2_, and 20 mM HEPES (pH 7.1)) and 20 μL of serial 5-fold dilutions of the test sample in EMEM. The assay plates were left in the CO_2_ incubator at 34 °C for 3 h, and then the cells with test samples received 20 μL of fresh EMEM comprising approximately 100 tissue culture infectious doses (TCID_50_) of HRV-2 strain HGP (ATCC, VR-482). The final concentrations of the samples tested were 100, 20, 4, 0.8, 0.16, and 0.0 μM. Following incubation in the CO_2_ incubator for 3 days, the cells were stained with crystal violet to visualize any protection of cells against the virus-induced cytopathic effect.

Cytotoxicity of the crude extracts and pure compounds for HEp-2 cells was tested as described by Mollel et al.^49^ Briefly, the cells, seeded in cluster 96-well plates the day prior to the experiment, were rinsed with DMEM, and 50 μL of fresh DMEM was added. Then, the cells received 50 μL of DMEM that comprised the test compounds at 5-fold increasing concentrations within a range of 0.16–100 μM or 0.16–100 μg/mL for the crude extracts. After incubation of cells with the test samples for 3 days in the CO_2_ incubator, 15 μL of the CellTiter 96 AQeous One Solution reagent (Promega, Madison, WI, USA) was added. The assay plates were shaken and left in the incubator for a further 1–2 h, and the absorbance of the samples was recorded at 490 nm. Cytotoxicity of the test samples for HeLa cells was performed in the same manner, except that EMEM was used instead of DMEM as the maintenance medium.

### Antibacterial Assays

The antibacterial activity of the isolated compounds was evaluated against *Bacillus subtilis* strain YB866 (Gram-positive) and *Escherichia coli* strain MG1655 (Gram-negative). The compounds were dissolved in DMSO according to their solubility and stored at −20 °C. Bacterial species were cultured as previously described by Mueller and Hinton^51^ and Doyle et al.^52^ For the determination of antibacterial activity, bacterial cells were grown overnight in cation-adjusted Mueller-Hinton II broth (MHB). The culture was diluted to OD = 0.05 in MHB. The concentration of the compounds tested was 3% v/v in 10 μL as the final volume (see Supporting Information). The assay was carried out in transparent 384-well plates at 37 °C without agitation for 18 h. After incubation, viability was measured by adding 1 μL of resazurin (AlamarBlue) per well and incubating at 37 °C for 1–2 h. Fluorescence was measured with a POLARstar Omega microplate reader (544–590 nm). Cells exposed to 3% v/v of DMSO were used as a positive control. The assay was performed in three independent replicates. Results are presented as the fluorescence mean normalized by the fluorescence of the positive control. The cutoff for *B. subtilis* was 0.1 and that for *E. coli* was 0.5; compounds with higher values were considered as nonactive against bacteria.

### Antiplasmodial Assays

For *P. falciparum* antiplasmodial assays, the chloroquine-resistant strain (Dd2) used in this study was cultured in RPMI medium supplemented with 10% A^+^ human plasma (Hematology Center of University of Campinas) at 5% hematocrit in type O^+^ human red blood cells (Hematology Center of University of Campinas) and maintained at 37 °C in a 1% O_2_, 5% CO_2_, and 94% N_2_ atmosphere, as described before.^53^ Synchronous cultures were obtained from treatment with a 5% d-sorbitol (Sigma-Aldrich) solution. Test compound inhibition assays were performed as described previously.^54^ Briefly, synchronized ring-stage (>90%) infected erythrocytes were dispensed in triplicate into 96-well plates (0.5% parasitemia and 2% hematocrit) in the presence of 50 μM of each crotofolane diterpenoid or the drug vehicle (DMSO), as a control. After 72 h of incubation, parasitemia was assessed by fluorometry using SybrGreen fluorescent dye. The growth inhibition values were calculated on GraphPad Prism software and expressed as percentage relative to the drug-free control. The experiments were carried out in three independent assays.

### Hemolysis Assay

The hemolysis assay was carried out according to Wang et al.^55^ with some modifications. Suspensions of erythrocytes (2% hematocrit) were incubated with the test compounds at 50 μM of crotofolane diterpenoids or the drug vehicle (DMSO), as a control, at 37 °C, 5% CO_2_, for 4 h. The reaction mixtures were centrifuged at 1000*g* for 5 min, and the absorbances of the supernatants were measured at 540 nm using a Biotek Synergy-HT spectrophotometer. The hemolytic rate was calculated in relation to the hemolysis of erythrocytes in 10% Triton X100 that was taken as 100%. The experiments were determined as in three independent assays.

## Data Availability

The original FIDs, NMReDATA files,^56,57^ and CSEARCH^41,58,59^ reports for compounds **1**–**6** are freely available on Zenodo with DOI: 10.5281/zenodo.6866841.

## Supporting Information Available

NMR and MS data for the isolated compounds and antiviral, antibacterial, cytotoxicity, and antiplasmodial data (PDF)
X-ray crystallographic data for compound **1** (CIF)

## Supplementary Material

np2c01007_si_001.pdf

np2c01007_si_002.cif
