# Supplementary material for: Crotofolane Diterpenoids and Other Constituents Isolated from Croton kilwae
Source: J Nat Prod. 2023 Feb 7;86(2):380–9. doi: 10.1021/acs.jnatprod.2c01007 (PMC9972476; doi:10.1021/acs.jnatprod.2c01007)
Supplement: Supplementary file 1 — np2c01007_si_001.pdf [file np2c01007_si_001.pdf]

SUPPORTING INFORMATION

Crotofolane Diterpenoids and Other Constituents from *Croton kilwae*

Emanuel T. Mahambo, Colores Uwamariya, Masum Miah, Gabriela Paula Di Santo Meztler, Edward Trybala, Joanna Said, Lianne H.E. Wieske, Jas S. Ward, Kari Rissanen, Joan J. E. Munissi, Per Sunnerhagen, Tomas Bergström, Stephen S. Nyandoro, and Mate Erdelyi

Table of Contents S1

1. The structures of known compounds **7-19** S2
2. Spectroscopic data for compound **1** S3
3. Spectroscopic data for compound **2** S7
4. Spectroscopic data for compound **3** S11
5. Spectroscopic data for compound **4** S15
6. Spectroscopic data for compound **5** S19
7. Spectroscopic data for compound **6** S23
8. Spectroscopic data of compound **7** S27
9. Spectroscopic data for compound **8** S31
10. Spectroscopic data for compound **9** S35
11. Spectroscopic data for compound **10** S39
12. Spectroscopic data for compound **11** S43
13. Spectroscopic data for compound **12** S47
14. Spectroscopic data for compound **13** S51
15. Spectroscopic data for compound **14** S55
16. Spectroscopic data for compound **15** S59
17. Spectroscopic data for compound **16** S63
18. Spectroscopic data for compound **17** S67
19. Spectroscopic data for compound **18** S68
20. Spectroscopic data for compound **19** S72
21. Other spectroscopic and physical data of known compounds (**7-19**) S76
22. X-ray crystallography of compound **1** S77
23. Antiviral, antibacterial and cytotoxicity data S78
24. Antiplasmodial data S80

The original NMR spectra and the corresponding NMReDATA for compounds **1-6** are freely available on Zenodo as DOI:10.5281/10.5281/zenodo.6866841.

1. The structures of known compounds 7-19

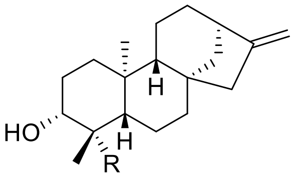

7 R = CH_2OH

8 R = COOH

12 R = Me

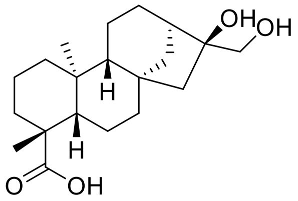

9

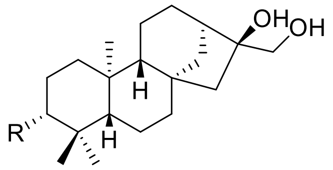

10 R = OH

11 R = H

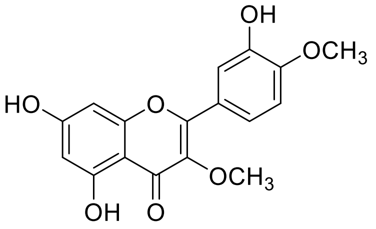

13

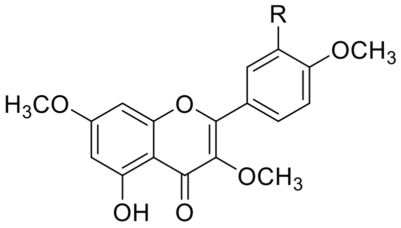

14 R = H

15 R = OCH_3

16 R = OH

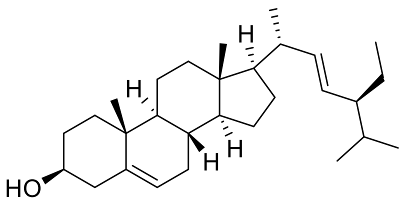

17

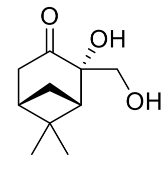

18

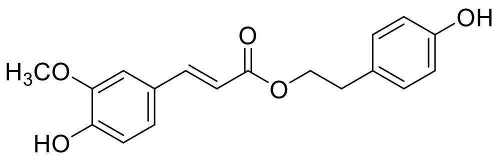

19

Figure S1. Structures of known compounds **7-19**

2. Spectroscopic data for compound 1

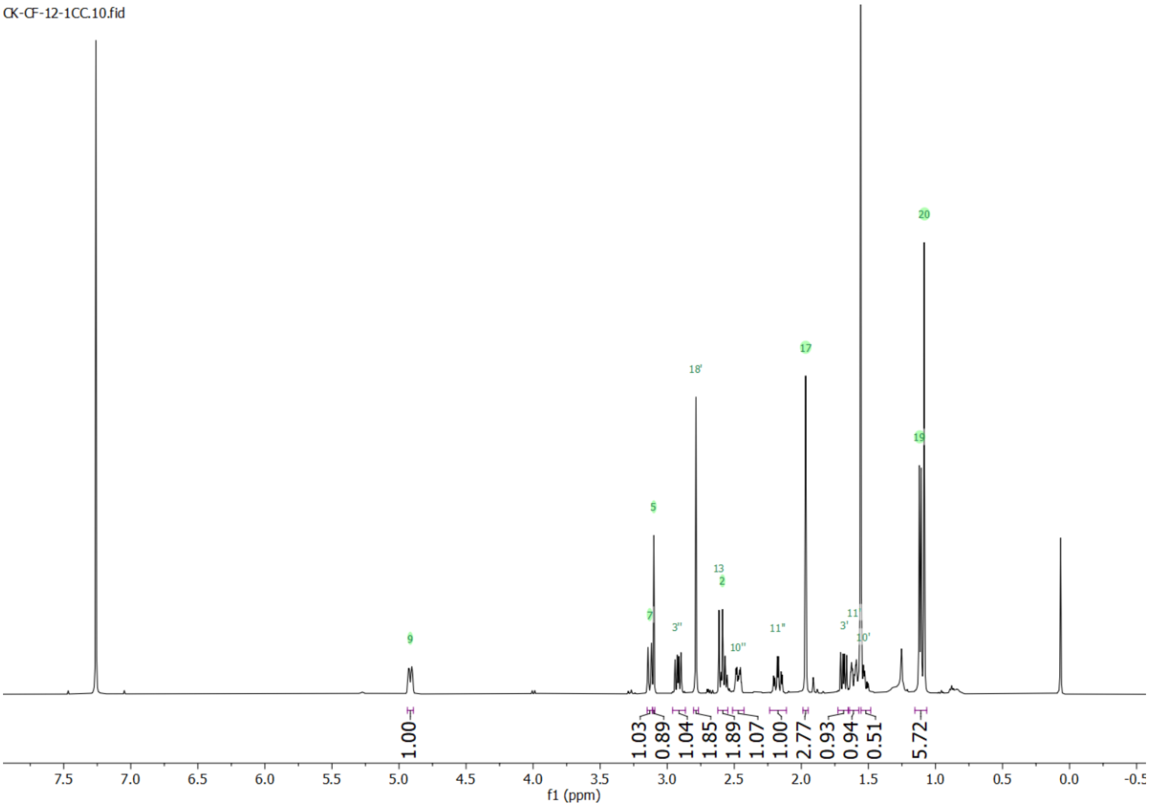

Figure S2. The ^{1}H NMR spectrum of compound **1** (500 MHz, $CDCl_3$)

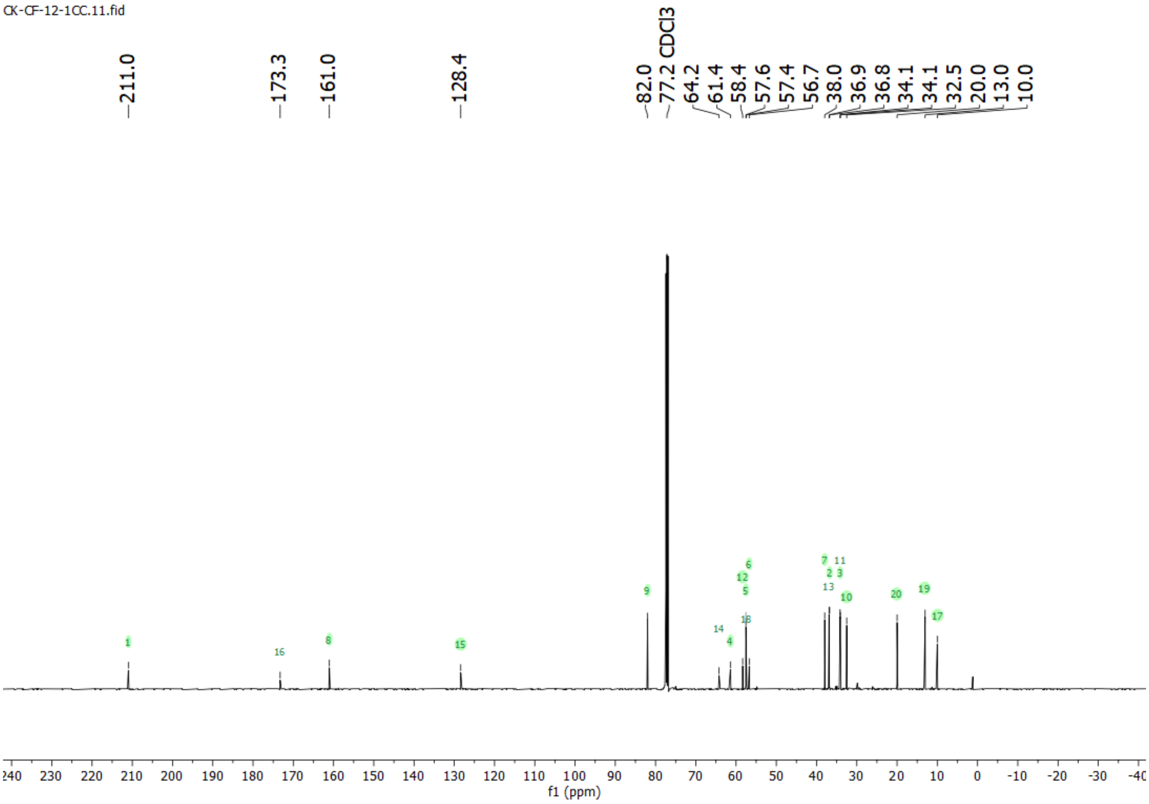

Figure S3. The ^{13}C NMR spectrum of compound **1** (125 MHz, $CDCl_3$)

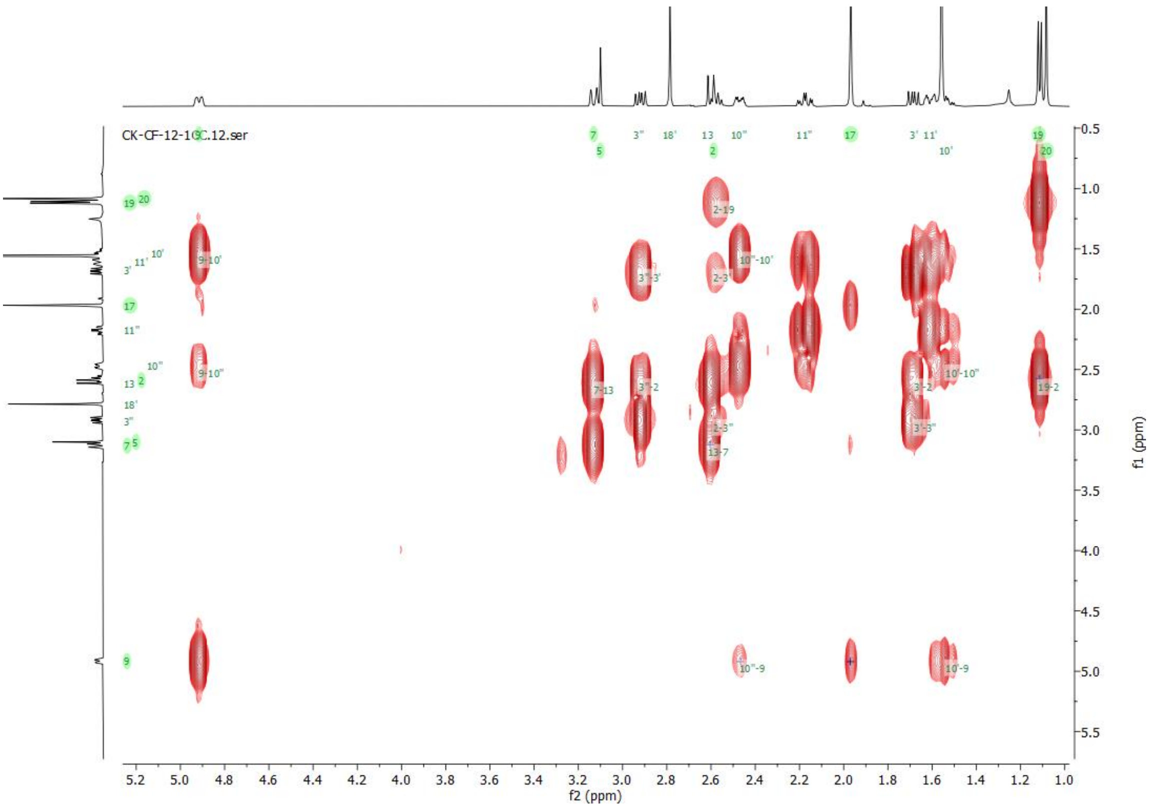

Figure S4. The COSY spectrum of compound **1** (500 MHz, $CDCl_3$)

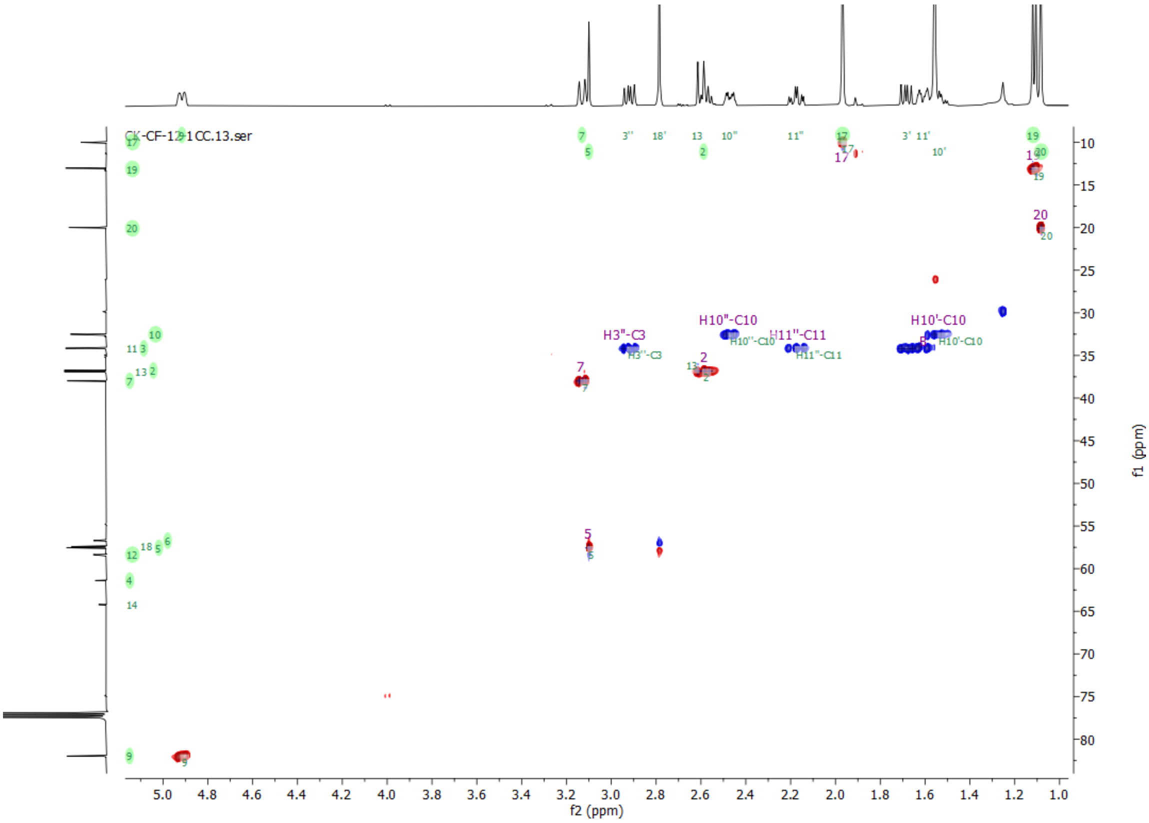

Figure S5. The HSQC spectrum of compound **1** (500/125 MHz, $CDCl_3$)

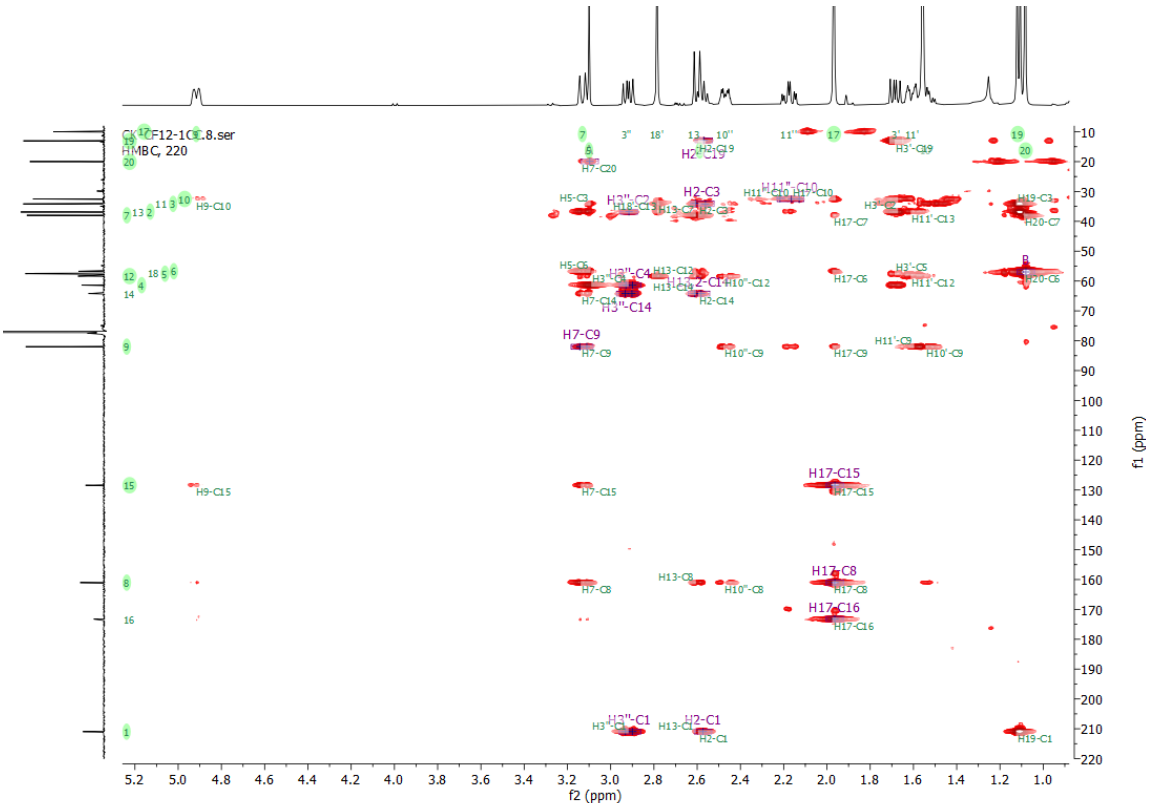

Figure S6. The HMBC spectrum of compound **1** (500/ 125 MHz, $CDCl_3$)

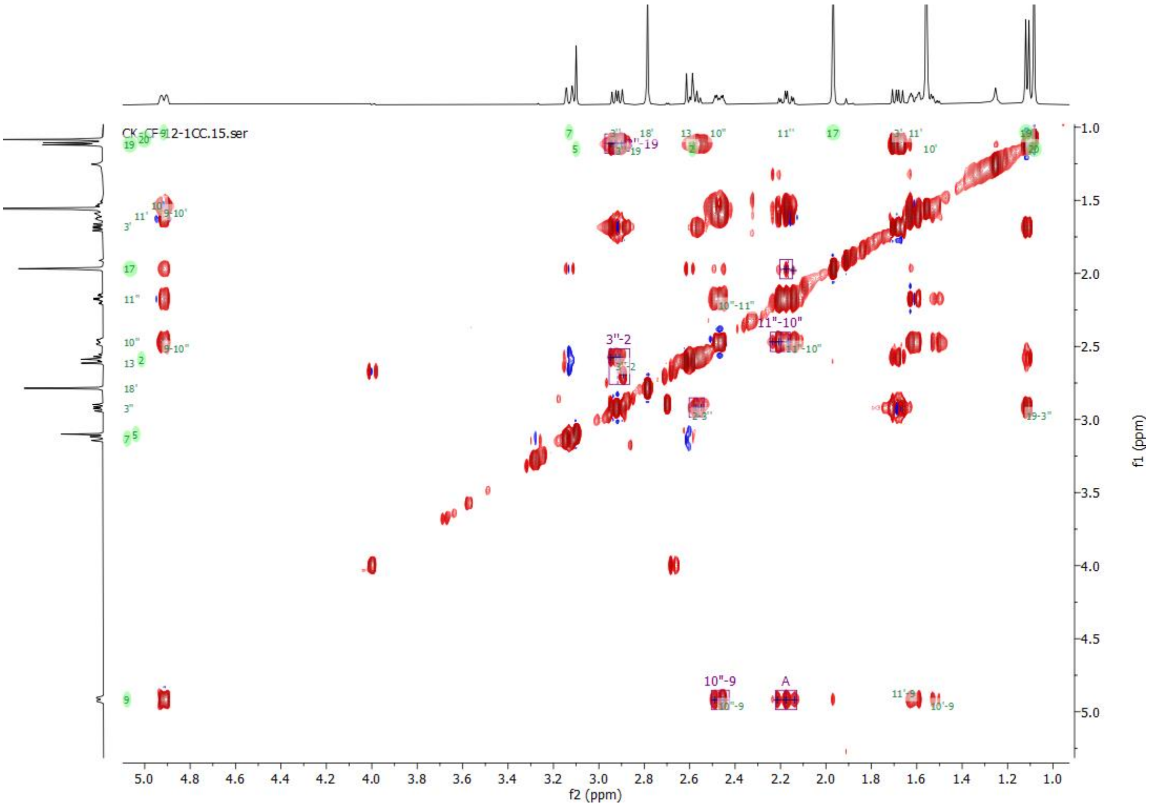

Figure S7. The TOCSY spectrum of compound **1** (500 MHz, $CDCl_3$)

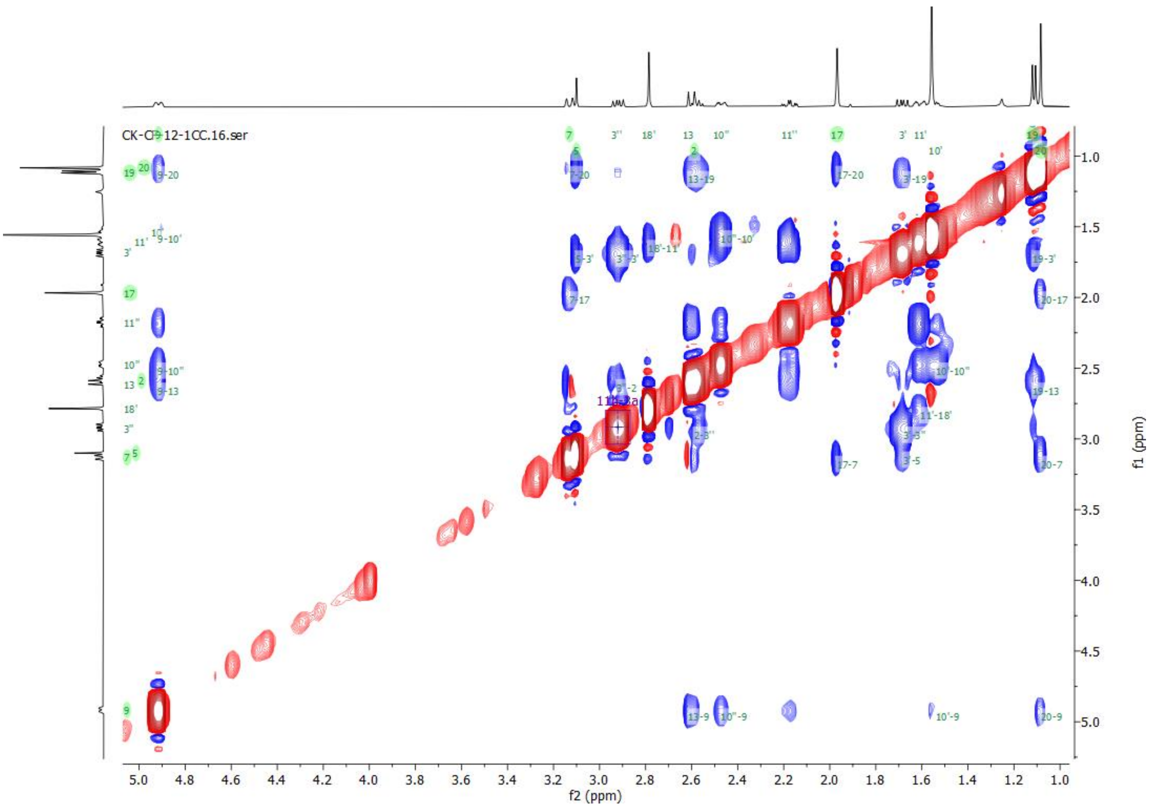

Figure S8. The NOESY spectrum of compound **1**. (500 MHz, $CDCl_3$)

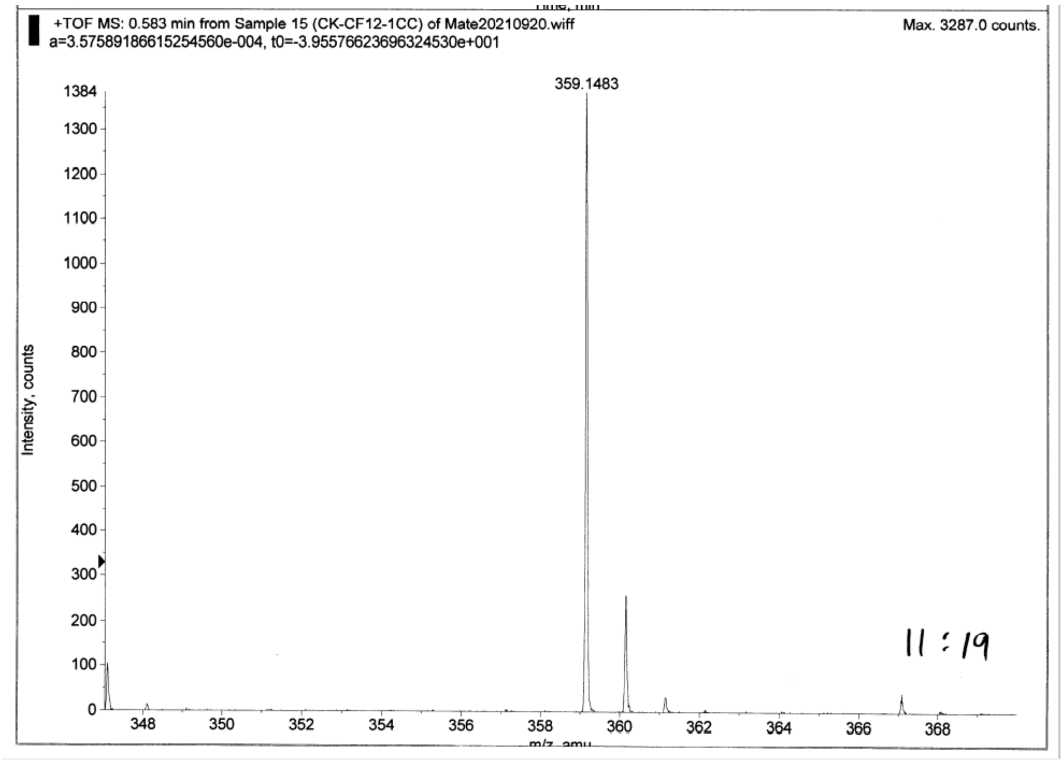

Figure S9.The HRESIMS of compound **1**.

3. Spectroscopic data for compound 2

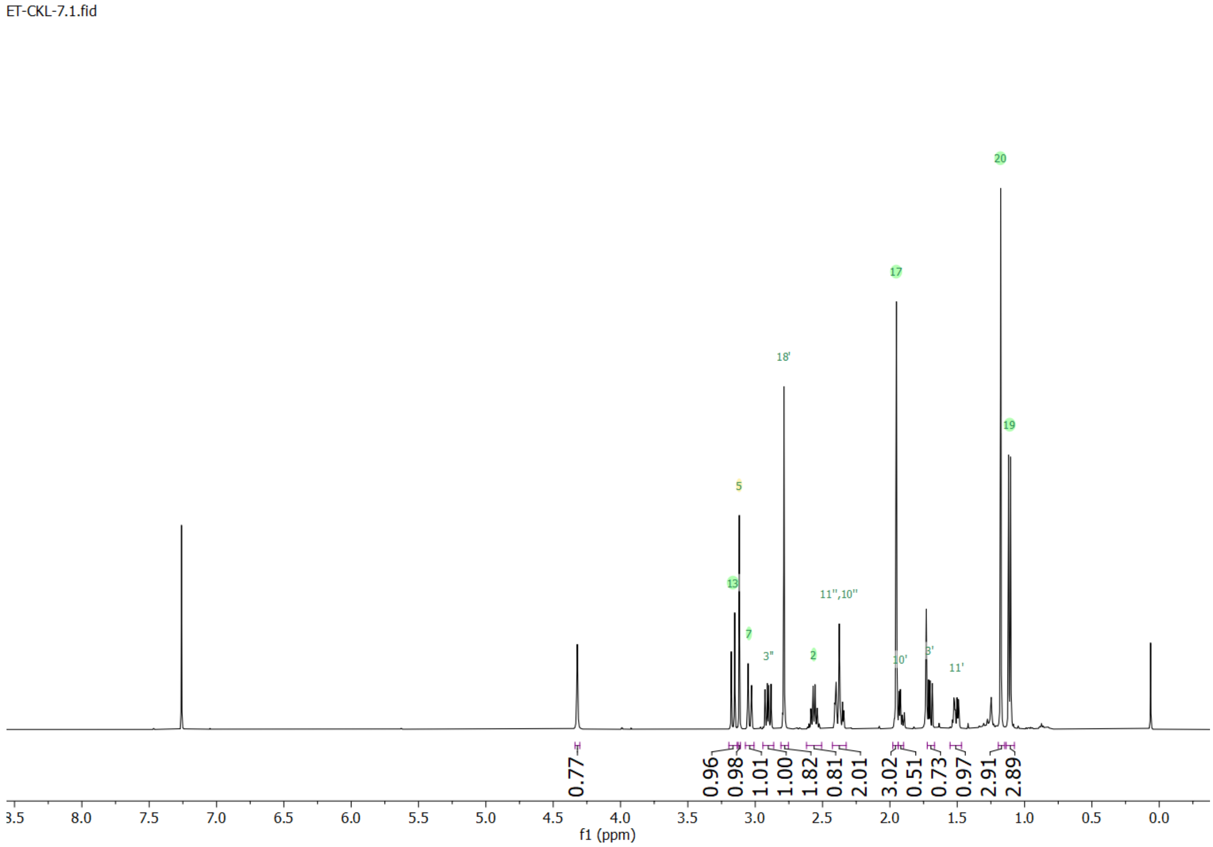

Figure S10. The ^{1}H NMR spectrum of compound **2** (500 MHz, $CDCl_3$)

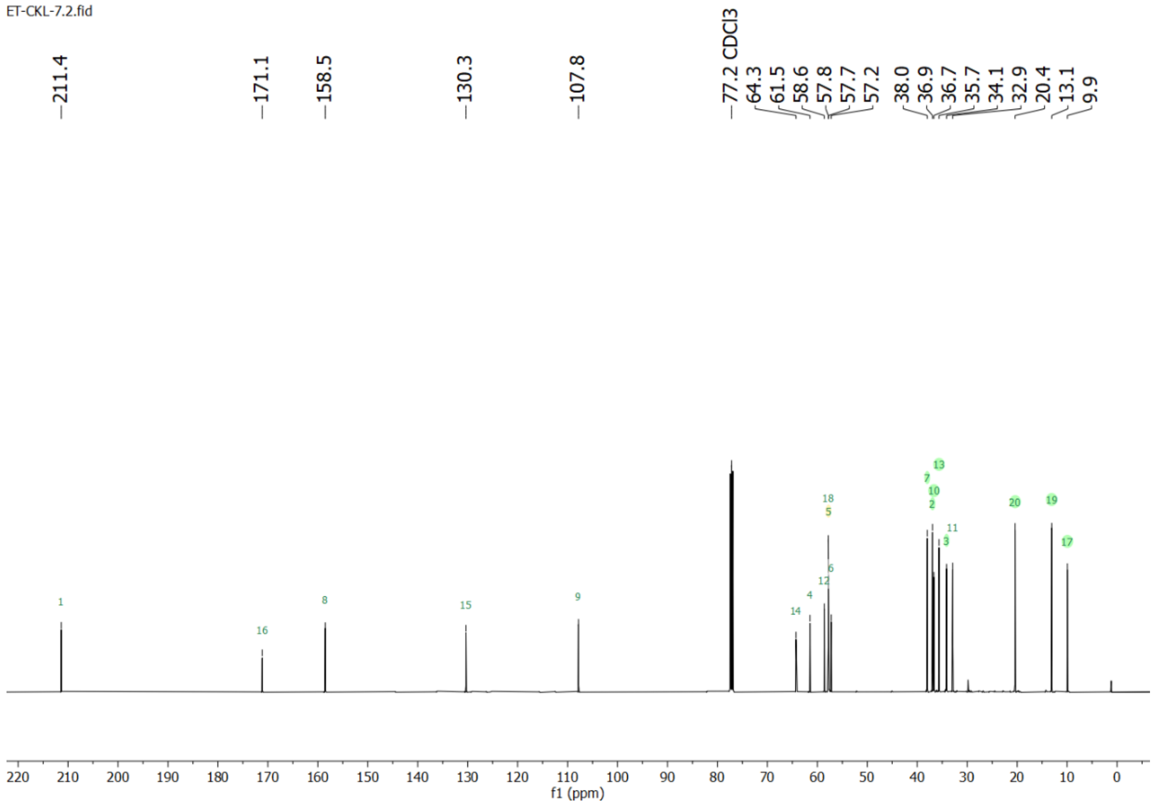

Figure S11. The ^{13}C NMR spectrum of compound **2** (125 MHz, $CDCl_3$)

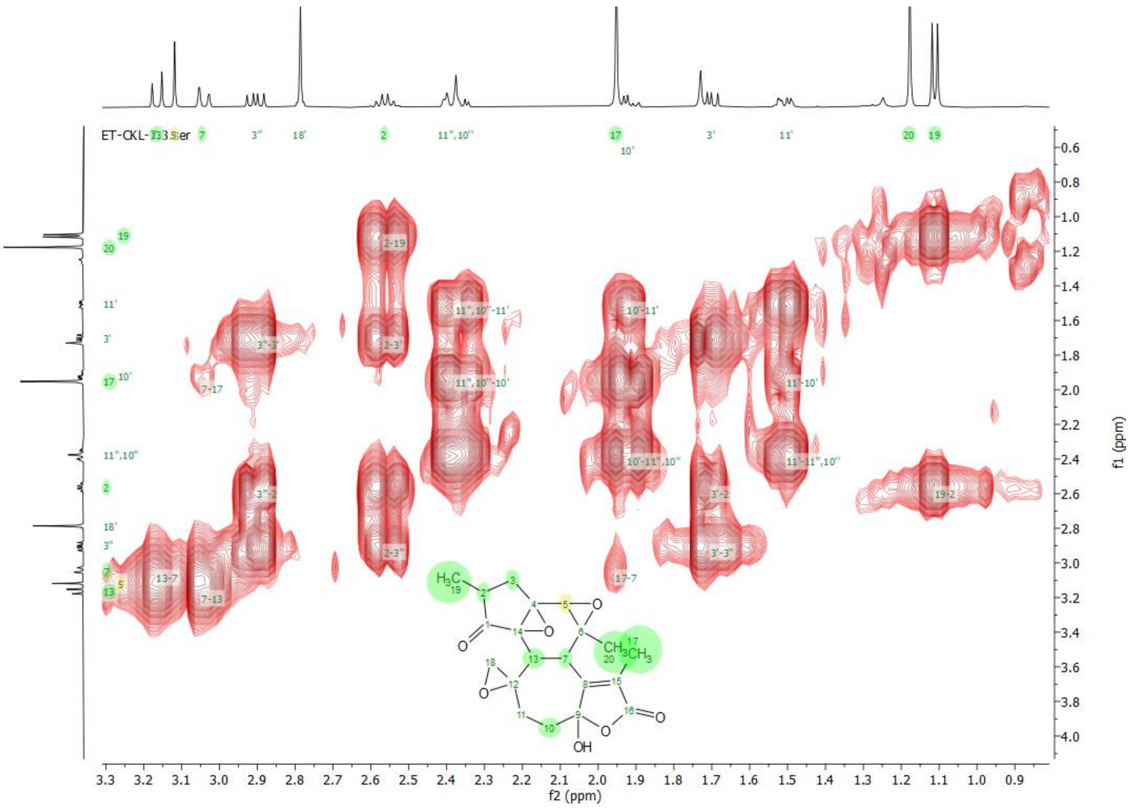

Figure S12. The COSY spectrum of compound **2** (500 MHz, $CDCl_3$)

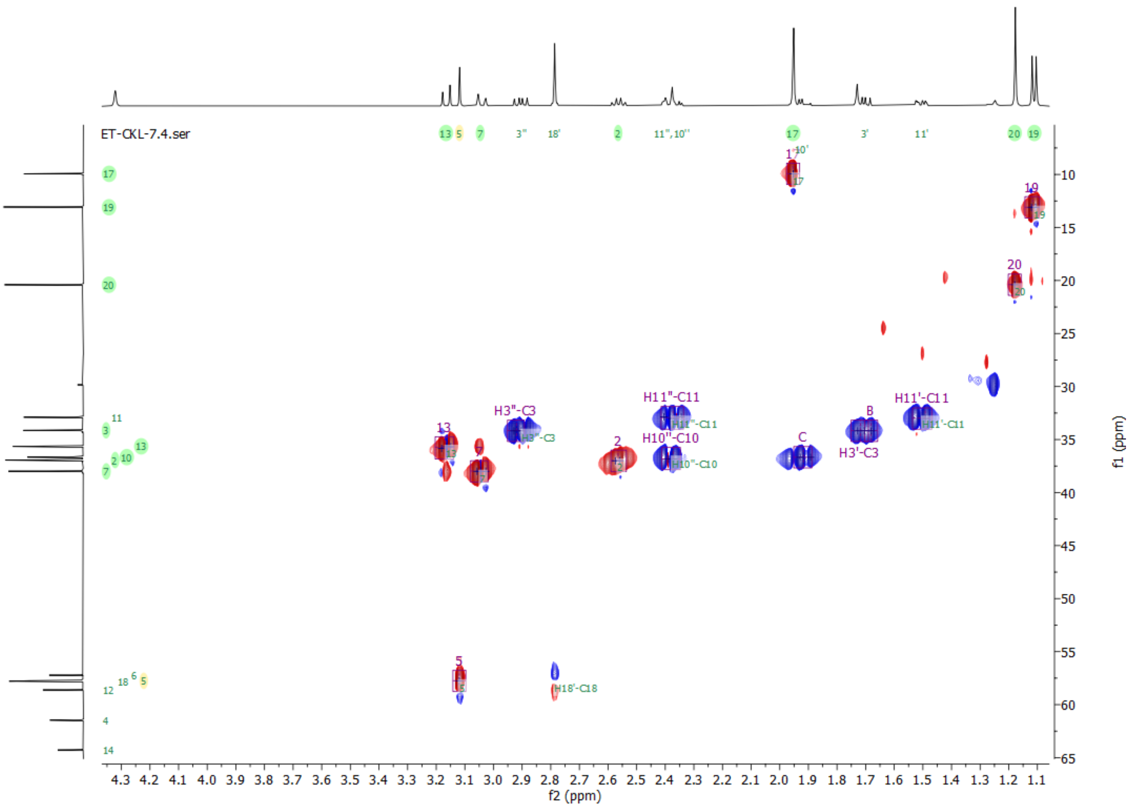

Figure S13. The HSQC spectrum of compound **2** (500/ 125 MHz, $CDCl_3$)

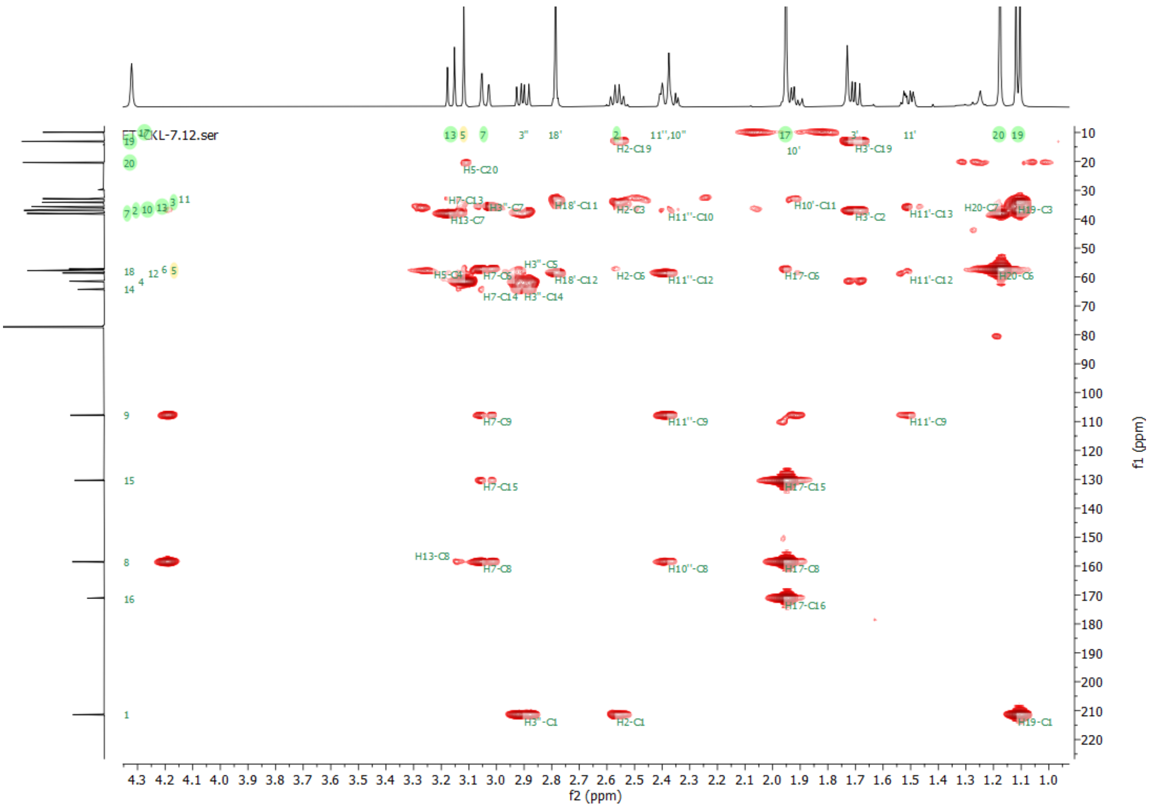

Figure S14. The HMBC spectrum of compound **2** (500/125 MHz, $CDCl_3$)

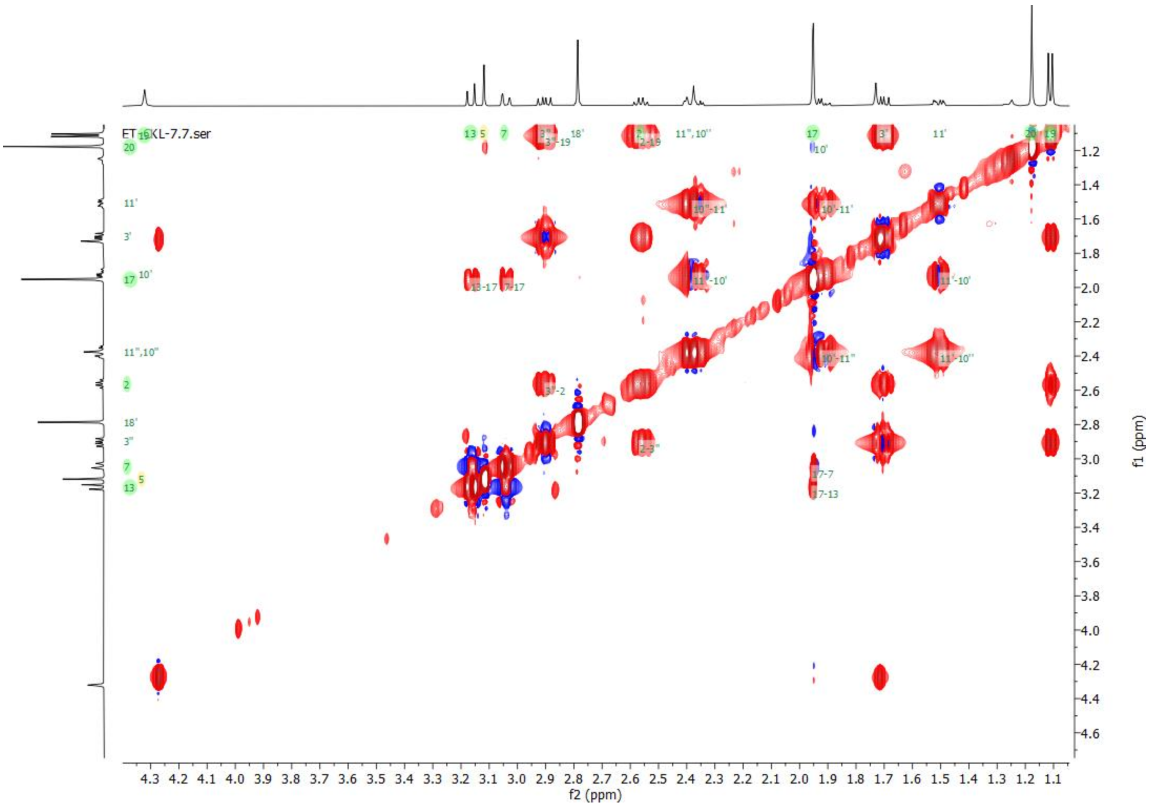

Figure S15. The TOCSY spectrum of compound **2** (500 MHz, $CDCl_3$)

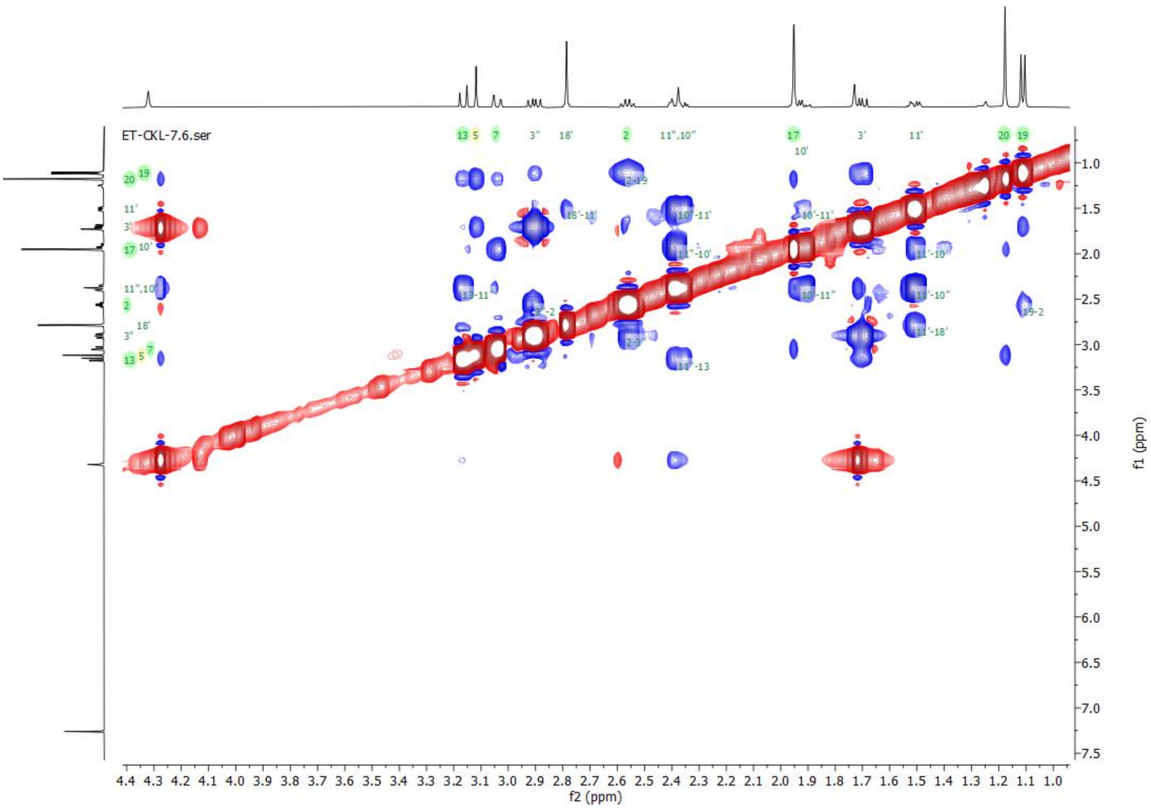

Figure S16. The NOESY spectrum of compound **2** (500 MHz, $CDCl_3$)

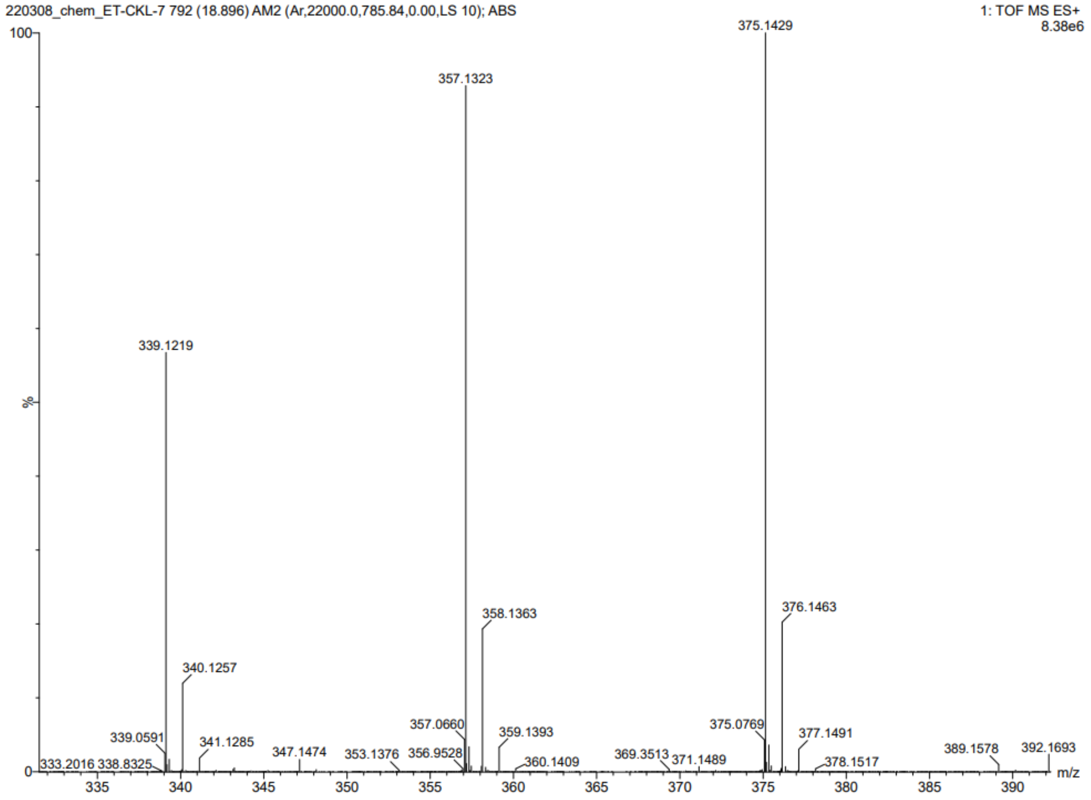

Figure S17. The HRESIMS of compound **2**

4. Spectroscopic data for compound 3

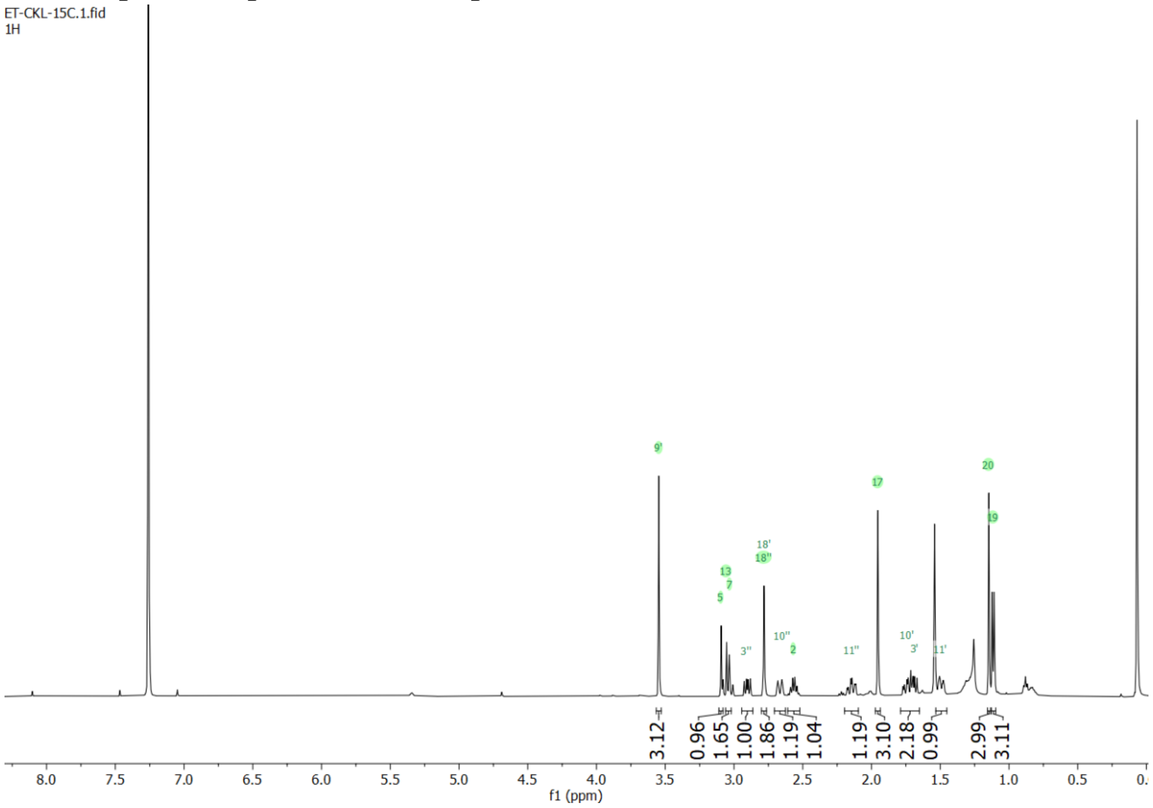

Figure S18. The ^{1}H NMR spectrum of compound **3** (500 MHz, $CDCl_3$)

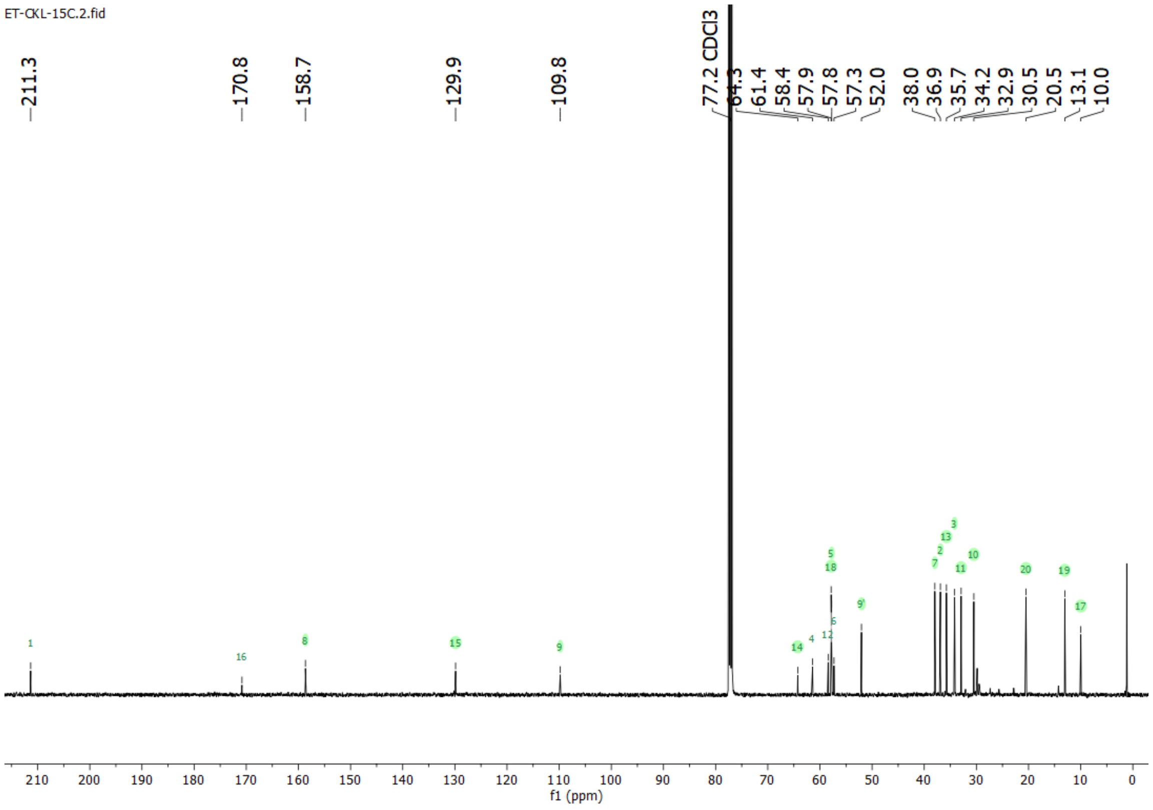

Figure S19. The ^{13}C NMR spectrum of compound **3** (125 MHz, $CDCl_3$)

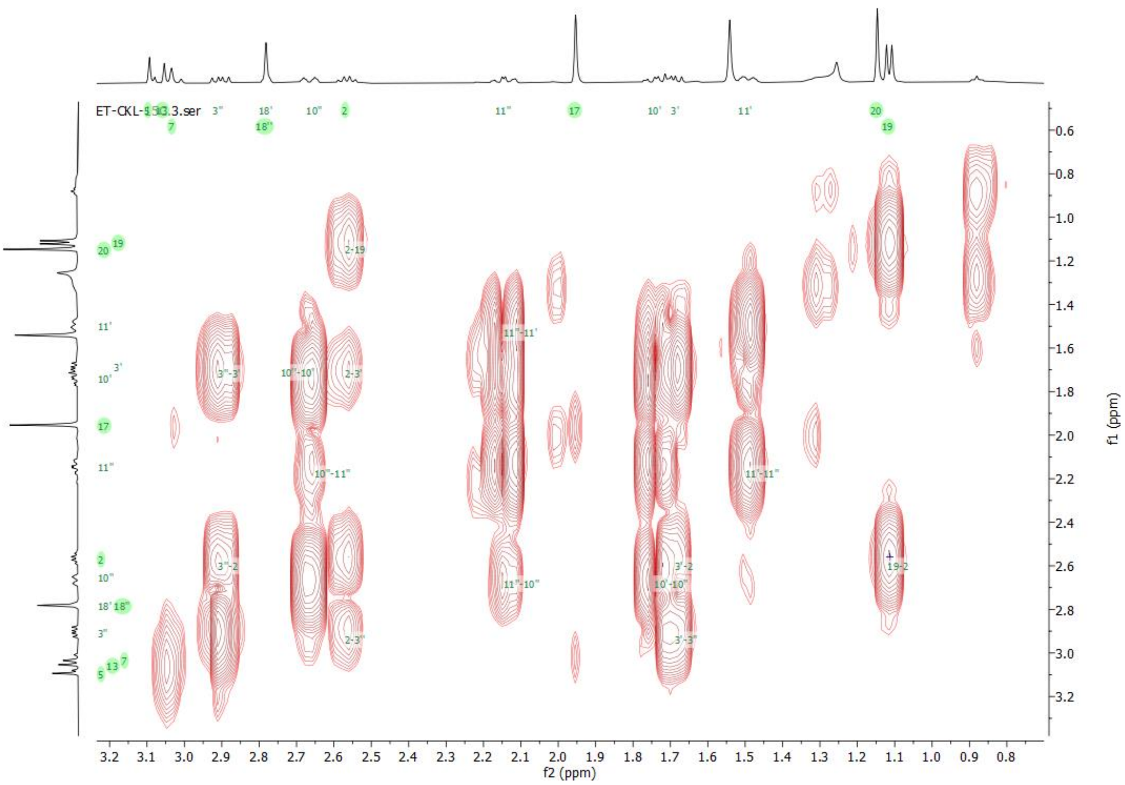

Figure S20. The COSY spectrum of compound **3** (500MHz, $CDCl_3$)

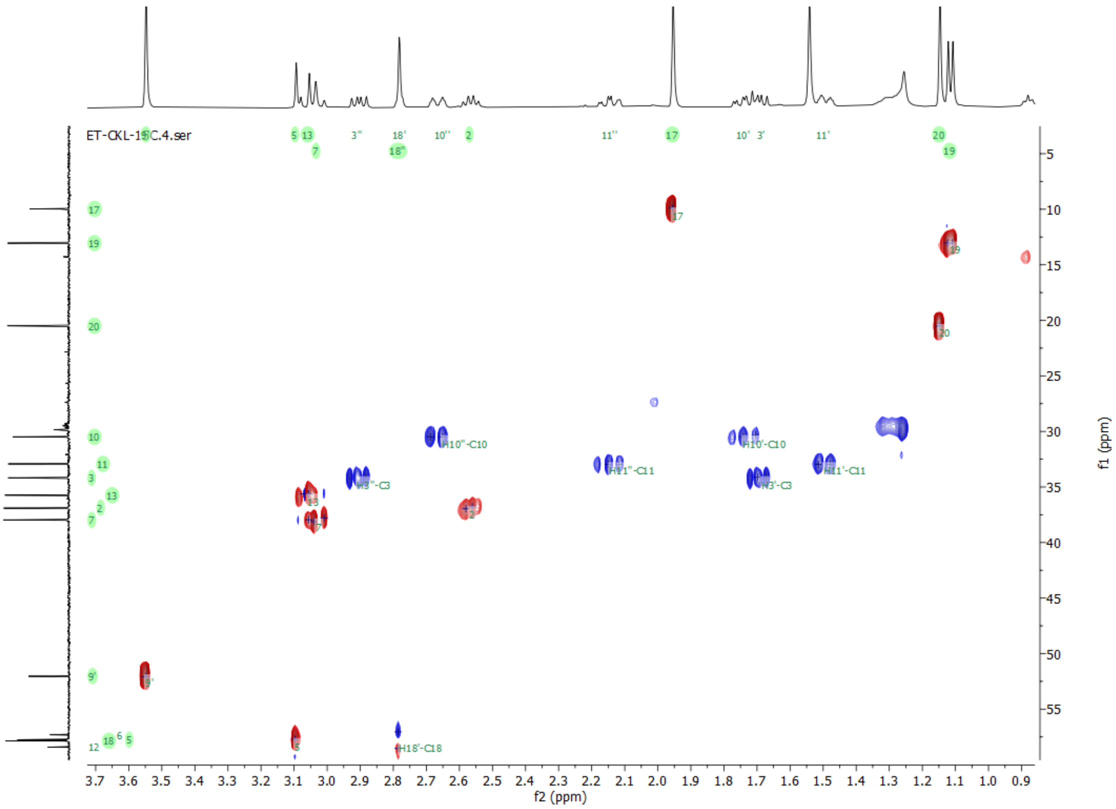

Figure S21. The HSQC spectrum of compound **3** (500/125 MHz, $CDCl_3$)

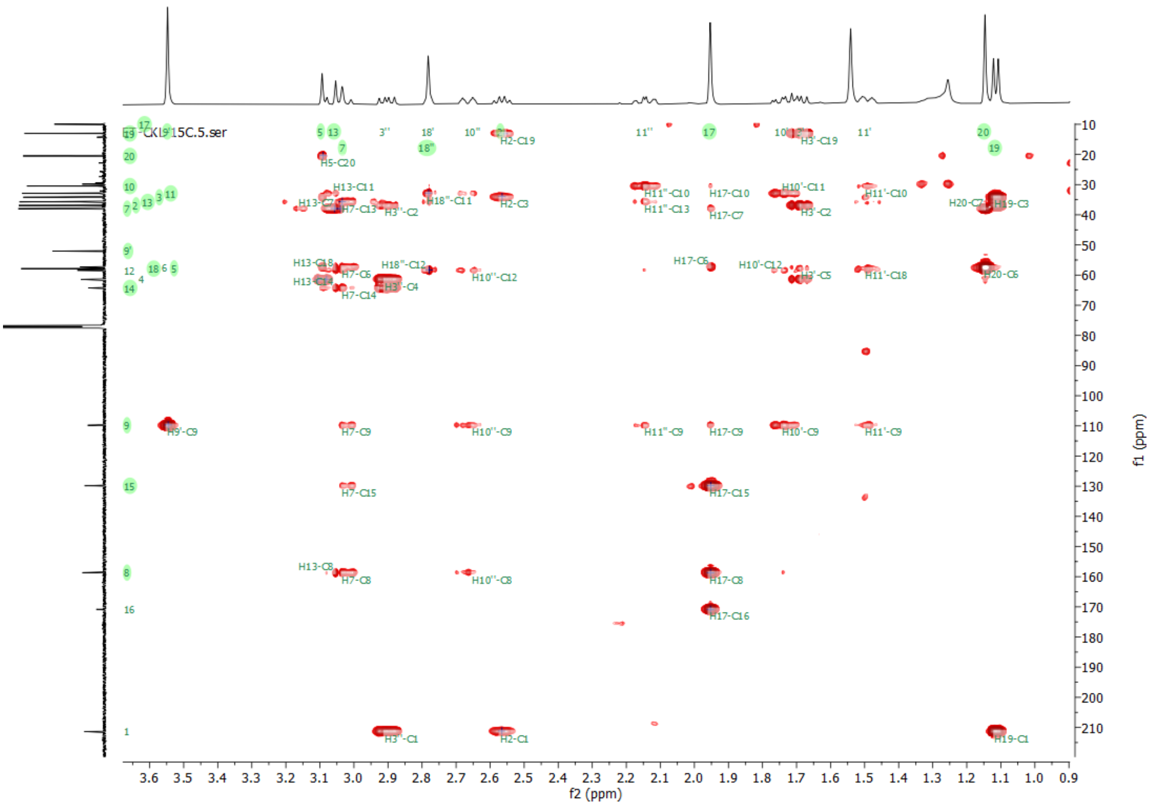

Figure 22. The HMBC spectrum of compound **3** (500/125 MHz, $CDCl_3$)

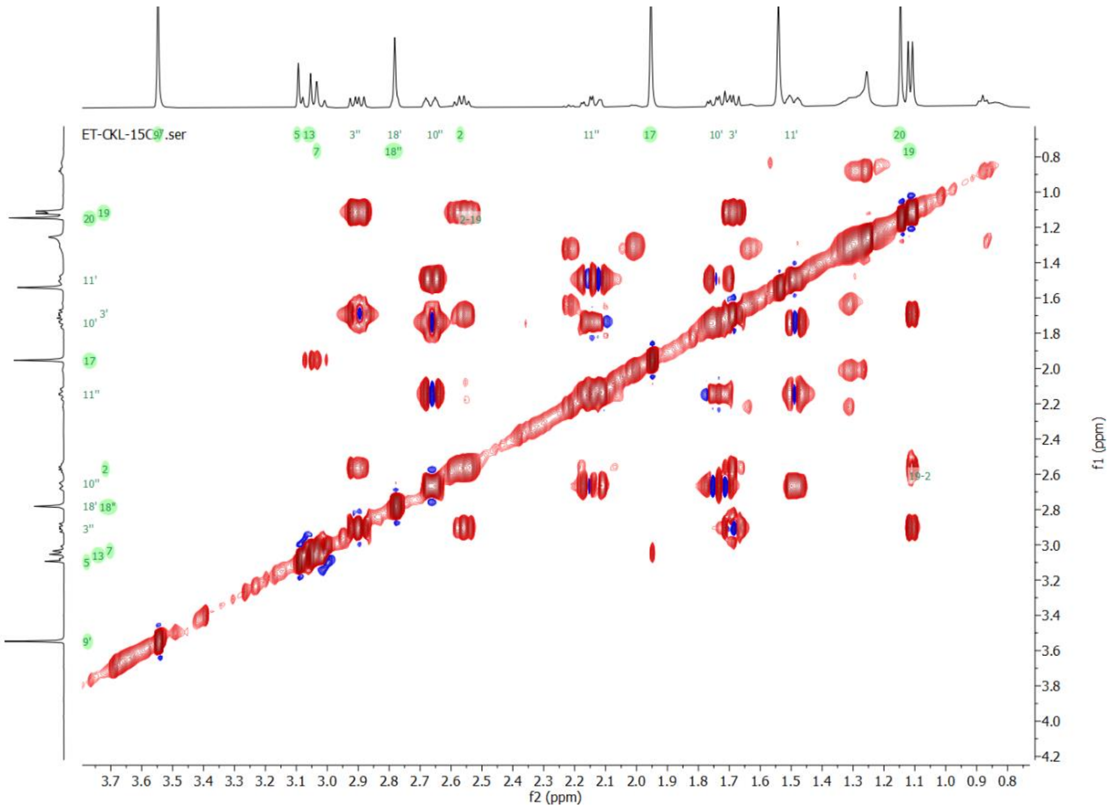

Figure S23. The TOCSY spectrum of compound **3** (500 MHz, $CDCl_3$)

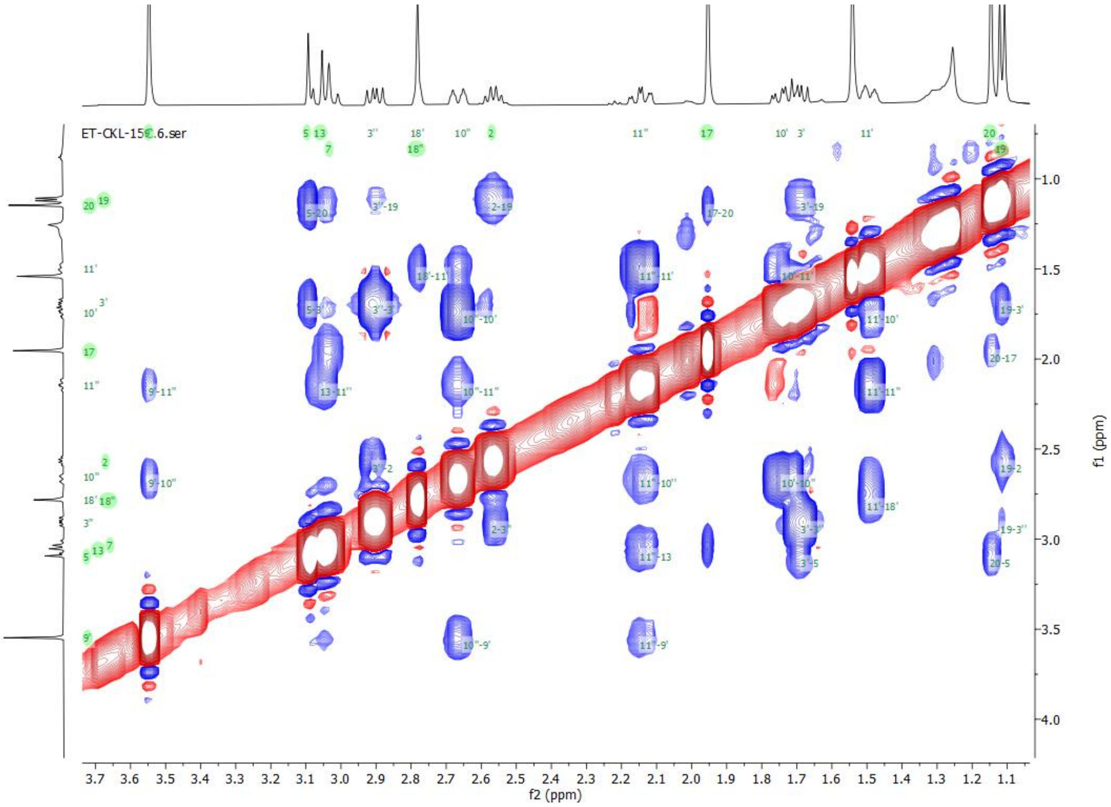

Figure S24. The NOESY spectrum of compound **3** (500 MHz, $CDCl_3$)

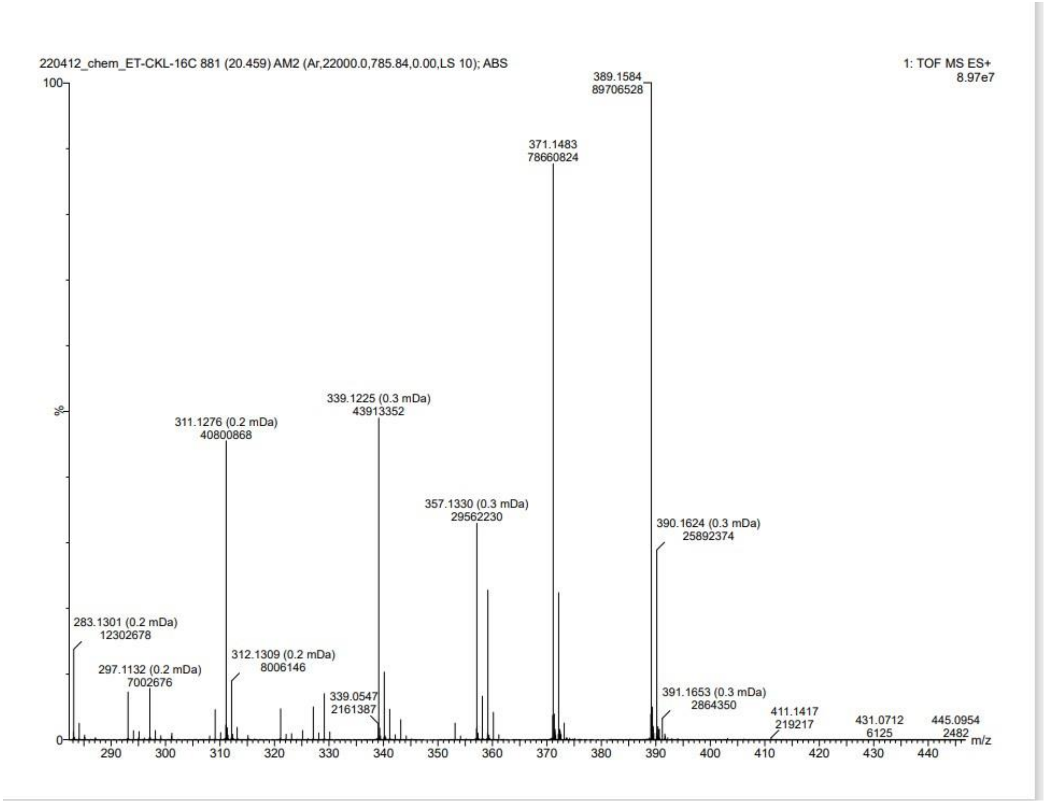

Figure S25. The HRESIMS of compound **3** (500 MHz, $CDCl_3$)

5. **Spectroscopic data for compound 4**

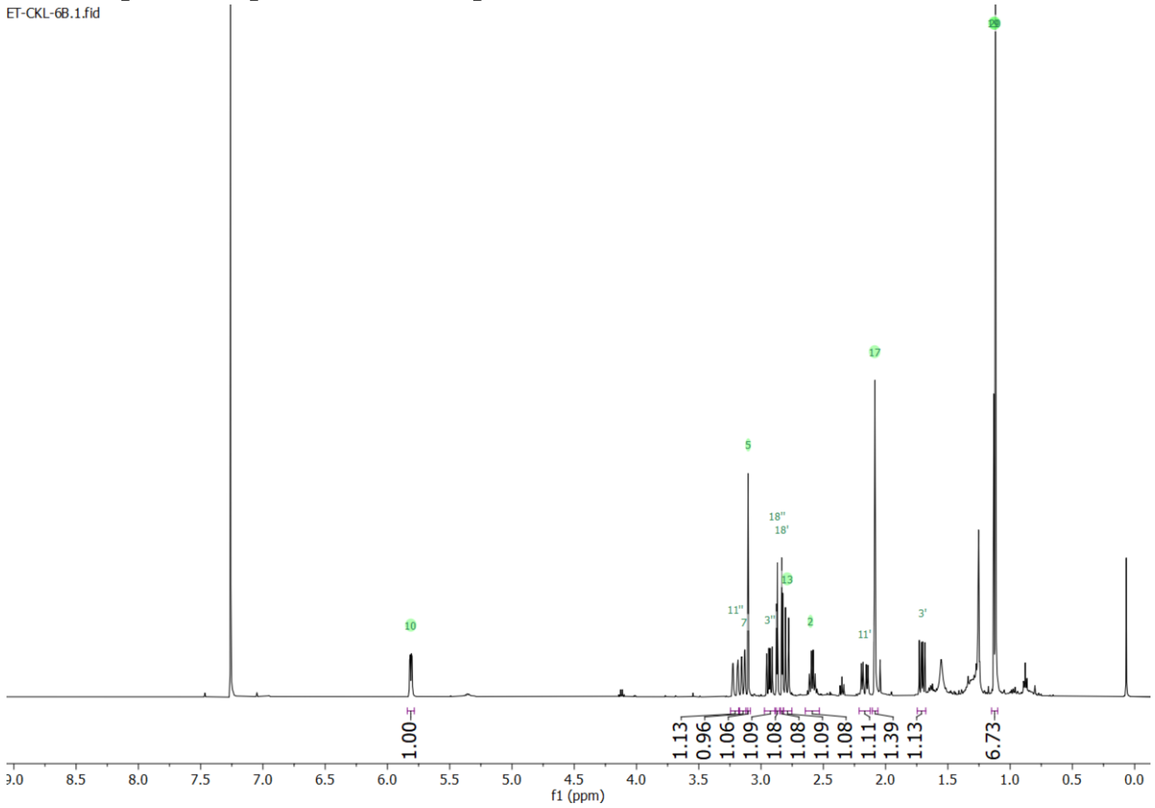

Figure S26. The ^{1}H NMR spectrum of compound **4** (500 MHz, $CDCl_3$)

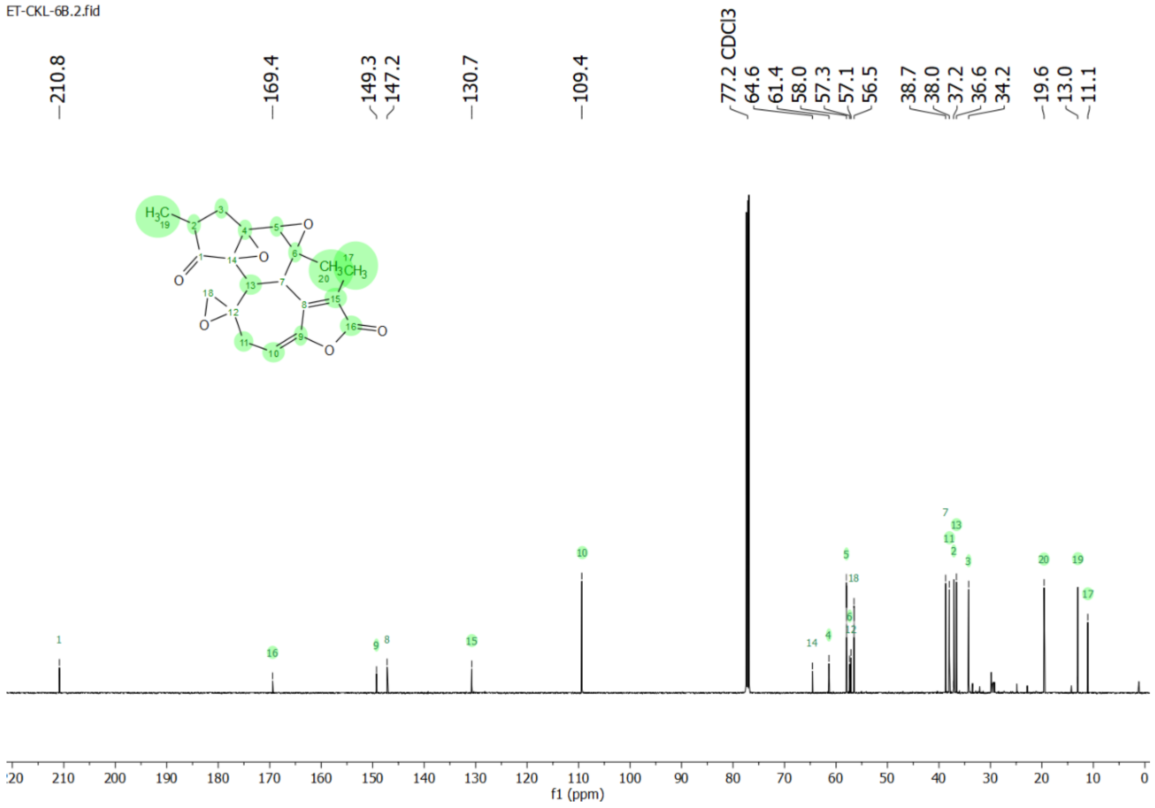

Figure S27. The ^{13}C NMR spectrum of compound **4** (125 MHz, $CDCl_3$)

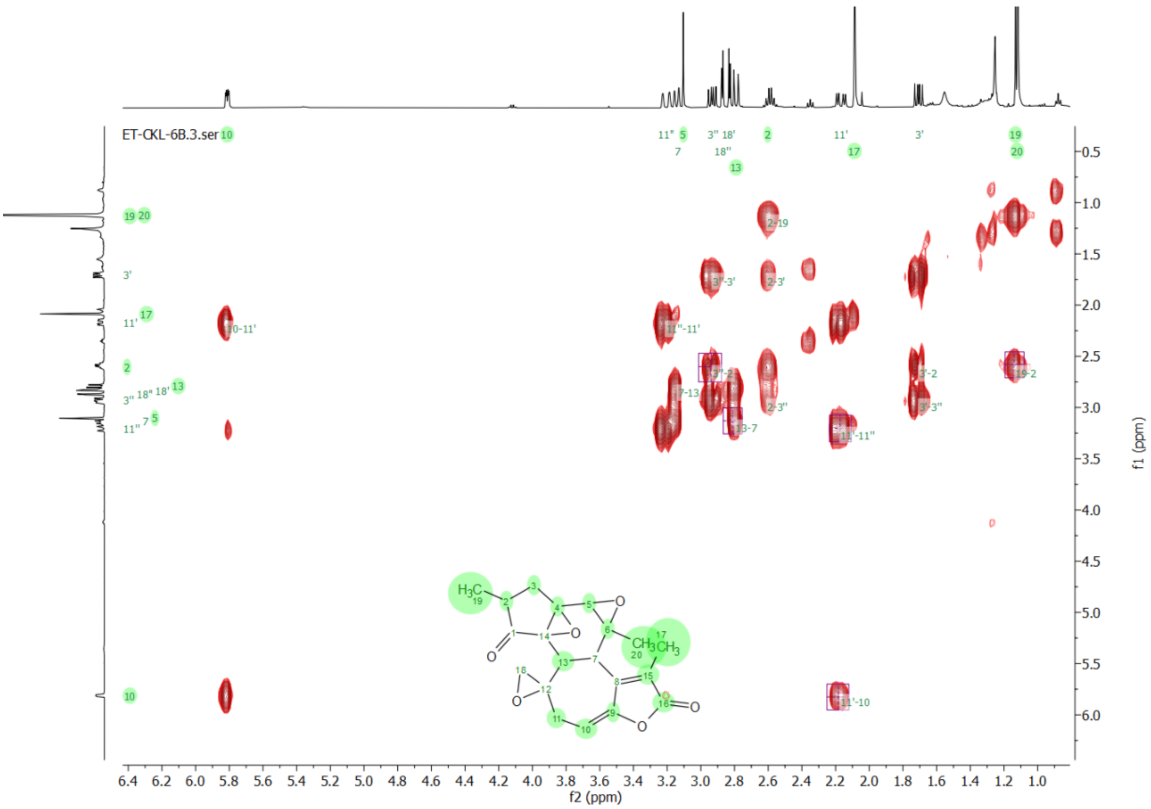

Figure S28. The COSY spectrum of compound **4** (500 MHz, $CDCl_3$)

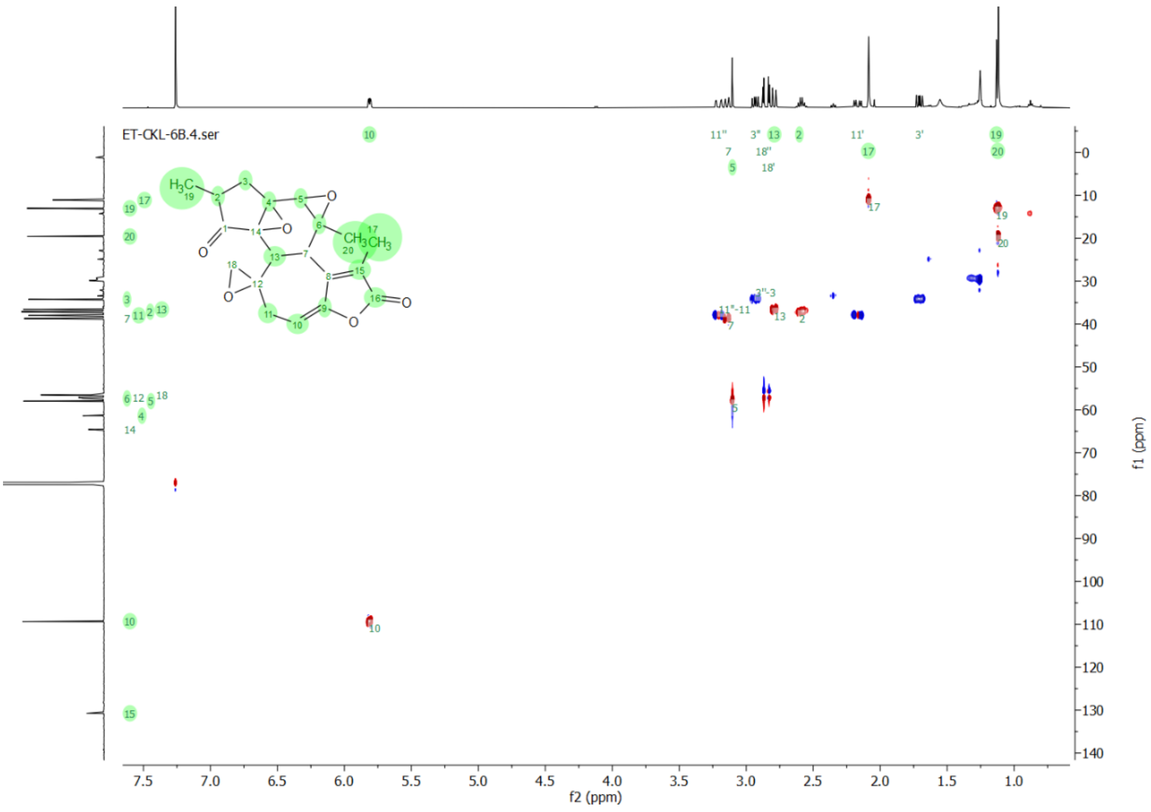

Figure S29. The HSQC spectrum of compound **4** (500/125 MHz, $CDCl_3$)

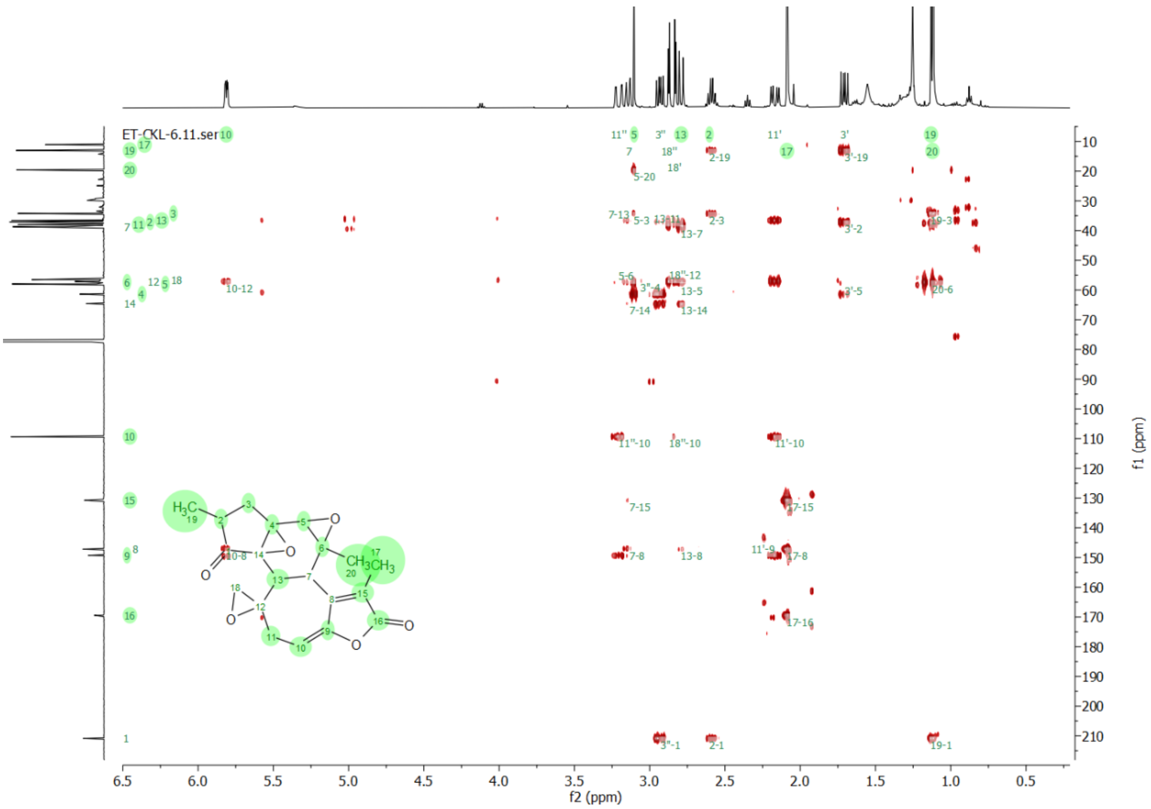

Figure S30. The HMBC spectrum of compound **4** (500/125 MHz, $CDCl_3$)

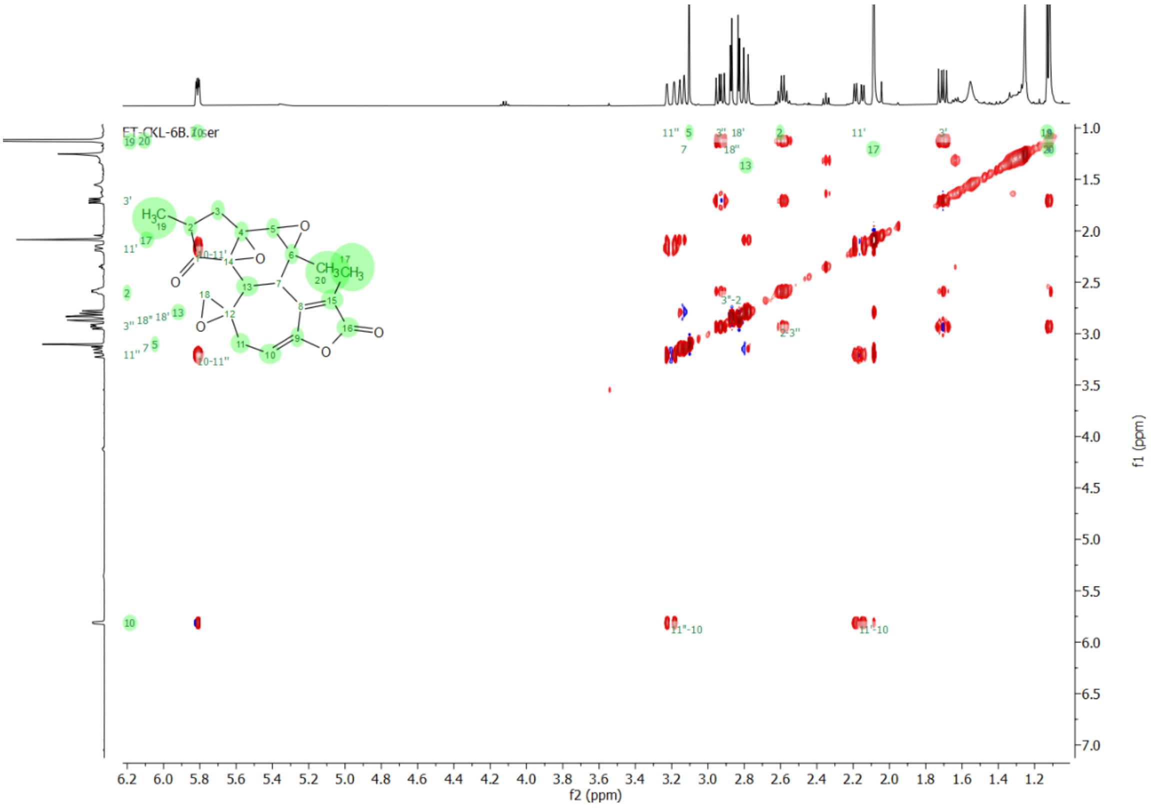

Figure S31. The TOCSY spectrum of compound **4** (500 MHz, $CDCl_3$)

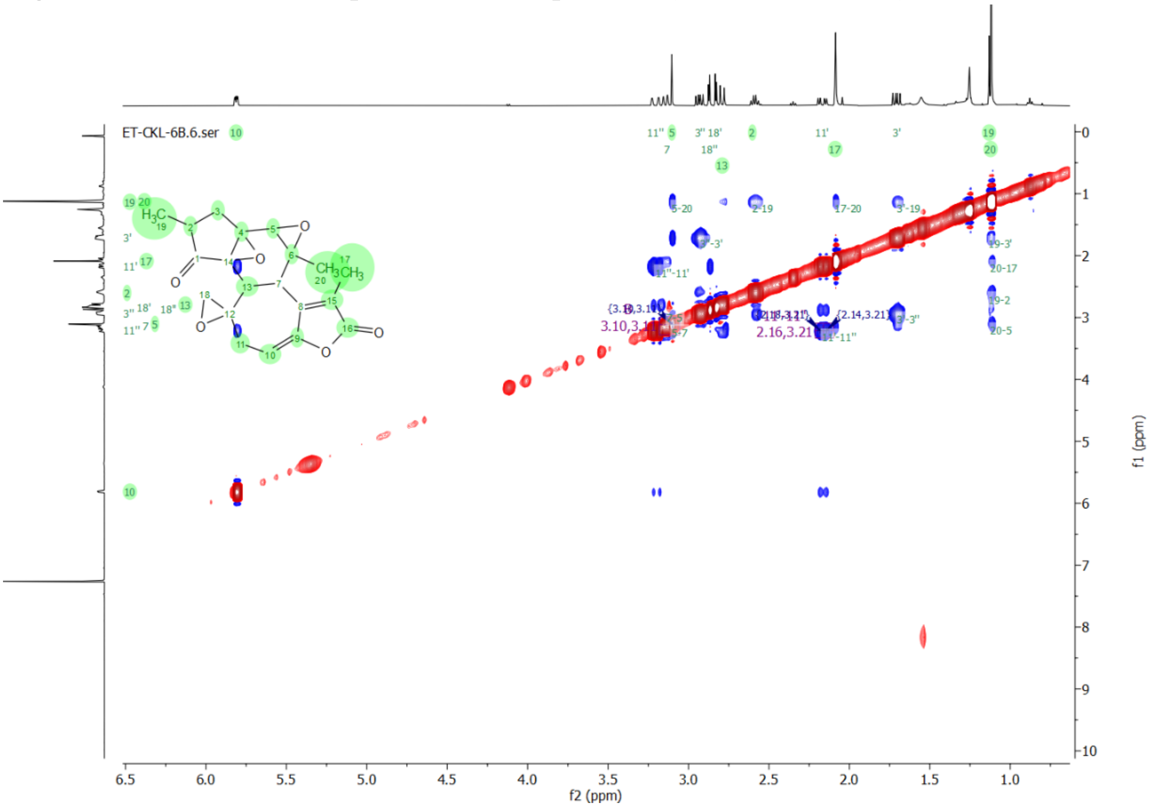

Figure S32. The NOESY spectrum of compound **4** (500MHz, $CDCl_3$)

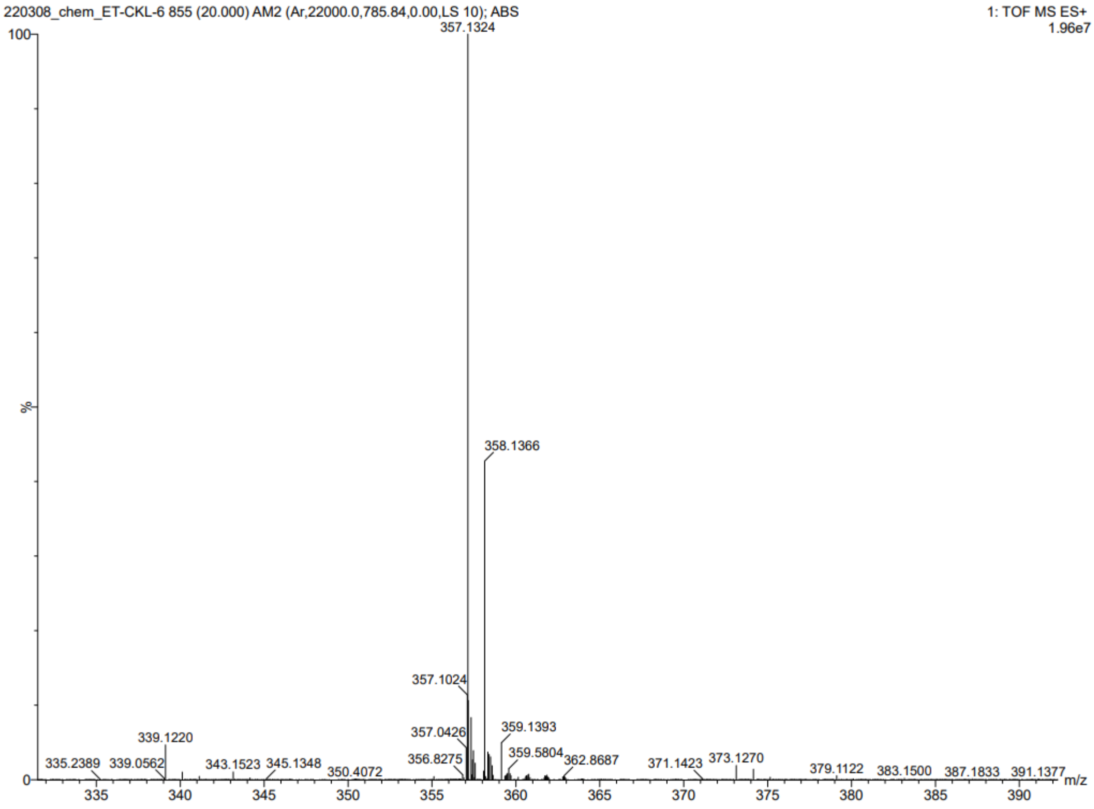

Figure S33. The HRESIMS of compound **4**

6. Spectroscopic data for compound 5

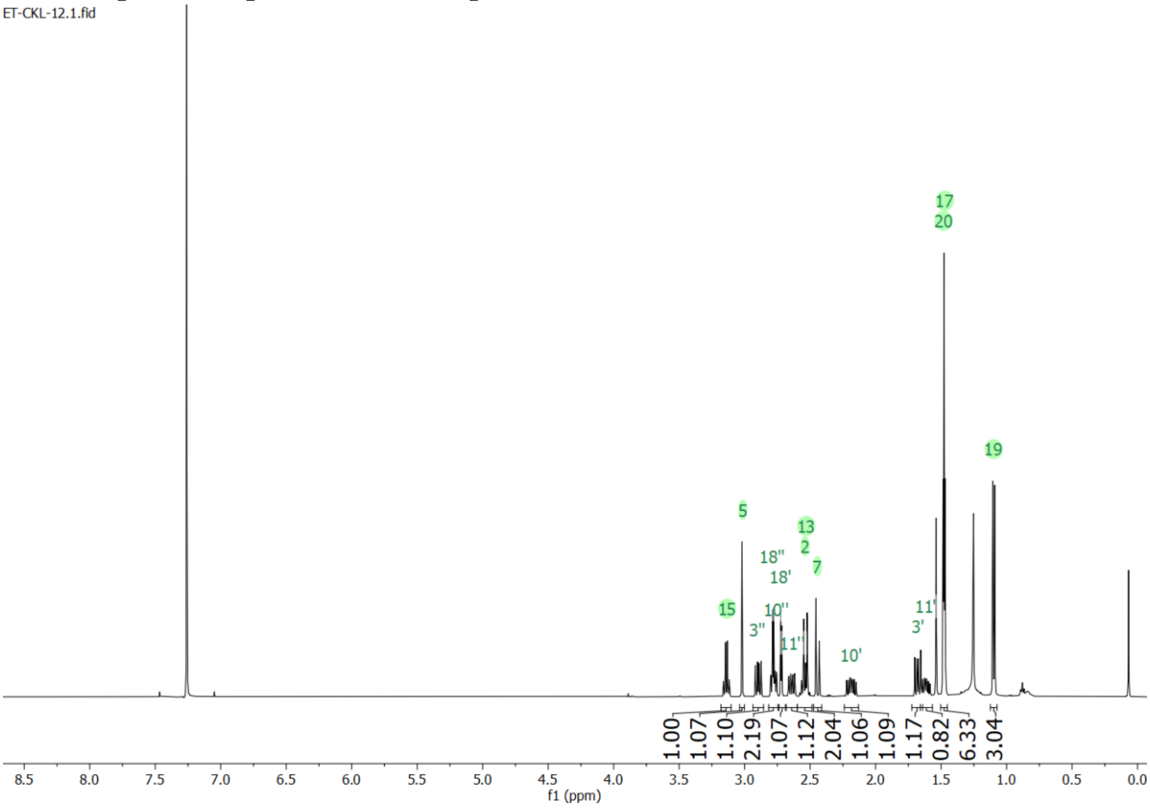

Figure S34. The ^{1}H NMR spectrum of compound **5** (500 MHz, $CDCl_3$)

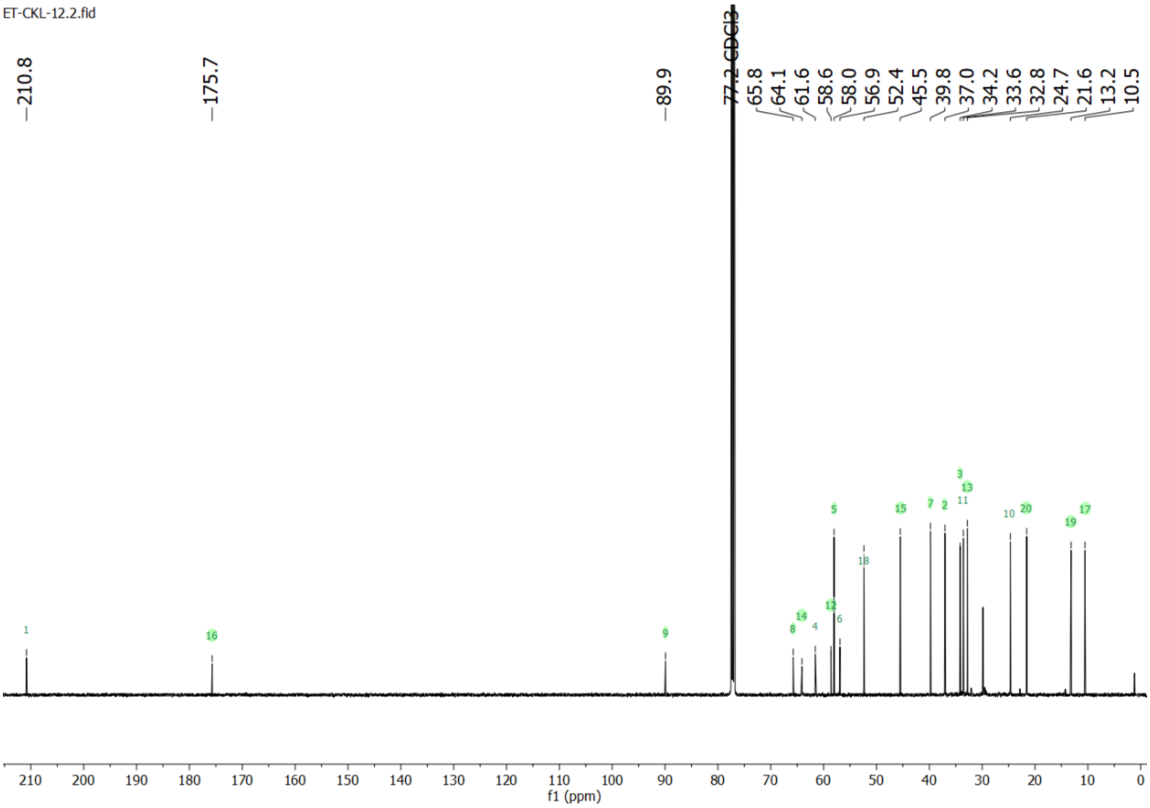

Figure S35. The ^{13}C NMR spectrum of compound **5** (125 MHz, $CDCl_3$)

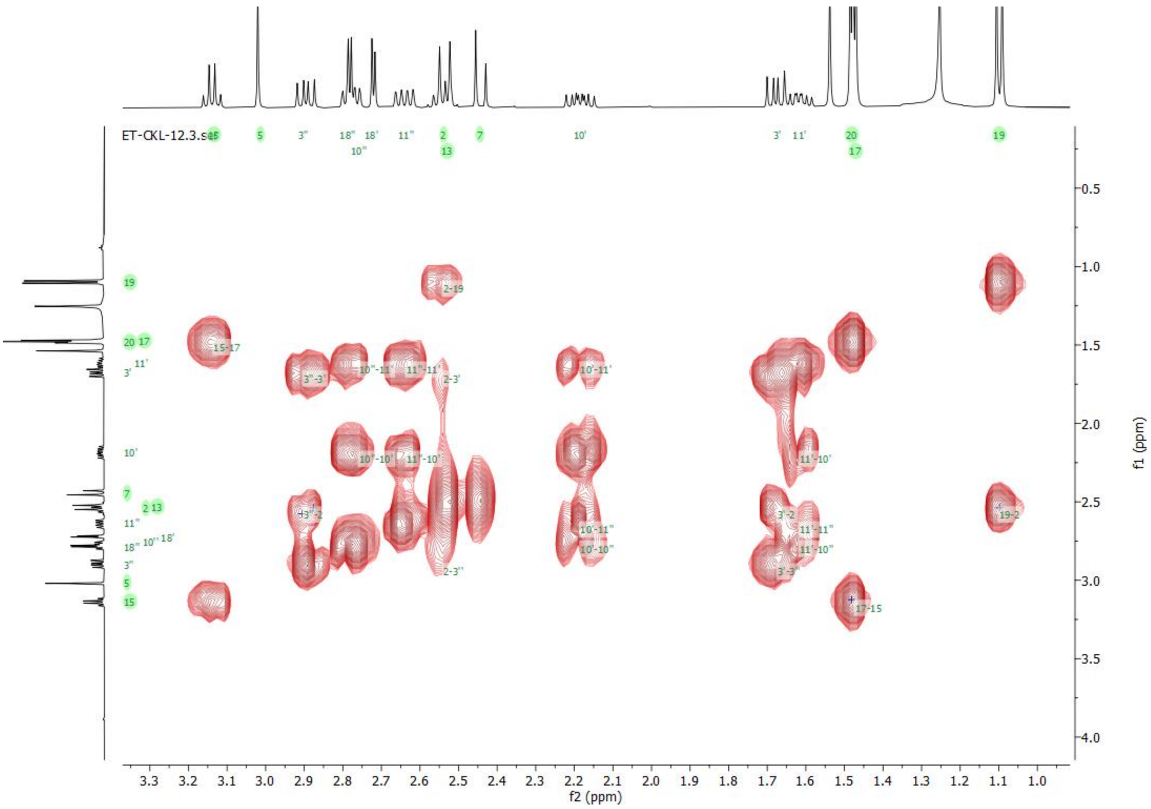

Figure S36. The COSY spectrum of compound **5** (500 MHz, $CDCl_3$)

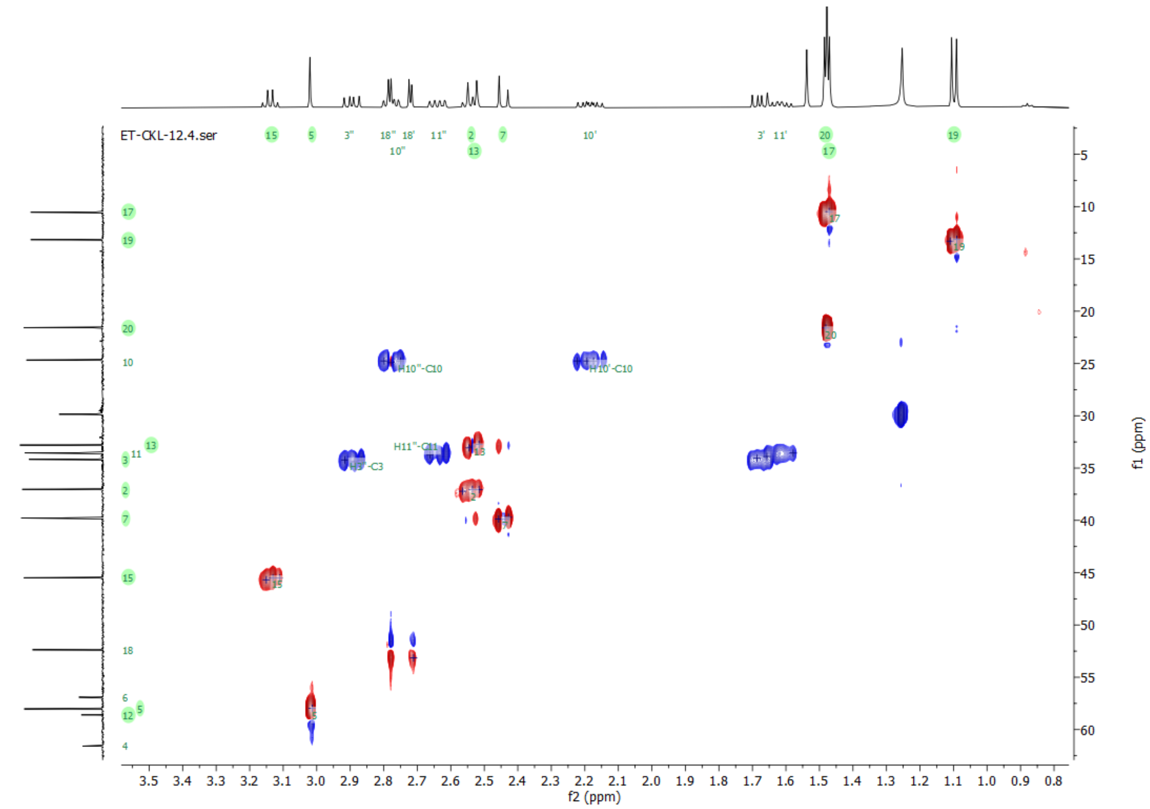

Figure 37. The HSQC spectrum of compound **5** (500/125 MHz, $CDCl_3$)

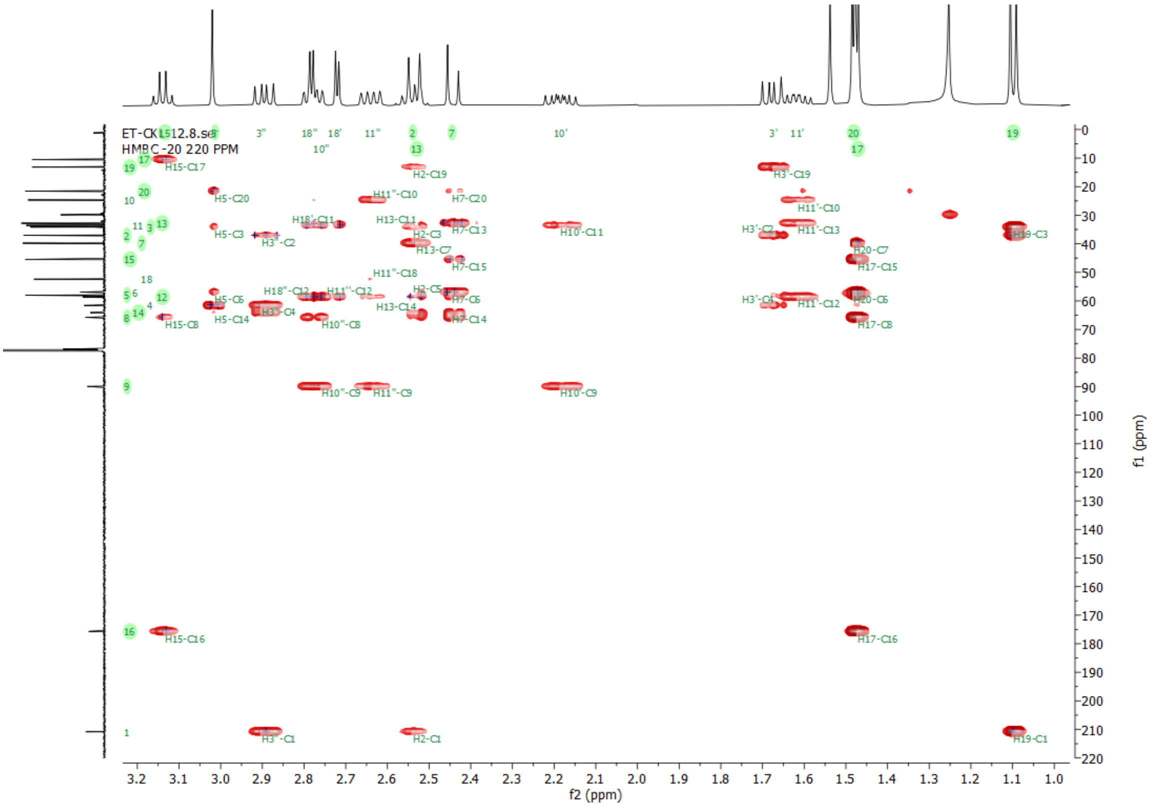

Figure S38. The HMBC spectrum of compound **5** (500 MHz, $CDCl_3$)

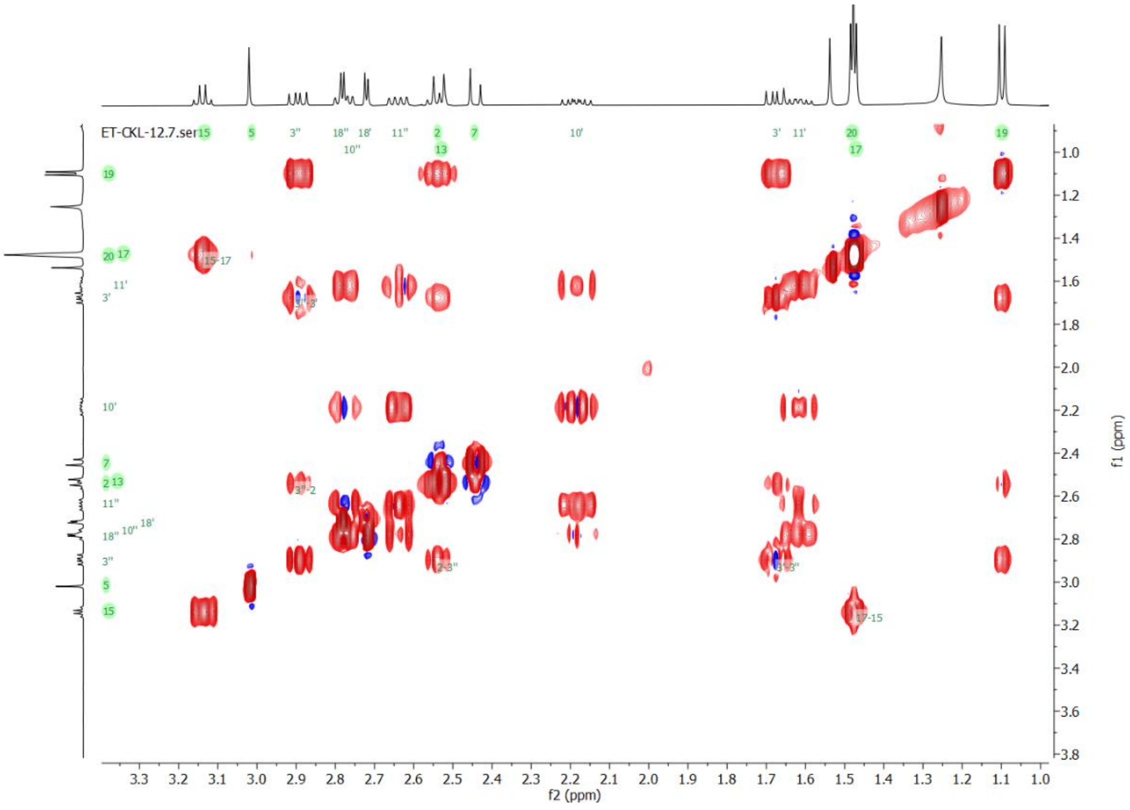

Figure S39. The TOCSY spectrum of compound **5** (500 MHz, CDCl3)

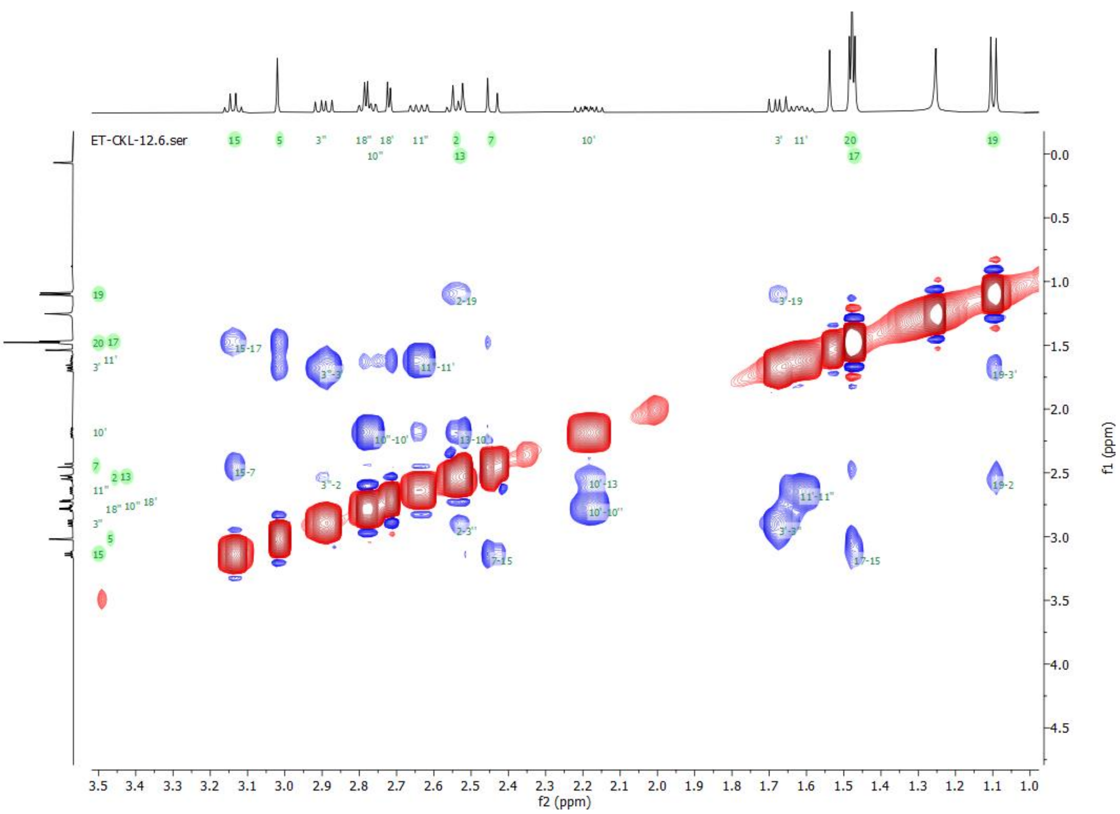

Figure S40. The NOESY spectrum of compound **5** (500 MHz, $CDCl_3$)

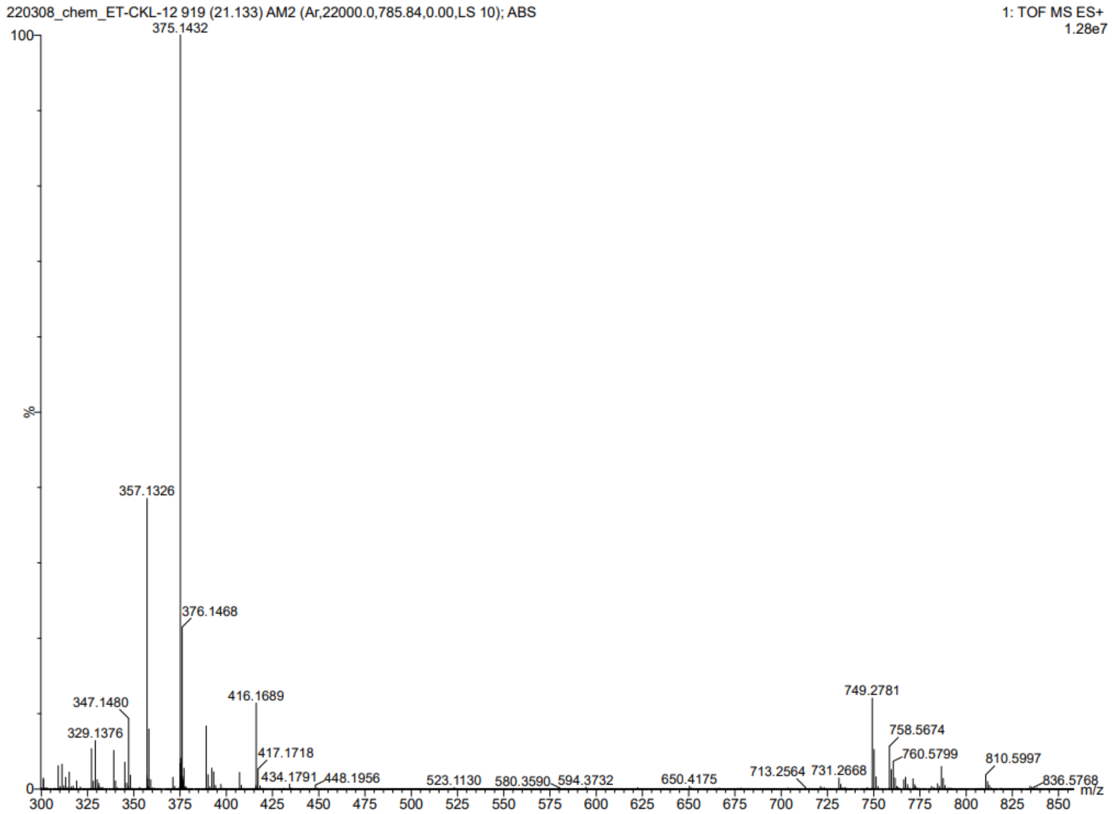

Figure S41. The HRESIMS of compound **5**

7. Spectroscopic data for compound 6

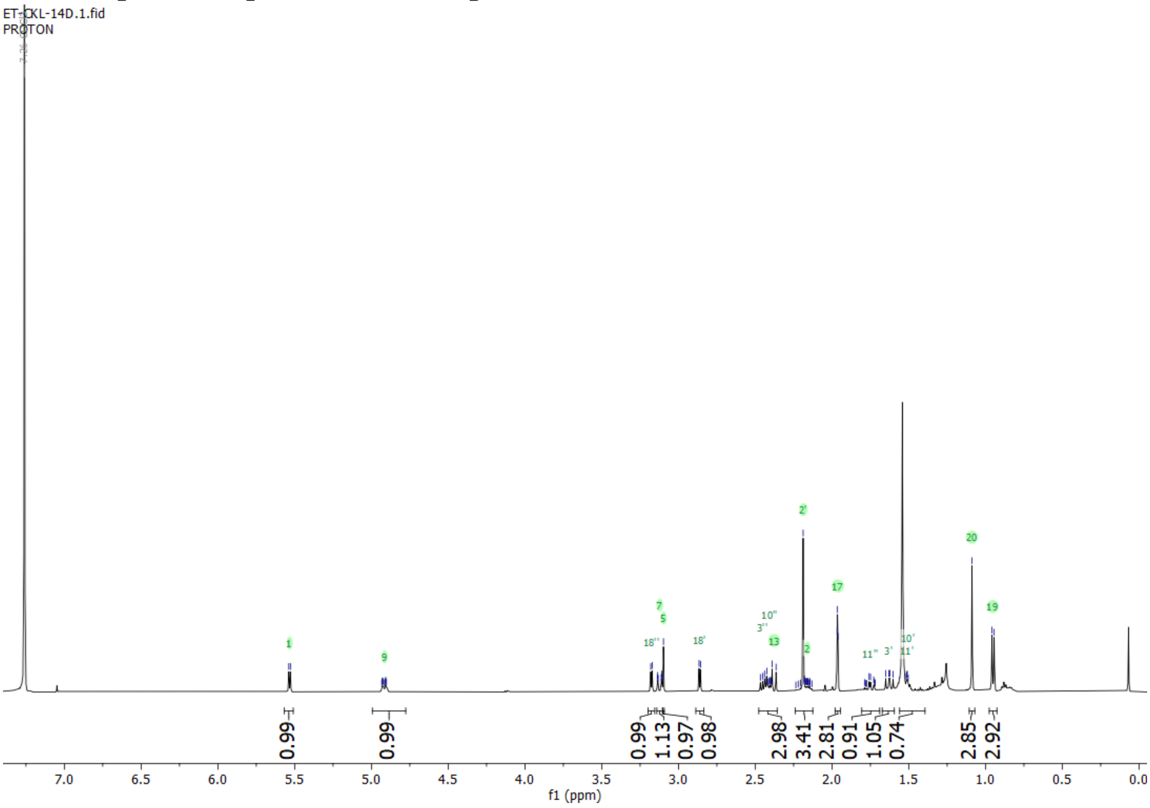

Figure S42. The ^{1}H NMR spectrum of compound **6** (500 MHz, $CDCl_3$)

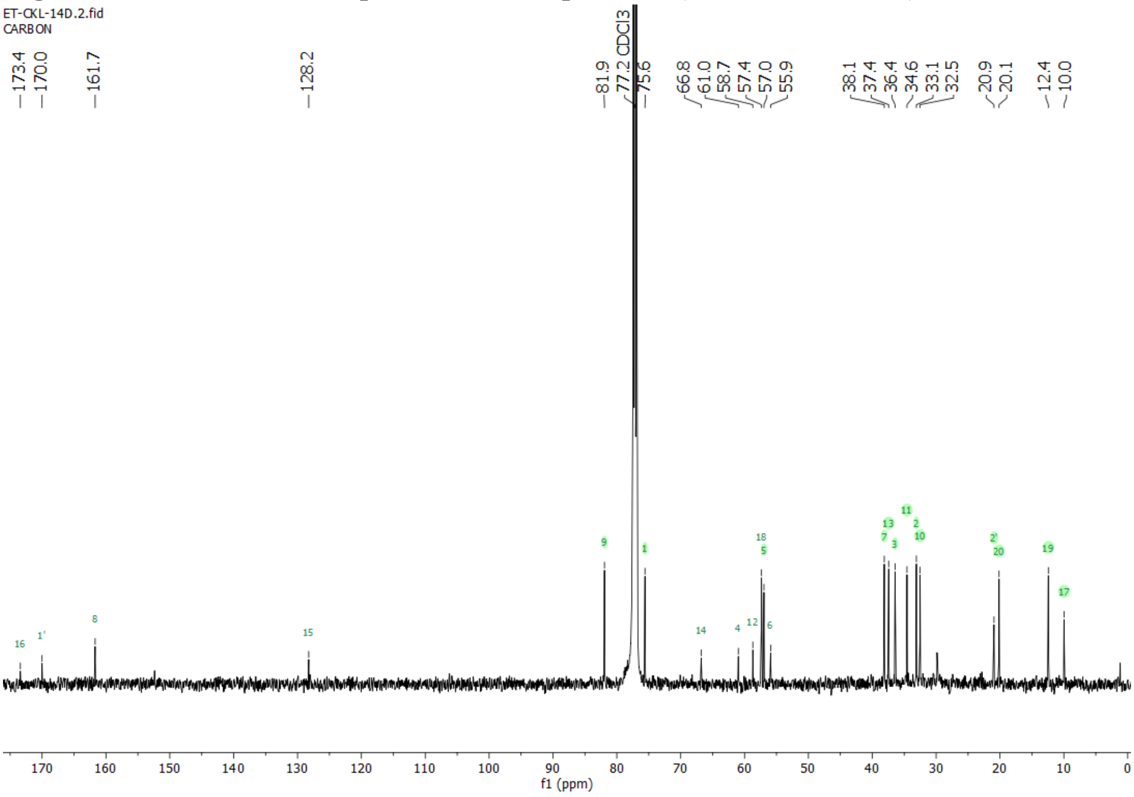

Figure S43. The ^{13}C NMR of compound **6** (125 MHz, $CDCl_3$)

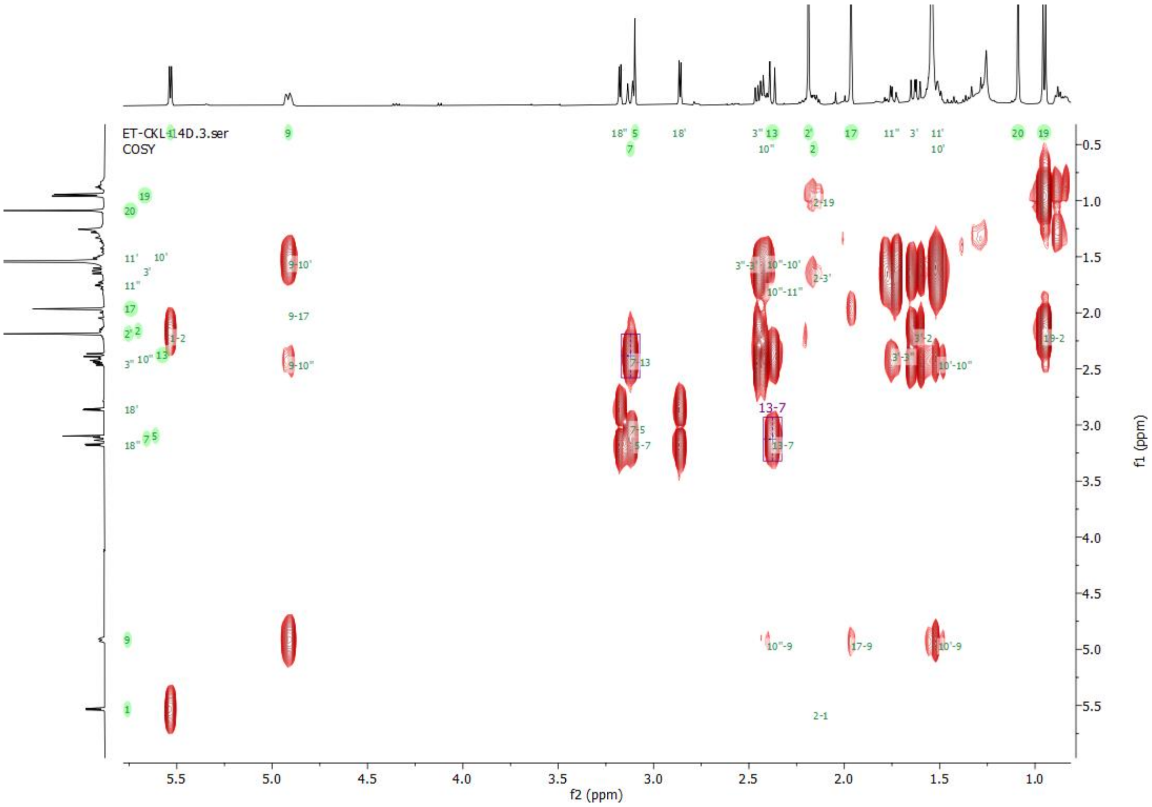

Figure S44. The COSY spectrum of compound **6** (500 MHz, $CDCl_3$)

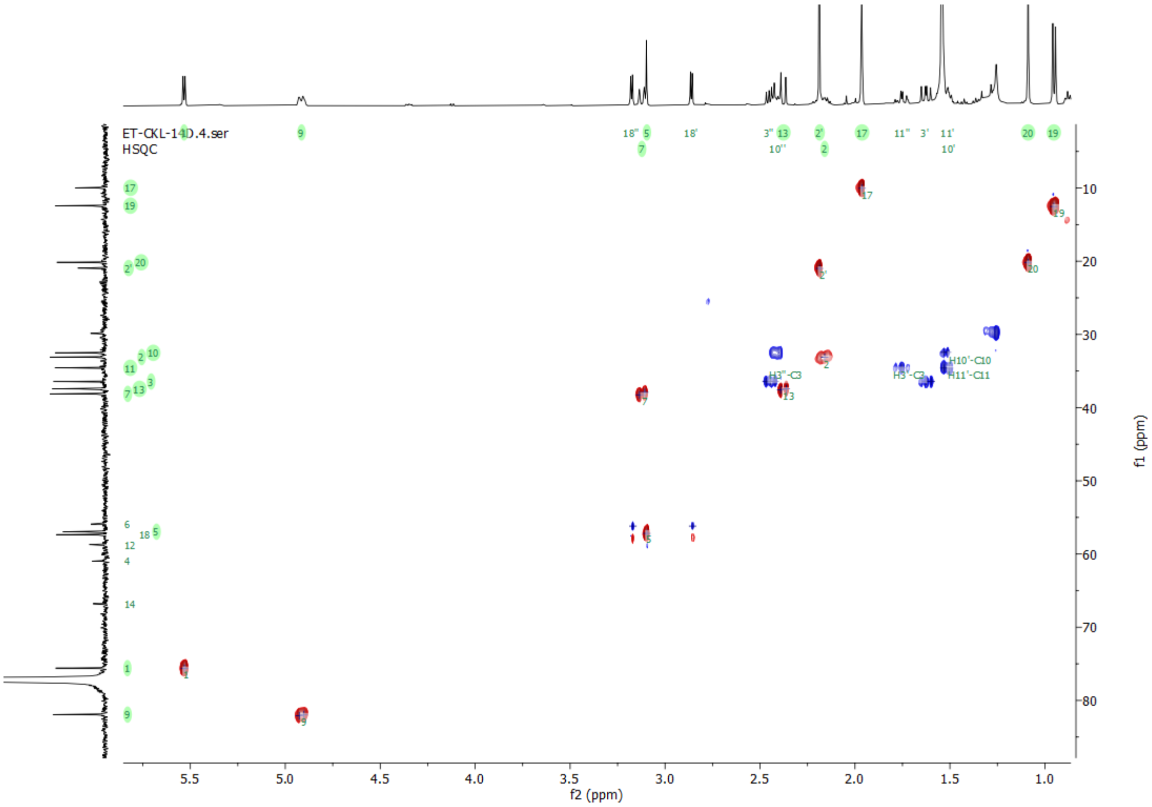

Figure S45. The HSQC spectrum of compound **6** (500 MHz, $CDCl_3$)

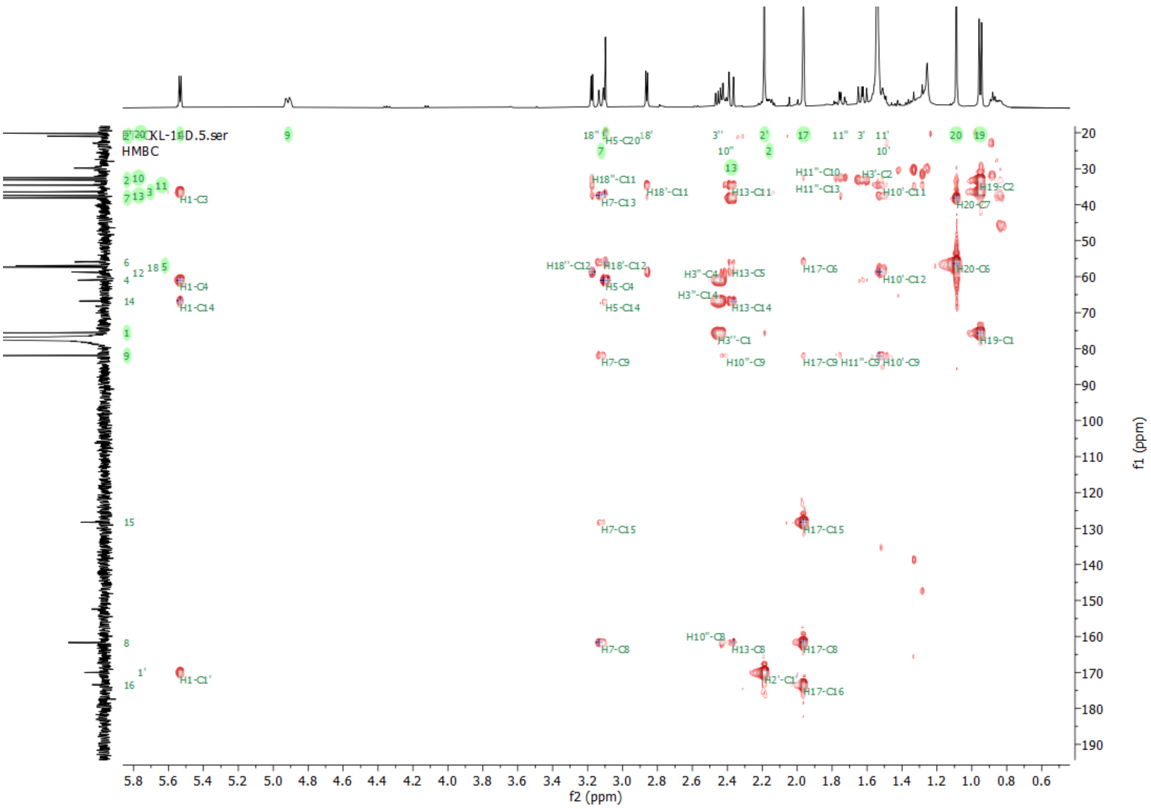

Figure S46. The HMBC spectrum of compound **6** (500/125 MHz, $CDCl_3$)

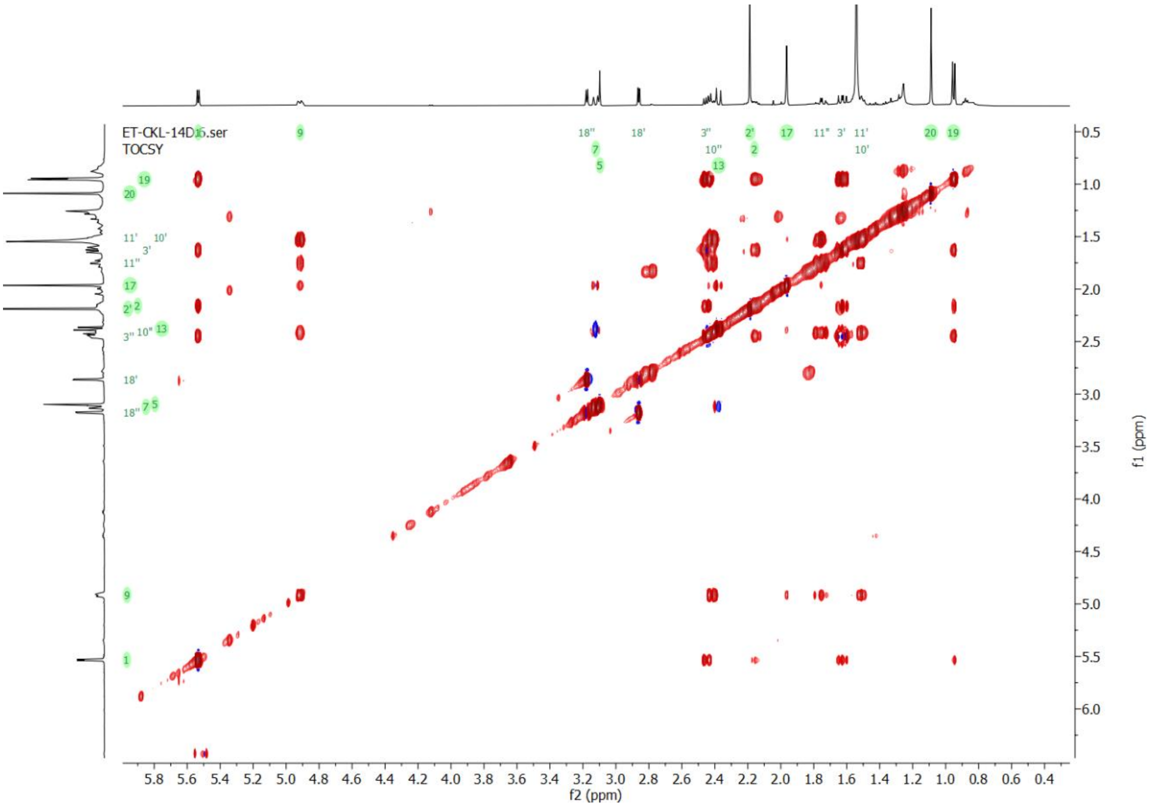

Figure S47. The TOCSY spectrum of compound **6** (500 MHz, $CDCl_3$)

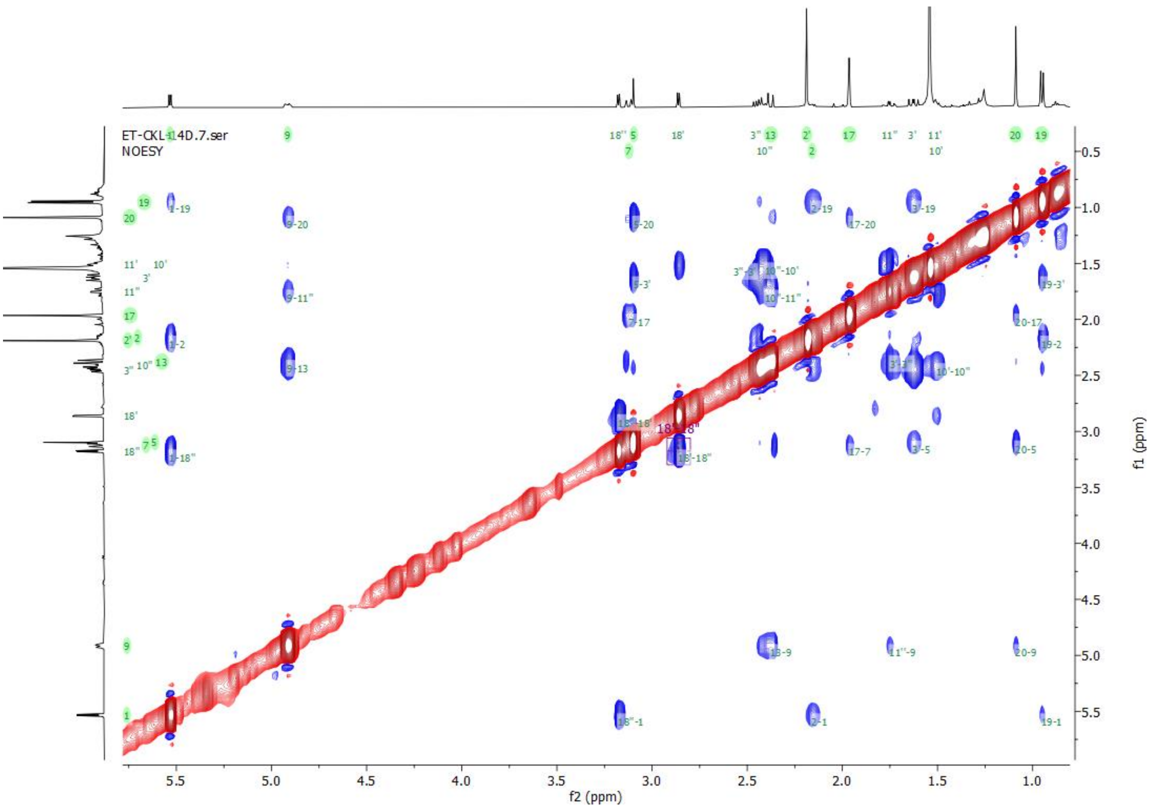

Figure S48. The NOESY spectrum of compound **6** (500 MHz, $CDCl_3$)

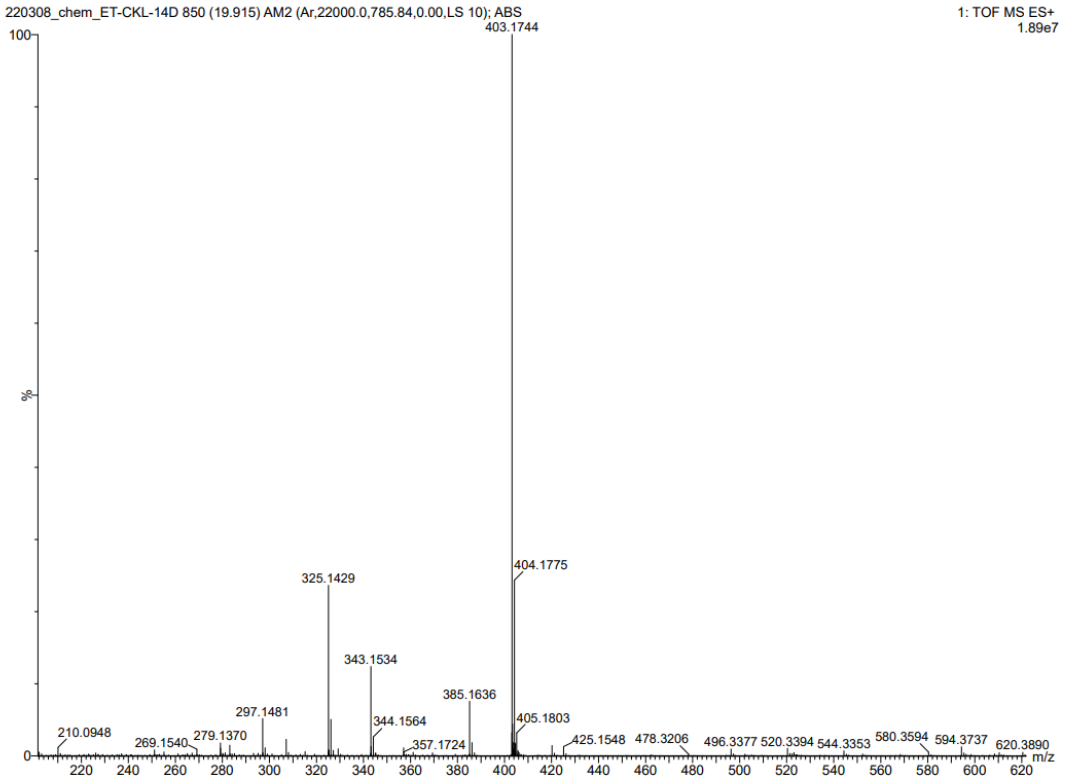

Figure S49. The HRESIMS of compound **6**

8. Spectroscopic data of compound 7

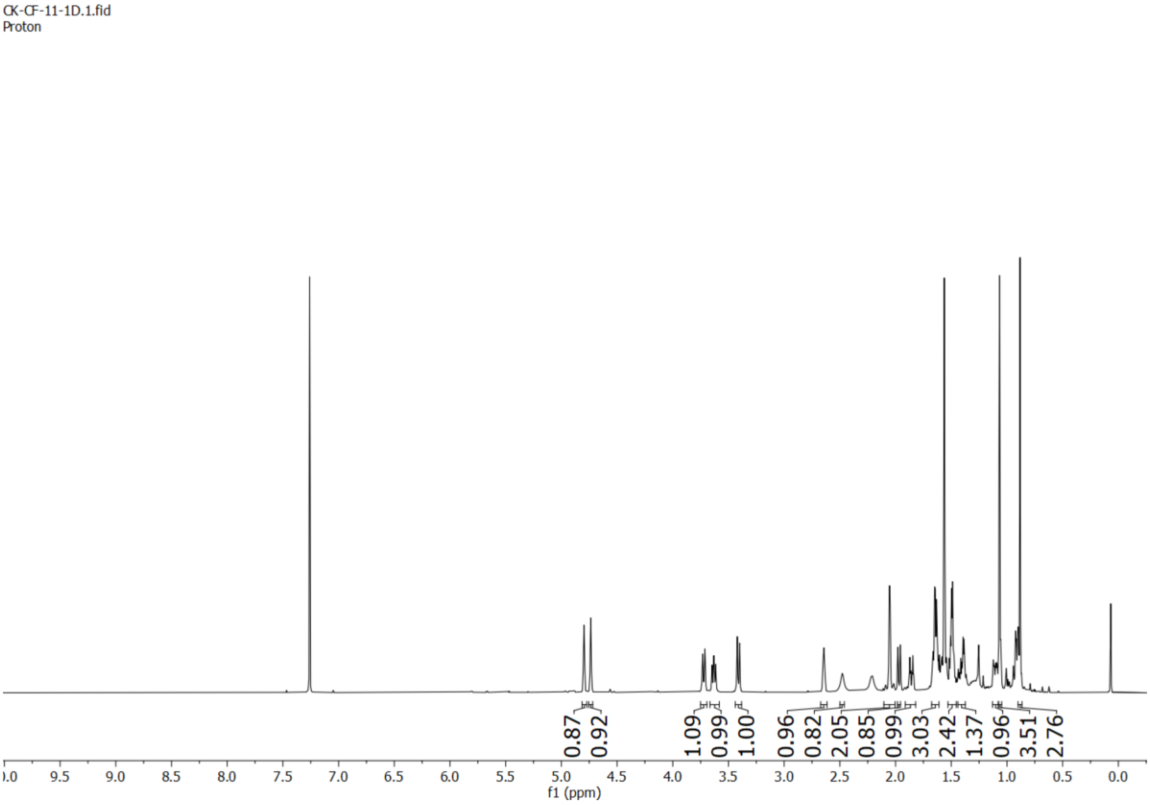

Figure S50. The ^{1}H NMR spectrum of compound **7** (500 MHz, $CDCl_3$)

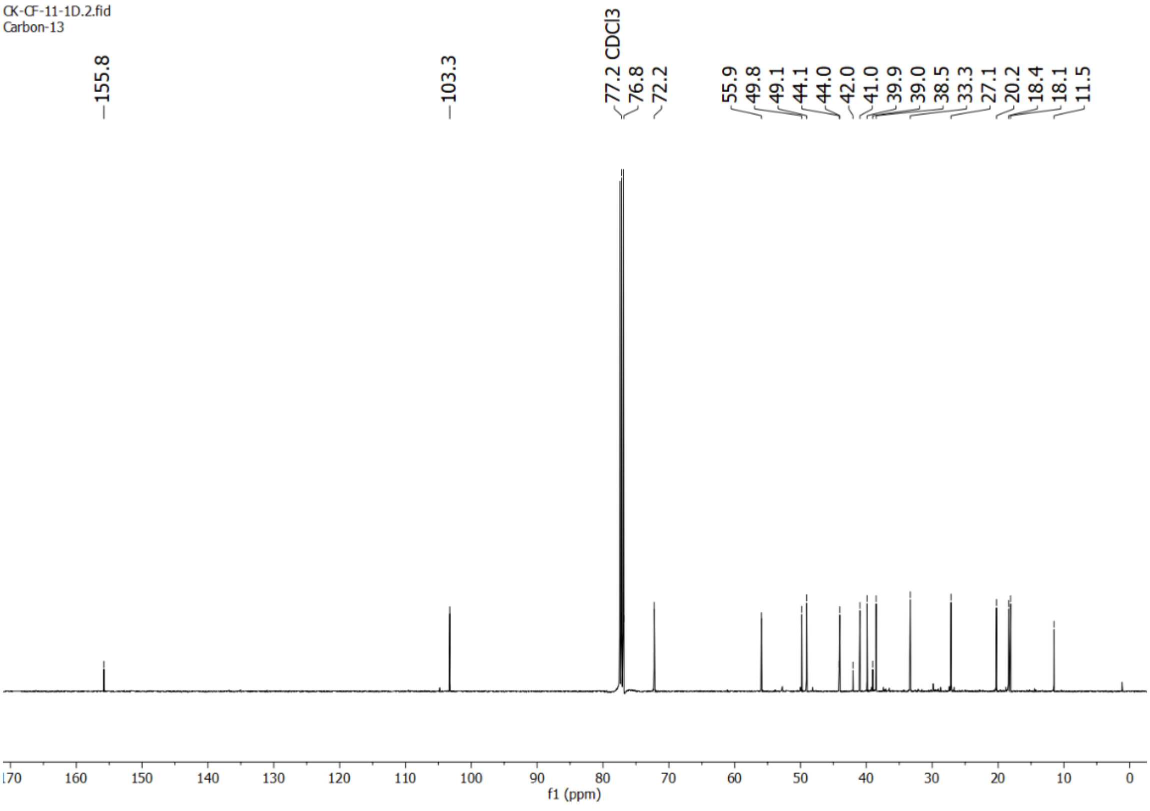

Figure S51. The ^{13}C NMR spectrum of compound **7** (125 MHz, $CDCl_3$)

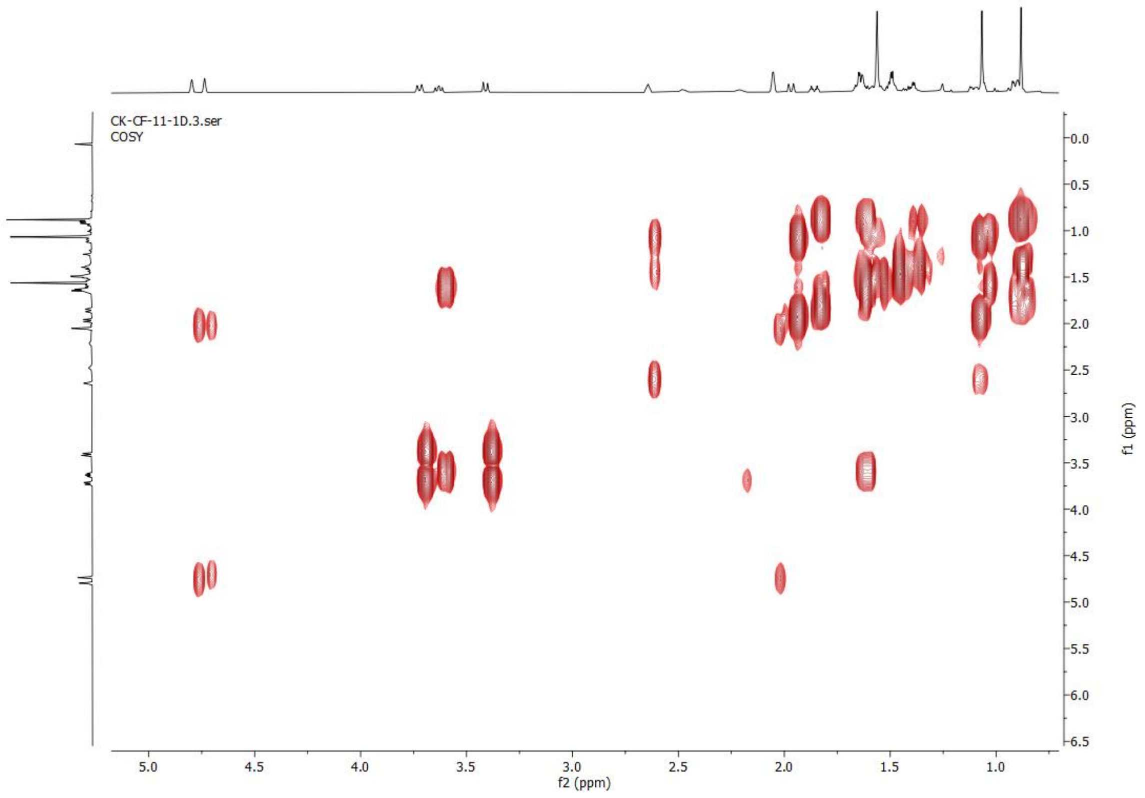

Figure S52. The COSY spectrum of compound **7** (500 MHz, $CDCl_3$)

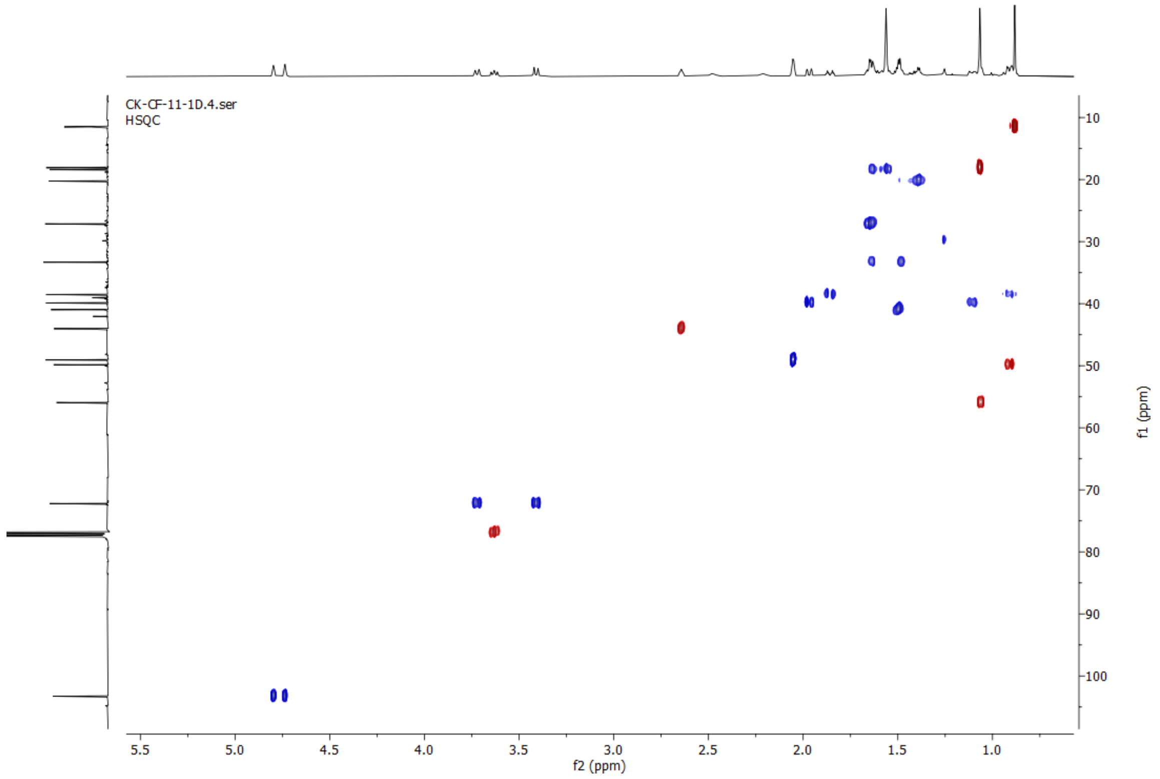

Figure S53. The HSQC spectrum of compound **7** (500/125 MHz, $CDCl_3$)

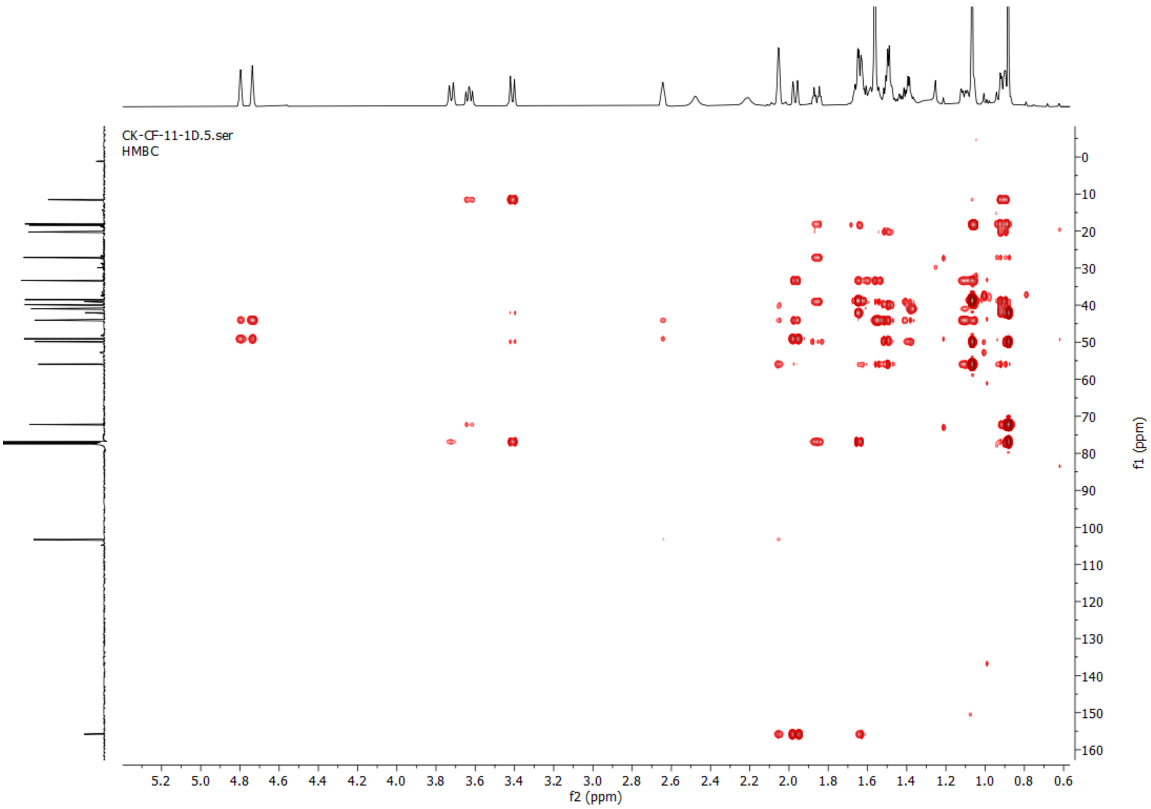

Figure S54. The HMBC spectrum of compound **7** (500 MHz, $CDCl_3$)

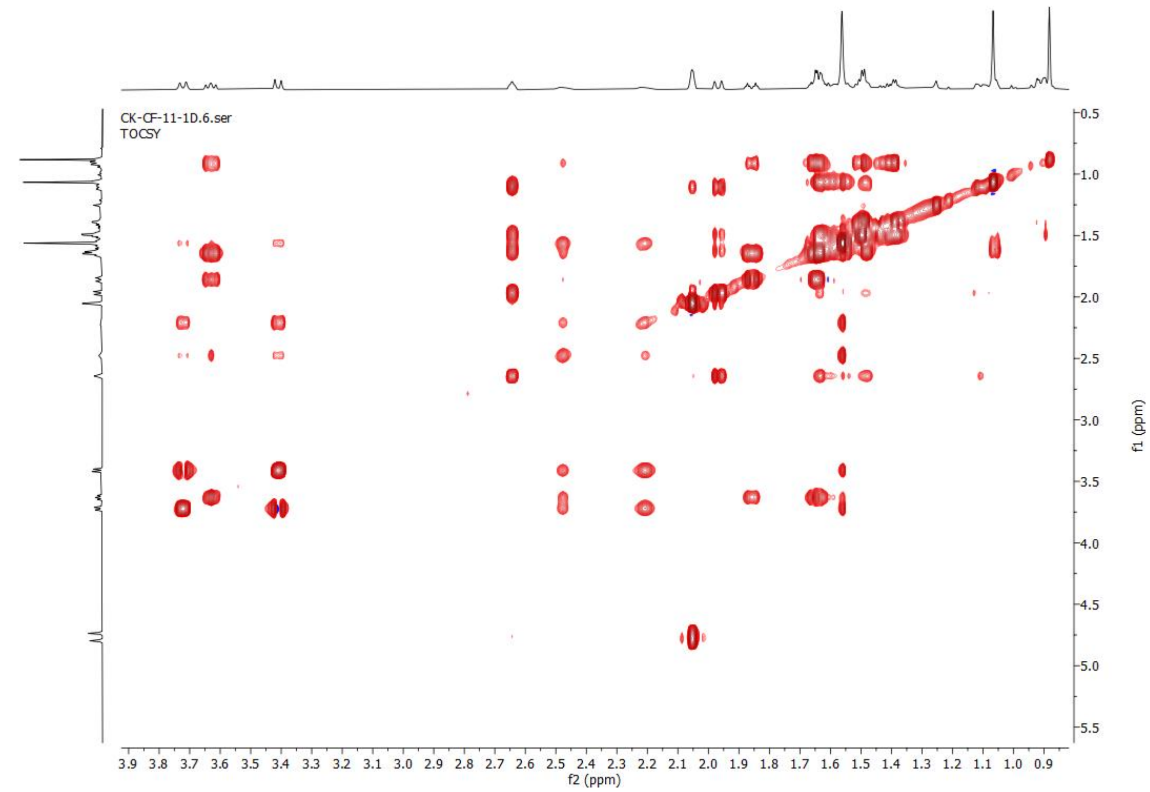

Figure S55. The TOCSY spectrum of compound **7** (500 MHz, $CDCl_3$)

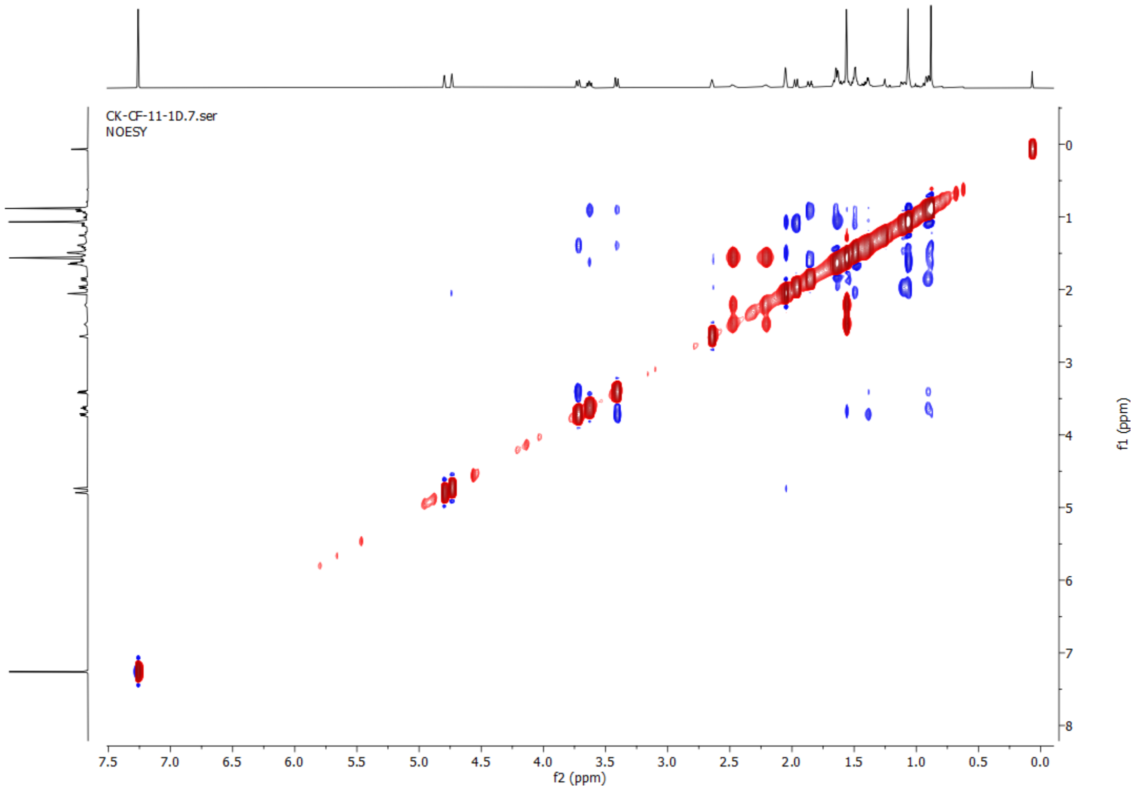

Figure S56. The NOESY Spectrum of compound **7** (500 MHz, $CDCl_3$)

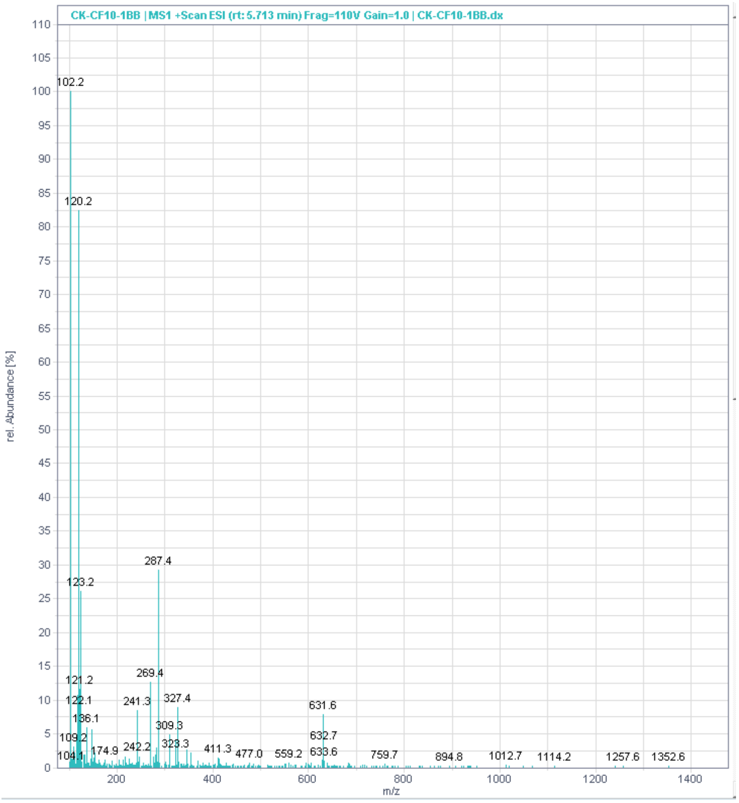

Figure S57. The LC-MS of compound **7**.

9. Spectroscopic data for compound 8

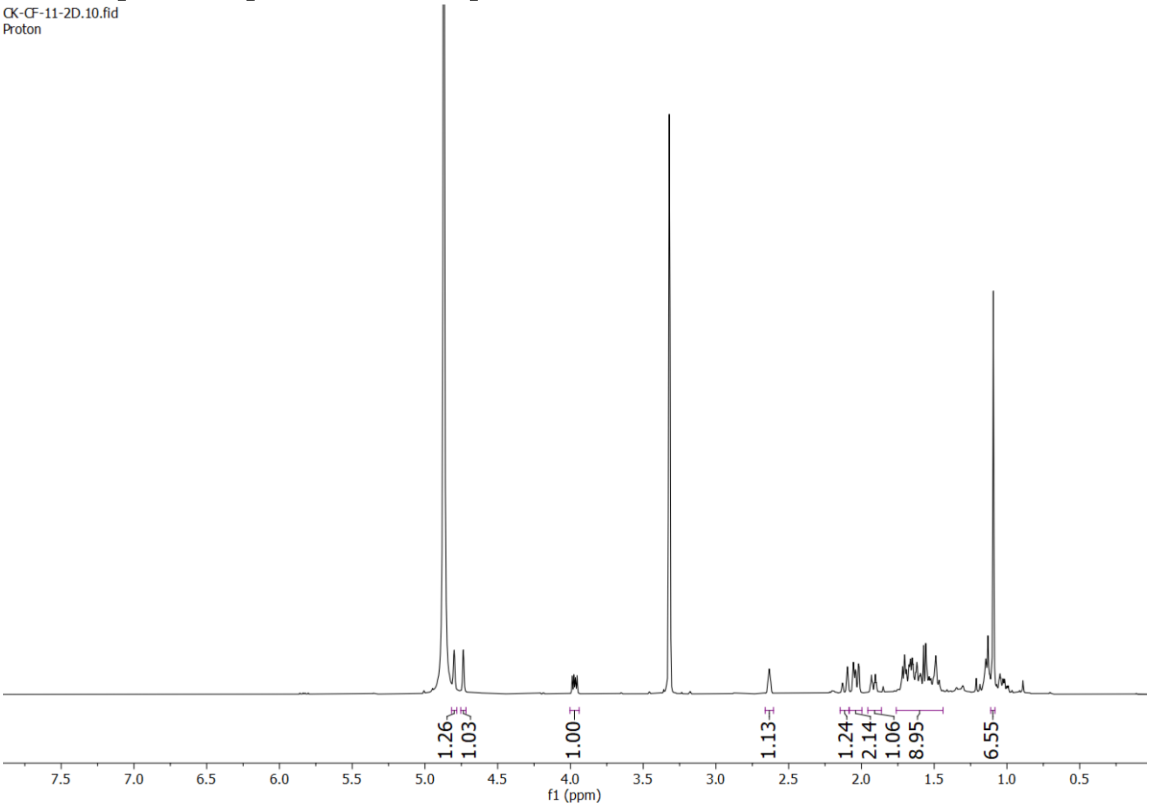

Figure S58. The ^{1}H NMR spectrum of compound **8** (500 MHz, CD_3OD)

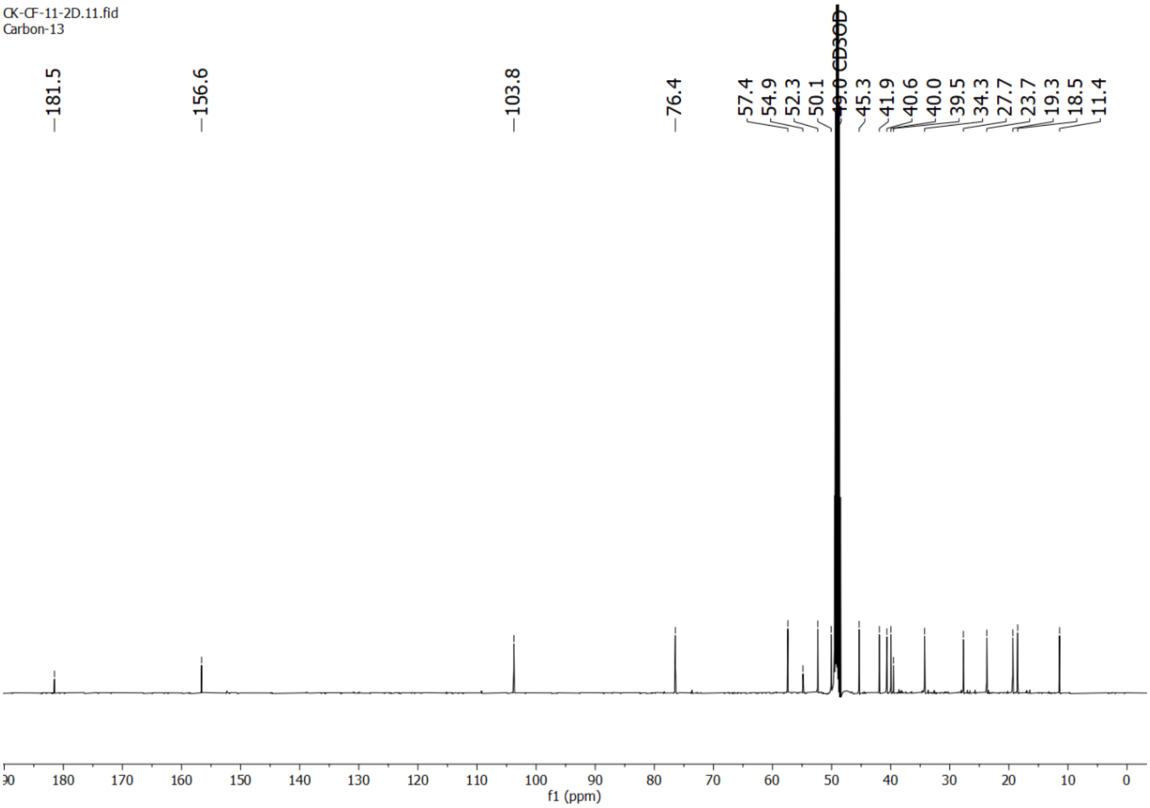

Figure S59. The ^{13}C NMR spectrum of compound **8** (125 MHz, CD_30D)

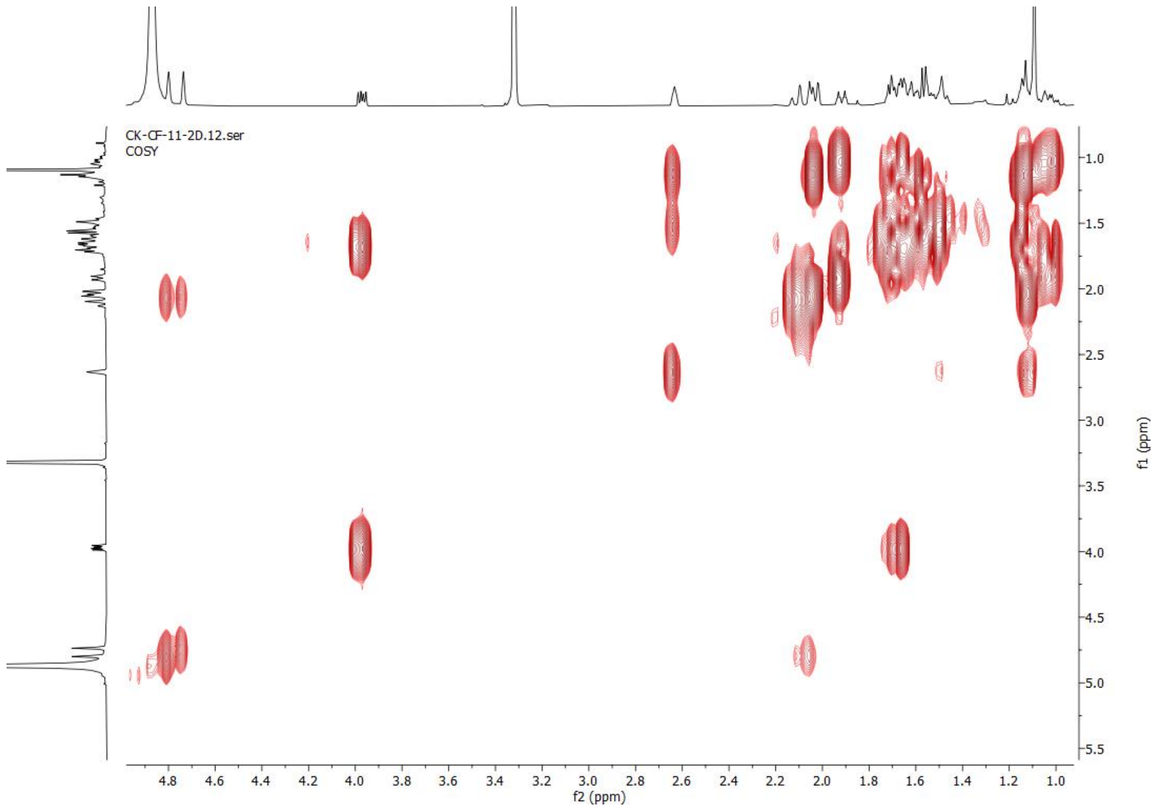

Figure S60. The COSY Spectrum of compound **8** (500 MHz, CD_3OD)

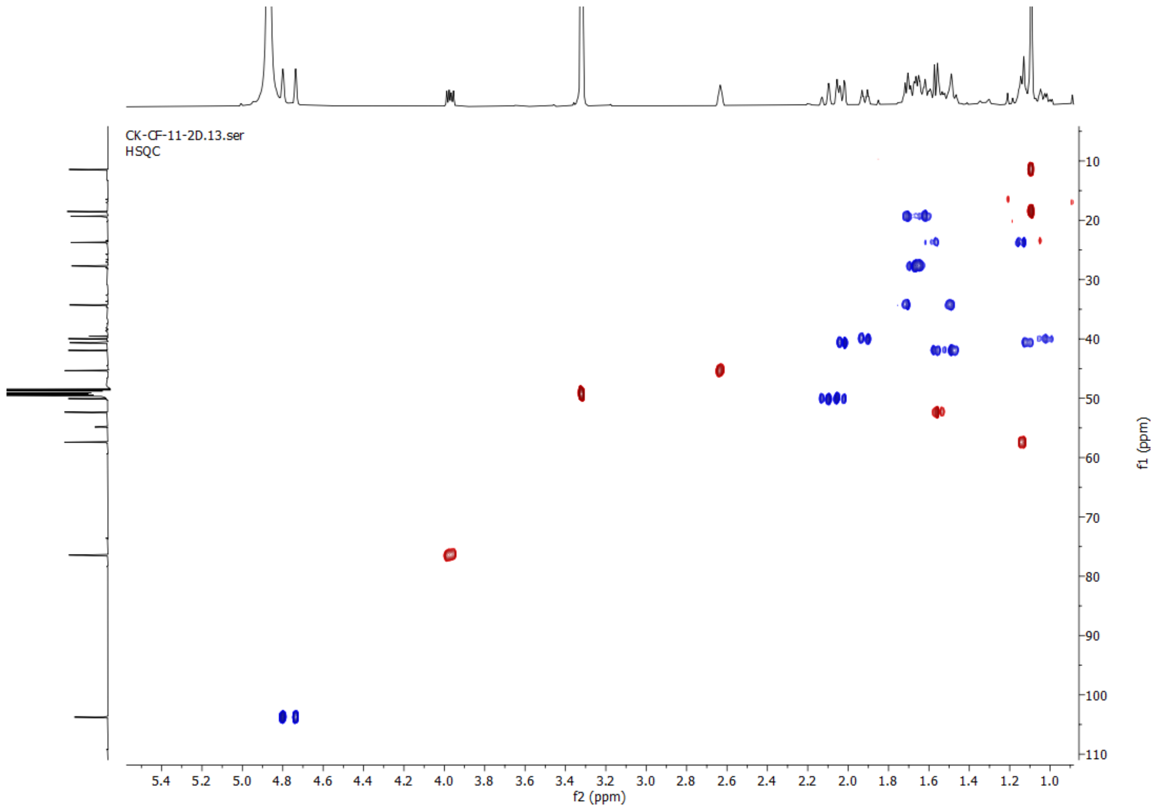

Figure S61. The HSQC spectrum of compound **8** (500/125 MHz, CD_3OD)

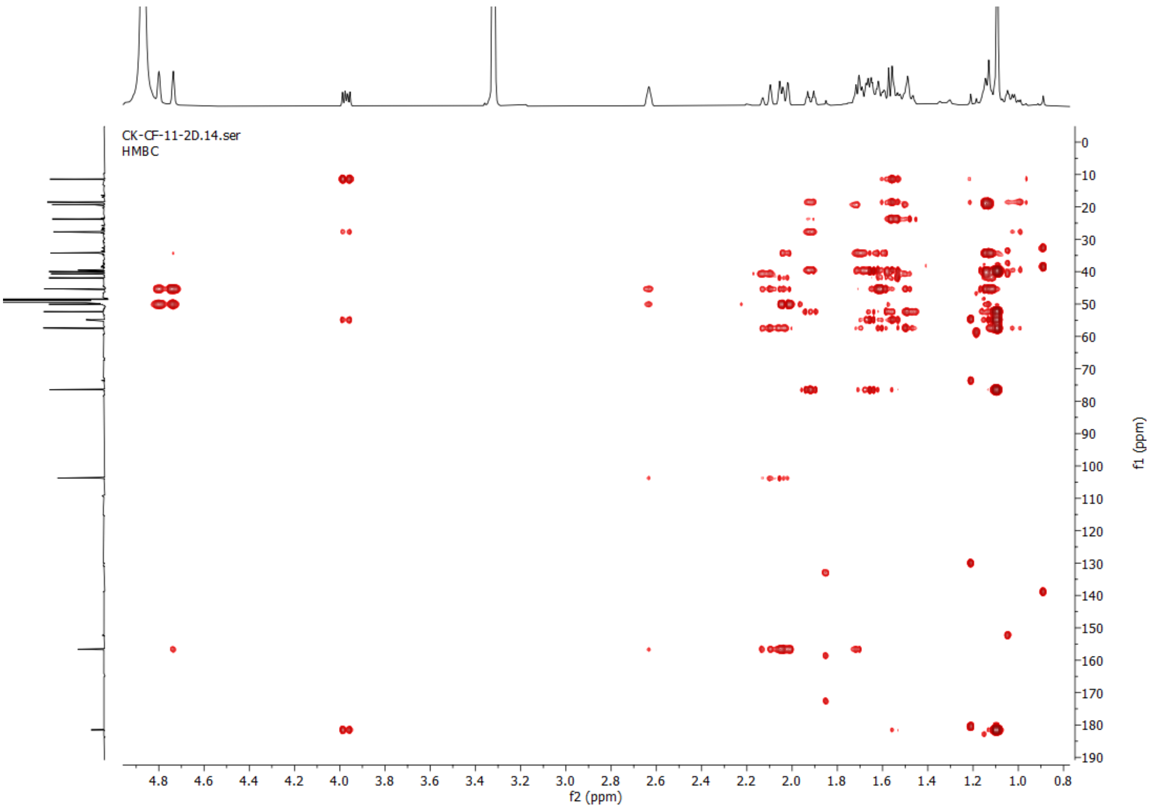

Figure S62. The HMBC spectrum of compound **8** (500/125 MHz, CD_3OD)

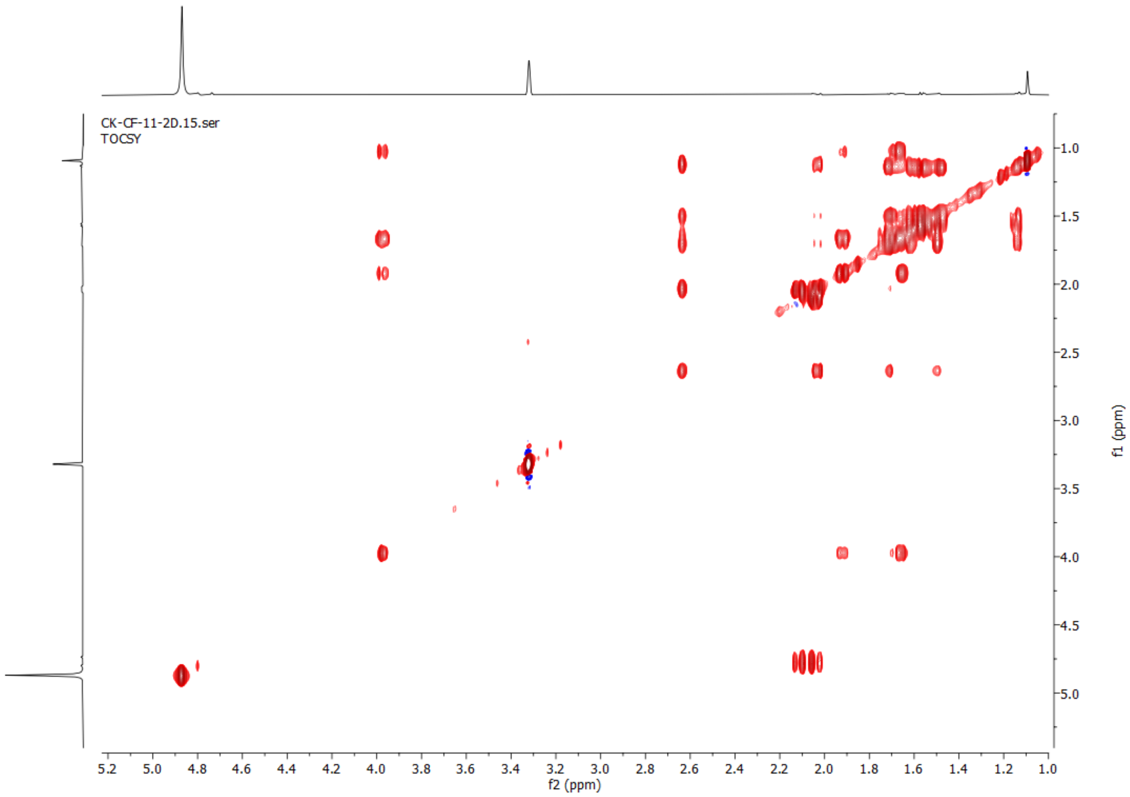

Figure S63. The TOSY spectrum of compound **8** (500 MHz, CD_3OD)

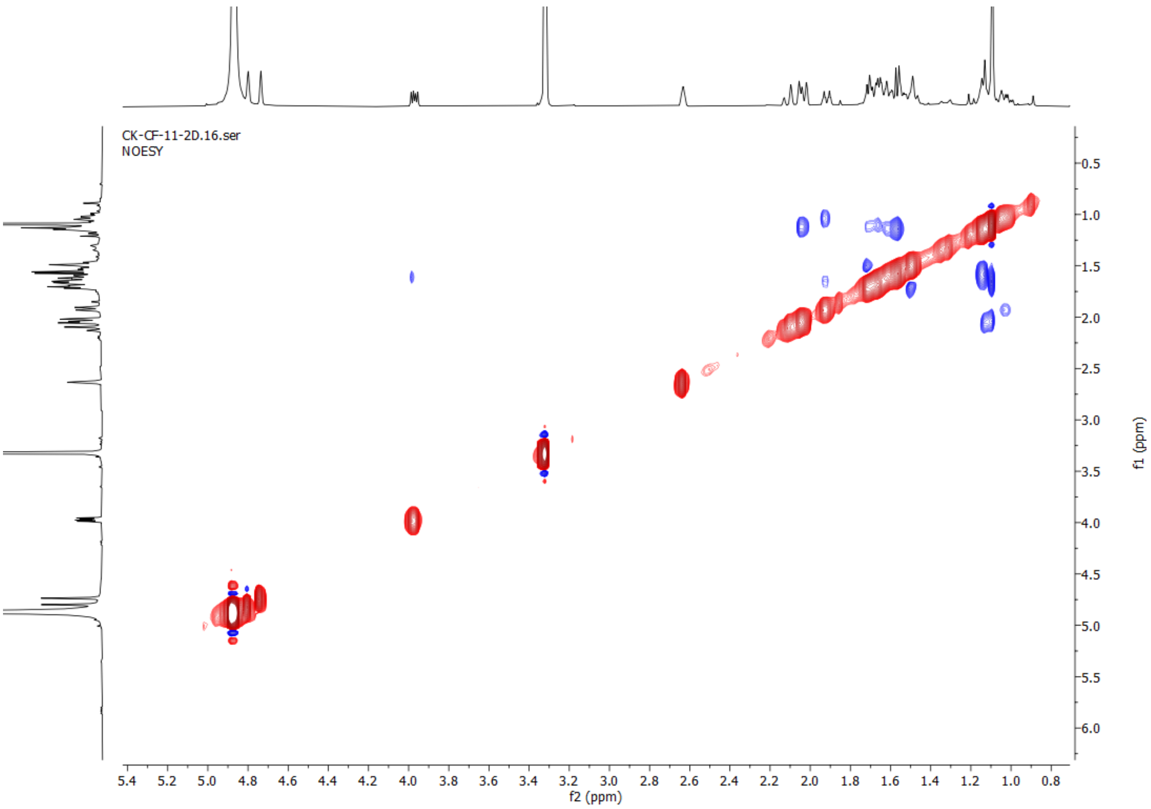

Figure S64. The NOESY spectrum of compound **8** (500 MHz, CD_3OD)

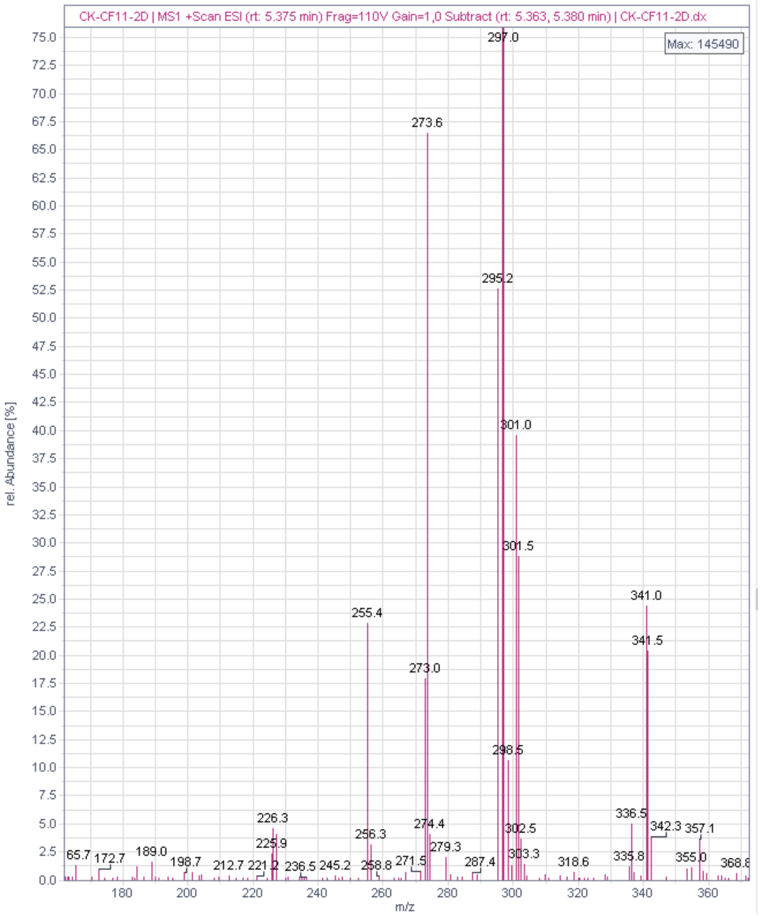

Figure S65. LC-MS of compound **8**

10. Spectroscopic data for compound 9

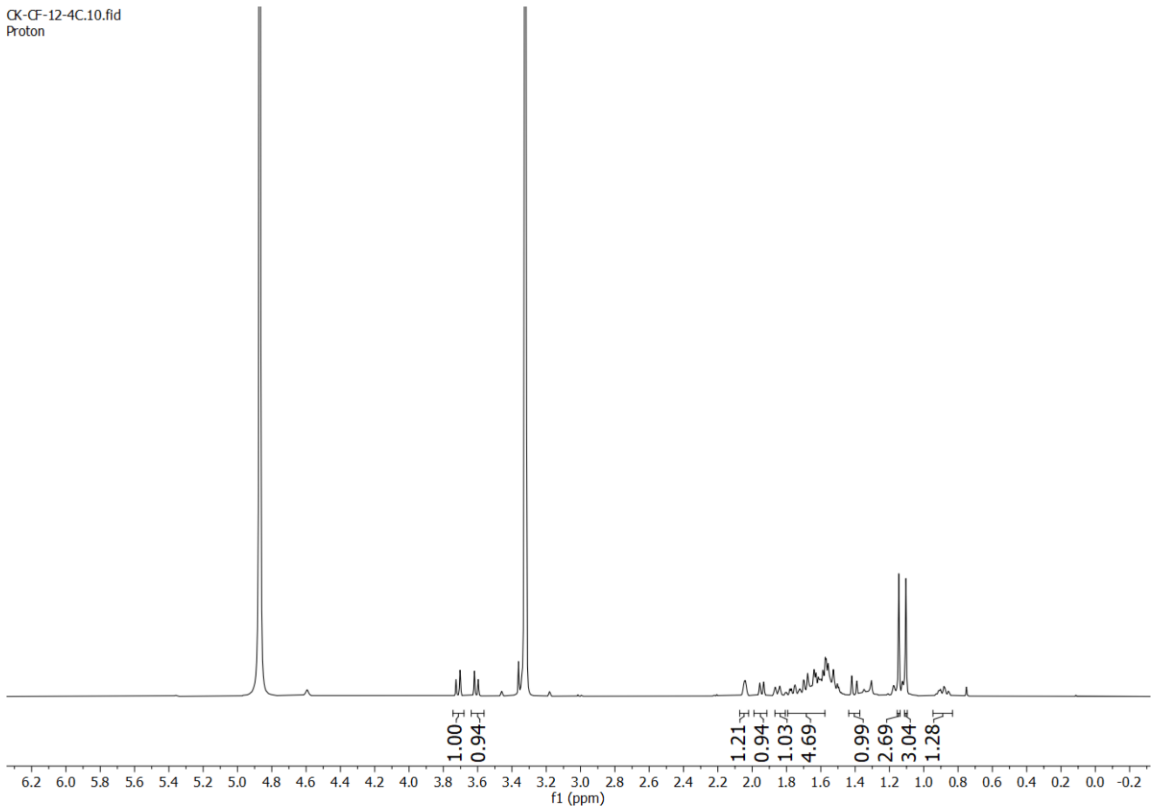

Figure S66. The ^{1}H NMR spectrum for compound **9** (500 MHz, CD_3OD)

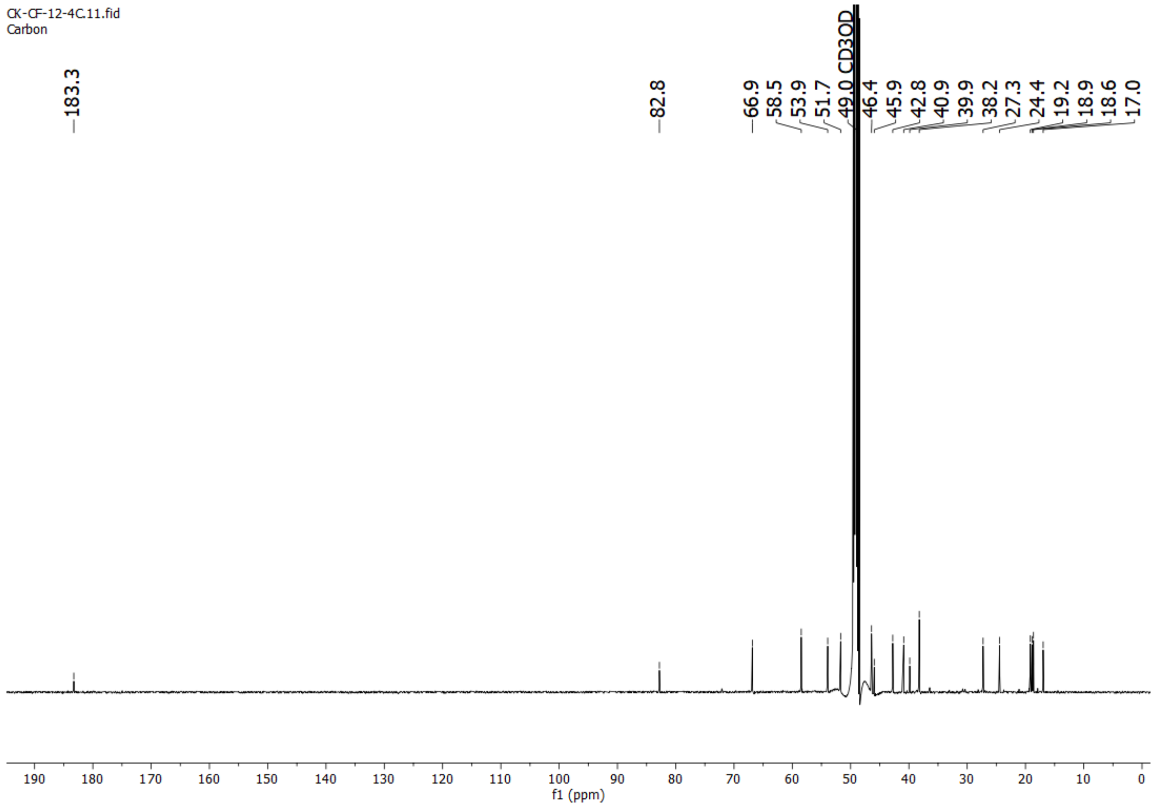

Figure S67. The ^{13}C NMR spectrum of compound **9** (125 MHz, CD_3OD)

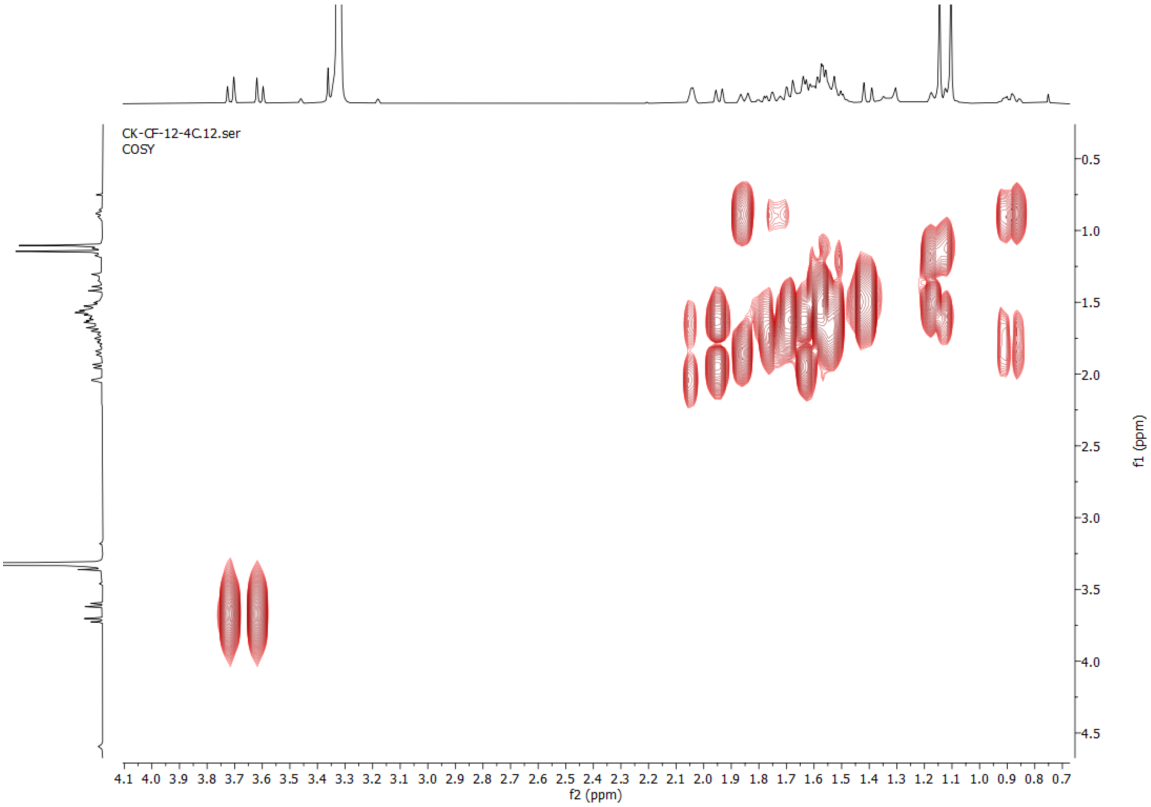

Figure S68. The COSY spectrum of compound **9** (500 MHz, CD_3OD)

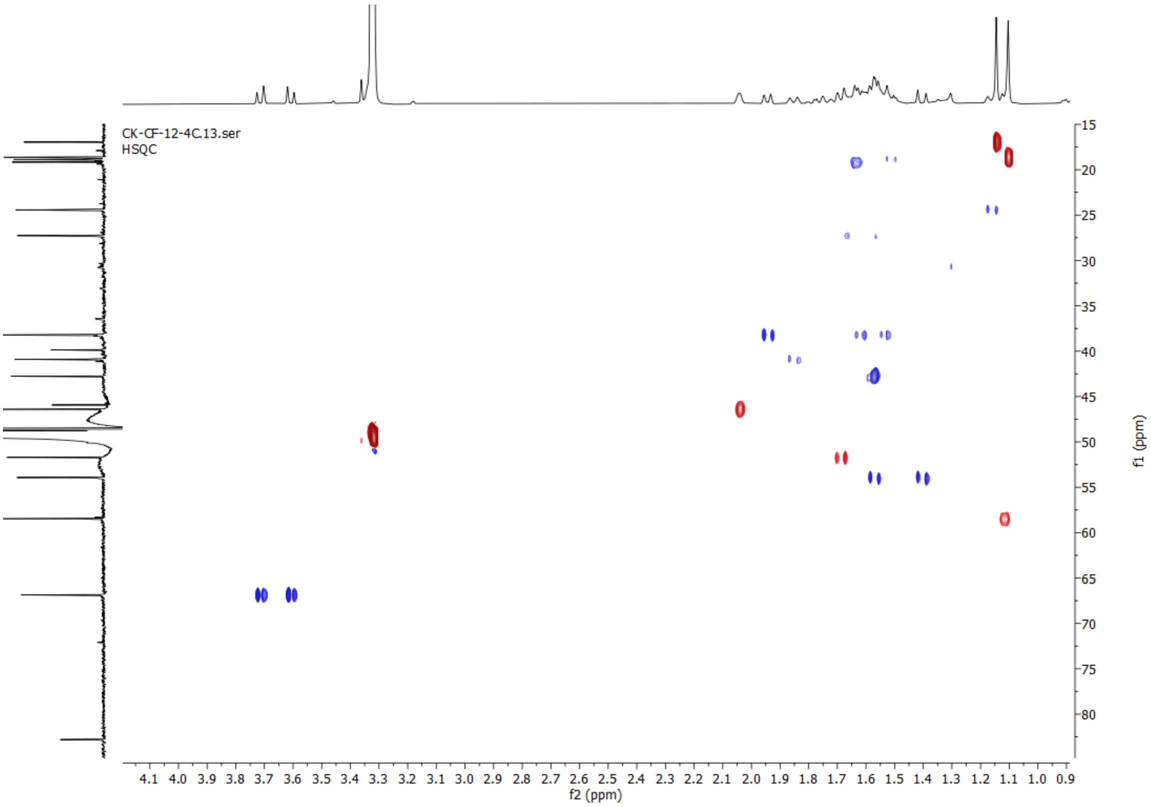

Figure S69. The HSQC spectrum of compound **9** (500/125 MHz, CD_3OD)

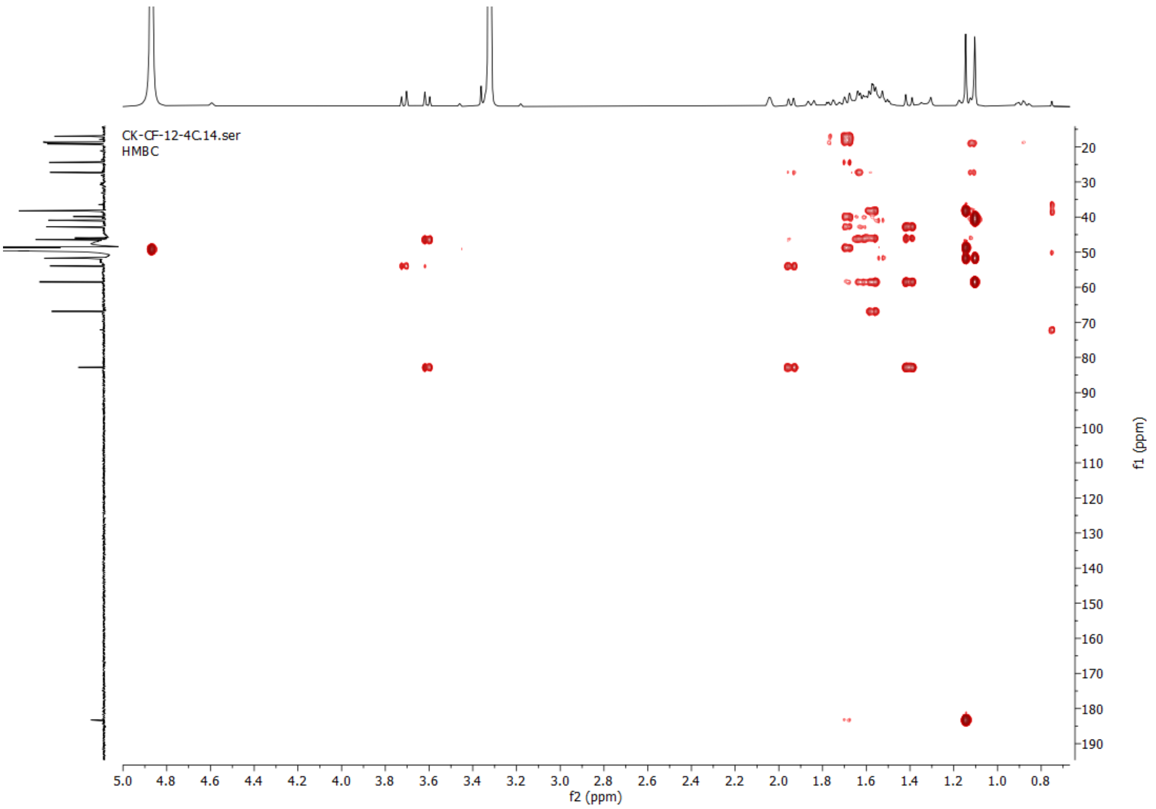

Figure S70. The HMBC spectrum for compound **9** (500/125 MHz, CD_3OD)

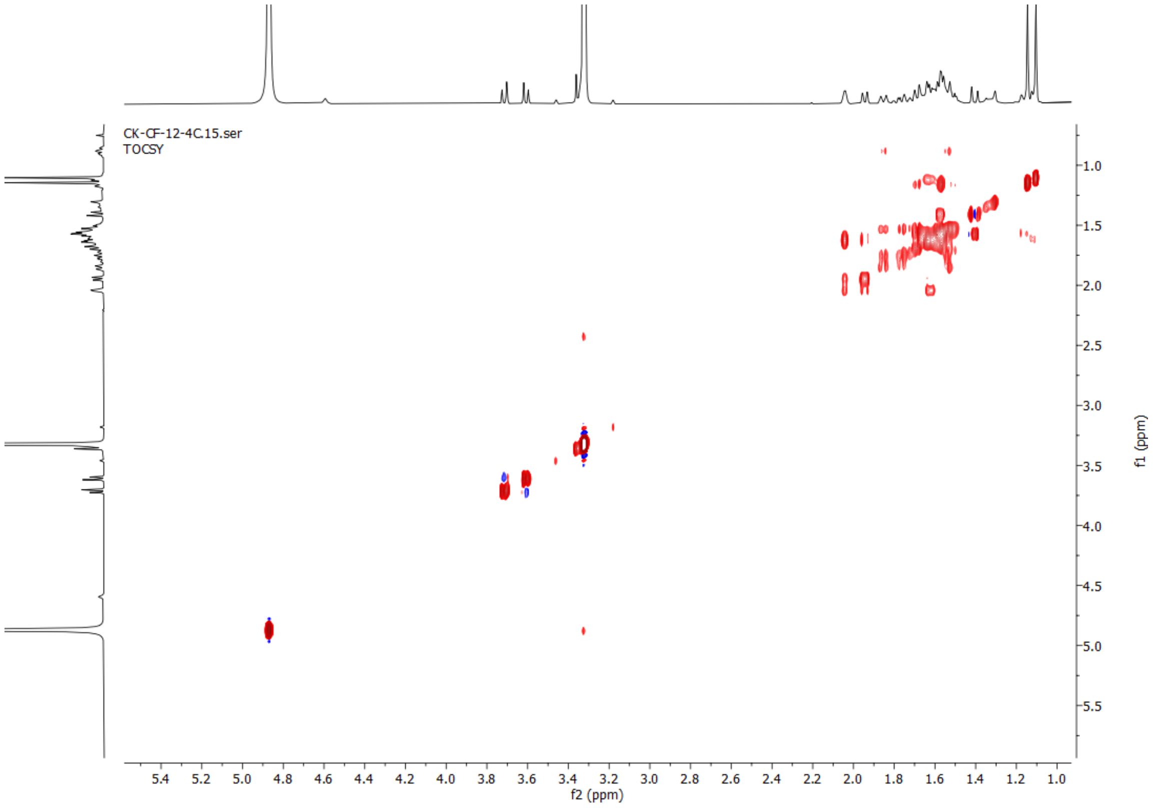

Figure S71. The TOCSY spectrum of compound **9** (500 MHz, CD_3OD)

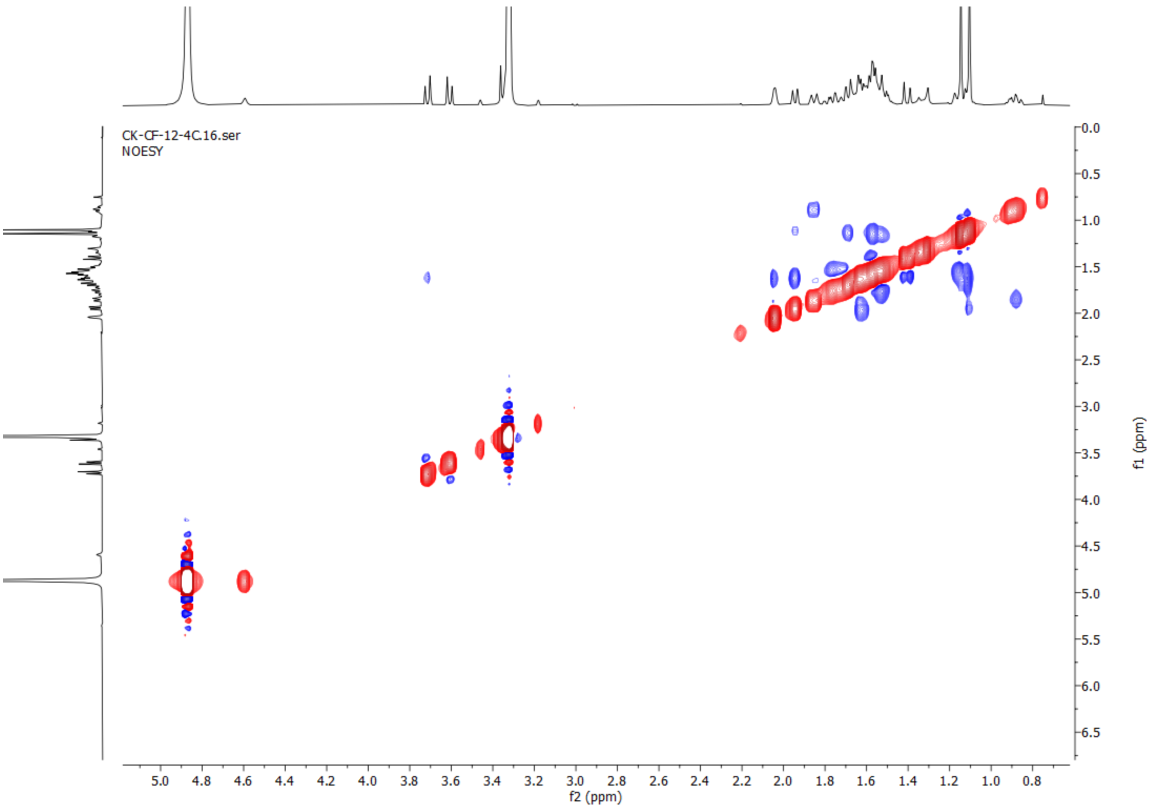

Figure S72. The NOESY spectrum of compound **9** (500 MHz, CD_3OD)

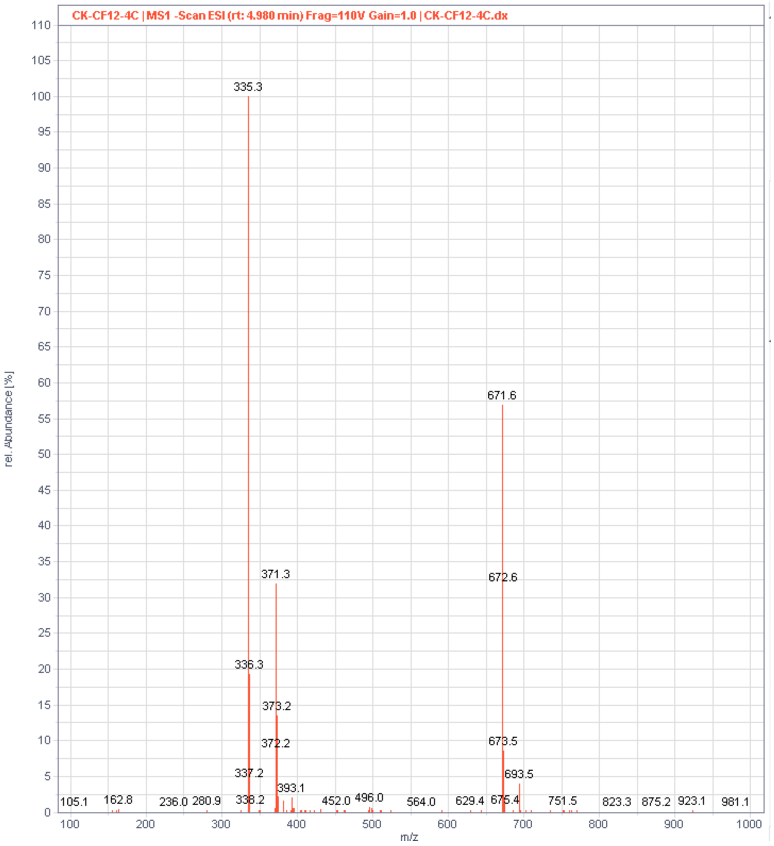

Figure S73. The LC-MS of Compound **9**

11. Spectroscopic data for compound 10

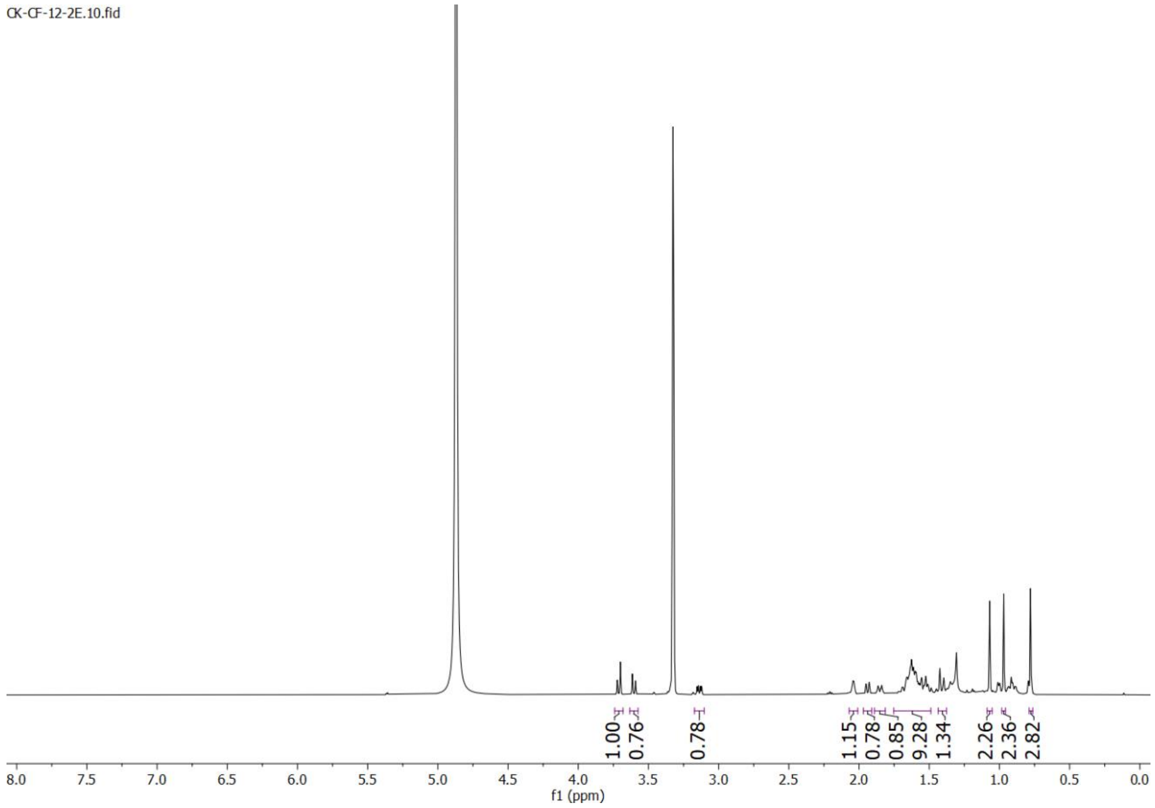

Figure S74. The ^{1}H NMR spectrum of compound **10** (500 MHz, CD_3OD)

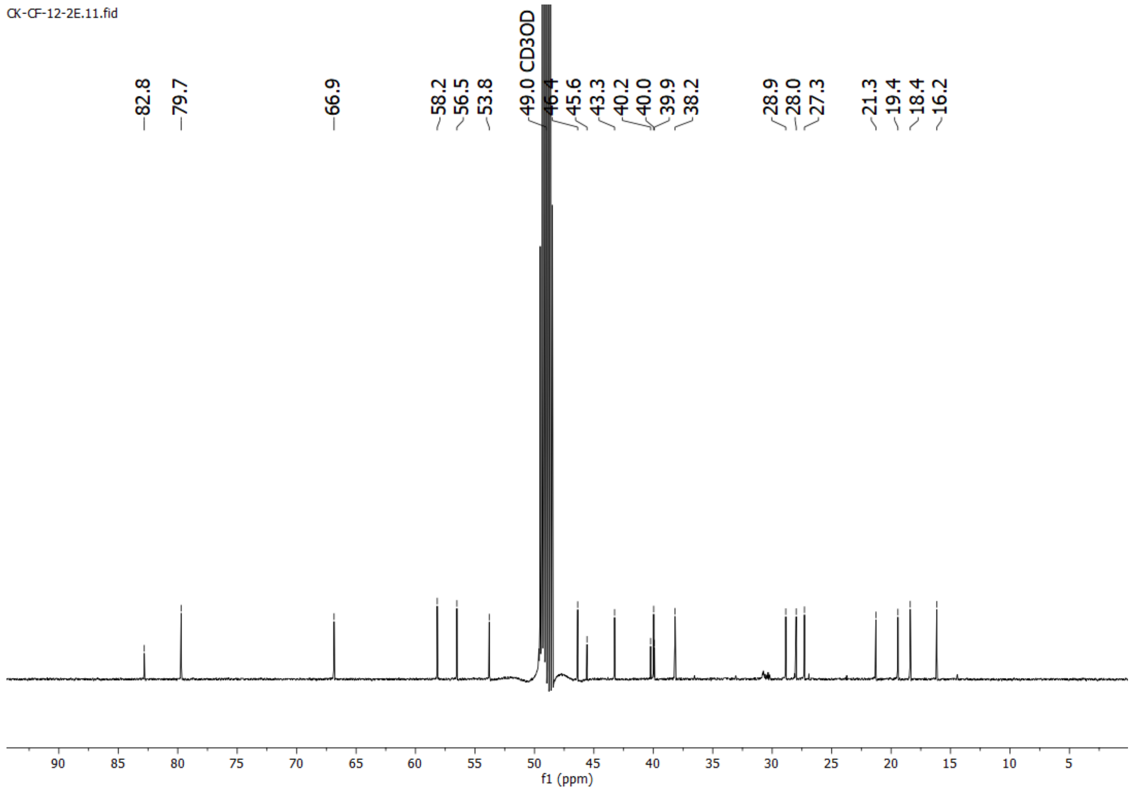

Figure **S75**. The ^{13}C NMR spectrum of compound **10** (125 MHz, CD_3OD)

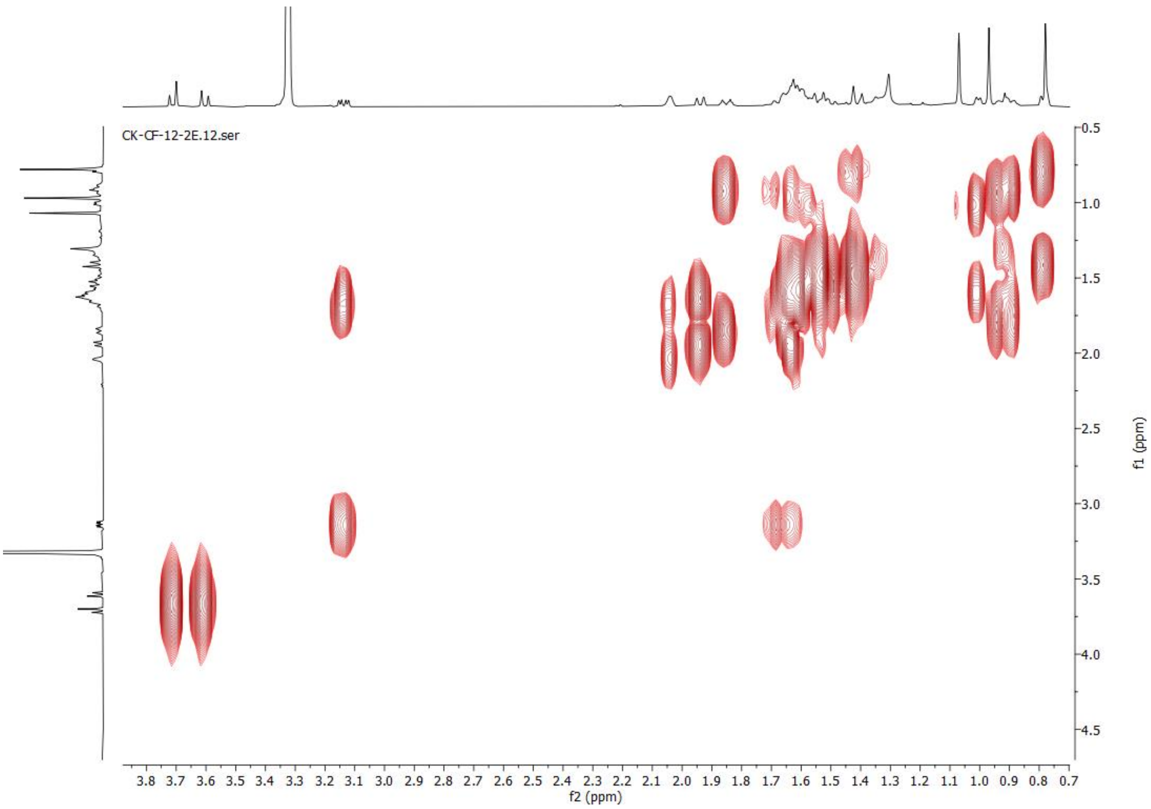

Figure S76. The COSY spectrum of compound **10** (500 MHz, CD_3OD)

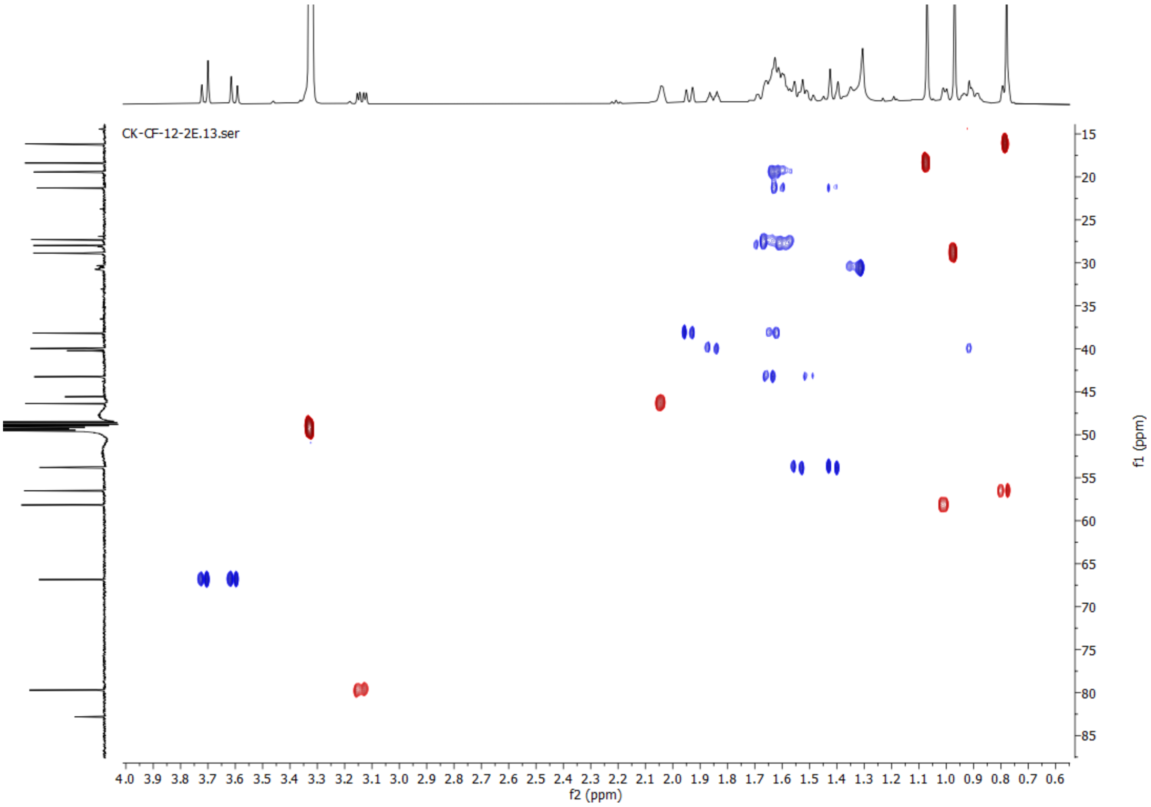

Figure S77.The HSQC spectrum of compound **10** (500/125 MHz, CD_3OD)

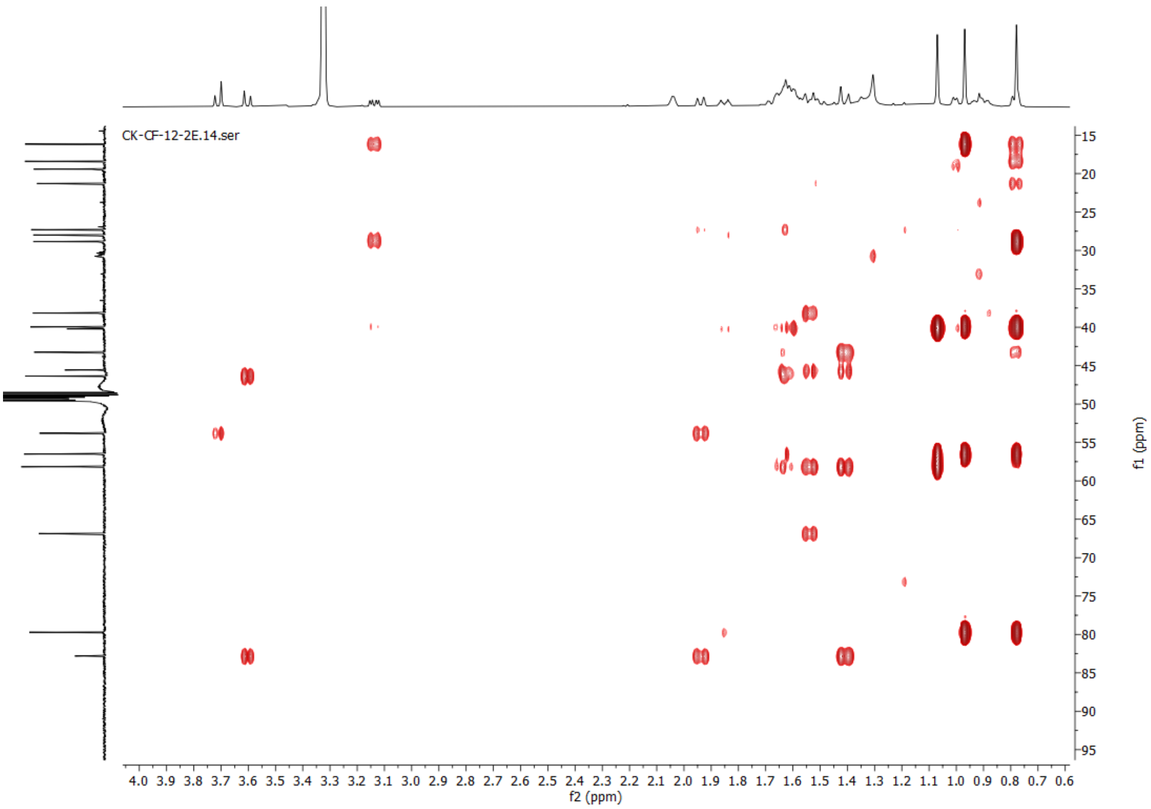

Figure S78. The HMBC spectrum of compound **10** (500/125 MHz, CD_3OD)

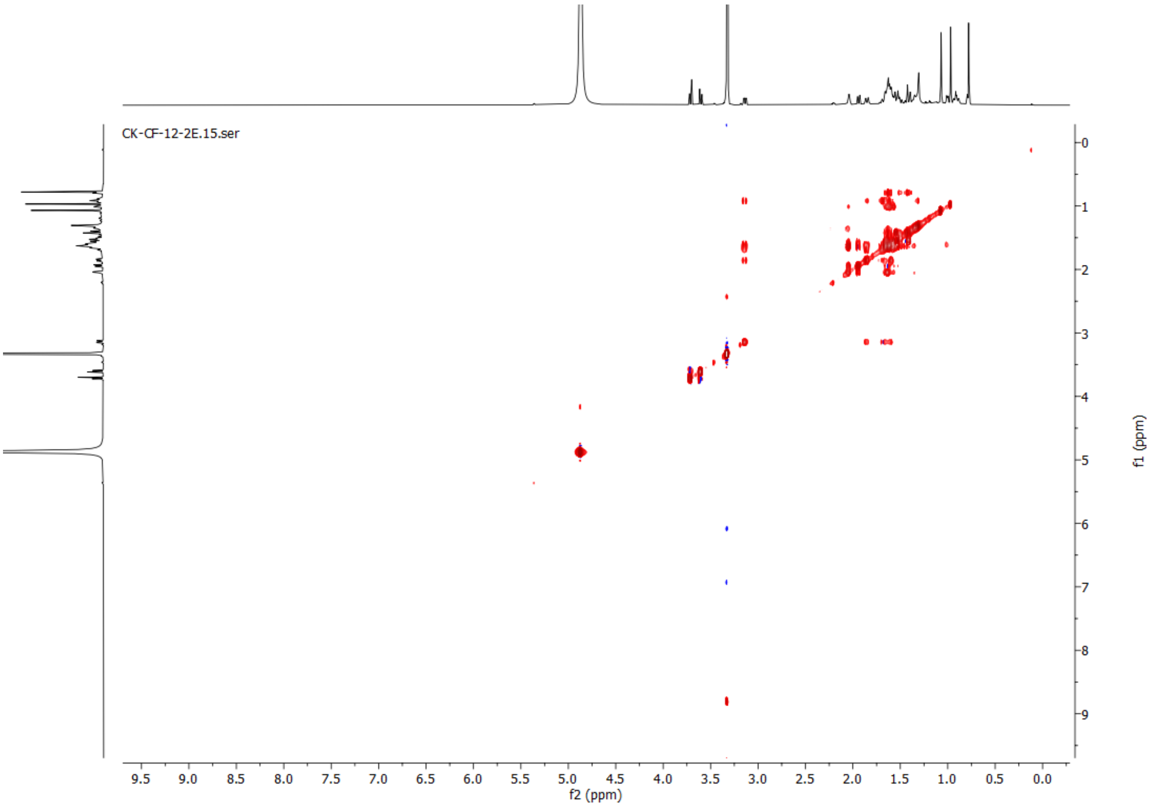

Figure S79. The TOCSY spectrum of compound **10** (500 MHz, CD_3OD)

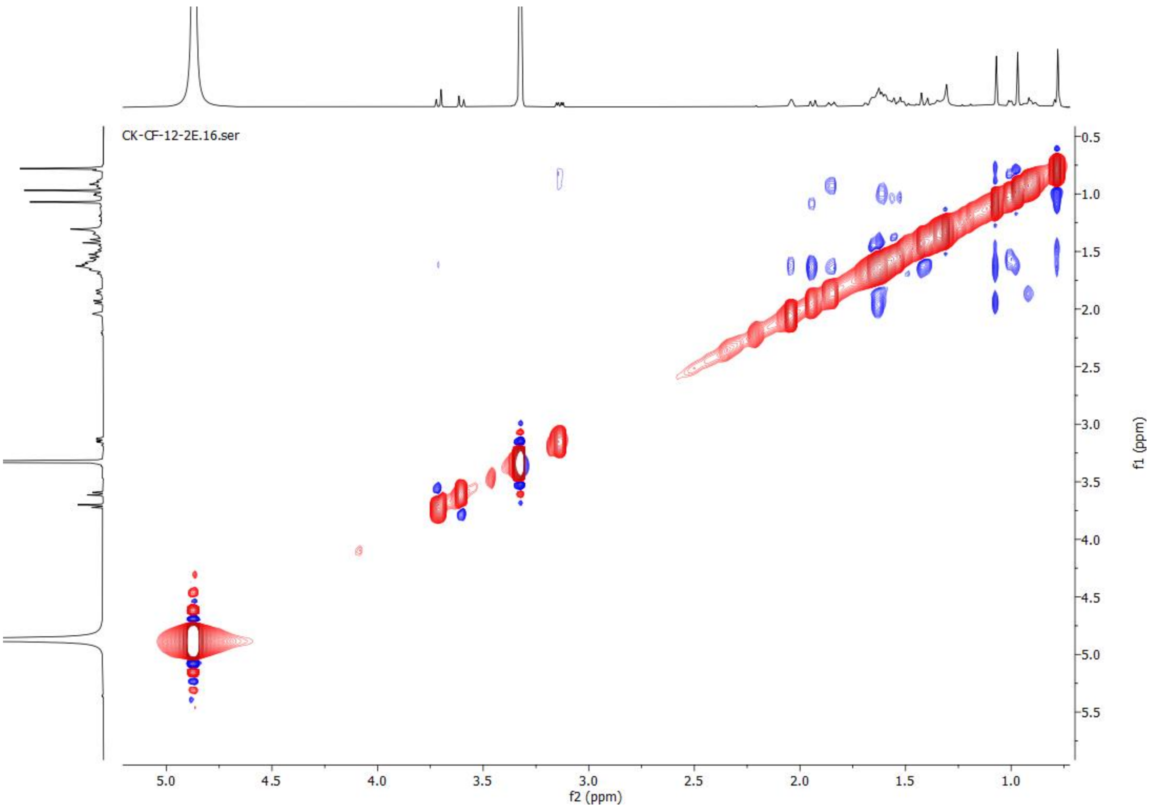

Figure S80. The NOESY spectrum of compound **10** (500 MHz, CD_3OD)

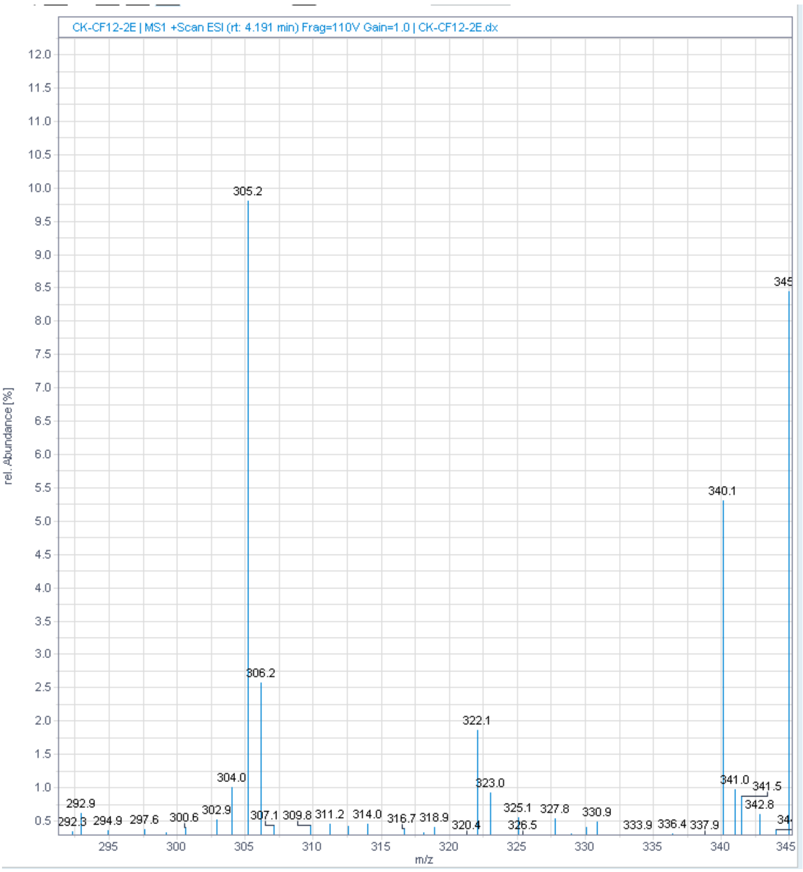

Figure S10. The LC-MS of compound **10**

12. Spectroscopic data for compound 11

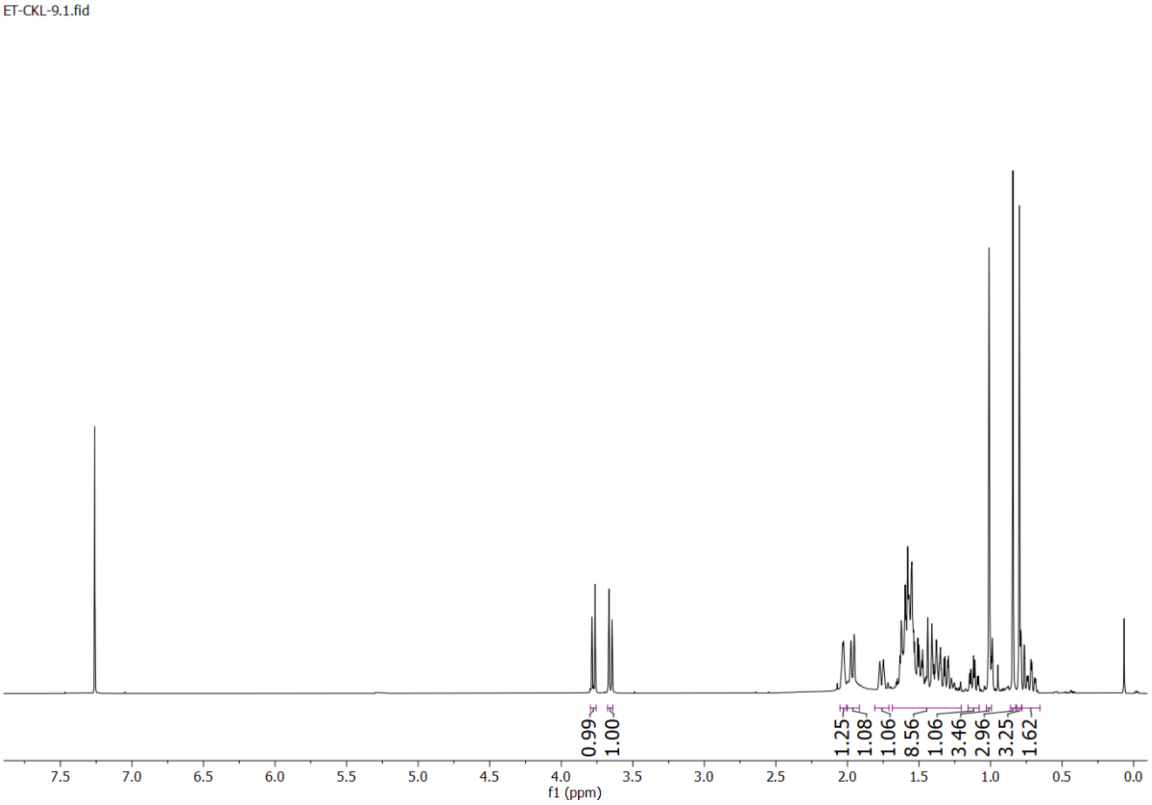

Figure S82. The ¹H NMR spectrum of compound **11** (500 MHz, CD_3OD)

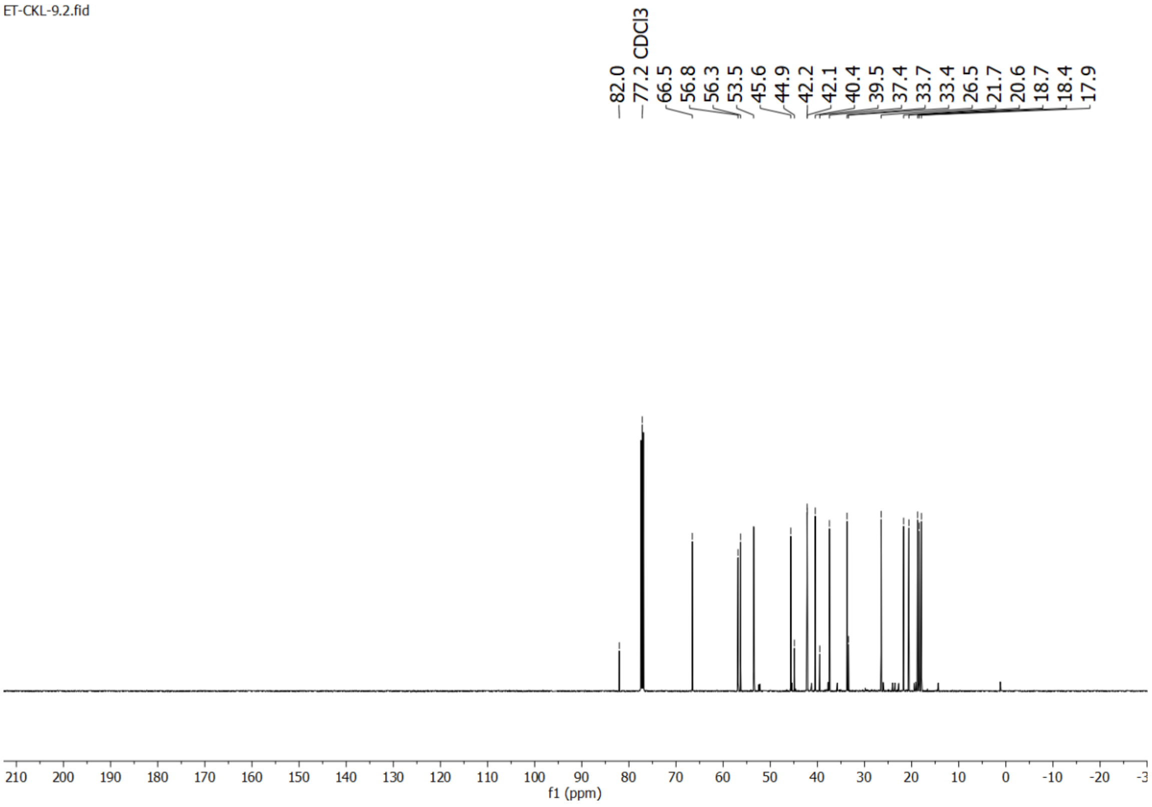

Figure S83. The ^{13}C NMR spectrum of compound **11** (125 MHz, CD_3OD)

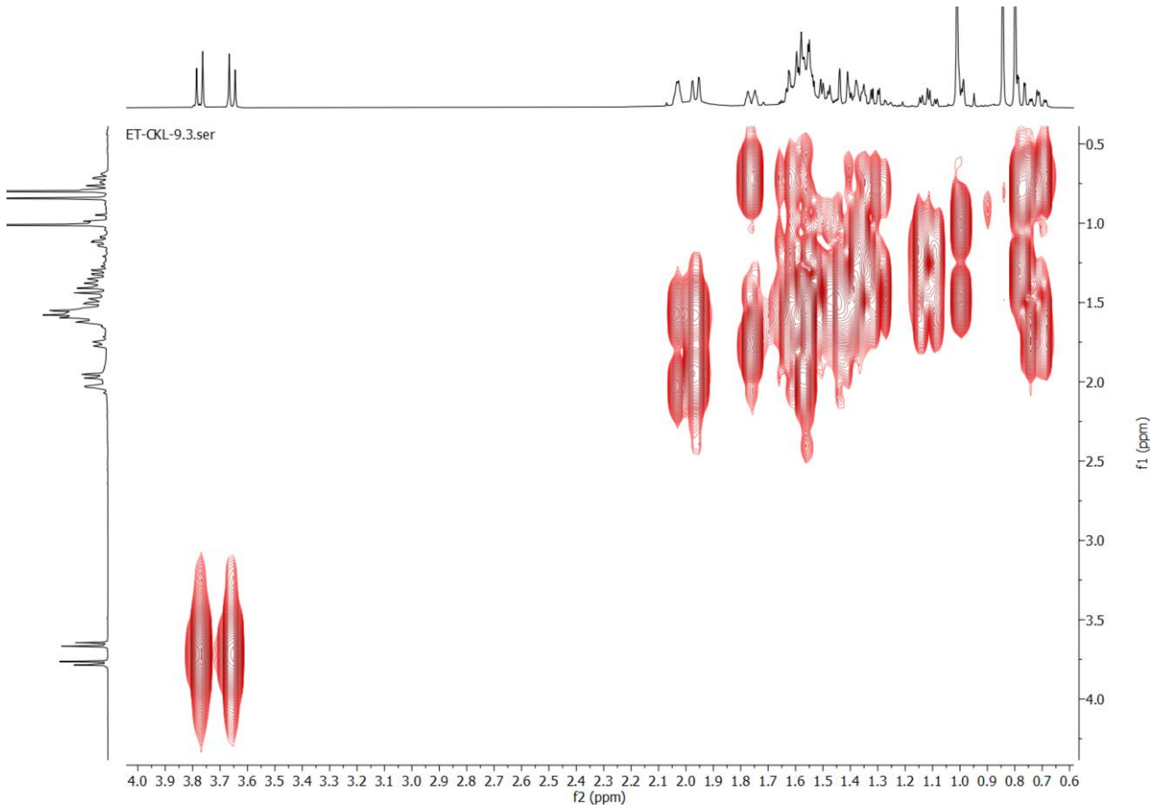

Figure S84. The COSY spectrum of compound **11** (500 MHz, CD_3OD)

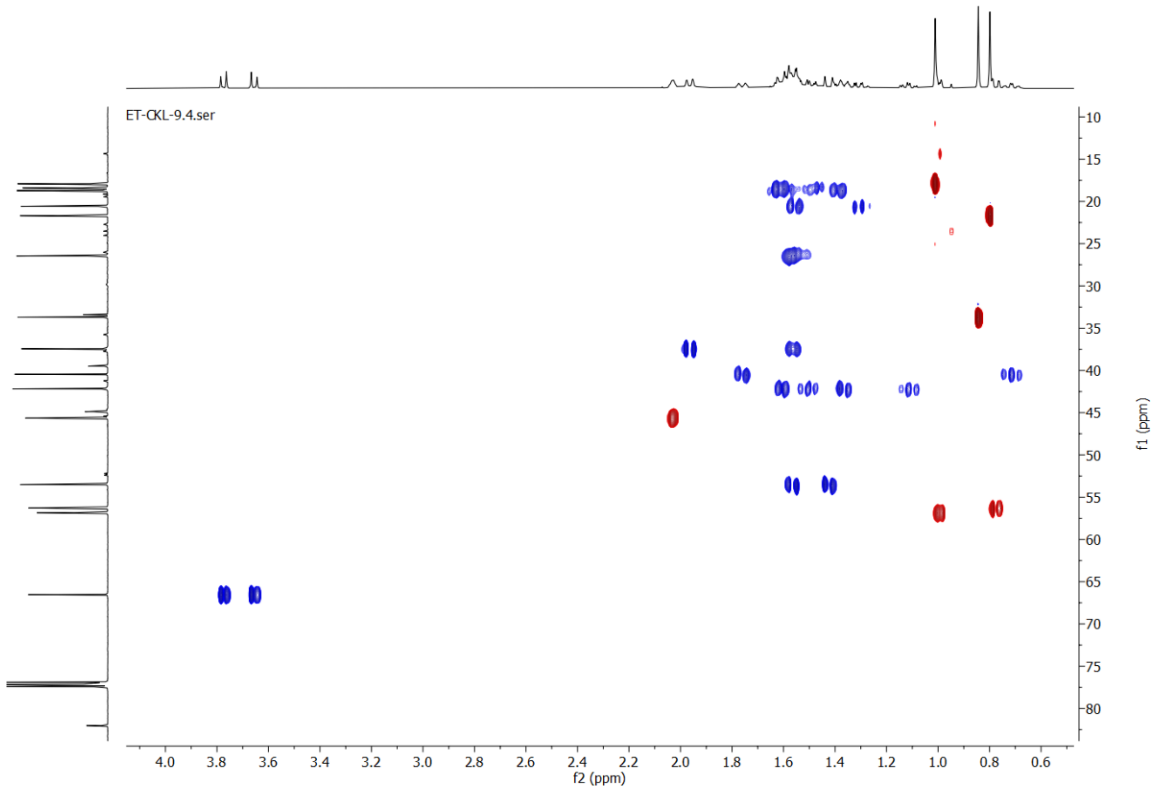

Figure S85. The HSQC spectrum of compound **11** (500/125 MHz, CD_3OD)

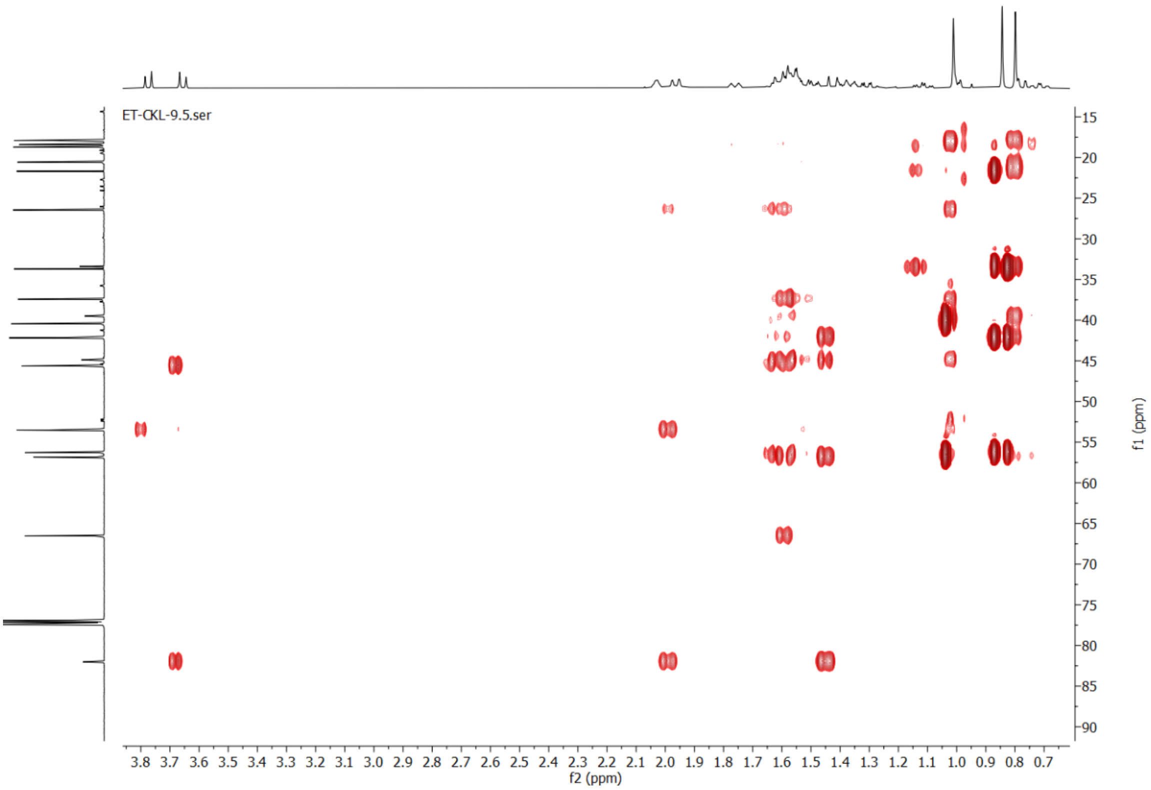

Figure S86. The HMBC spectrum of compound **11** (500/125 MHz, CD_3OD)

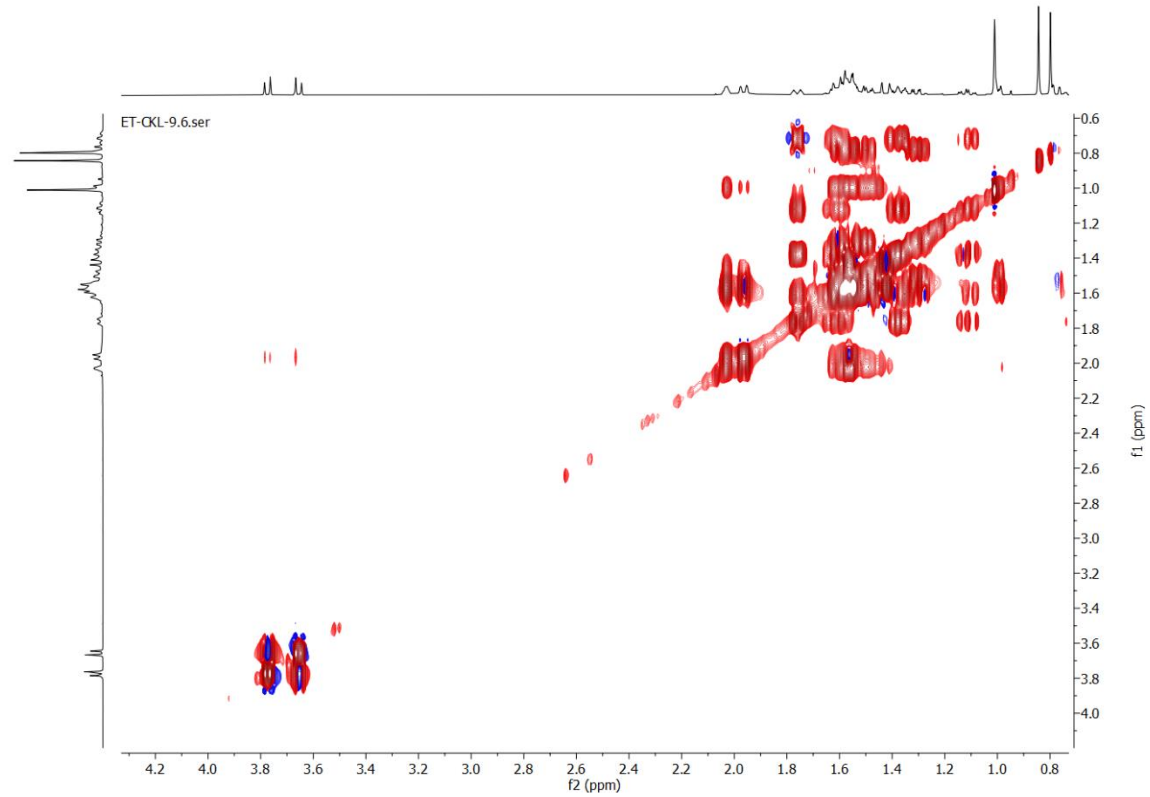

Figure S87. The TOCSY spectrum of compound **11** (500 MHz, CD_3OD)

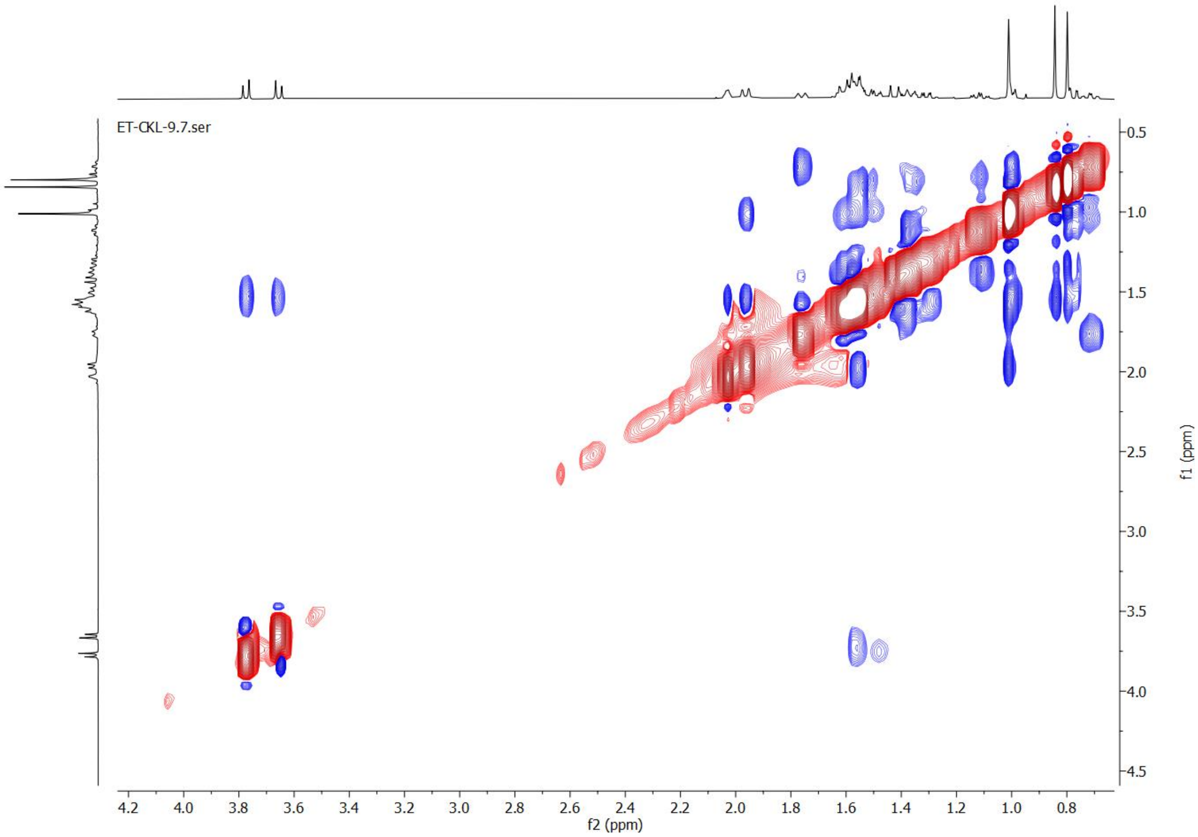

Figure S88. The NOESY spectrum of compound **11** (500 MHz, CD_3OD)

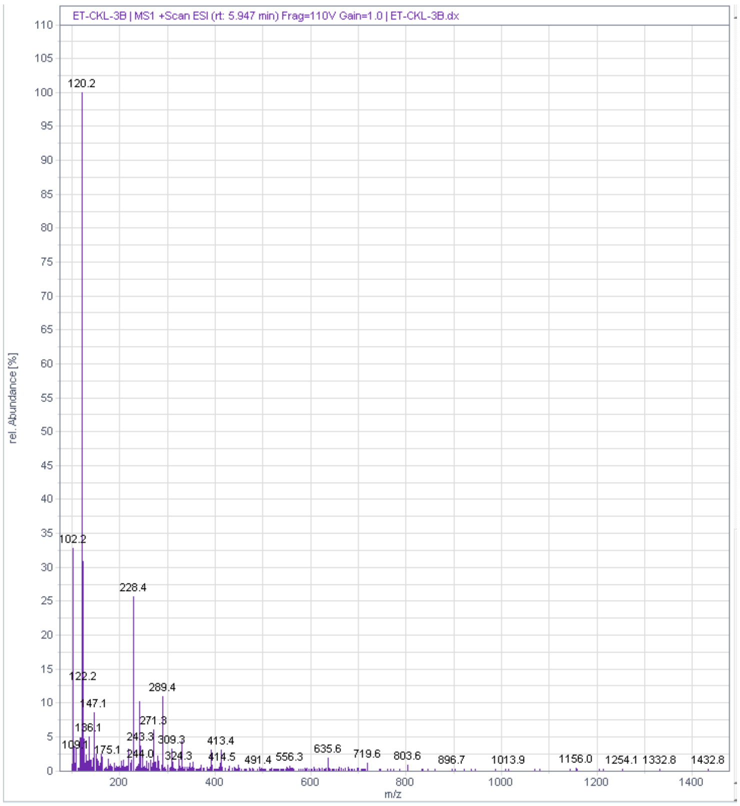

Figure S90. The LC-MS of compound **11**

13. Spectroscopic data for compound 12

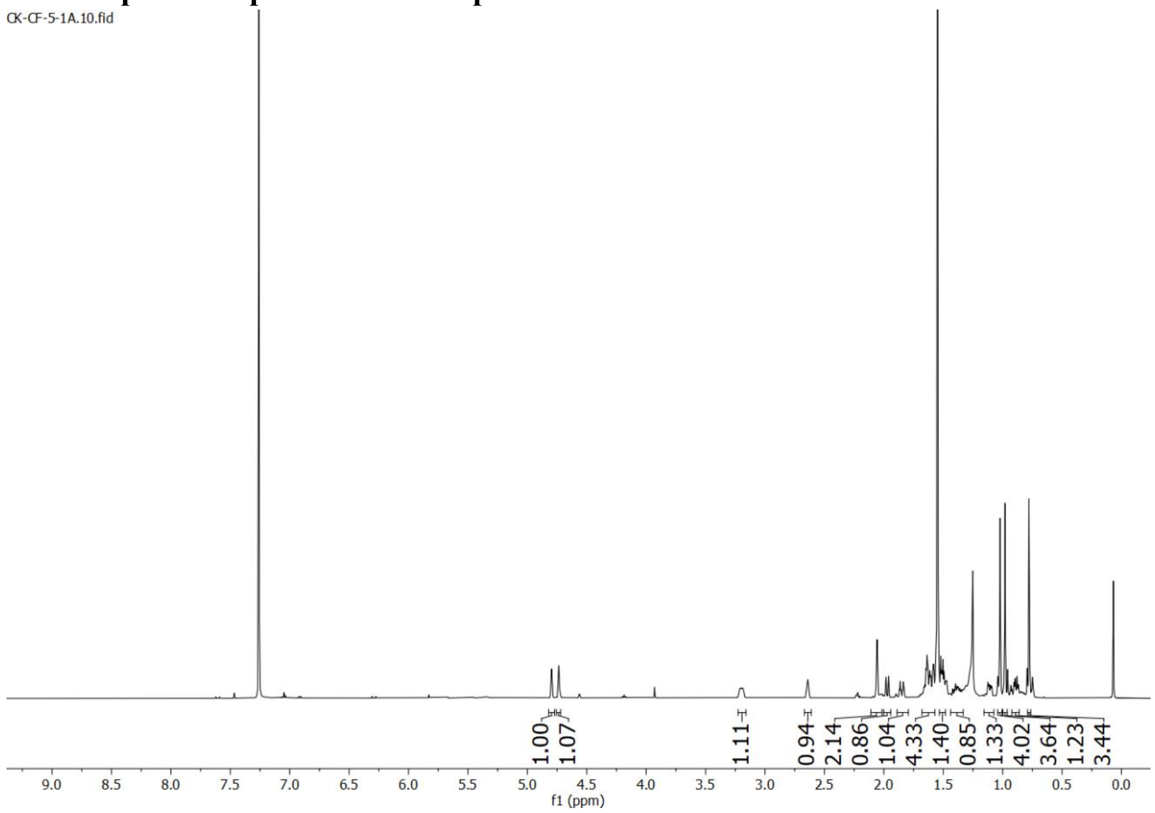

Figure S90. The ^{1}H NMR spectrum of compound **12** (500 MHz, $CDCl_3$)

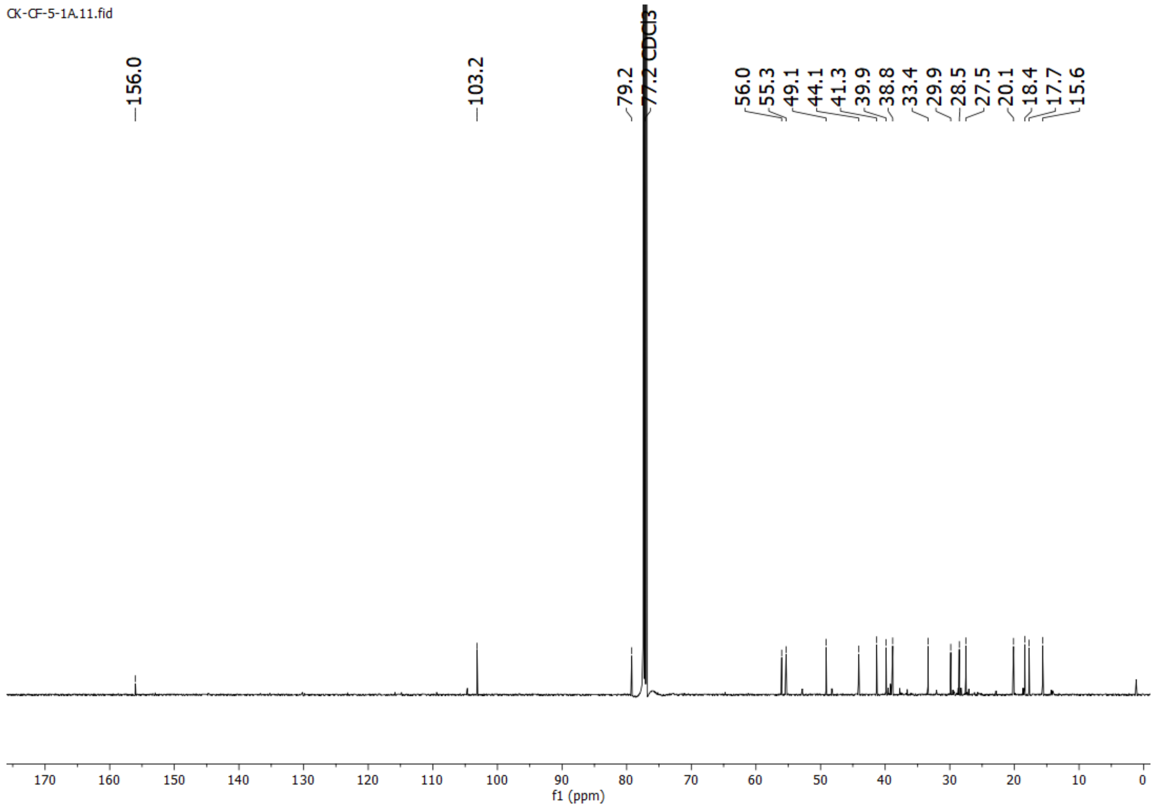

Figure S91. The ^{13}C NMR spectrum of compound **12** (125 MHz, $CDCl_3$)

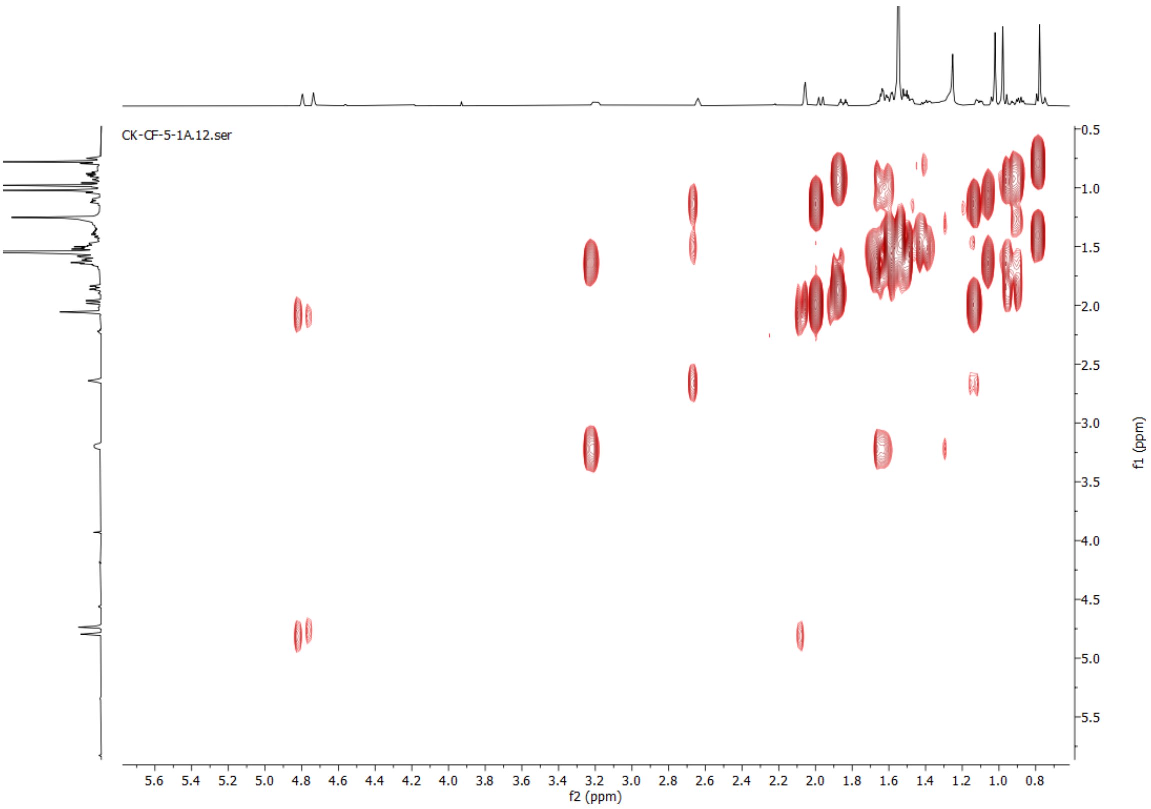

Figure S92. The COSY spectrum of compound **12** (500 MHz, $CDCl_3$)

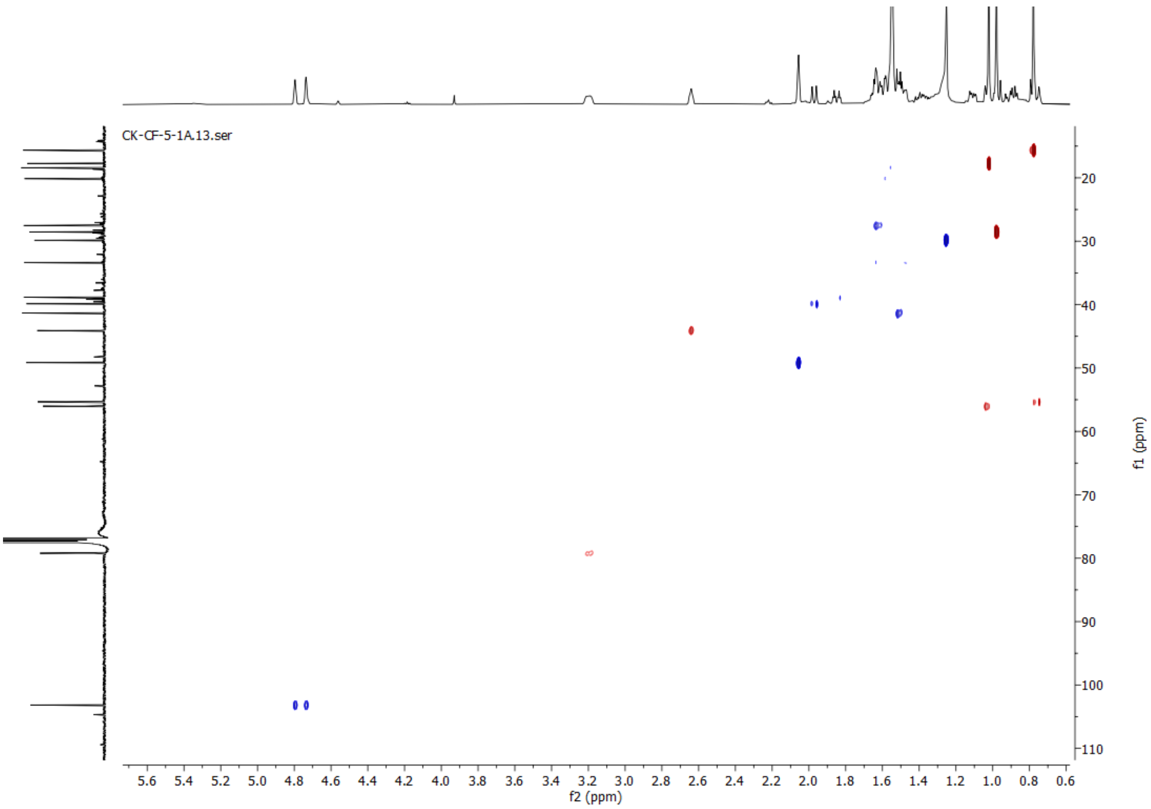

Figure S93. The HSQC spectrum of compound **12** (500 MHz, $CDCl_3$)

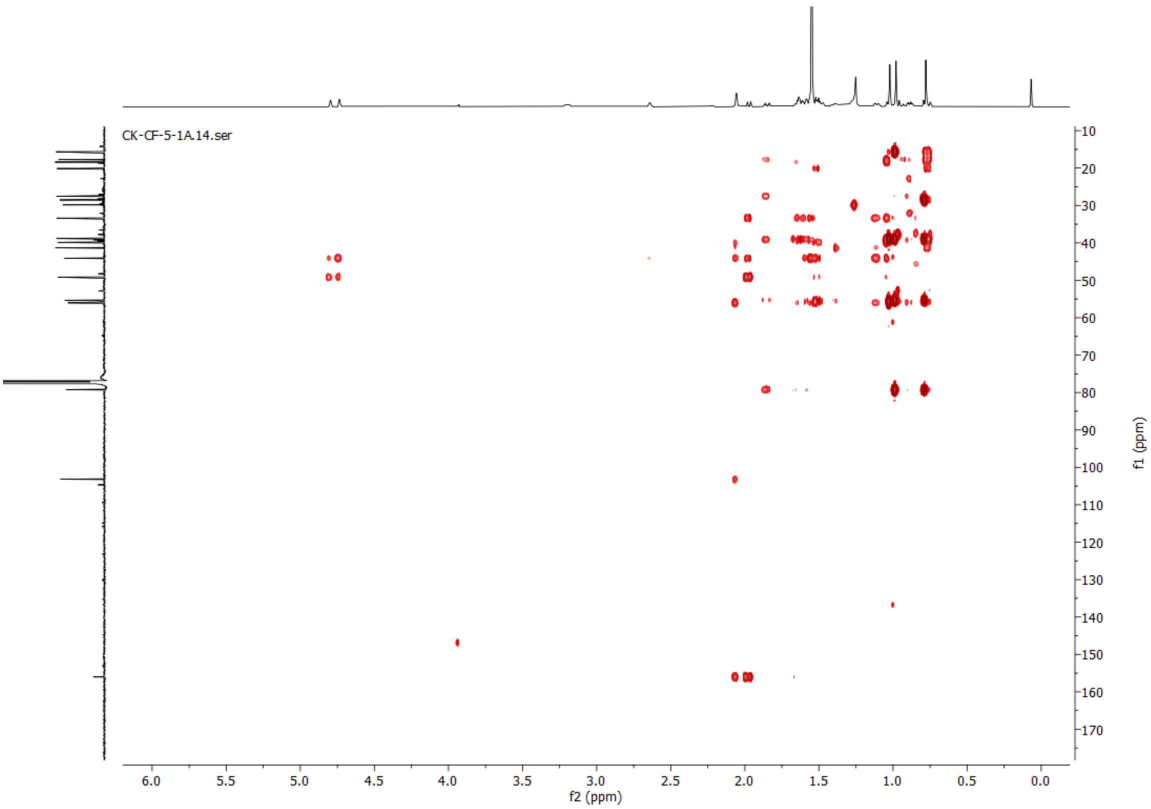

Figure S94. The HMBC spectrum of compound **12** (500/ 125 MHz, $CDCl_3$)

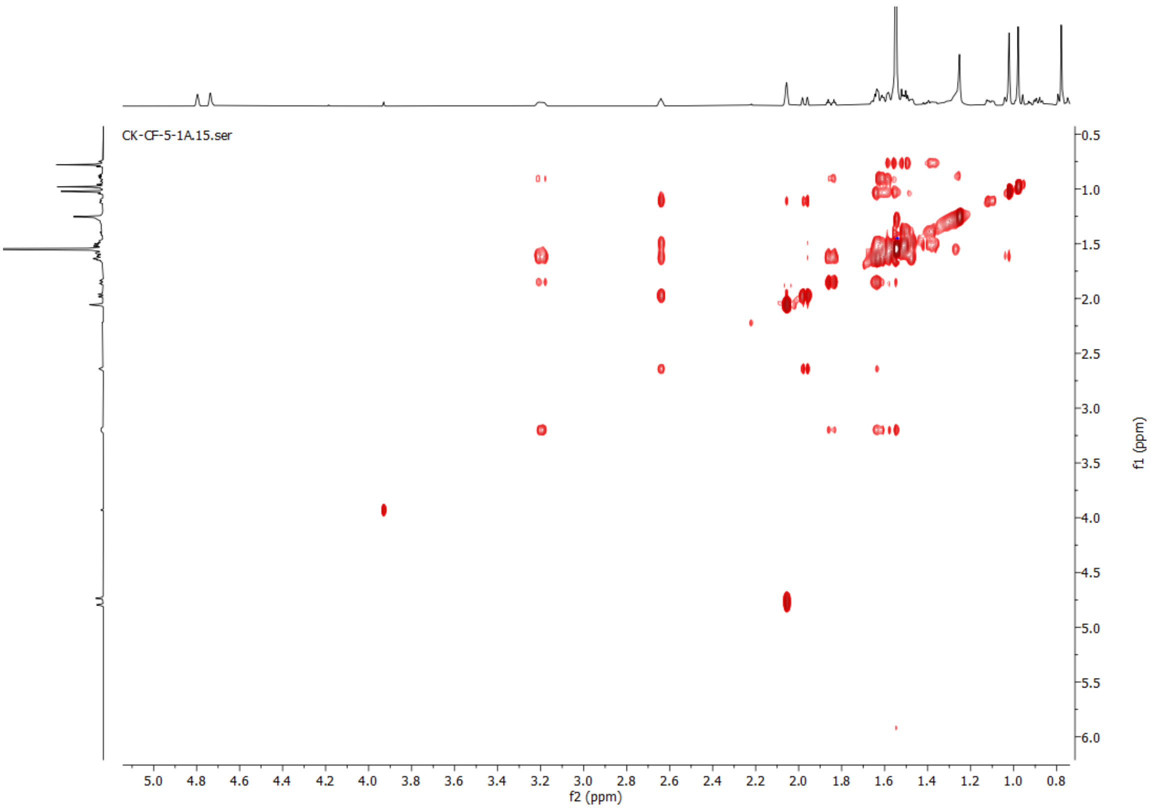

Figure S95. The TOCSY spectrum of compound **12** (500 MHz, $CDCl_3$)

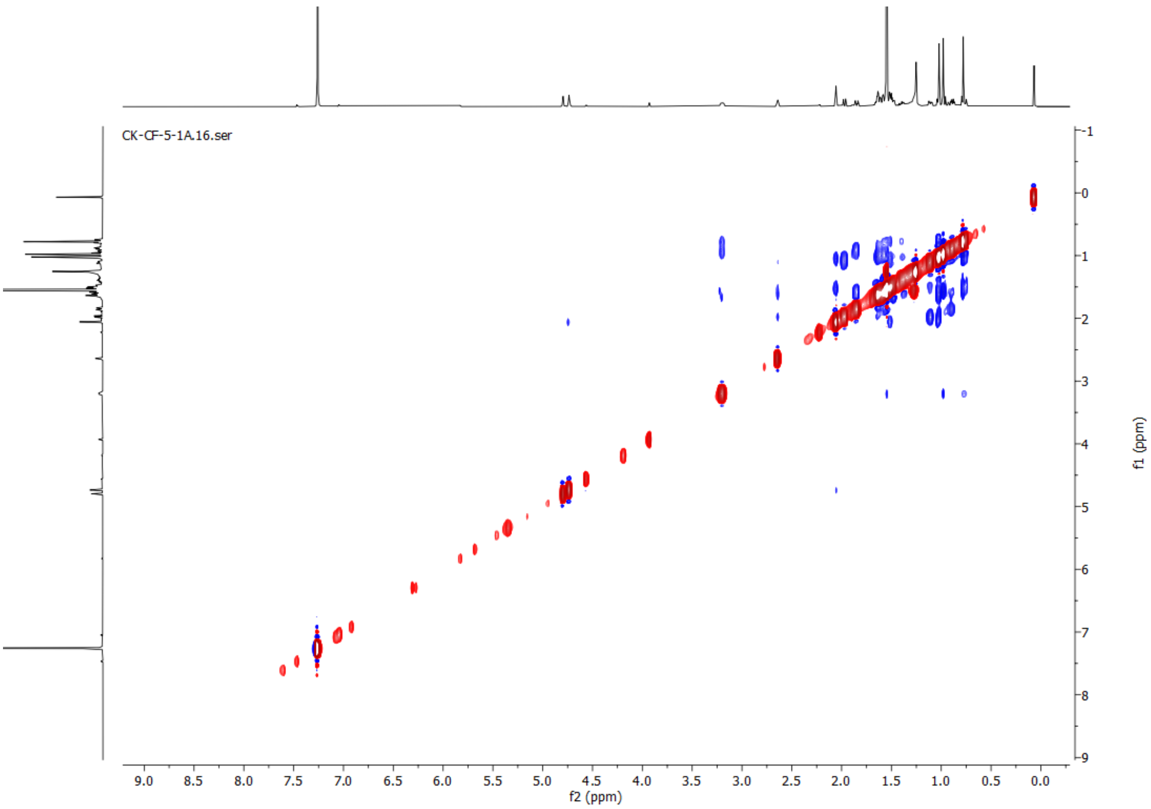

Figure S96. The NOESY spectrum of compound **12** (500 MHz, $CDCl_3$)

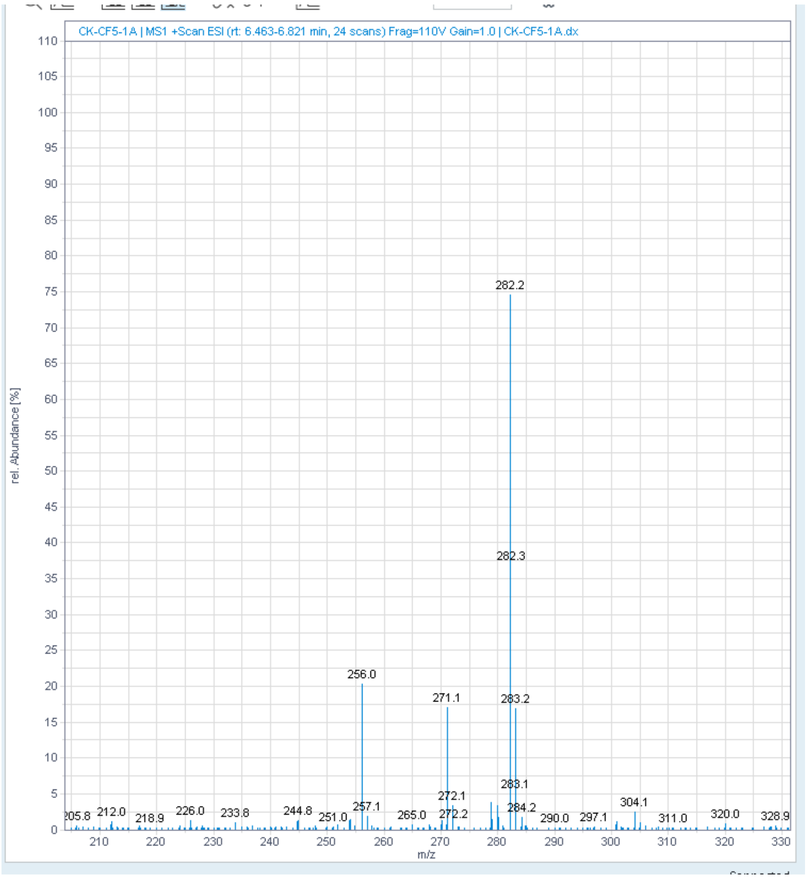

Figure S97. The LC-MS of compound **12**

14. Spectroscopic data for compound 13

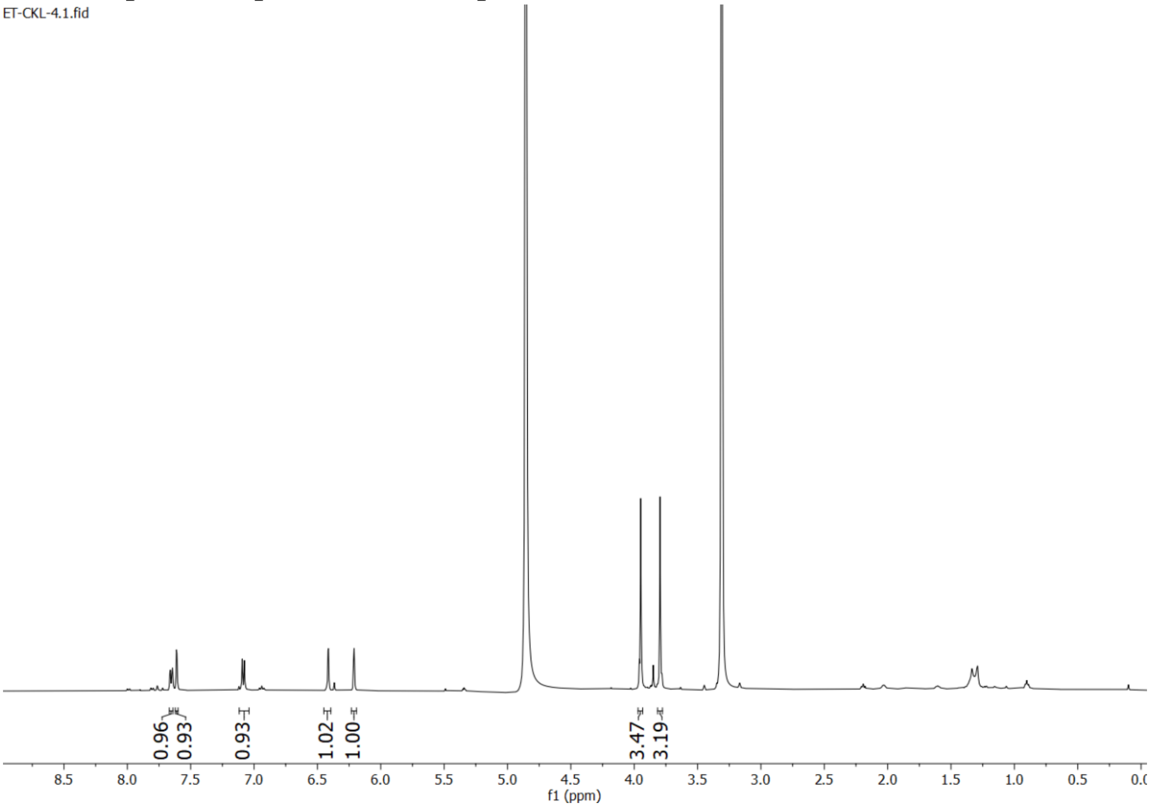

Figure S98. The ^{1}H NMR spectrum of compound **13** (500 MHz, CD_3OD)

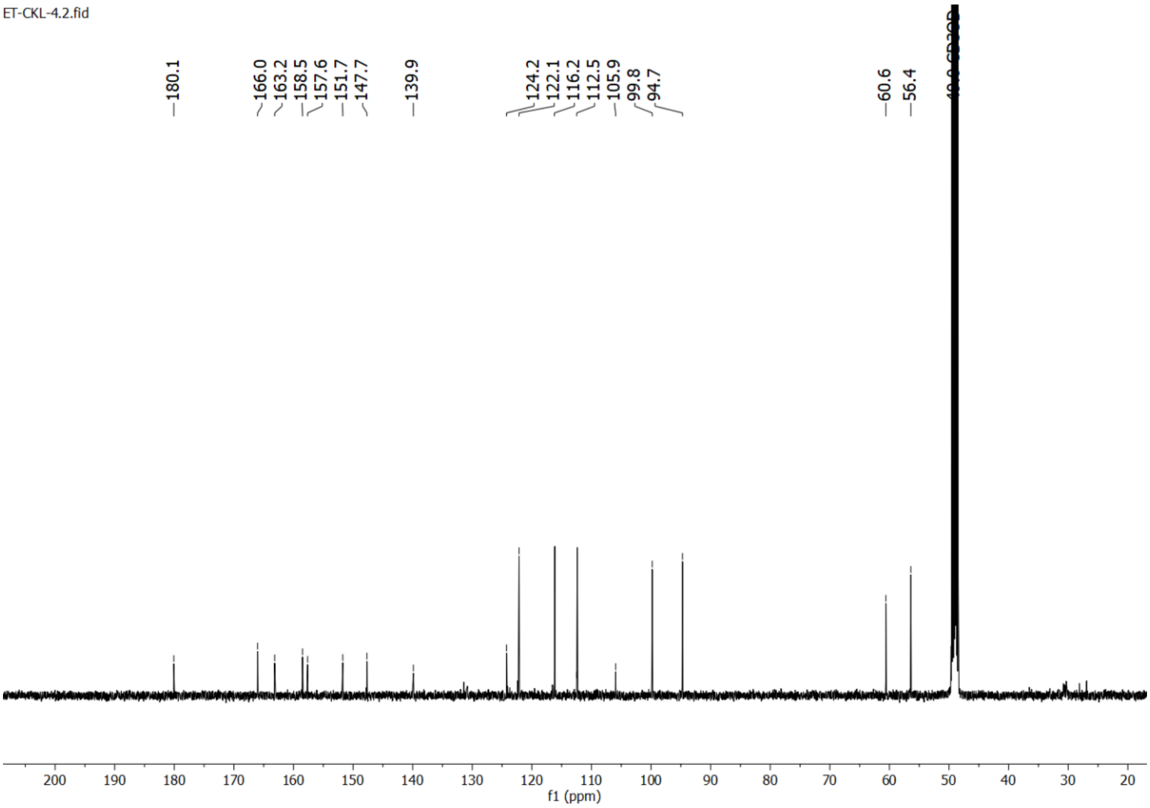

Figure S99. The ^{13}C NMR spectrum of compound **13** (125 MHz, CD_3OD)

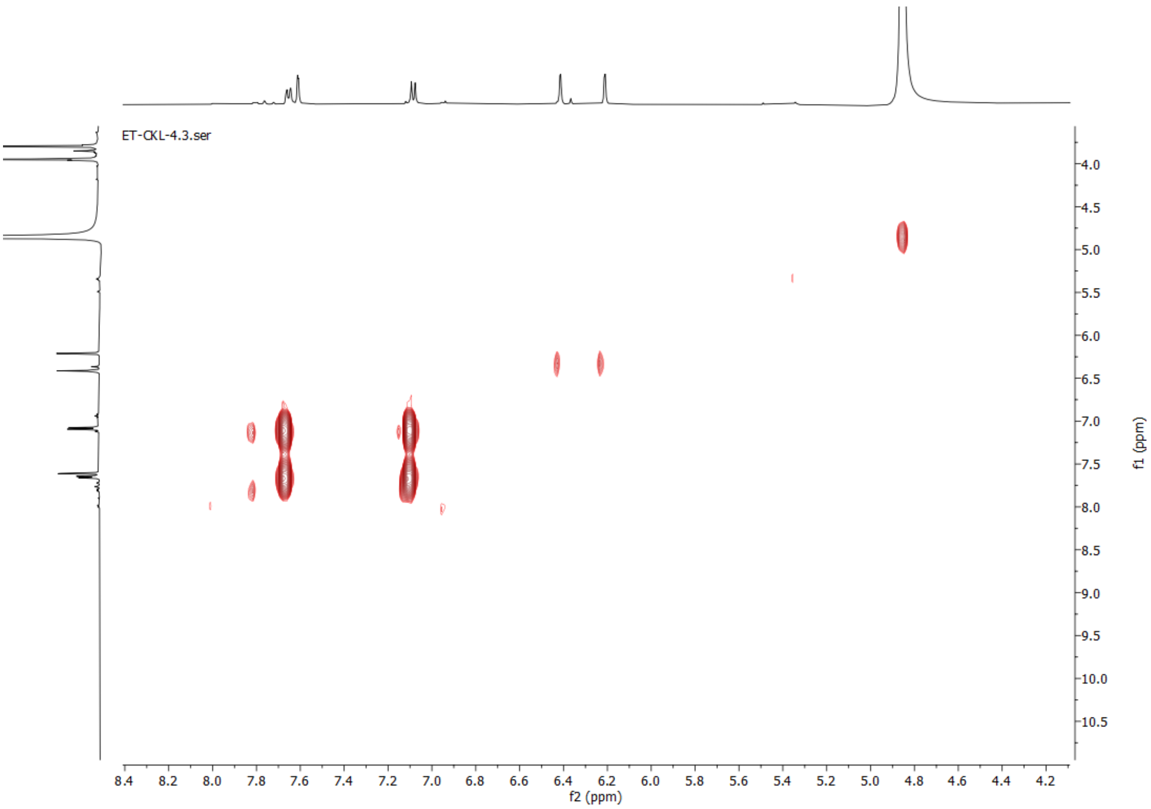

Figure S100. The COSY spectrum of compound **13** (500 MHz, CD_3OD)

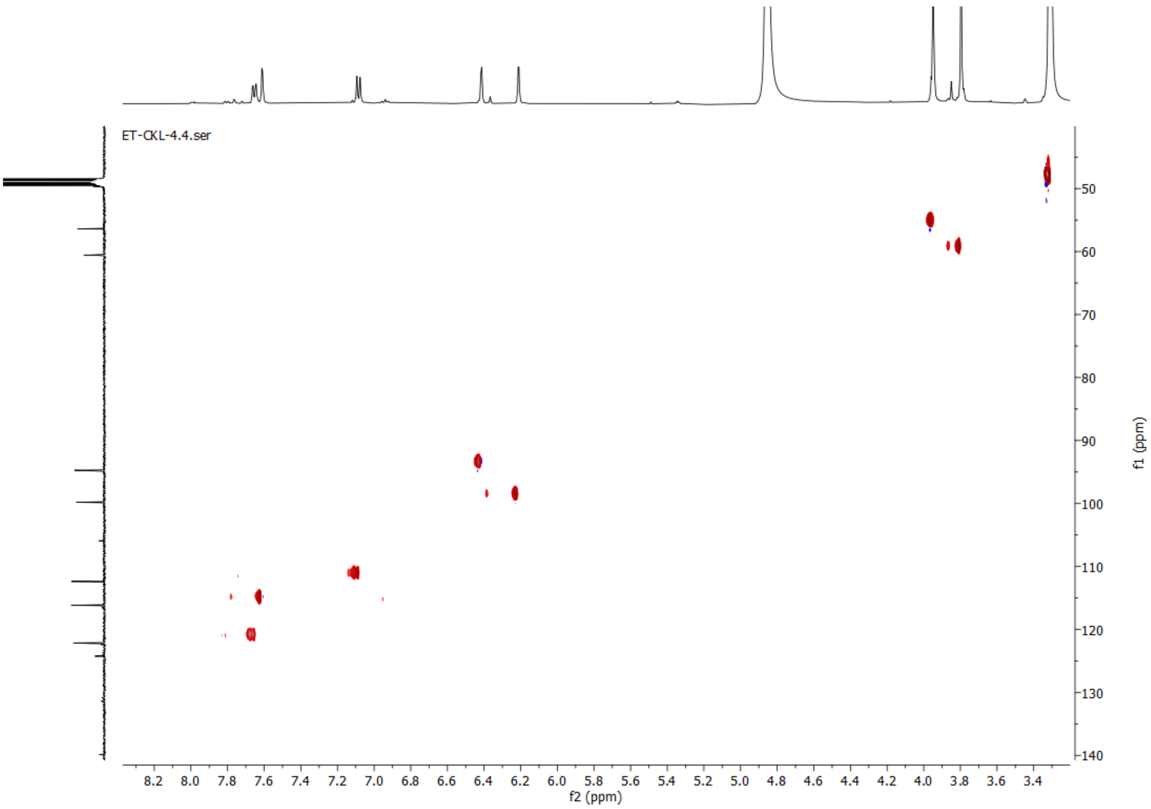

Figure S101. The HSQC spectrum of compound **13** (500 MHz/125 MHz, CD_3OD)

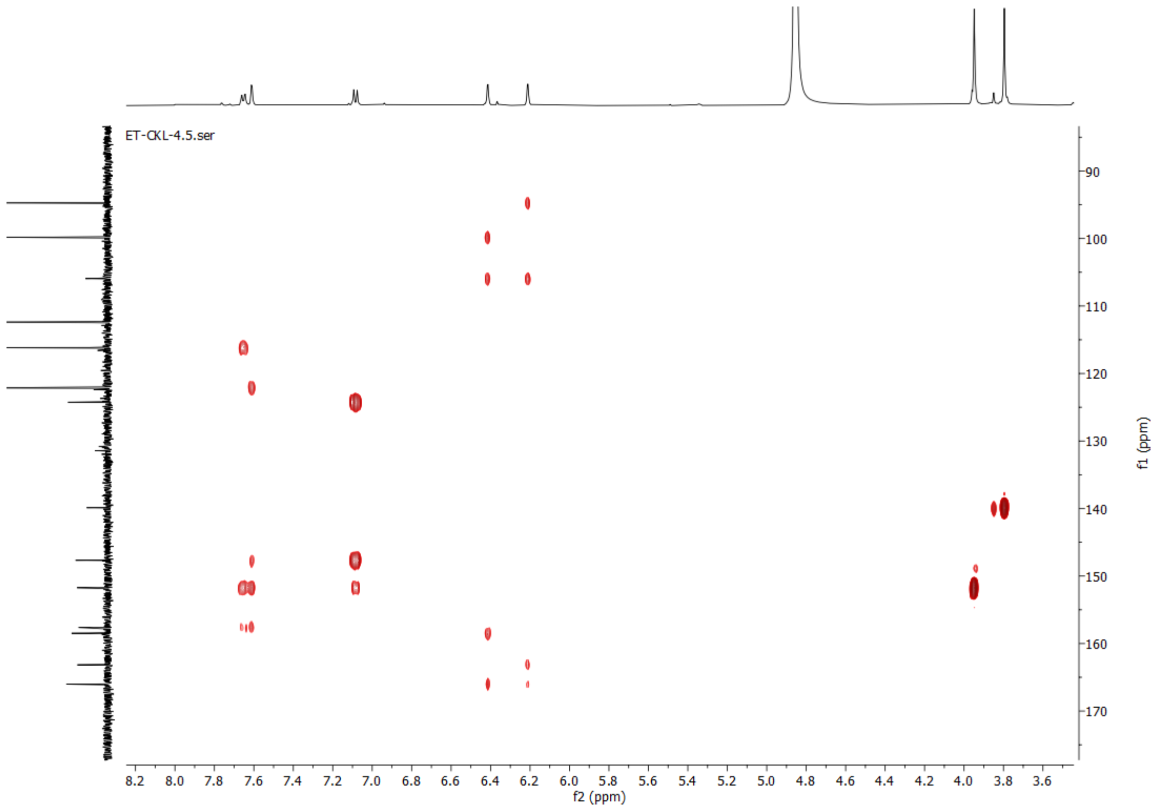

Figure S102. The HMBC spectrum of compound **13** (500/125 MHz, CD_3OD)

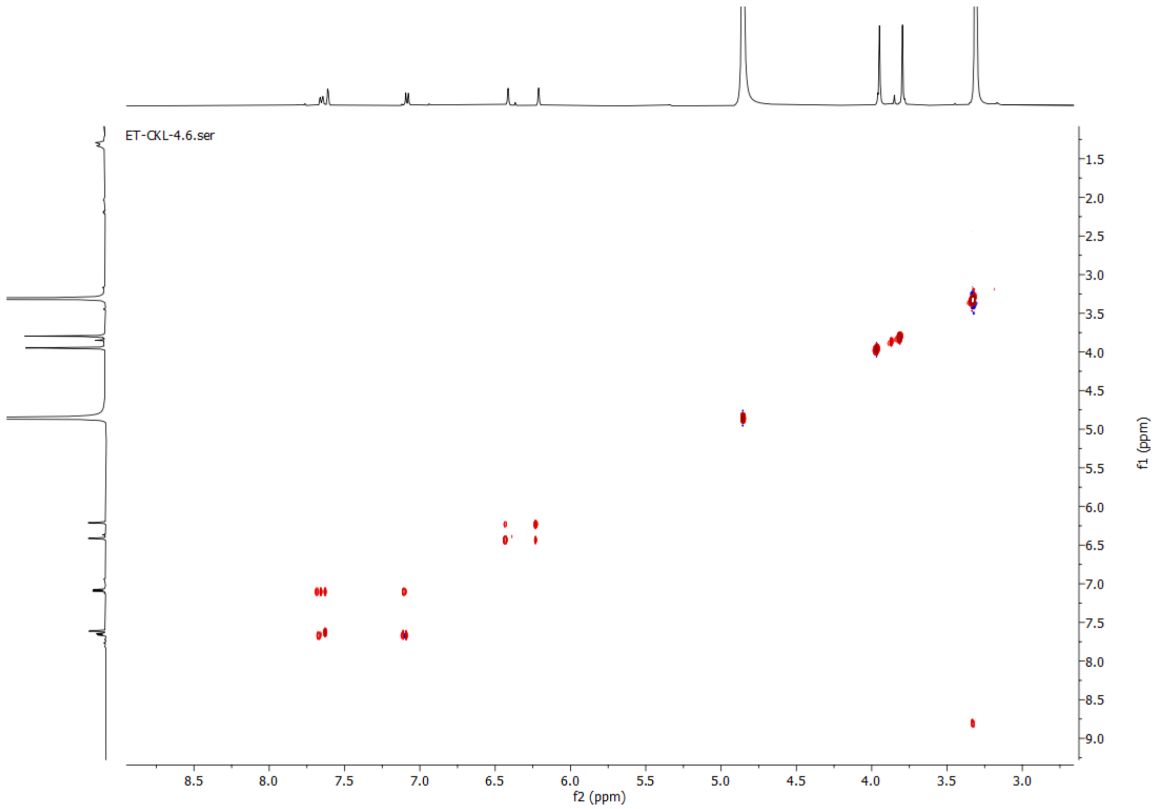

Figure S103. The TOCSY spectrum of compound **13** (500 MHz, CD_3OD)

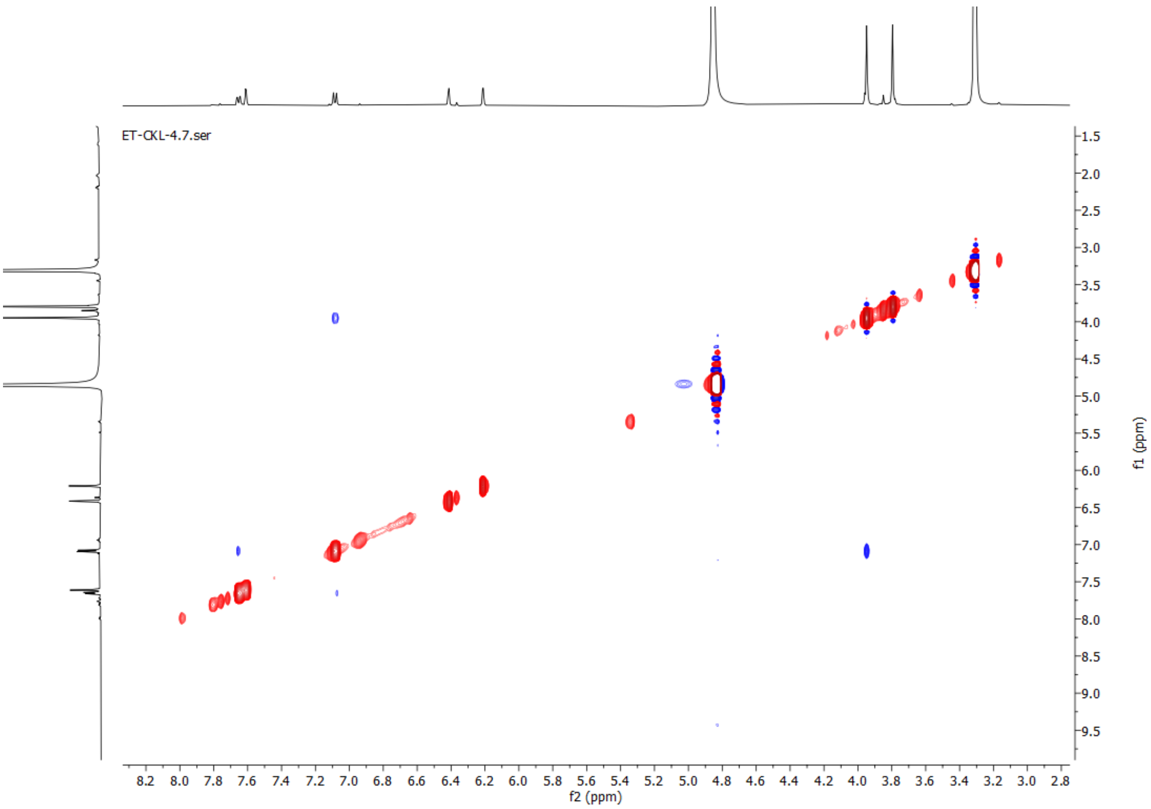

Figure S104. The NOESY spectrum of compound **13** (500 MHz, CD_3OD)

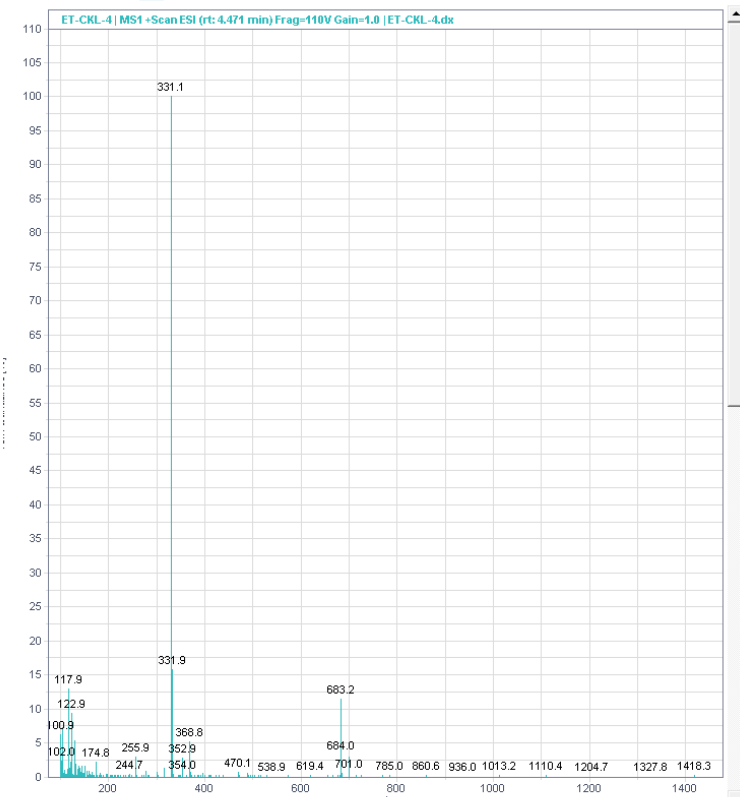

Figure S105. The LC-MS of compound **13**

15. Spectroscopic data for compound 14

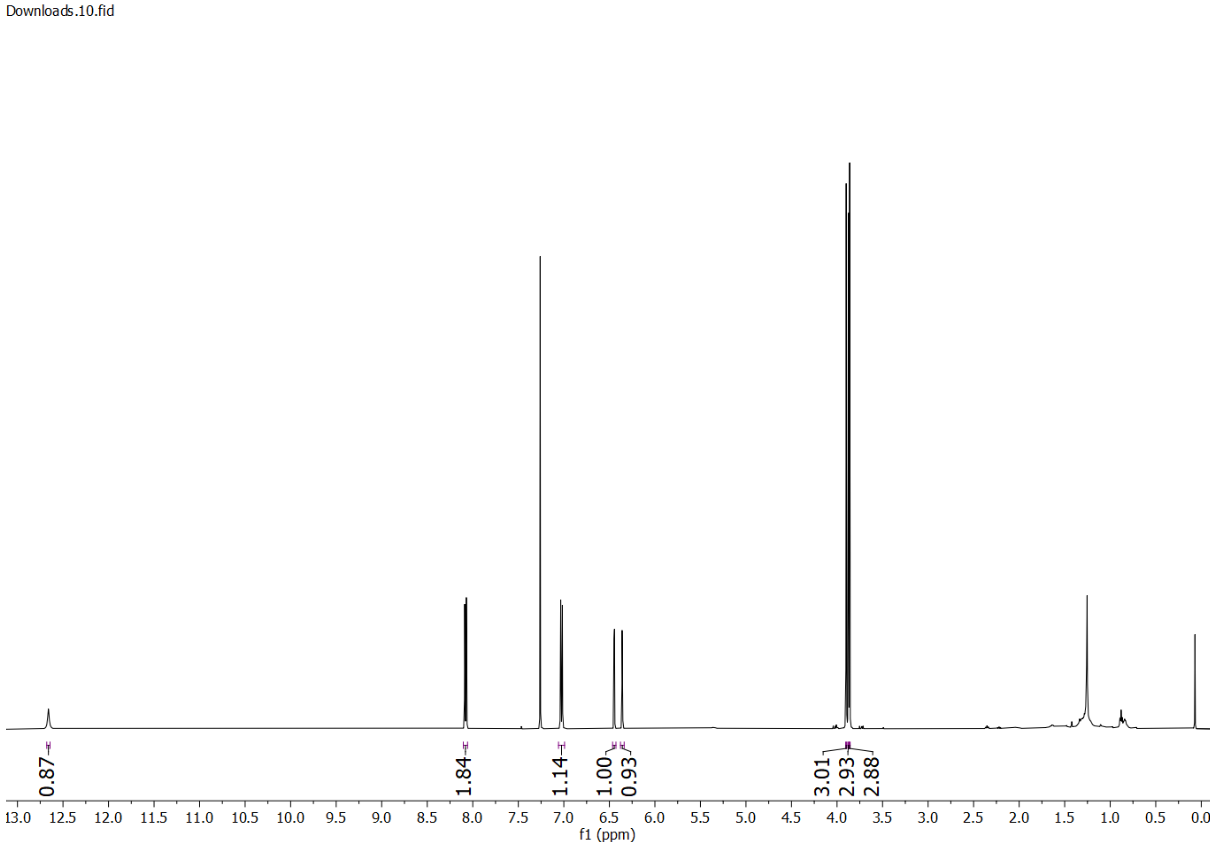

Figure S106. The ^{1}H NMR spectrum of compound **14** (500 MHz, $CDCl_3$)

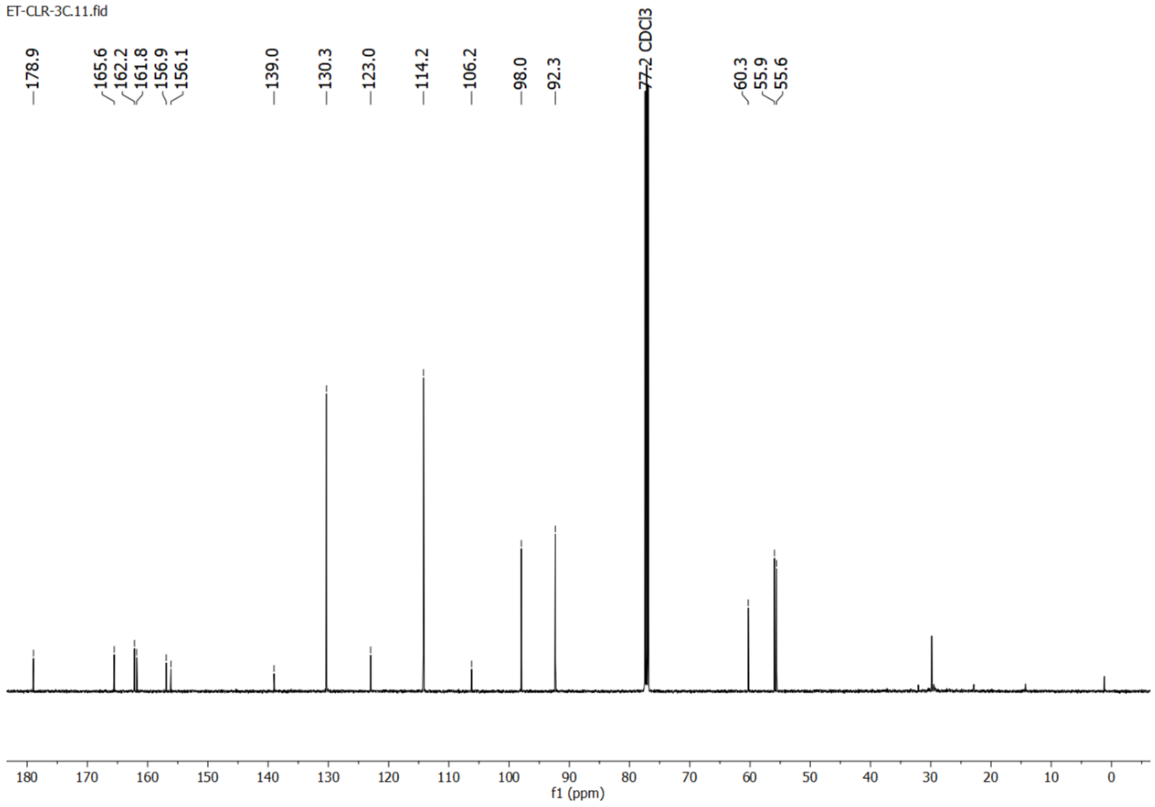

Figure S107. The ^{13}C NMR spectrum of compound **14** (125 MHz, $CDCl_3$)

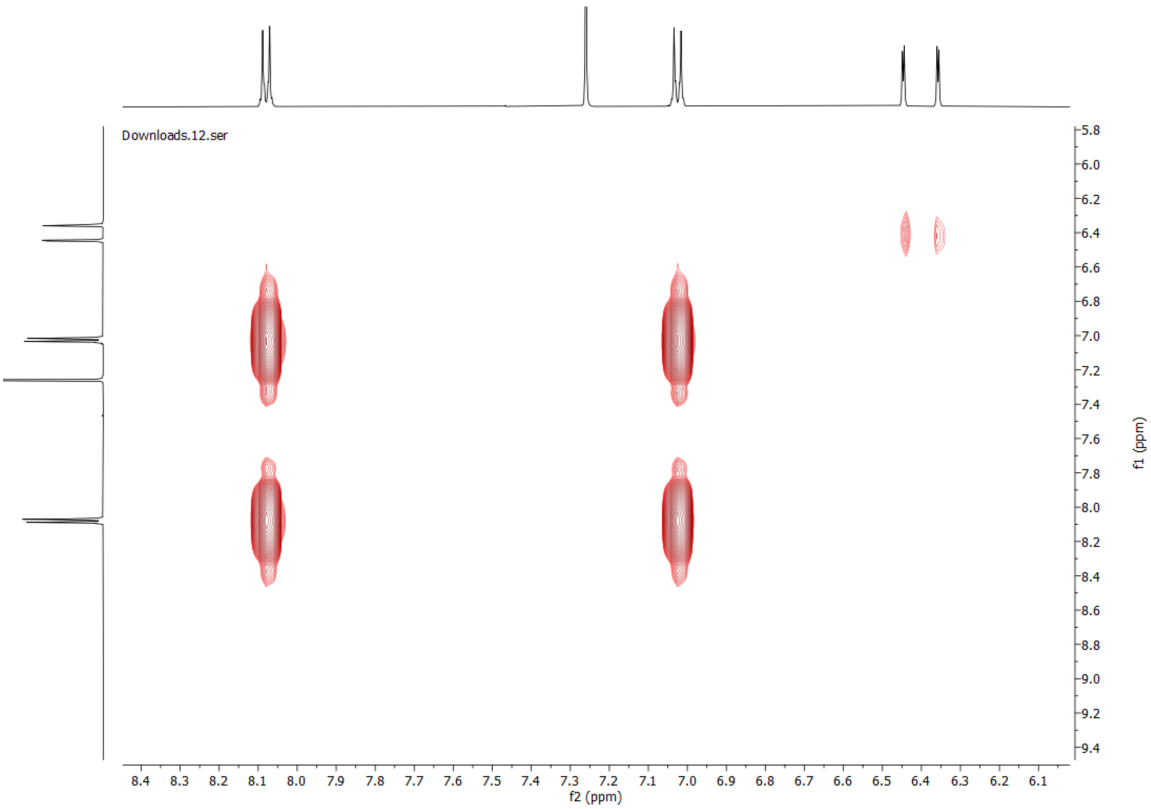

Figure S108. The COSY spectrum of compound **14** (500 MHz, $CDCl_3$)

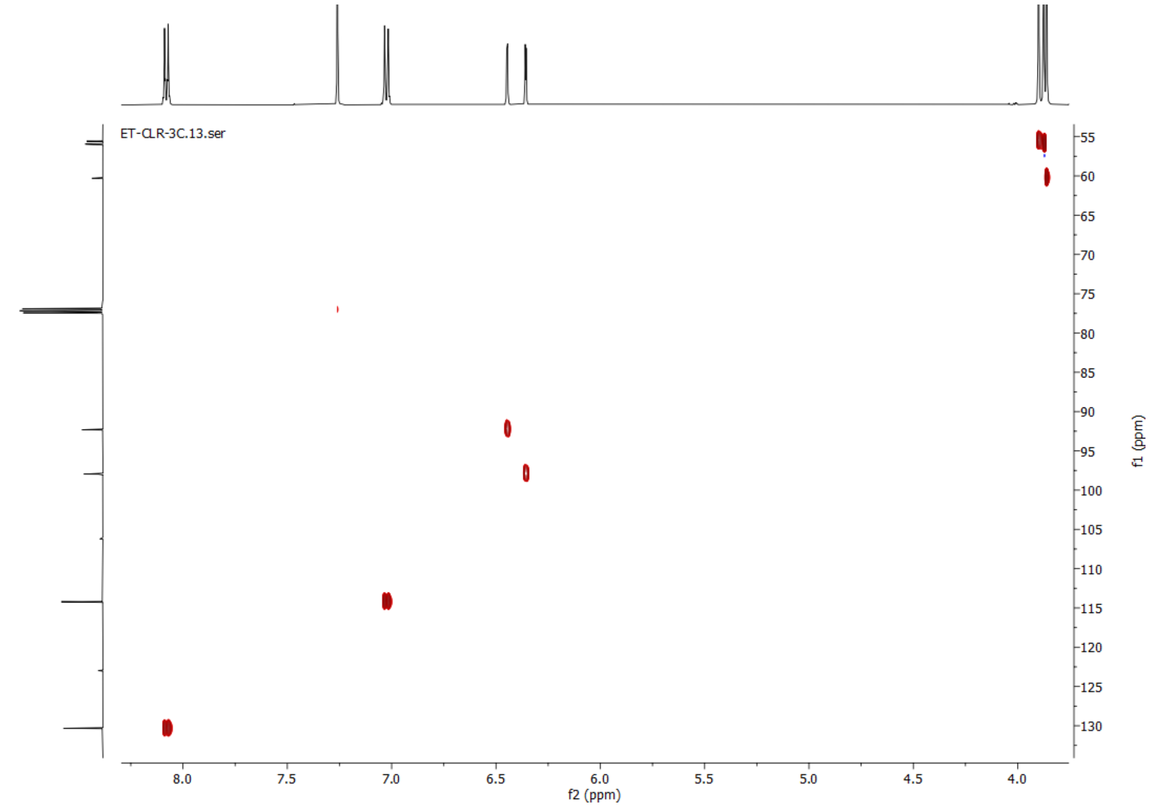

Figure S109. The HSQC spectrum of compound **14** (500/125 MHz, $CDCl_3$)

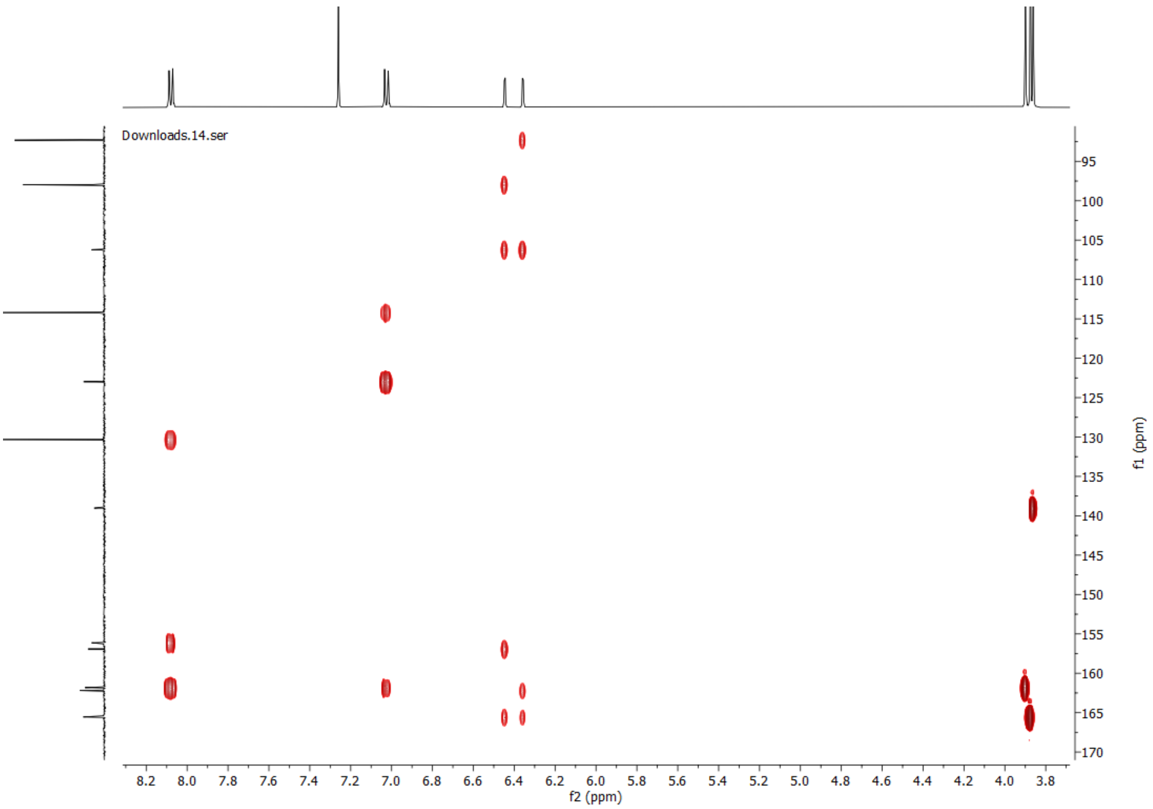

Figure S110. The HMBC spectrum of compound **14** (500/125 MHz, $CDCl_3$)

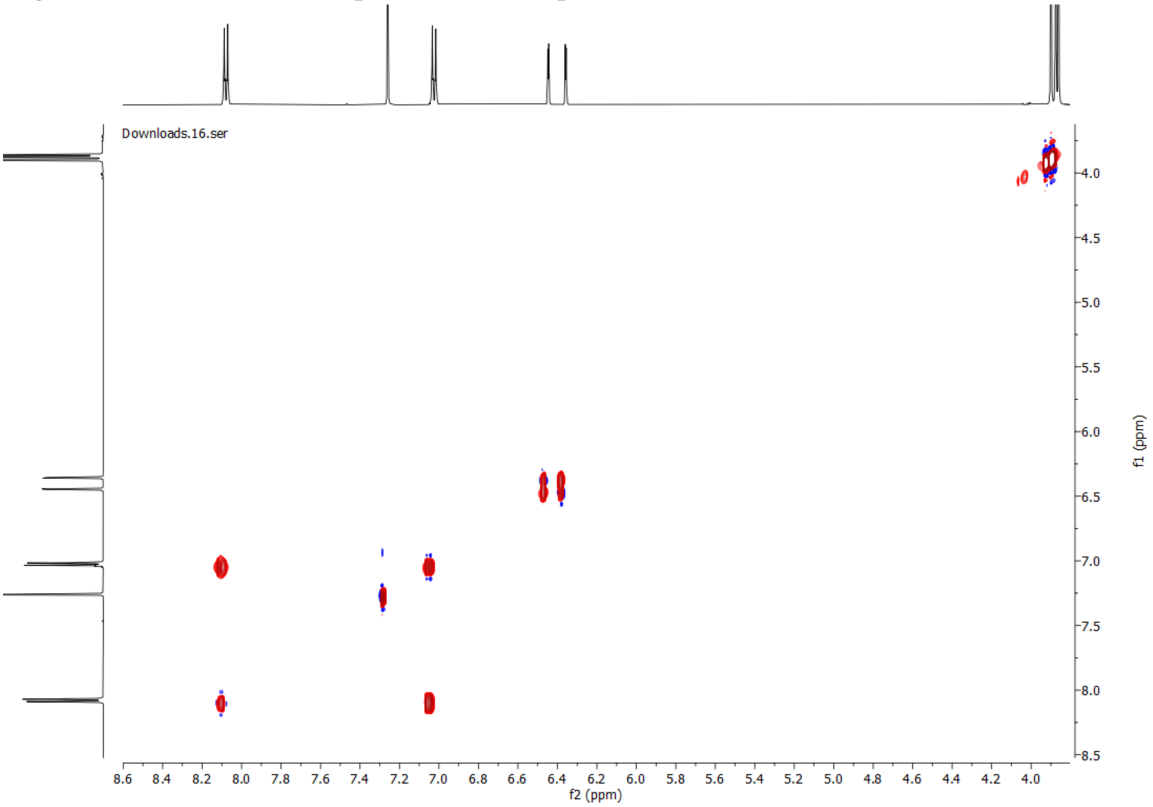

Figure S111. The TOCSY spectrum of compound **14** (500 MHz, $CDCl_3$)

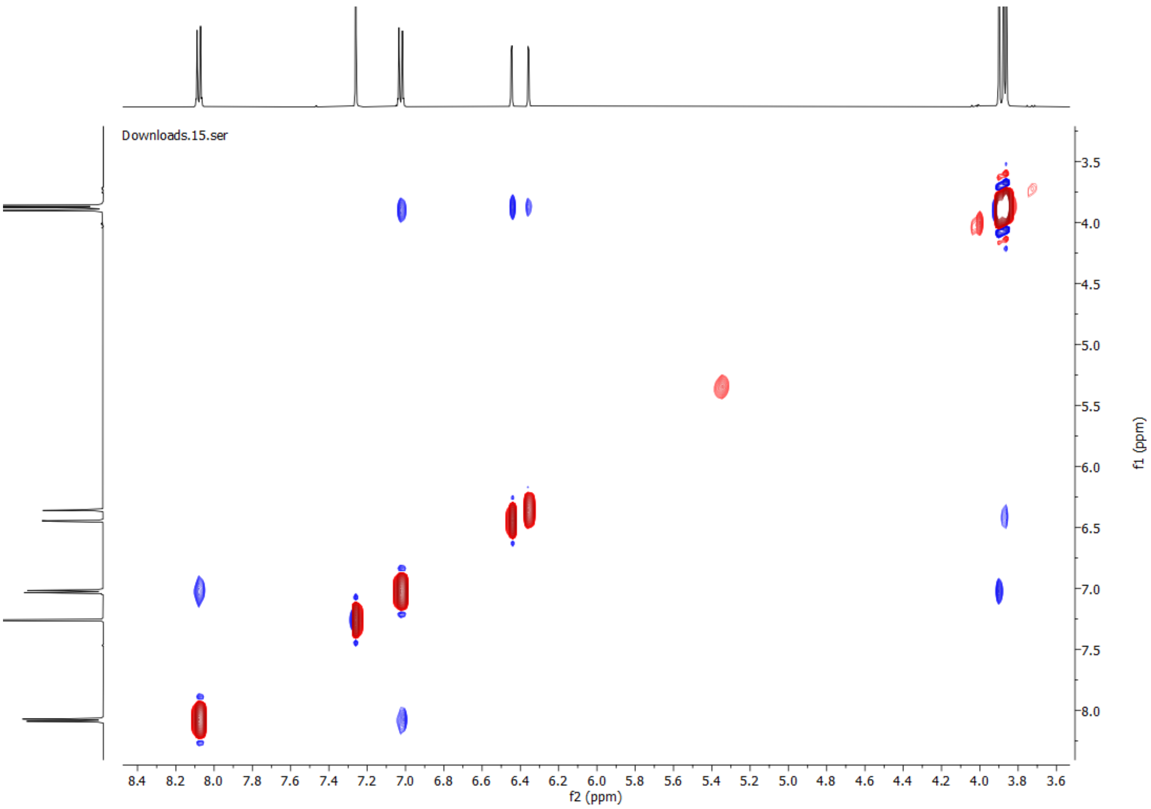

Figure S112. The NOESY Spectrum of compound **14** (500 MHz, $CDCl_3$)

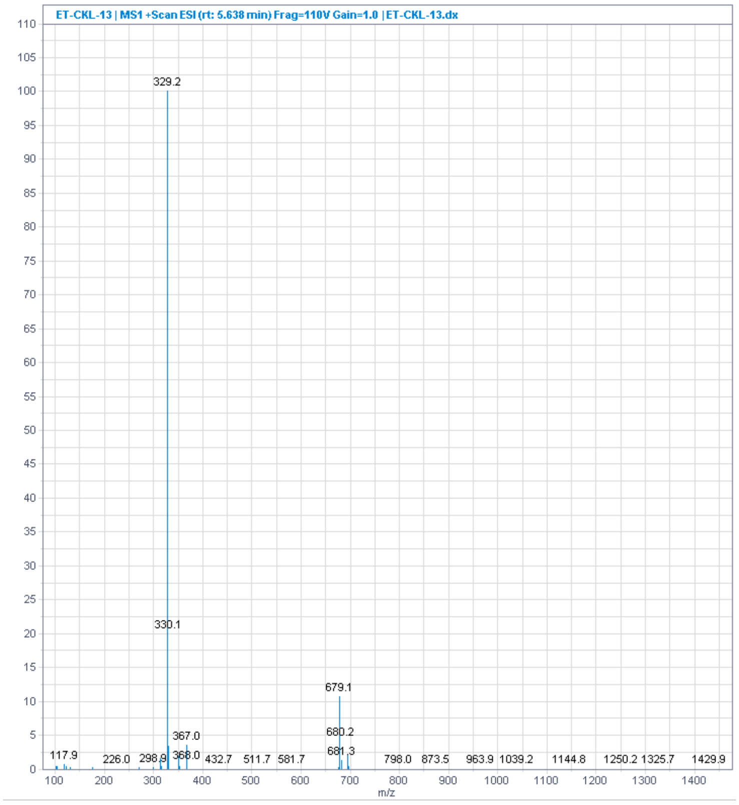

Figure S113. The LC-MS of compound **14**

16. Spectroscopic data for compound 15

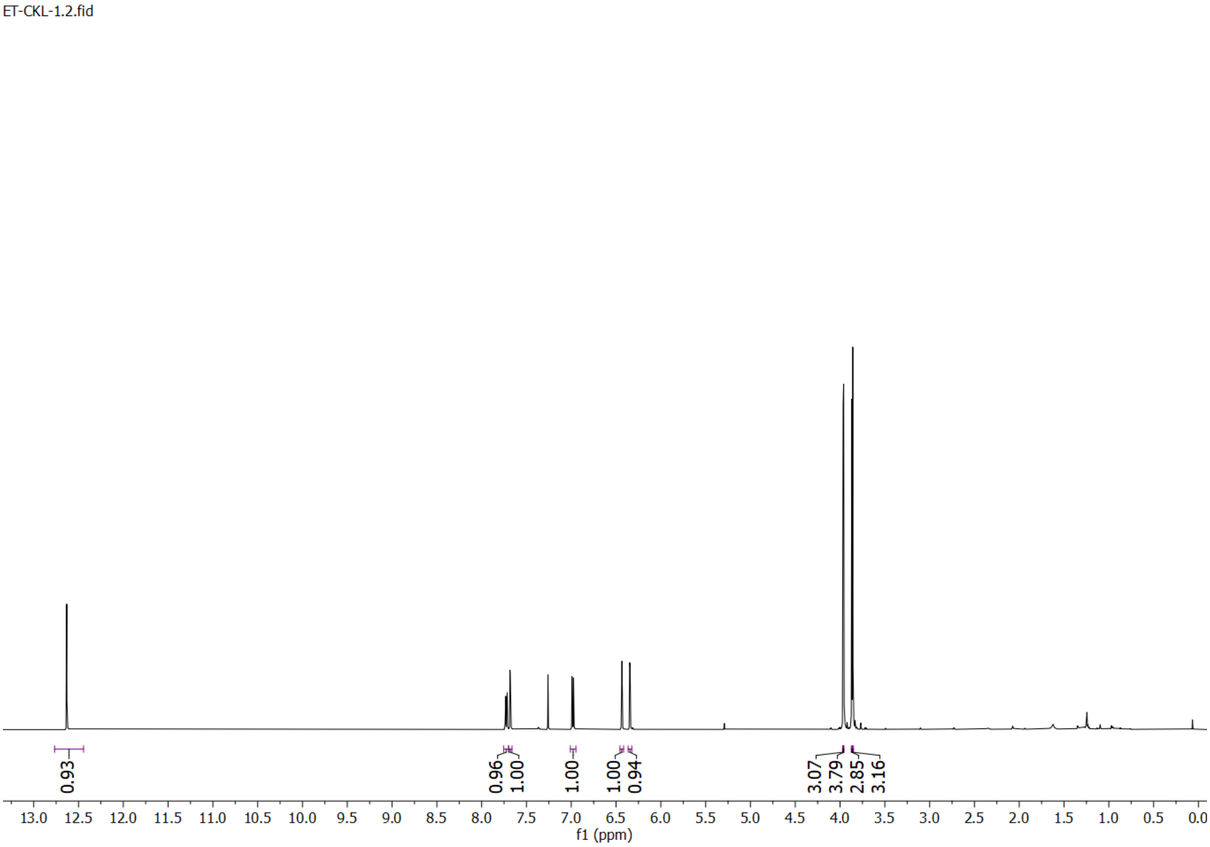

Figure S114. The ^{1}H NMR spectrum of compound **15** (500 MHz, $CDCl_3$)

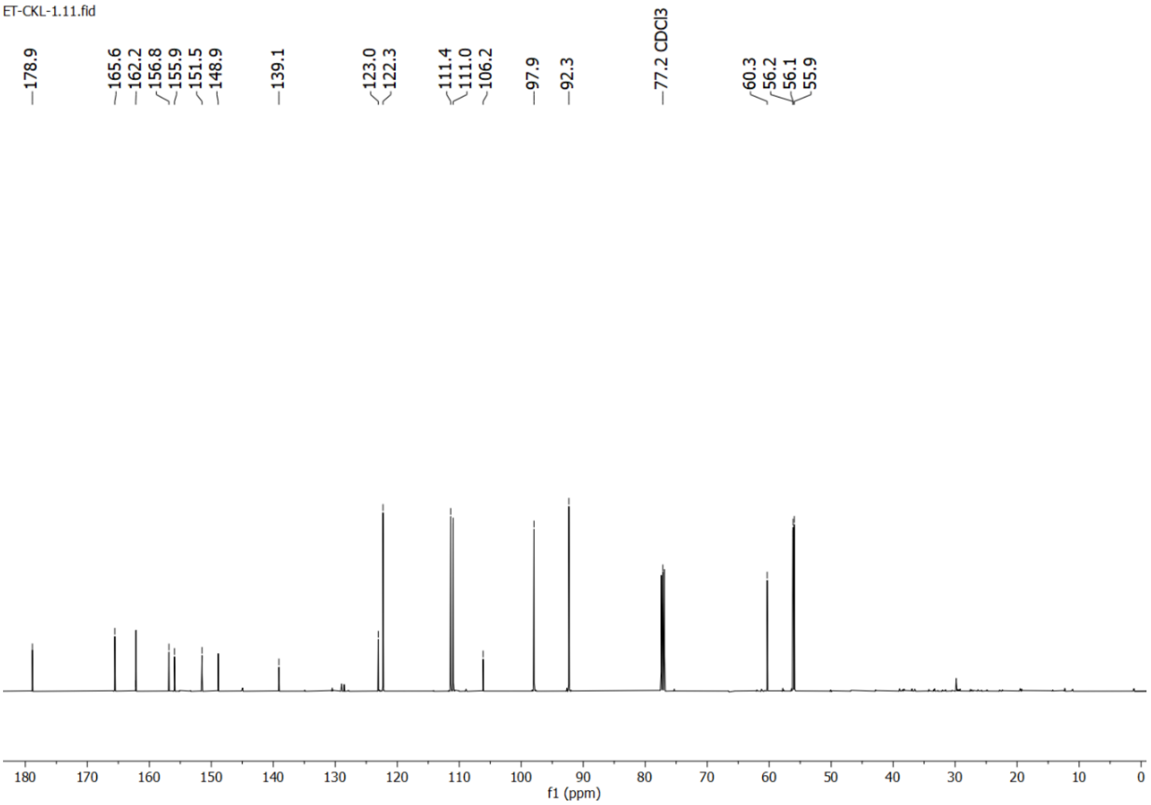

Figure S115. The ^{13}C NMR spectrum of compound **15** (125 MHz, $CDCl_3$)

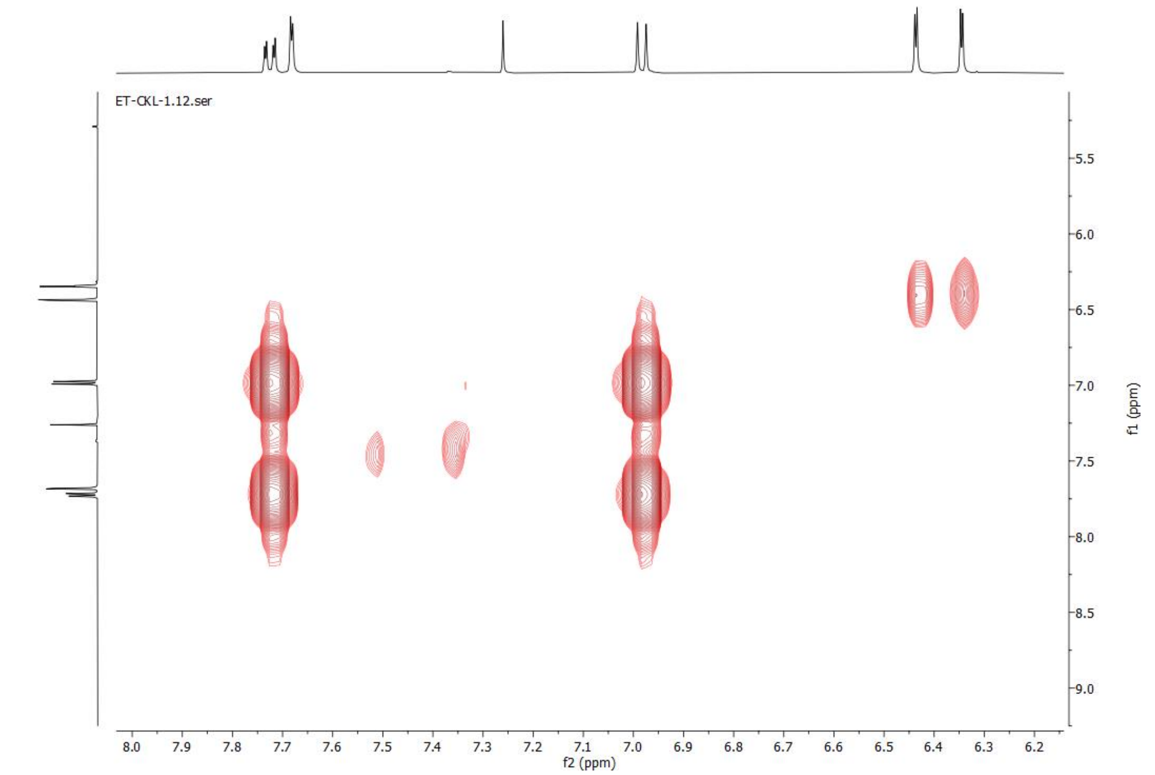

Figure S116. The COSY spectrum of compound **15** (500 MHz, $CDCl_3$)

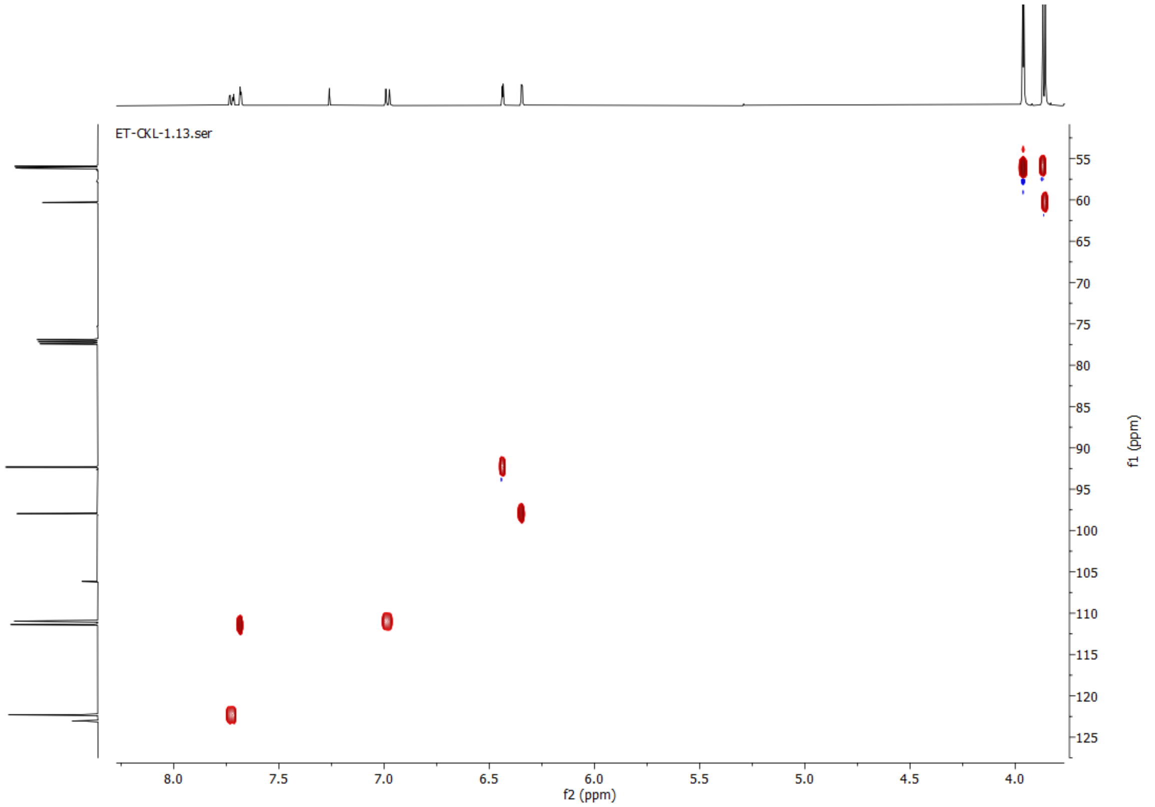

Figure S117. The HSQC spectrum of compound **15** (500/125 MHz, $CDCl_3$)

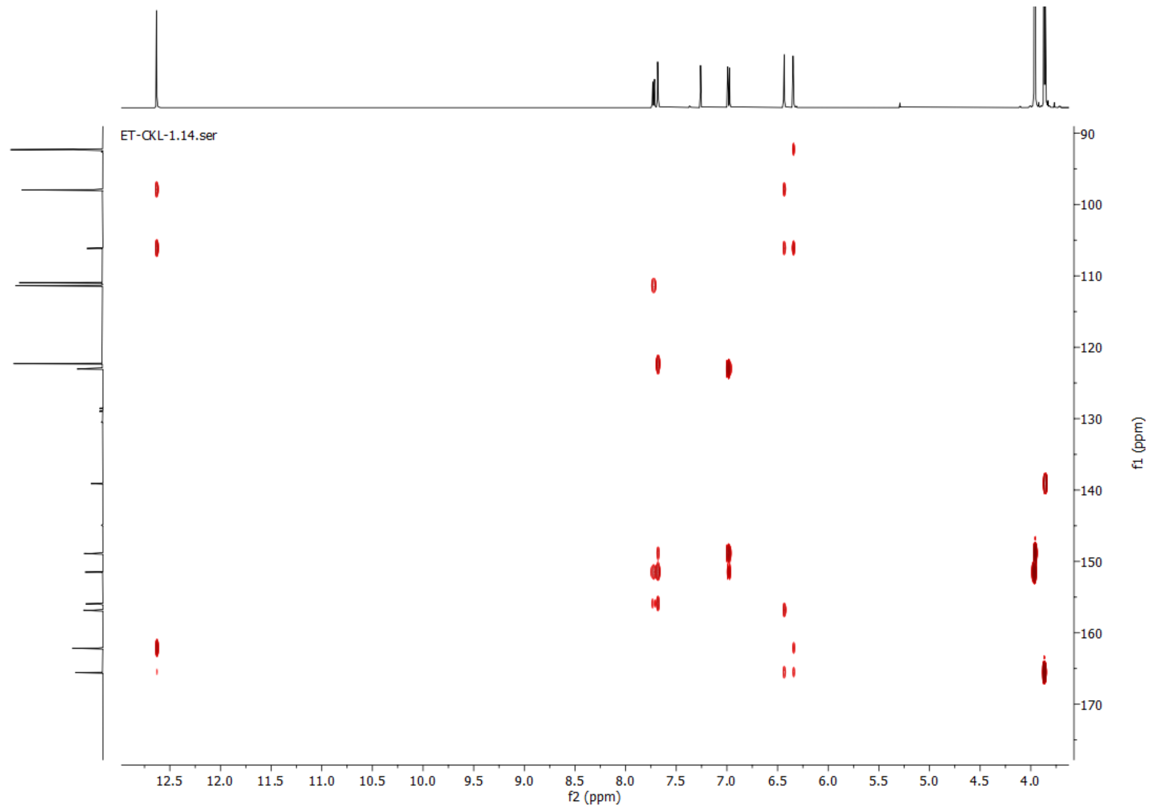

Figure S118. The HMBC spectrum of compound **15** (500 MHz, $CDCl_3$)

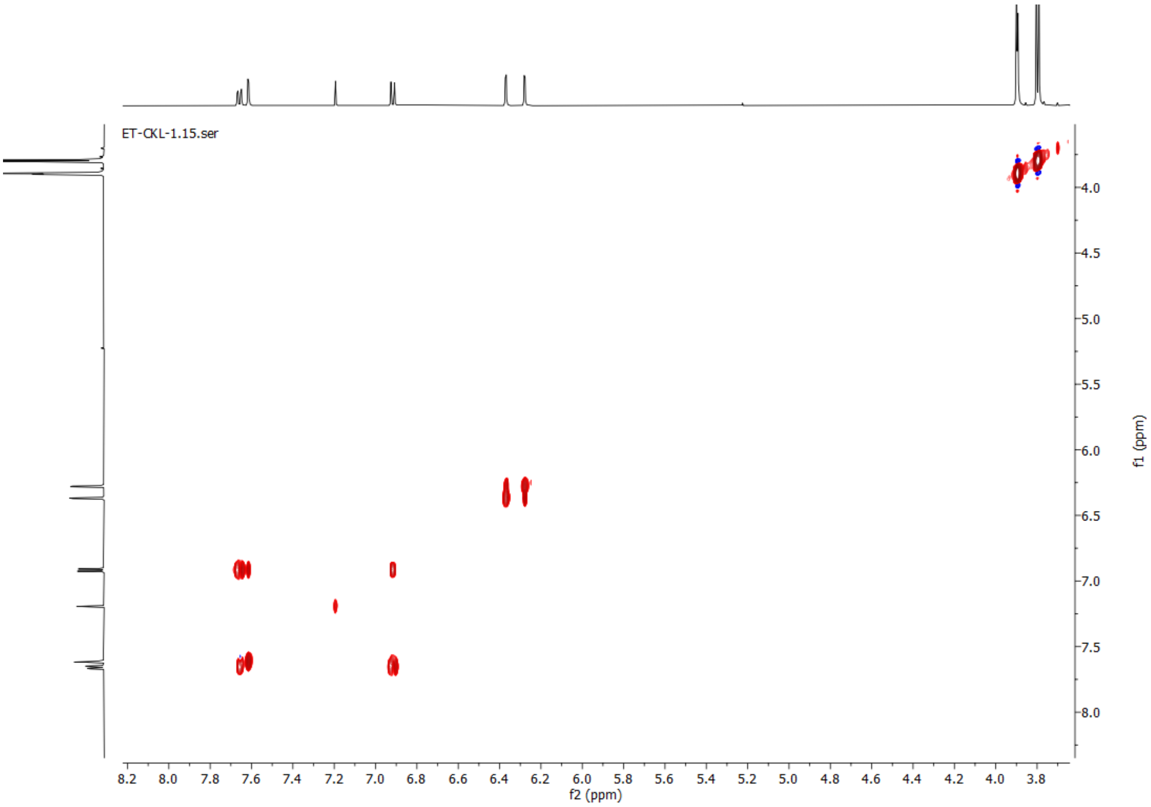

Figure S119. The TOCSY spectrum of compound **15** (500 MHz, $CDCl_3$)

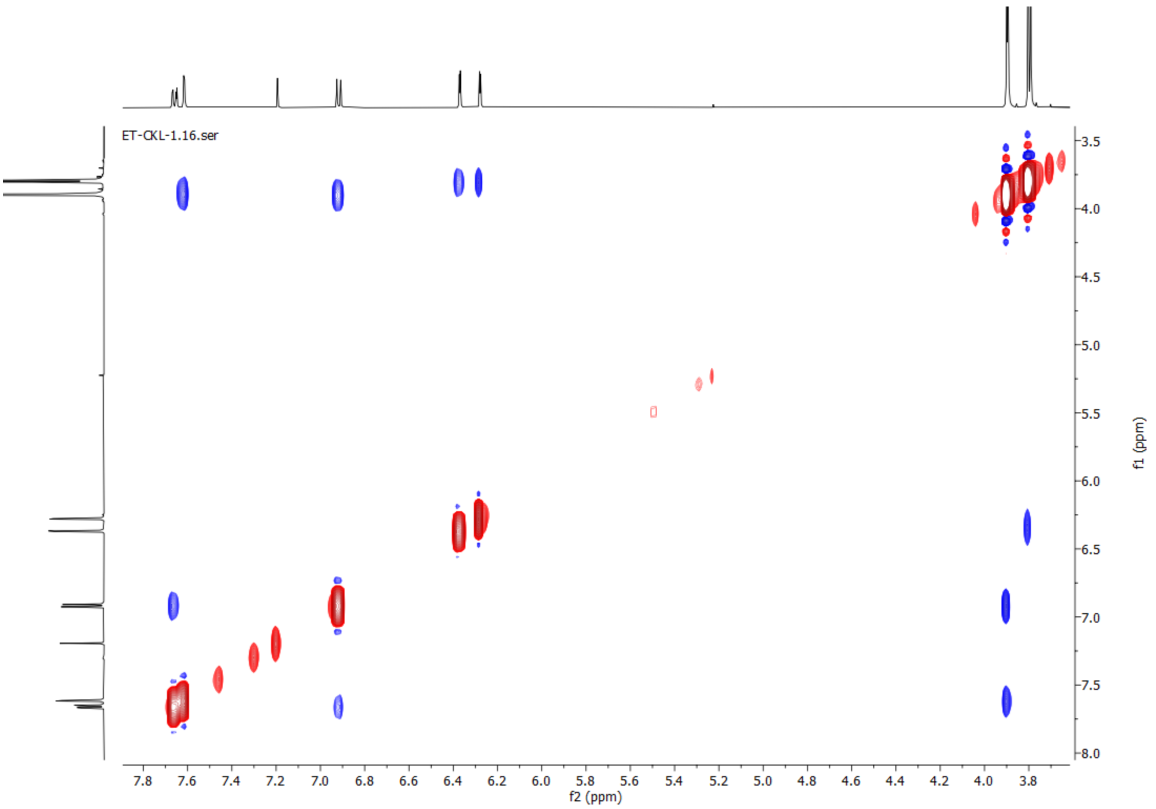

Figure S120. The NOESY spectrum of compound **15** (500 MHz, $CDCl_3$)

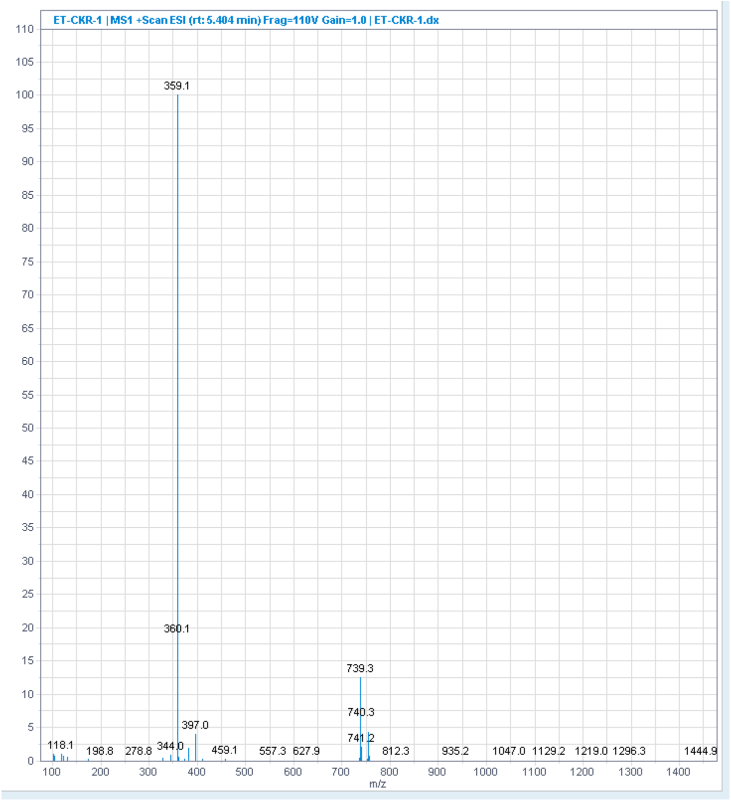

Figure S121. The LC-MS of compound **15**

17. Spectroscopic data for compound 16

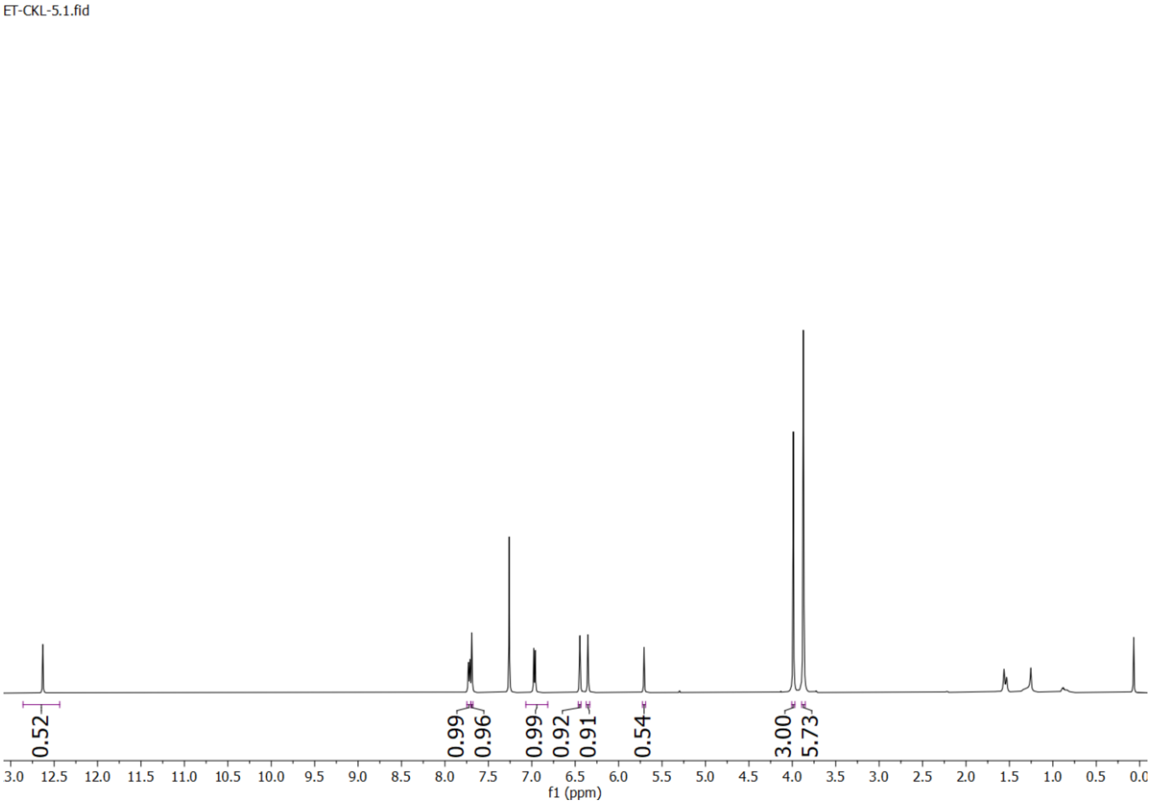

Figure S122. The ^{1}H NMR spectrum of compound **16** (500 MHz, $CDCl_3$)

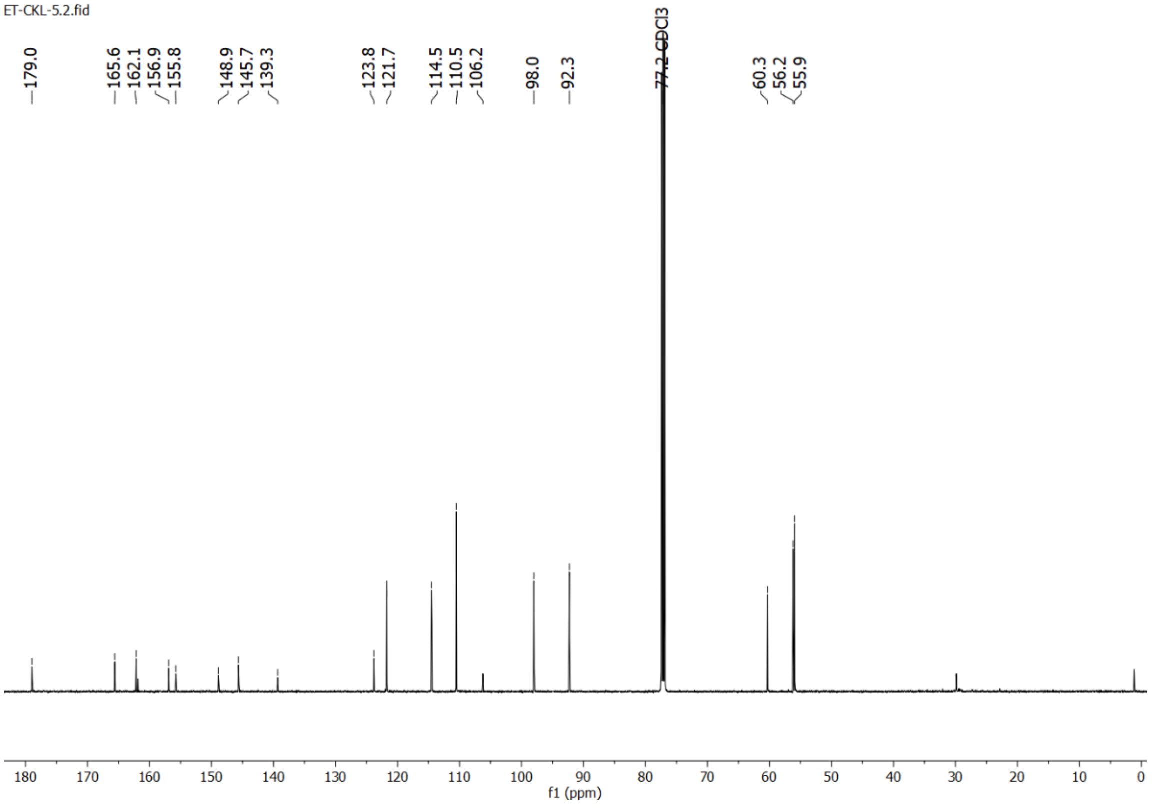

Figure S123. The ^{13}C NMR spectrum for compound **16** (125 MHz, $CDCl_3$)

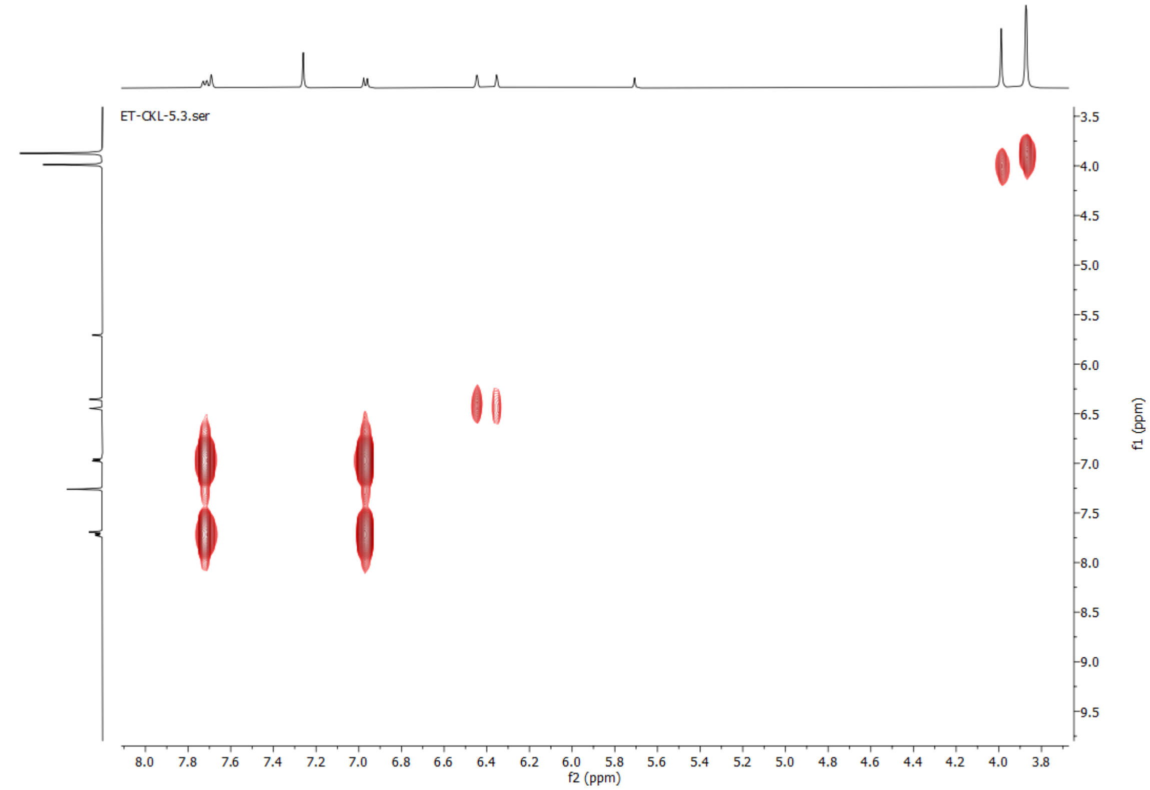

Figure S124. The COSY spectrum of compound **16** (500 MHz, $CDCl_3$)

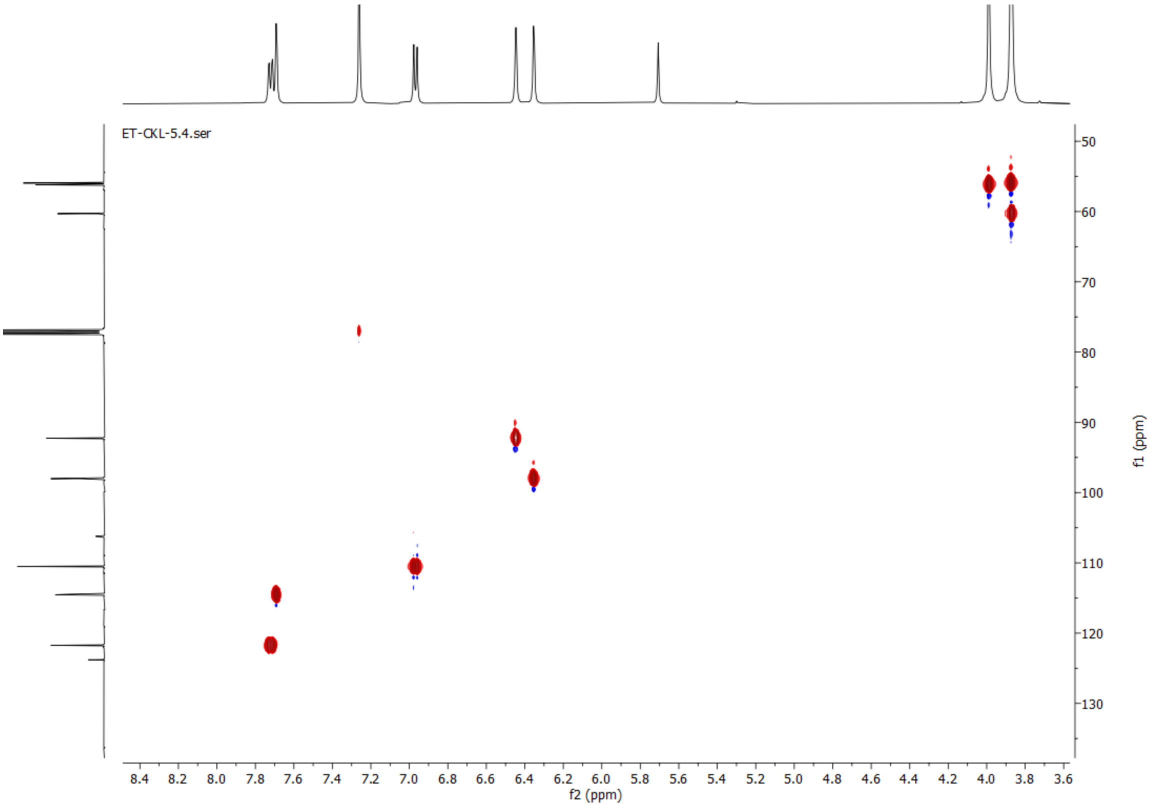

Figure S125. The HSQC spectrum of compound **16** (500 MHz, $CDCl_3$)

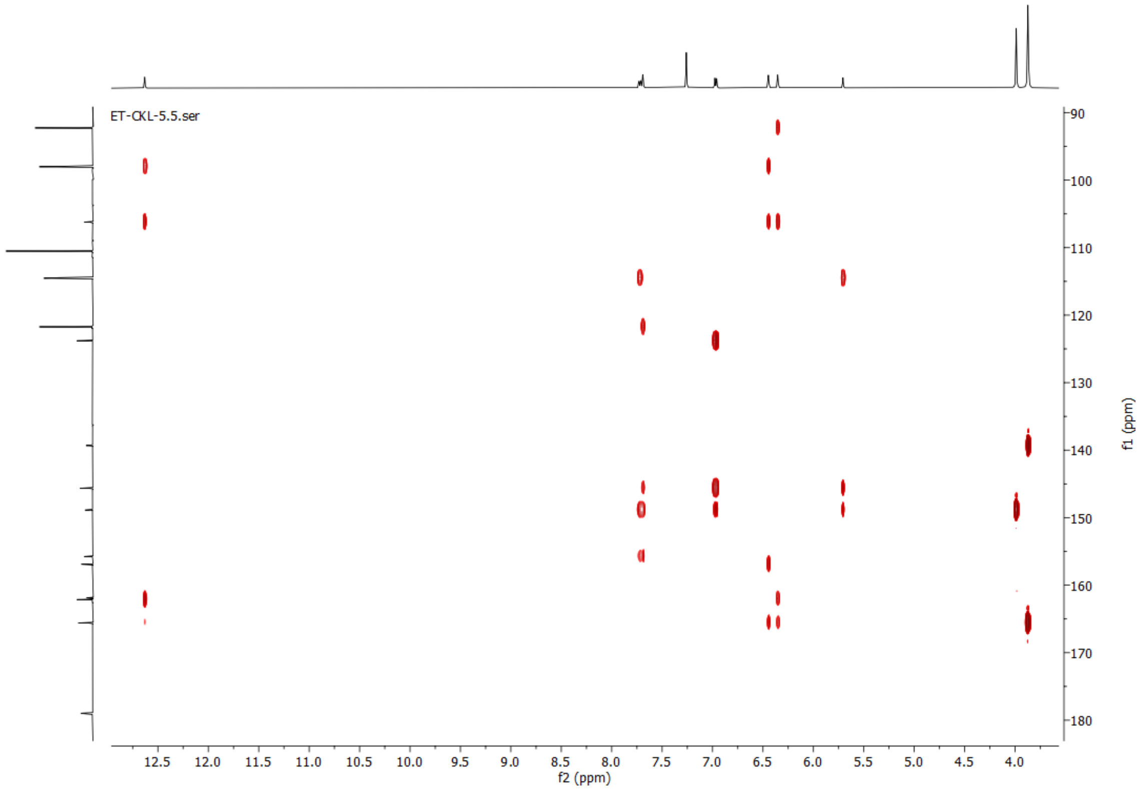

Figure S126. The HMBC spectrum of compound **16** (500/125 MHz, $CDCl_3$)

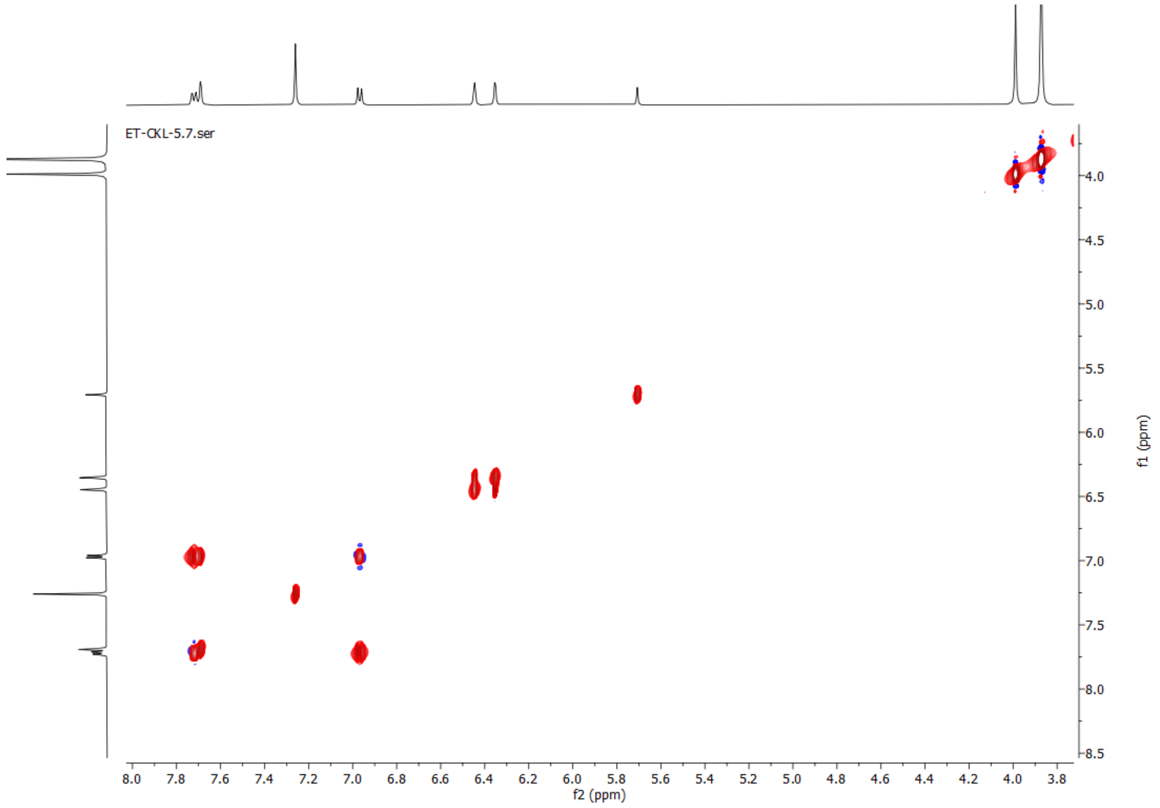

Figure S127. The TOCSY spectrum of compound **16** (500 MHz, $CDCl_3$)

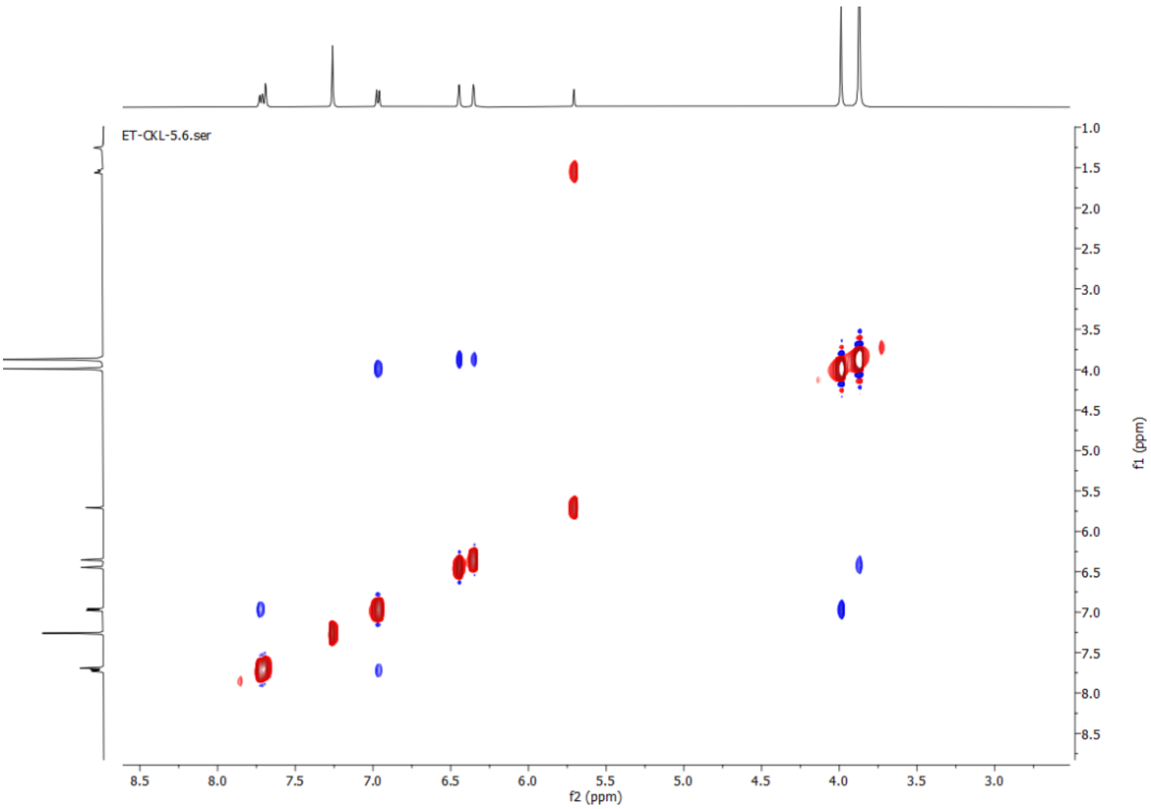

Figure S128. The NOESY spectrum of compound **16** (500 MHz, $CDCl_3$)

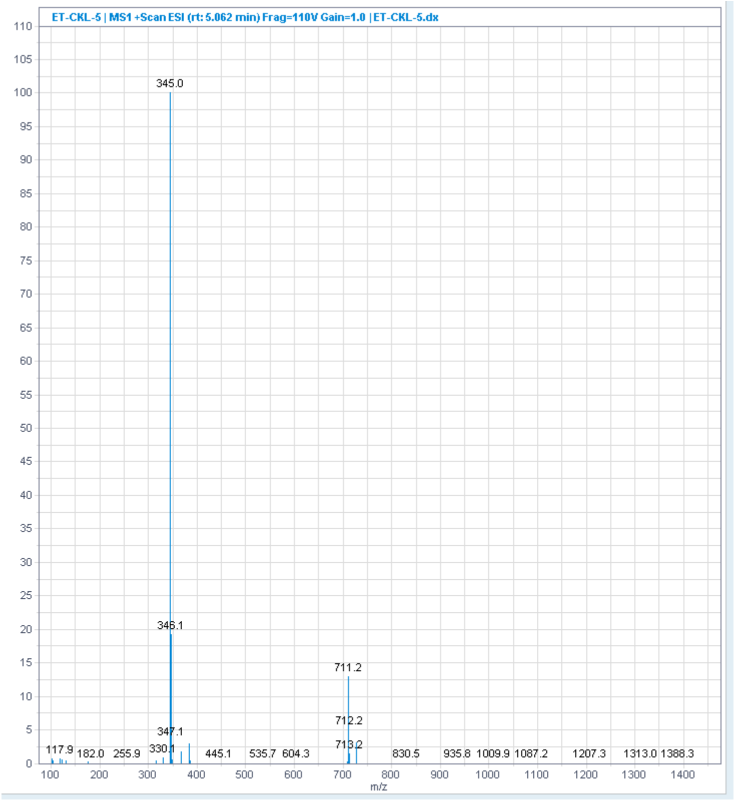

Figure S129. The LC-MS of compound **16**

18. Spectroscopic data for compound 17

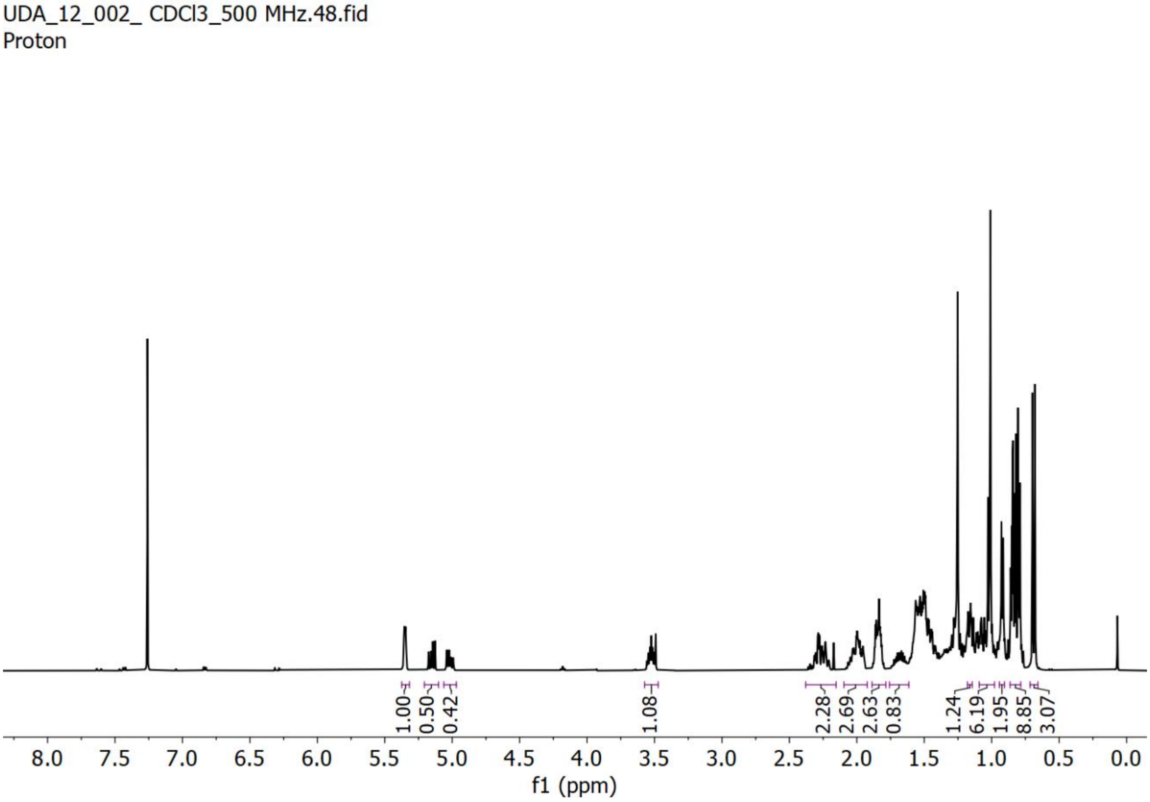

Figure S130. The ^{1}H NMR spectrum of compound **17** (500 MHz, $CDCl_3$)

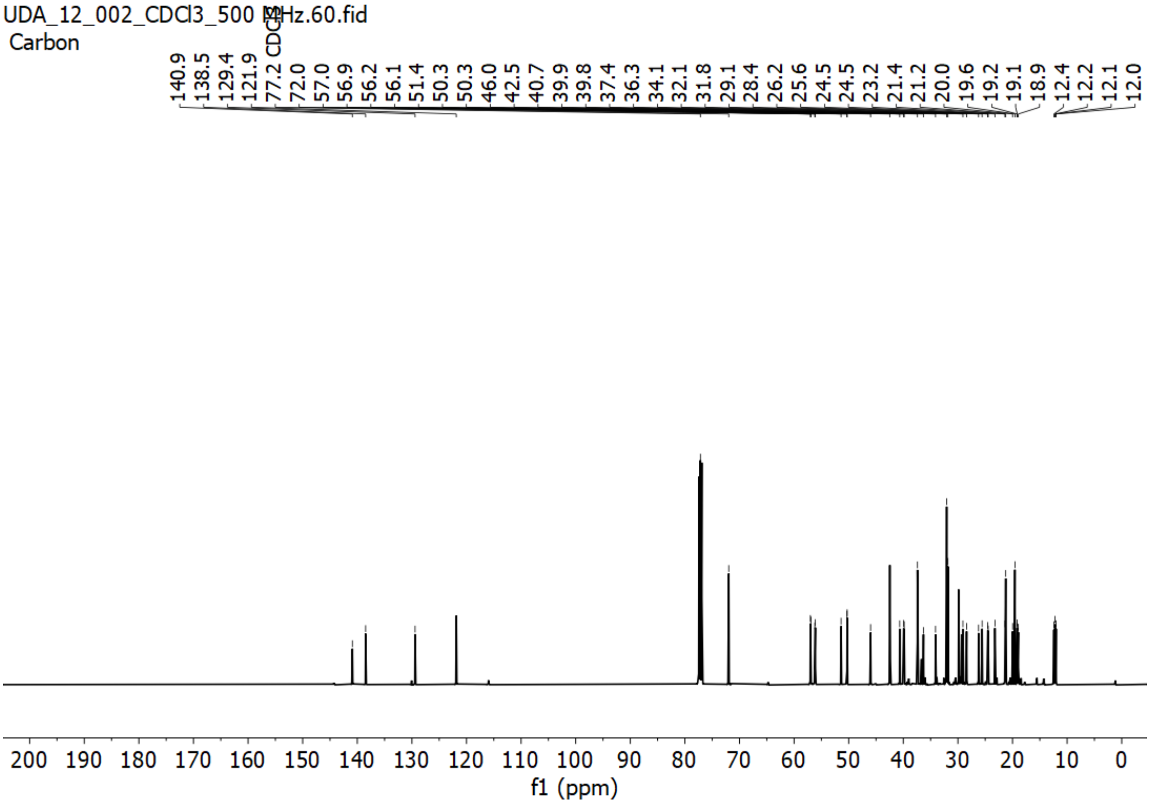

Figure S130. The ^{13}C NMR spectrum of compound **17** (125 MHz, $CDCl_3$)

19. Spectroscopic data for compound 18

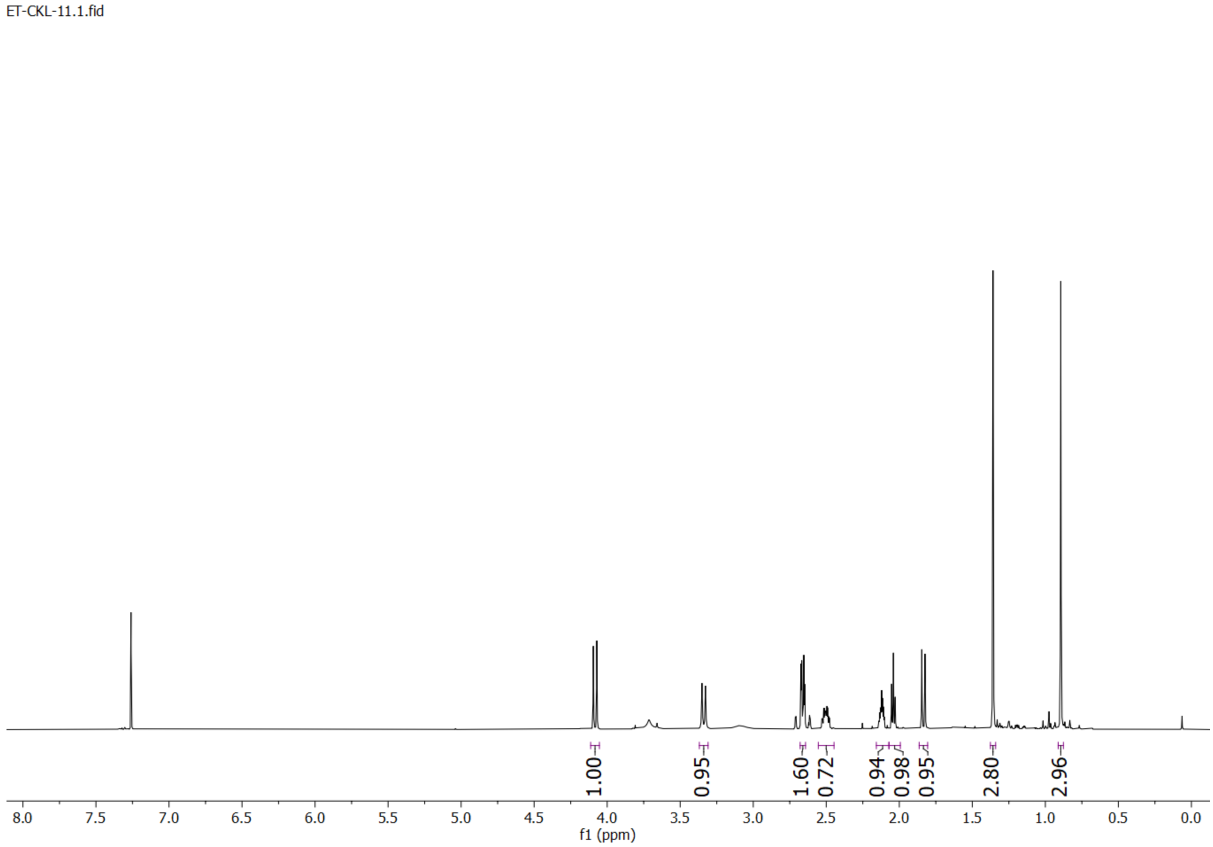

Figure S131. The ^{1}H NMR spectrum of compound **18** (500 MHz, $CDCl_3$)

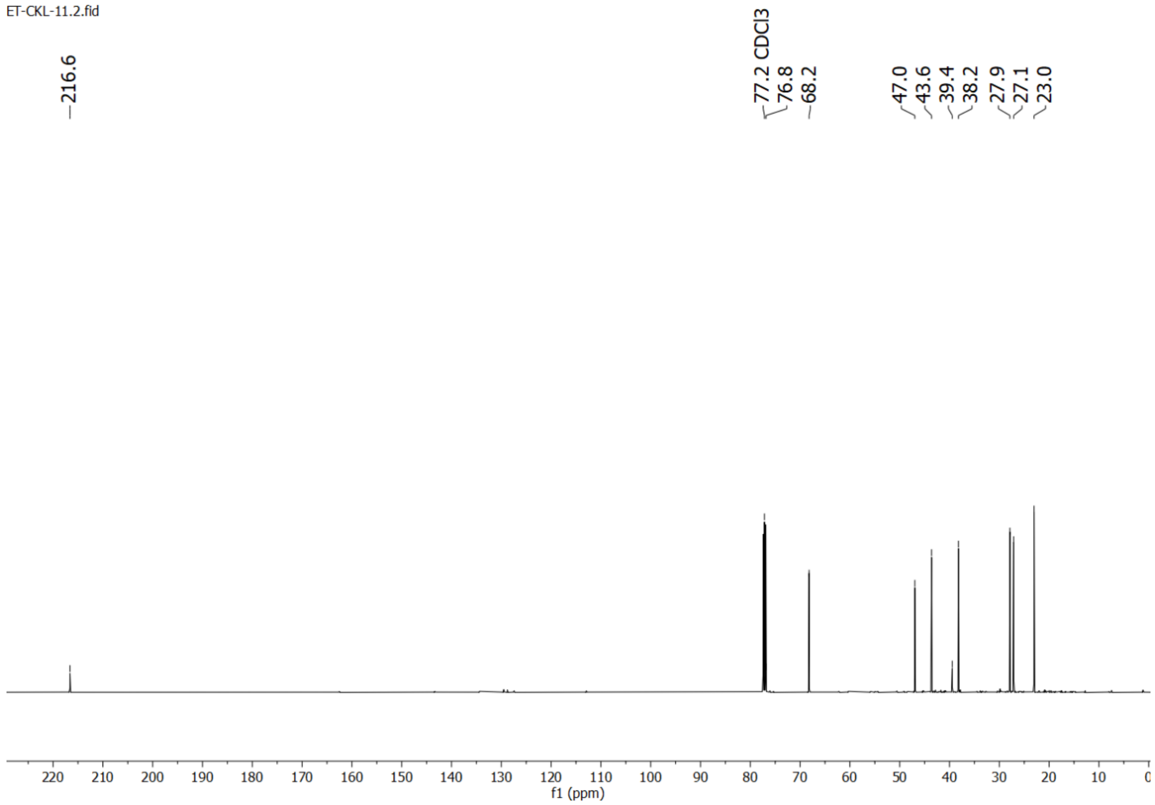

Figure S132. The ^{13}C NMR spectrum of compound **18** (125 MHz, CDCl3)

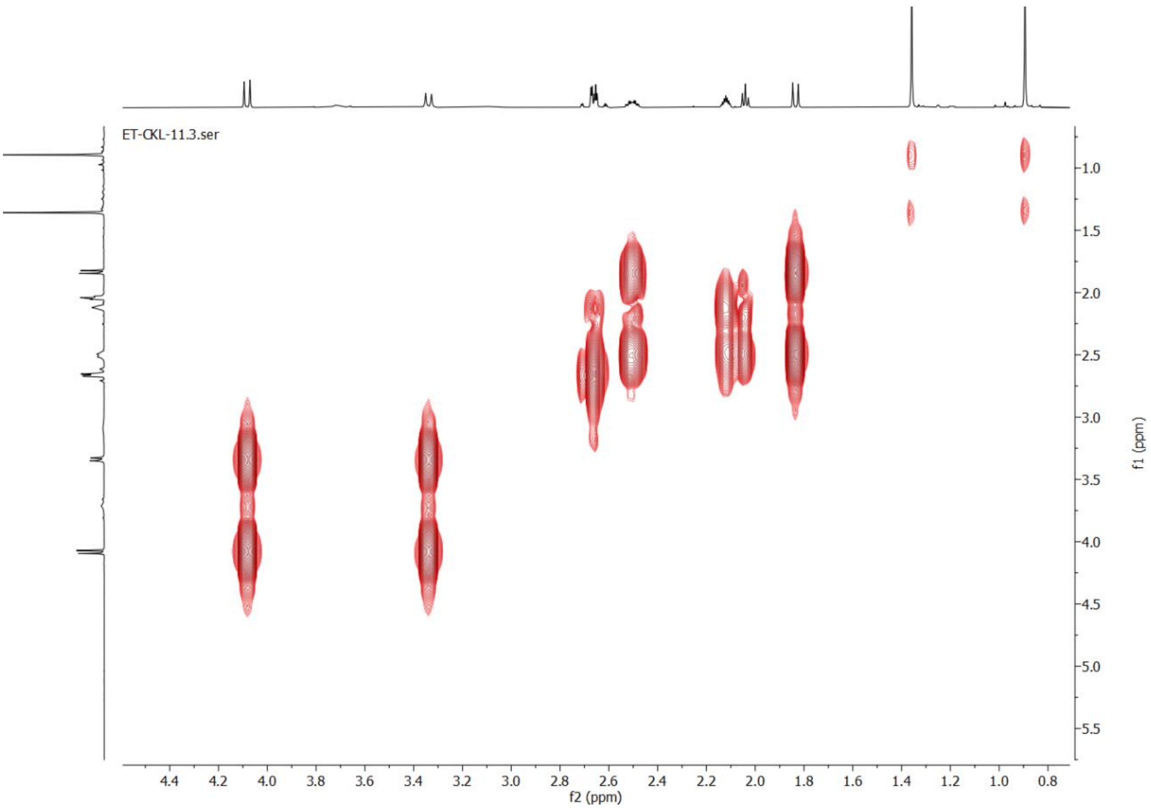

Figure S133. The COSY spectrum of compound **18** (500 MHz, $CDCl_3$)

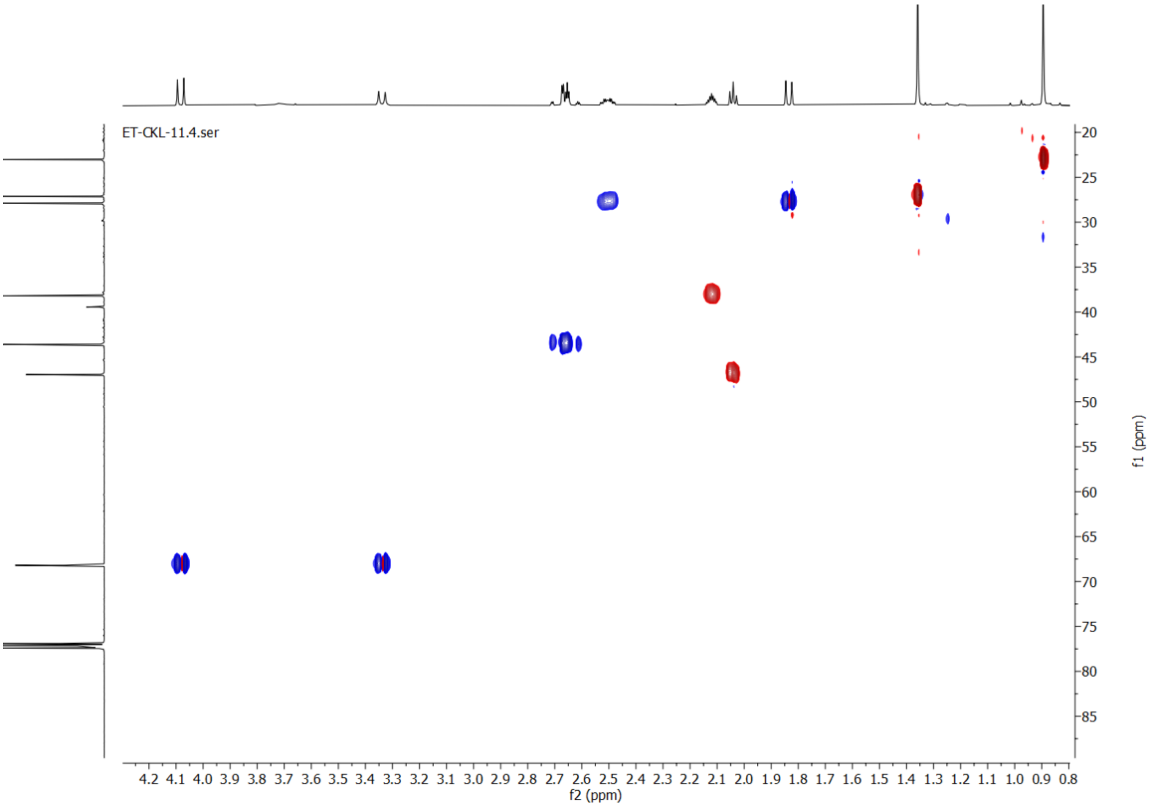

Figure S134. The HSQC spectrum of compound **18** (500/125 MHz, $CDCl_3$)

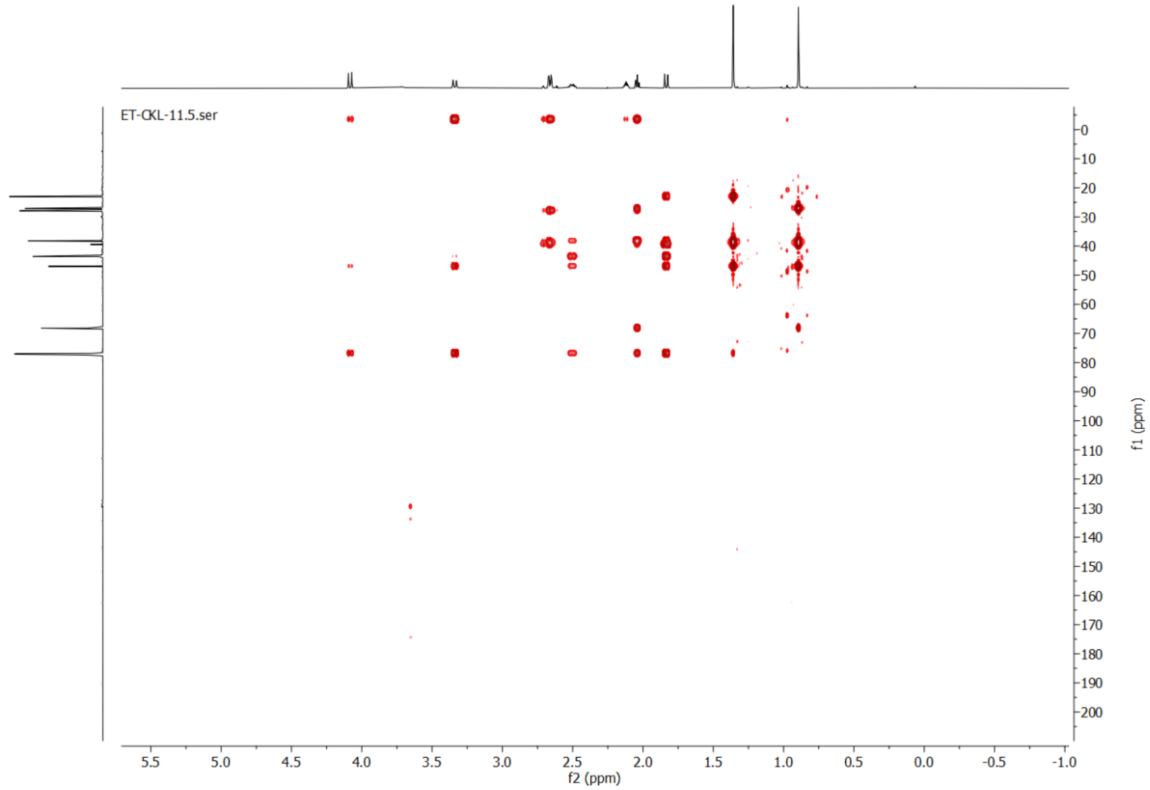

Figure S135. The HMBC spectrum of compound **18** (500/125 MHz, CDCl3)

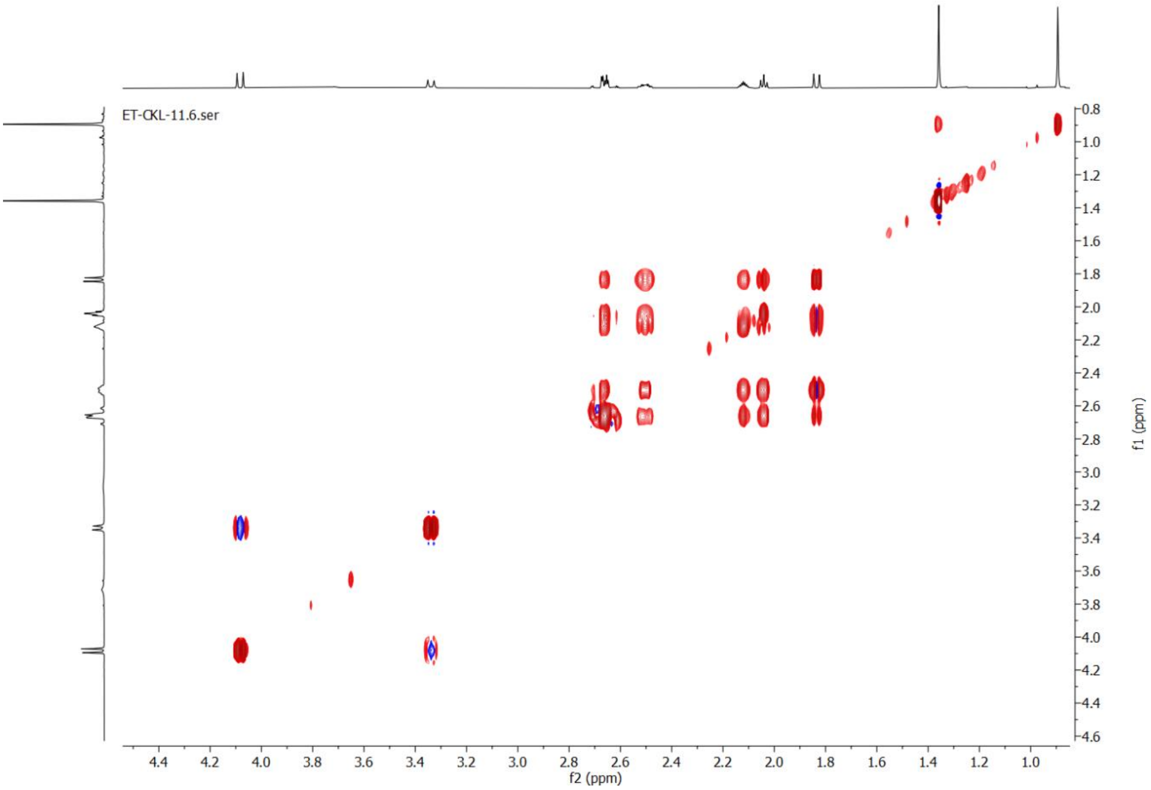

Figure S136. The TOCSY spectrum of compound **18** (500 MHz, $CDCl_3$)

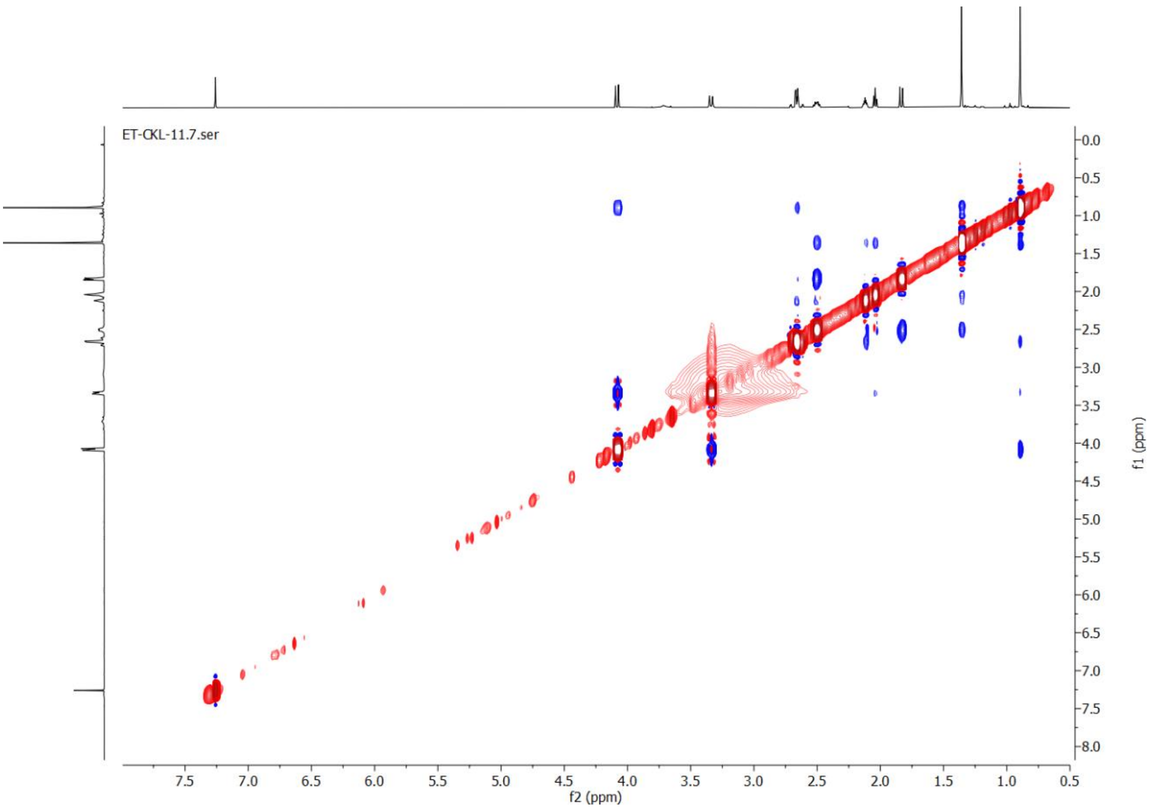

Figure S137. The NOESY spectrum of compound **18** (500 MHz, $CDCl_3$)

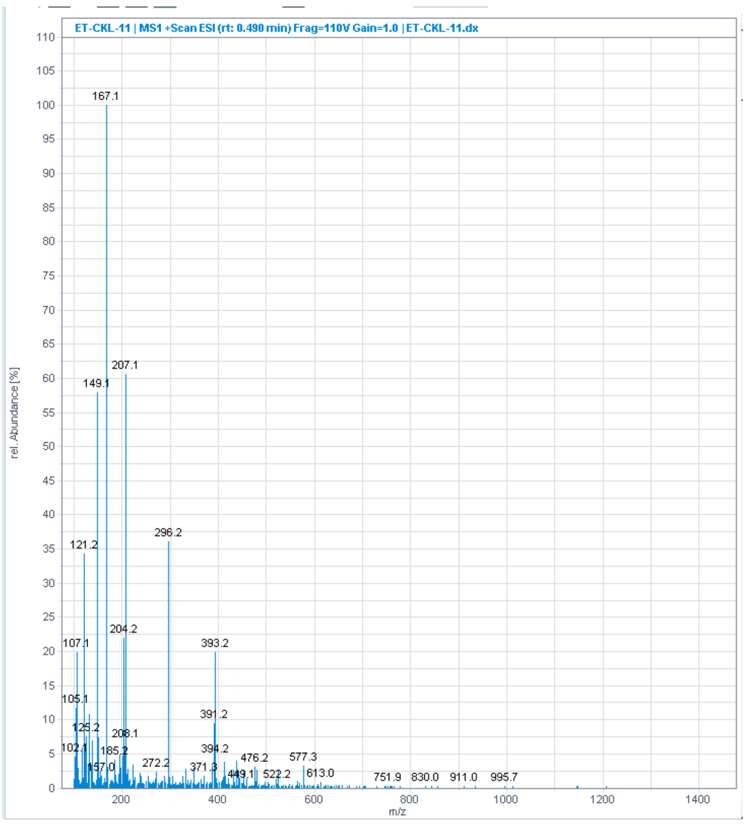

Figure S138. The LC-MS of compound **18**

20. Spectroscopic data for compound 19

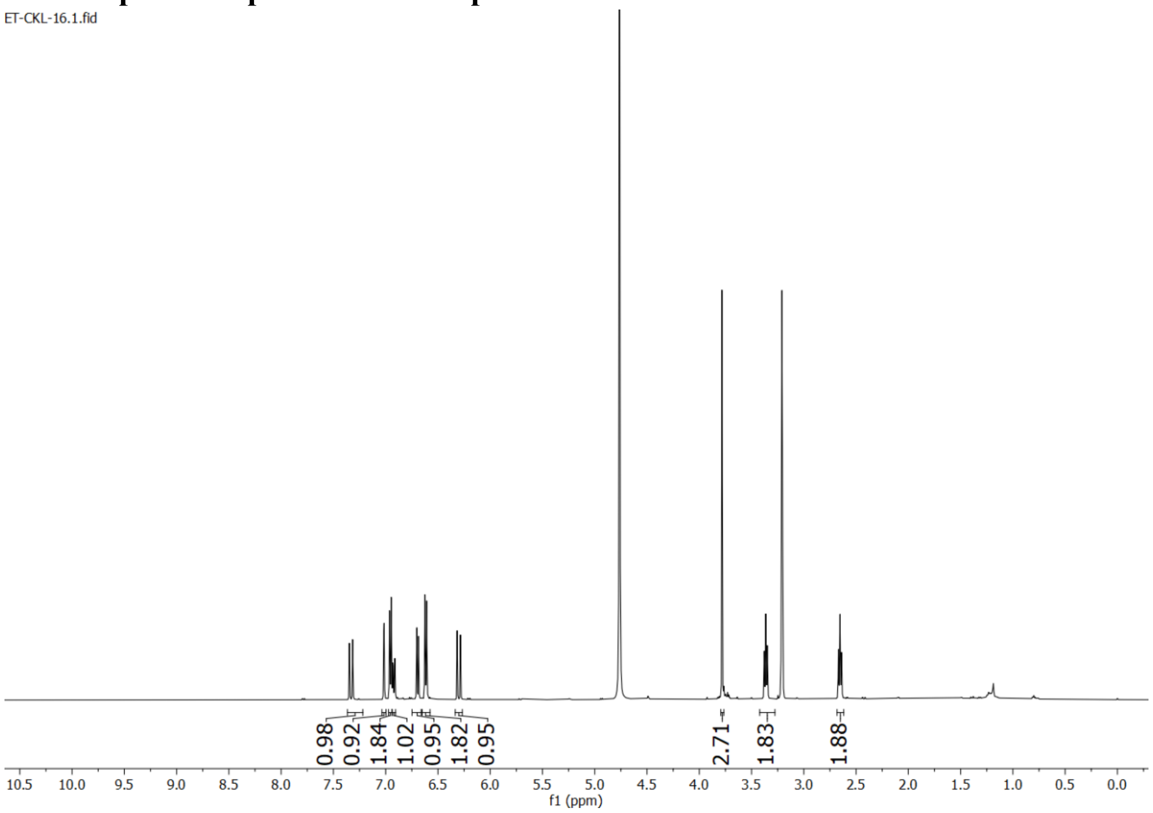

Figure S139. The ^{1}H NMR spectrum of compound **19** (500 MHz, CD_3OD)

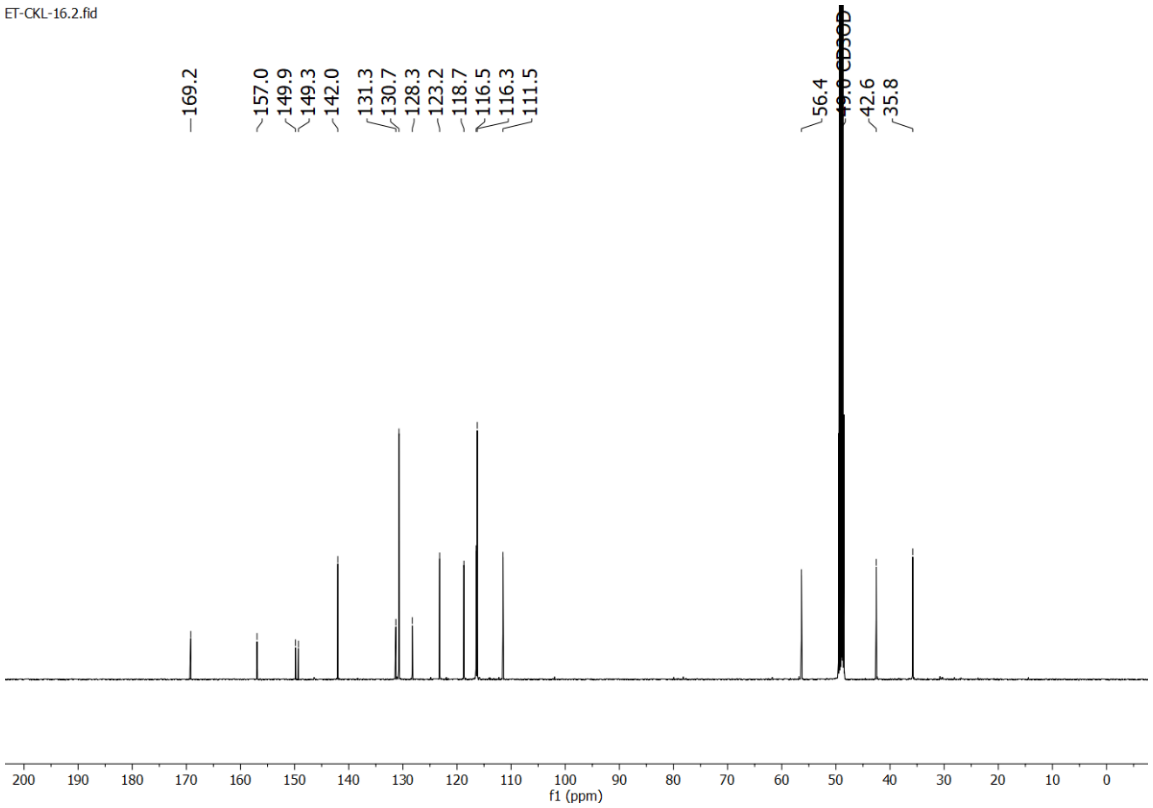

Figure 140. The ^{13}C NMR spectrum of compound **19** (125 MHz, CD_3OD)

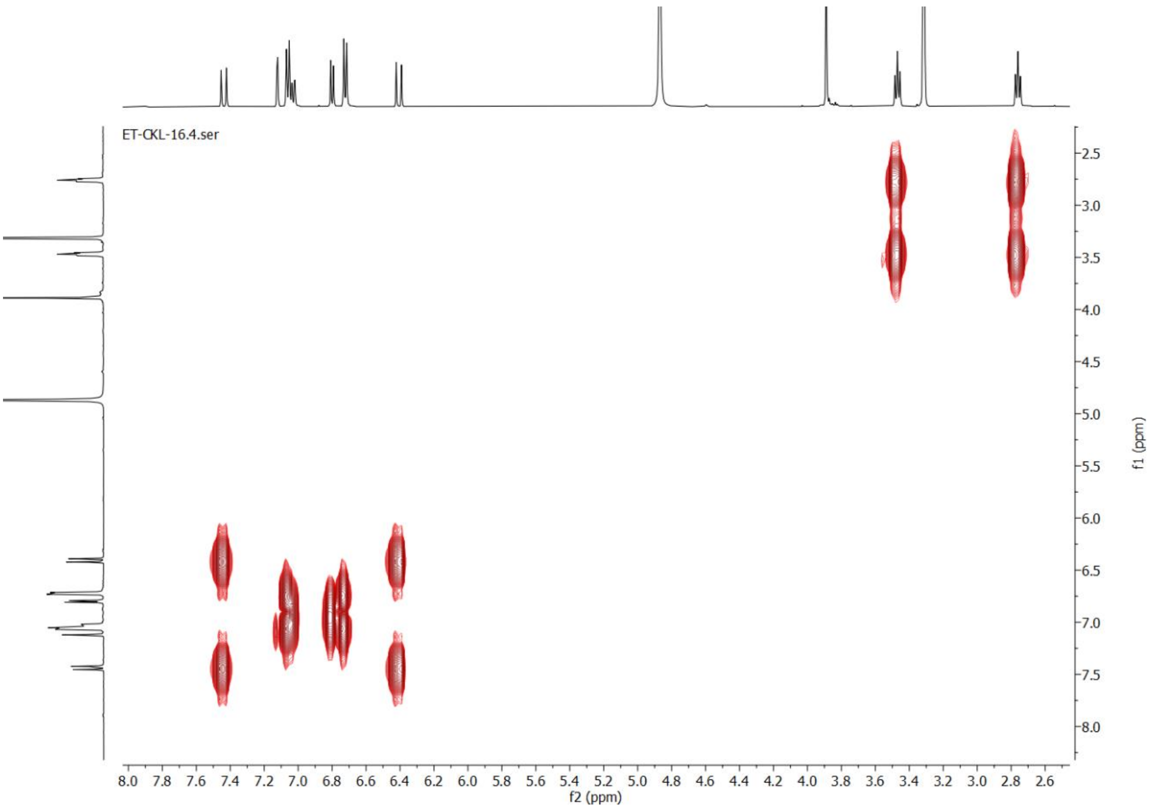

Figure 141. The COSY spectrum of compound **19** (500 MHz, CD_3OD)

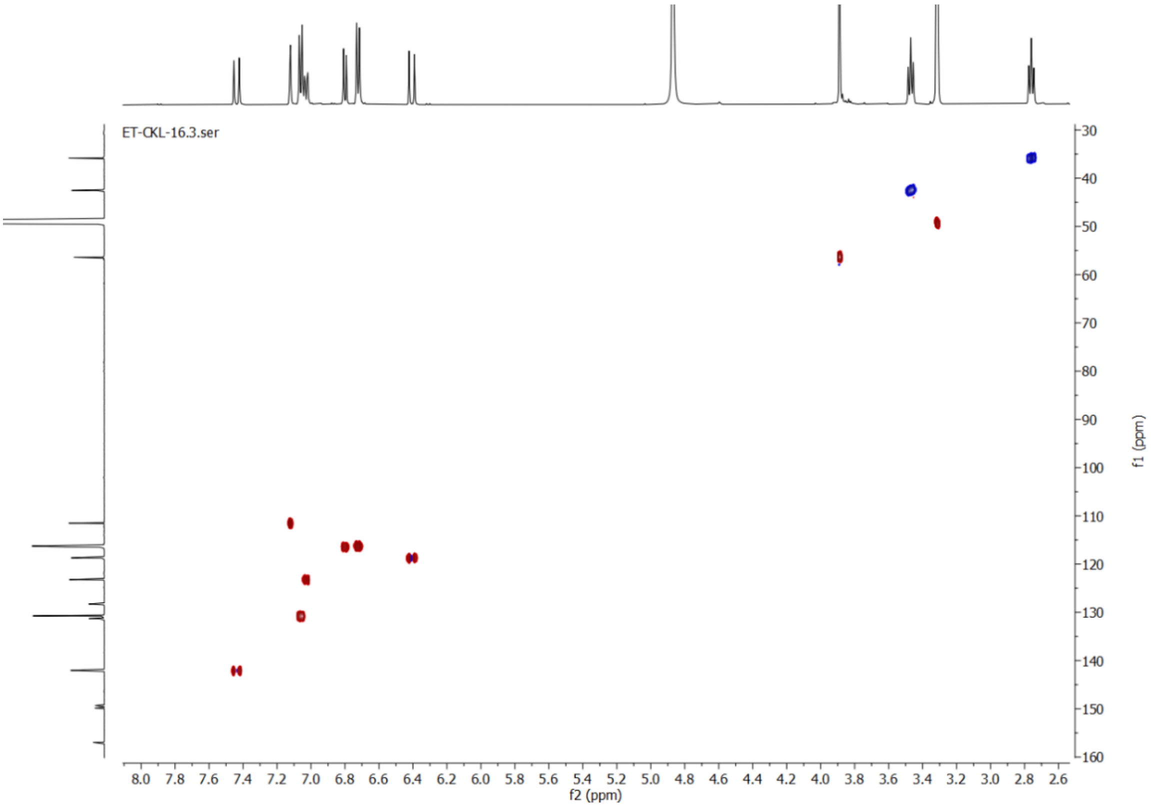

Figure 142. The HSQC spectrum of compound **19** (500/125 MHz, CD_3OD)

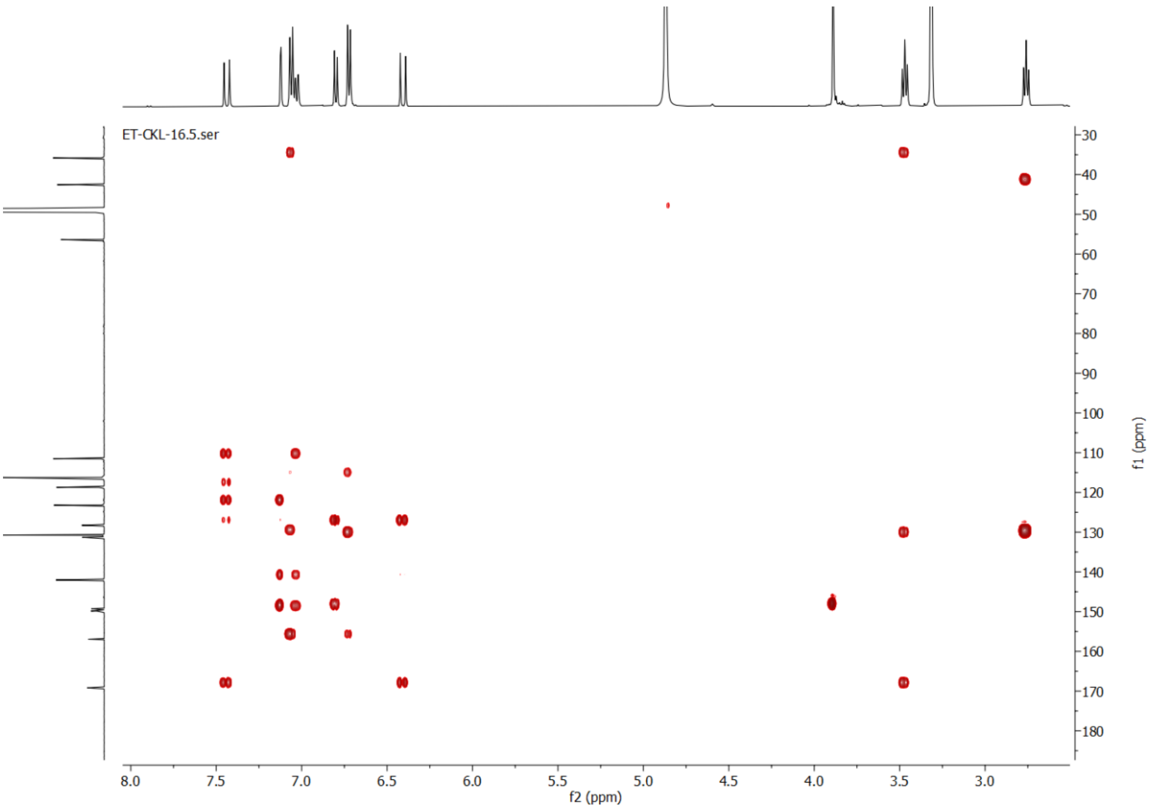

Figure 143. The HMBC spectrum of compound **19** (500/125 MHz, CD_3OD)

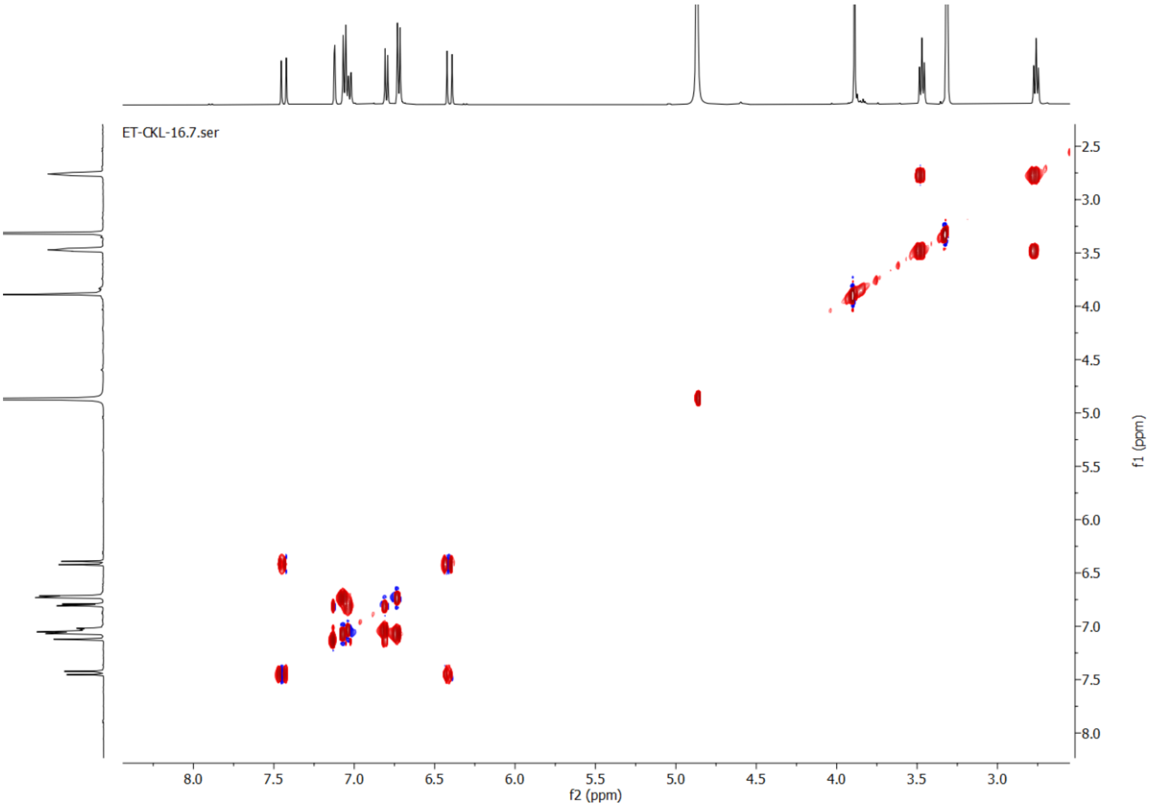

Figure S144. The TOCSY spectrum of compound **19** (500 MHz, CD_3OD)

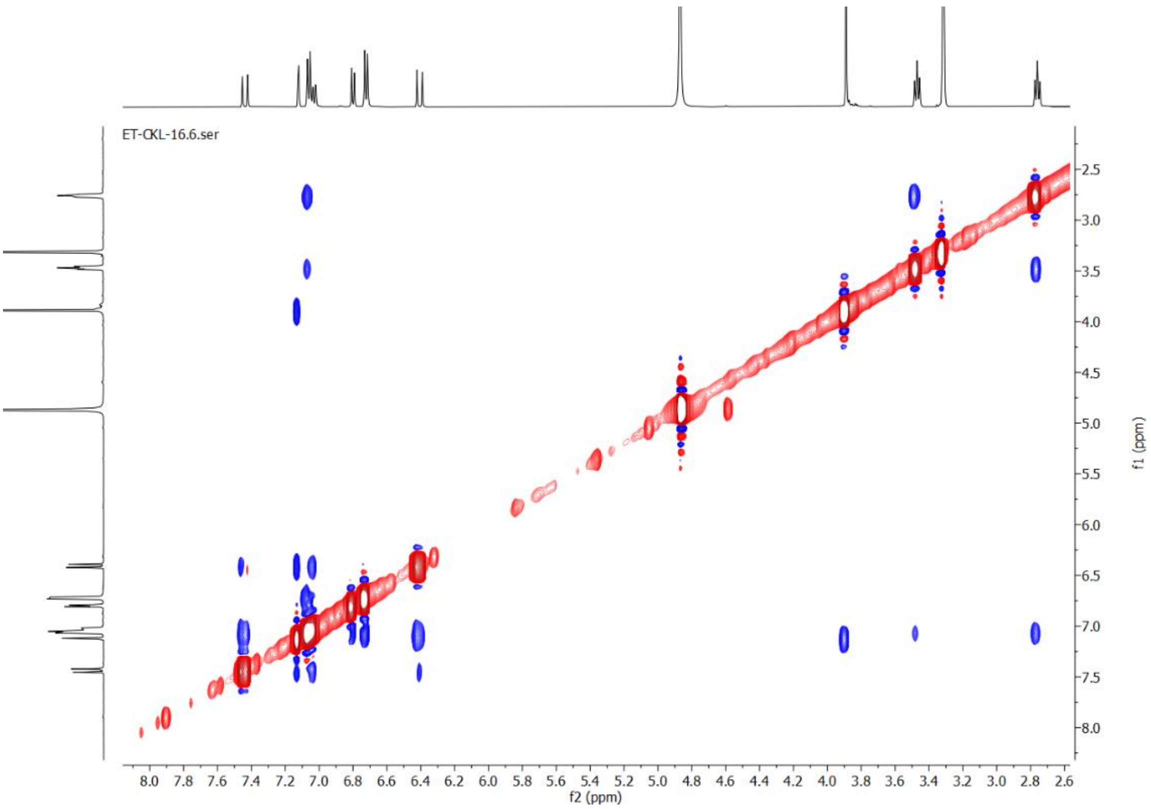

Figure S145. The NOESY spectrum of compound **19** (500 MHz, CD_3OD)

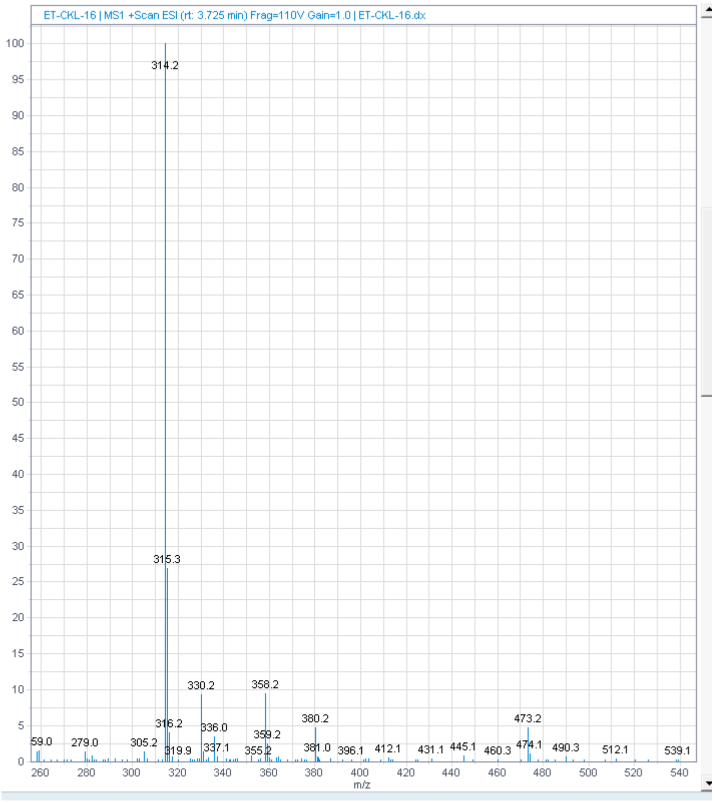

Figure S146. LC-MS of compound **19**

21. Other spectroscopic and physical data of known compounds (7-19)

ent-3β,19-dihydroxykaur-16-ene (**7**): $[\alpha]^{24}{}_{D}$ - 74 (c 0.07, $CHCl_3$); IR ν_{max} 3351, 2929, 1445, 1040, 873 cm^{-1}; LC-MS *m/z* 287.4 $[M-H_2O]^+$

ent-3β-hydroxy-kaur-16-en-19-oic acid (**8**): $[\alpha]^{24}{}_{D}$ - 50 (c 0.22, $CHCl_3$); IR ν_{max} 3422, 2860, 1704, 1448, 1388, 1245, 1149, 1081, 1037, 944, 873, 758 cm^{-1}; LC-MS *m/z* 301.5 $[M-H_2O]^+$

ent-16β,17-dihydroxy-kauran-19-oic acid (**9**)): $[\alpha]^{24}{}_{D}$ - 70 (c 0.05, $CHCl_3$); IR ν_{max} 3406, 2928, 2866, 1698, 1369, 1231 cm^{-1}; LC-MS *m/z* 335.3 $[M-H]^+$

ent-kaurane-3β, 16α, 17-triol (**10**): $[\alpha]^{24}{}_{D}$ -33 (c 0.32, $CHCl_3$); IR ν_{max} 3397, 2930, 1740, 1664, 1464, 1045, 757 cm^{-1}; LC-MS *m/z* 305.2 $[M-H_2O]^+$

ent-16β, 17-dihydroxykaurane (**11**): $[\alpha]^{24}{}_{D}$ -25 (c 0.12, $CHCl_3$); IR ν_{max} 3384, 2925, 2867, 2840, 1450, 1384, 1368, 1223, 1064, 1025, 877, 687 cm^{-1}; LC-MS *m/z* 289.4 $[M-H_2O]^+$

3β-hydroxy-kaur-16-eno (**12**): $[\alpha]^{24}{}_{D}$ + 6 (c 0.16, $CHCl_3$); IR ν_{max} 3309, 2926, 2854, 1656, 1443, 1384, 1367, 1094, 1039, 999, 873 cm^{-1}; LC-MS *m/z* 271.1 $[M-H_2O]^+$

3-*O*-methylquercetin (**13**): UV (MeOH) λ_{max} 254, 348 nm; IR ν_{max} 3354, 2930, 1652, 1607, 1439, 1281, 1258, 1211, 1170 cm^{-1}; LC-MS *m/z* 331.1 $[M+H]^+$

3,7,4′-tri-*O*-methylkaempferol (**14**): UV (MeOH) λ_{max} 267, 347 nm; IR ν_{max} 2921, 2850, 1712, 1658, 1600, 1583, 1496, 1455, 1428, 1441, 1258, 1174, 1093, 829, 812, 788 cm^{-1}; LC-MS m/z 329.2 $[M+H]^+$

3,7,3′,4′-tetra-*O*-methyl quercetin (**15**): UV (MeOH) λ_{max} 361, 348, 258 nm; IR ν_{max} 2915, 2839, 1761, 1652, 1603, 1588, 1418, 1312, 1152, 1125, 954, 717, 590, 572 cm^{-1}; LC-MS m/z 359.1

Ayanin (**16**): UV (MeOH) λ_{max} 255, 348, 359 nm; IR ν_{max} 3390, 2940, 2848, 1644, 1600, 1517, 1495, 1444, 1355, 1340, 821, 803, 640, 571, 562 cm^{-1}; LC-MS m/z 345.0 $[M+H]^+$

pinane-type monoterpenoid (**18**): $[\alpha]^{24}{}_{D}$ +36 (c 0.73, $CHCl_3$); IR ν_{max} 3410, 2928, 2874, 1711, 1472, 1408, 1388, 1371, 1327, 1305, 1270, 1195, 1031, 869, 839, 724, 587, 540 cm^{-1}; LC-MS m/z 167.1 $[M-H_2O]^+$

p-hydroxyphenylethyl ferute (**19**): IR ν_{max} 3340, 1653, 1591, 1514, 1450, 1269, 1124, 1032, 978, 822, 754 cm^{-1}; LC-MS m/z 315.5 $[M+H]^+$

22. X-ray crystallography of compound 1

Diffraction quality crystals of compound **1** were obtained by slow evaporation of a CH_2Cl_2 solution.

Crystal data for compound 1 (CCDC-2209300): $2(C_{20}H_{22}O_6)\cdot H_2O$, M = 734.77, colorless block, 0.18 × 0.31 × 0.46 mm, monoclinic, space group I2, a = 14.5703(2) Å, b = 8.6823(1) Å, c = 14.1008(2) Å, β = 103.077(1)°, V = 1737.54(4) $Å^3$, Z = 2, D_{calc} = 1.404 gcm^{-3}, F000 = 780, μ = 0.87 mm^{-1}, T = 120.0(1) K, θ_{max} = 76.3°, 3425 total reflections, 3392 with $I_o > 2\sigma(Io)$, R_{int} = 0.017, 3425 data, 243 parameters, 1 restraint, GooF = 1.06, 0.62 < dΔρ < 0.17 eÅ 3, $R[F^2 > 2\sigma(F^2)]$ = 0.031, $wR(F^2)$ = 0.084

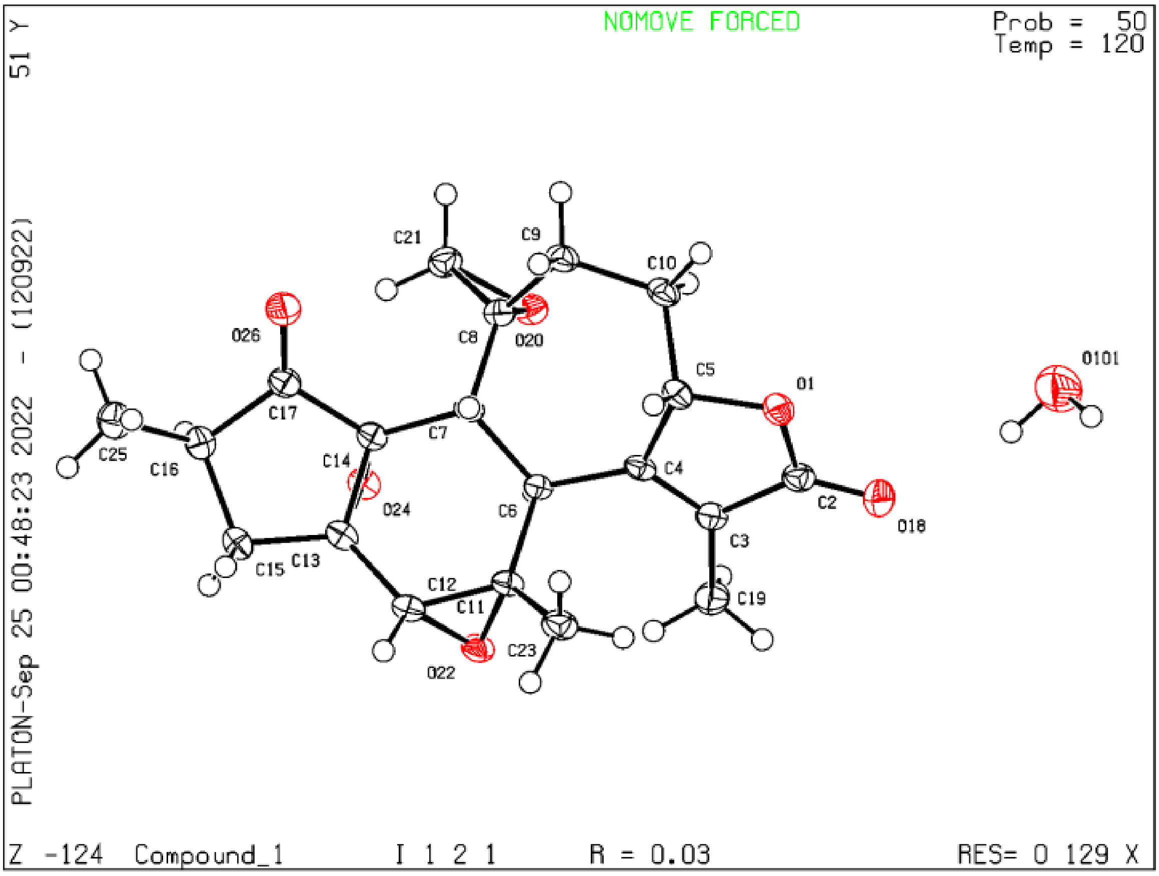

23. Antiviral, antibacterial and cytotoxicity data

Table S1. Anti-RSV activity and cytotoxicity for HEp-2 cells of selected compounds showing IC_{50}, CC_{50} and SI.

Compound	IC_{50}[a] (µM/µg/mL)	CC_{50}[b] (µM/µg/mL)	SI[c]
**1**	>100	>100	≥ 1.0
**2**	>100	>100	≥ 1.0
**4**	>100	>100	≥ 1.0
**5**	>100	>100	≥ 1.0
**7**	10.2	50.5	4.9
**11**	>100	>100	≥ 1.0
**14**	>100	>100	≥ 1.0
**15**	>100	>100	≥ 1.0
**16**	6.1	>100	> 16.4
Leaves crude	>20	>100	≥ 5.0
Stem crude	>20	>100	≥ 5.0

[a] Concentration of compound that inhibited the number of RSV plaques in HEp-2 cells by 50%

[b] Concentration of compound that reduced the viability of HEp-2 cells by 50%

[c] CC_{50}/IC_{50}

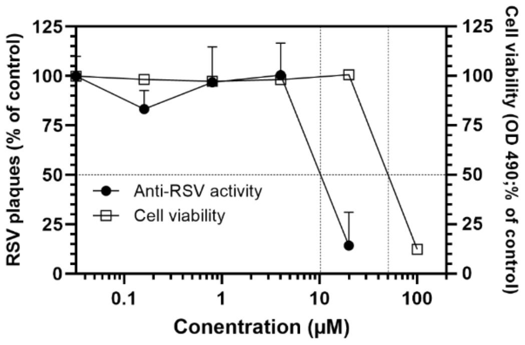

7

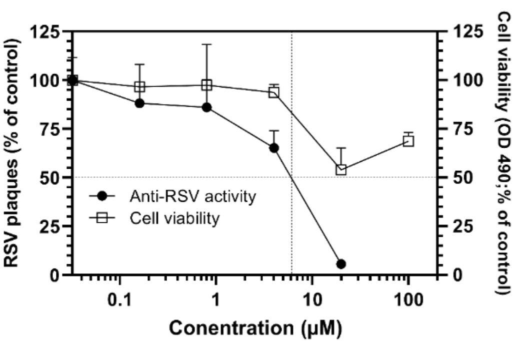

16

Figure S147. Anti-RSV dose response curve for compound **7** and **16.**

Table S2. Anti-HRV-2 activity and cytotoxicity for HeLa cells of selected compounds.

Compound	Anti-HRV-2 activity[a]							Cytotoxicity CC_{50} (µM)	SI
	Concentration (µM)						IC50[b] (µM)		
	100	20	4	0.8	0.16	0.0			
**2**	+[c]	-[d]	-	-	-	-	44.6	>100	>2.2
	+	-	-	-	-	-			
	+	-	-	-	-	-			
**5**	+	-	-	-	-	-	44.6	>100	>2.2
	+	-	-	-	-	-			
	+	-	-	-	-	-			
**16**	T[e]	C[f]	+	-	-	-	1.8	>100	55.6
	T	C	+	-	-	-			
	T	C	+	-	-	-			

[a] Antiviral activity determined by the reduction of the viral cytopathic effect in HeLa cells

[b] Calculated by the Reed and Muench formula for computation of 50% endpoint

[c] Protection against virus-induced cytopathic effect (>50% of cell monolayer protected)

[d] Lack of protection against virus-induced cytopathic effect

[e] Toxicity of compound for cells

[f] Cytostatic effect of compound for cells (poor cell growth accompanied by morphological alteration of cell shape)

Table S3. Antibacterial activity of selected isolated compounds and crude extracts. The concentration of the compounds tested for antibacterial activity is given in µg/µl. The results are given as the fluorescence mean normalized by the fluorescence of the positive control. The assay was performed in three independent replicates. For *B. subtilis* the cut off 0.1 whereas for *E. coli* 0.5 was applied, and compounds with higher values were considered as non-active against bacteria. Cells exposed to 3%v/v of DMSO were used as a positive control.

**Compound code**	***B subtilis* (mean)**	***E.coli* (mean)**	**Tested conc. (µg/µl)**
**1**	1.02 ± 0.13	0.62 ± 0.07	1.44
**2**	0.74 ± 0.09	0.56 ± 0.01	1.20
**4**	0.95 ± 0.06	1.16 ± 0.05	0.60
**5**	0.99 ± 0.03	1.23 ± 0.16	0.60
**7**	0.99 ± 0.07	1.25 ± 0.02	1.20
**11**	1.00 ± 0.09	1.11 ± 0.08	1.32
**14**	1.00 ± 0.07	1.04 ± 0.06	0.60
**16**	1.00 ± 0.07	1.02 ± 0.08	1.20
**15**	1.00 ± 0.07	0.76 ± 0.17	0.60
C. kilwae leave crude	0.89 ± 0.04	0.99 ± 0.14	0.30
C. kilwae stem crude	0.83 ± 0.02	1.45 ± 0.07	1.98

24. Antiplasmodial data

Table S3. Data from the in vitro assay growth inhibition of asexual blood stage *P. falciparum* (Dd2) for Crotofolanes.

Compounds	% Inhibition
Artesunate	100 ± 0.1
**1**	81 ± 24.0
**2**	100 ± 0.1
**3**	79 ± 18.5
**4**	42 ± 15.5
**5**	26 ± 8.4
**6**	60 ± 24.0

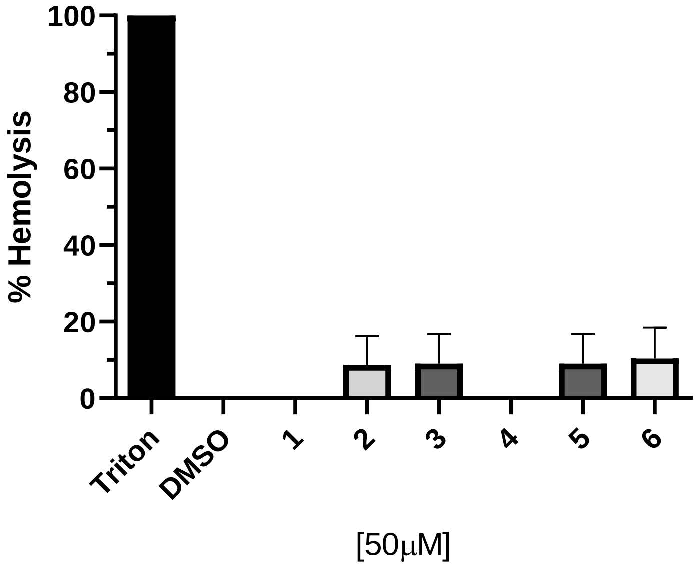

Figure S148. Hemolytic effects for crotofolanes at 50µM. Data from three independent experiments, as the average ± standard deviation. The hemolytic rate was calculated in relation to the hemolysis of erythrocytes in Triton X100 10% which was taken as 100%, after 4 hours of incubation.
